# SmI_2_/Sm‐Induced Reductive Silacyclization of Alkene/Diene Derivatives Using Dichlorosilanes or 1,2‐Dichlorodisilanes via Reductive Radical‐Polar Crossover

**DOI:** 10.1002/chem.202503424

**Published:** 2026-01-16

**Authors:** Zhengwei Chen, Daigo Kondo, Tsutomu Mizota, Leo Onishi, Huiying Mu, Koji Miki, Akiya Ogawa, Kouichi Ohe

**Affiliations:** ^1^ Department of Energy and Hydrocarbon Chemistry Graduate School of Engineering Kyoto University, Nishikyo‐Ku Kyoto Japan; ^2^ Organization For Research Promotion Osaka Metropolitan University Sumiyoshi Ward Osaka Japan

**Keywords:** chlorosilane, radical‐polar crossover, samarium reagent, silacyclization

## Abstract

The development of efficient strategies for constructing cyclic organosilicon frameworks is considered of great importance because of their structural diversity and synthetic utility. Herein, we report a SmI_2_/Sm‐mediated silacyclization of unsaturated organic compounds with readily available dichlorosilanes and ‐disilanes, enabling convenient access to 4−7‐membered silacyclic compounds. Mechanistic investigations indicate that the reaction involves silyl radical intermediates and proceeds via a reductive radical‐polar crossover (RRPCO) pathway. The present findings showcase the broad potential of Sm reagents in Si─C bond formation, providing a versatile strategy for constructing diverse silicon‐containing frameworks.

## Graphical abstract

A SmI_2_/Sm‐mediated silacyclization unlocks a concise route to 4–7‐membered silacycles from simple unsaturated substrates and dichlorosilanes/disilanes. The complementary functions of samarium reductants trigger a reductive radical–polar crossover, offering a versatile strategy for constructing multiple Si─C bonds efficiently.

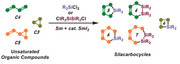

## 1 Introduction

Cyclic organosilicon compounds hav attracted considerable attention owing to their broad applications and largely untapped potential in synthetic organic chemistry [1, 2], drug design [3, 4], and materials science [5]. Substituting carbon frameworks with silicon analogues endows the molecules with distinctive chemical and physical characteristics. One prominent strategy for accessing silacycles involves the direct incorporation of silicon moieties into unsaturated organic substrates, typically using specific silylene precursors [6, 7, 8, 9, 10]. While impressive progress has been made, the limited availability of these precursors and their dependence on multistep synthesis still hinder their practical and bench‐top implementation. Accordingly, the development of a general silacyclization methodology that employs readily accessible silylene equivalents under mild conditions remains more imminent than ever.

**FIGURE 1:**
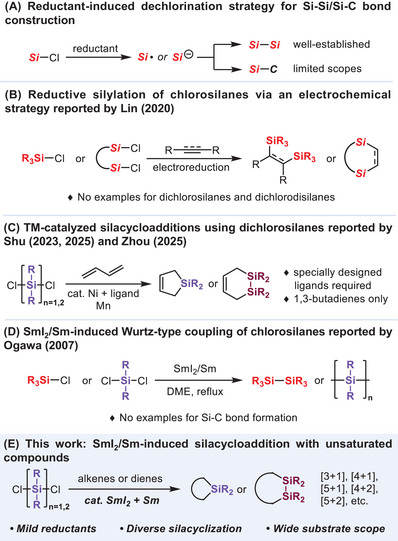
Strategies for reductive Si−Cl bond activation and their applications in Si─Si or Si─C bond formation.

Chlorosilanes are widely used as silylation reagents in laboratory and industrial settings. Beyond their electrophilicity, reductive Si─Cl bond activation offers access to nucleophilic silicon species, thereby enabling new approaches for Si─Si and Si─C bond formation (Figure 1A). The Kipping method has long been a cornerstone for the reductive cleavage of strong Si─Cl bonds of chlorosilanes [11], applying to oligosilane or polysilane synthesis [12, 13, 14]. Recently, the electrochemical reduction of chlorosilanes has emerged as an effective tool for the silylation of alkenes and Si─Si coupling reactions under mild conditions (Figure 1B) [15, 16]. Nevertheless, the electrolysis of a mixture of dichlorosilanes and butadienes mainly results in polysilane formation, while the desired silacycles are obtained only in low yields, even using a large excess of dienes [17]. Dichlorosilanes and dichlorodisilanes—considered as formal silylene or disilene equivalents with significant potential for the construction of diverse silacarbocycles—remain largely unexplored. Despite the remarkable progress in transition‐metal‐catalyzed silacycloadditions in terms of catalytic performance, these transformations still rely on specially designed ligands, and are currently limited to butadienes as trapping partners for silylene equivalents (Figure 1C) [18, 19, 20, 21, 22].

Because of its low toxicity and high chemoselectivity, SmI_2_ has been widely used in organic chemistry as a mild and efficient reductant [23, 24, 25]. Moreover, it is noted that the combination of SmI_2_ and Sm metal exhibits an even stronger reducing capability than either reagent alone, which has been exploited in various types of coupling reactions [26, 27, 28, 29, 30]. Early investigations showed that the SmI_2_/Sm system can reduce Si─Cl bonds, generating silyl radicals [31, 32, 33, 34] or silyl anions [35, 36] (silylenoid) as possible intermediates [37]. However, these reactions were exclusively limited to Si─Si coupling, including polysilane synthesis (Figure 1D). We envisaged that the potential of samarium reagents—as practical yet powerful reductants—has been far underestimated and remains largely underexplored in the Si─C bond formation. Herein, we describe a versatile silacyclization between dichlorosilanes/disilanes and a wide range of alkenes or dienes enabled by the SmI_2_/Sm system (Figure 1E).

## 2 Results and Discussion

**SCHEME 1:**
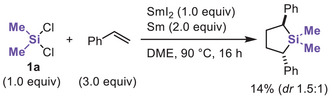
Reaction of dichlorodimethylsilane and styrene using the SmI_2_/Sm system.

We first tested the reaction of Me_2_SiCl_2_ (**1a**) and styrene using SmI_2_/Sm with the previously reported optimized ratio for the reduction of Si─Cl bonds. Interestingly, a mixture of 2,5‐diphenylsilacyclopentanes was obtained in a 14% NMR yield, along with the formation of polysilane, of which the molecular weight was not determined (Scheme 1 and Figures S1, S2). This observation can be rationalized by the insertion of styrene into the Si─C bond of an initially formed silirane derived from styrene, as reported by Seyferth [38]. Despite the low yield, this result demonstrated the successful trapping of SiMe_2_ equivalent with alkenes and inspired us to broaden the scope of unsaturated alkene derivatives for accessing a wide range of silacarbocycles.

We then carried out the reaction using **1a** and *(1E,3E)*‐1,4‐diphenyl‐1,3‐butadiene (**2a**), selected as a representative diene (Table 1). When SmI_2_ (0.3 equiv) and Sm (2.0 equiv) were used at 90°C in 1,2‐dimethoxyethane, silacyclopent‐3‐ene **3aa** was obtained in 76% yield with high *cis*‐selectivity (entry 1). The use of iodine and diiodoethane as additives resulted in diminished yields (entries 2 and 3). Using LiI instead of SmI_2_ led to markedly reduced yields, indicating that iodide is not responsible for the reactivity (entry 4). A lower yield of **3aa** was observed in the absence of SmI_2_, while only a trace amount of **3aa** was formed without Sm (entries 5 and 8). Notably, a catalytic amount of SmI_2_ (0.3 equiv) proved sufficient to provide a comparable yield relative to the stoichiometric amount (entries 1, 6, and 7). Lowering the reaction temperature decreased the yield of **3aa** (entry 9).

**TABLE 1:** Optimization and control experiments.

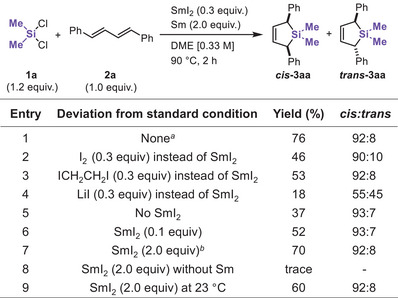

^a^ Me_2_SiCl_2_ **1a** (0.6 mmol), diene **2a** (0.5 mmol), and 1,2‐dimethoxyethane (DME, 1.5 mL).

^b^ Use of SmI_2_ in 10 mL DME (0.1 M).

With the optimized conditions established, we explored the substrate scope of the reaction. The results are summarized in Table 2. The generality of SiR_2_ was first examined by using several commercially available dichlorosilanes **1**. Alkyl‐ and aryl‐substituted dichlorosilanes could react with **2a** to afford silacyclopent‐3‐enes in 62%–76% yields (**3aa−3fa**) with high *cis‐*selectivity. The high diastereoselectivity can be explained by the allylic strain of the plausible intermediate (*vide infra* and Scheme S3). Next, we examined the scope of 1,3‐dienes **2** using Me_2_SiCl_2_. Diphenyl substituted butadienes with different substitution patterns also delivered the desired products in good yields (**3ab, 3ac**, and **3ad**). Mono‐phenyl‐substituted dienes afforded **3ae** (52%) and **3af** (89%). We further examined 2‐aryl‐substituted dienes bearing a naphthyl, benzodioxolyl, or ferrocenyl group, providing **3ag**, **3ah**, and **3ai** in 66%, 57%, and 71% yields, respectively. *para*‐Fluoro (**2j**) and chloro (**2k**) substituents on 2‐aryl were tolerated under the reaction conditions, yielding **3aj** and **3ak** in 65% and 31% yields without dehalogenation. 2‐Aryl‐1,3‐dienes with methoxy (**2l**) or *N,N*‐dimethylamino (**2m**) groups furnished silacyclopentene **3al** and **3am** in 64% and 78% yields, respectively. When two diene units were present, both could react to give **3an** in 40% yield. Fused silacarbocycle **3ao** and **3co** were obtained in 81% and 40% yields, and the synthesis of **3ao** was scalable to 3 mmol. Meanwhile, conjugated triene **2p** produced **3ap** as a sole product in modest yield, with no [6+1] product being obtained. Aliphatic dienes (**2q–2u**) were also compatible: the reactions of 2‐cyclohexyl‐ (**2q**) and 2‐adamantyl‐1,3‐butadiene (**2r**) furnished **3aq** in 45% yield and **3ar** in 80% yield. Moreover, the silacyclization was applicable to natural products such as myrcene (**2s**) or dienes derived from *β*‐ionone (**2t**) and pregnenolone (**2u**), giving **3as–3au** in 52%–70% yields. Notably, silacyclization of **2t** occurred selectively at the least substituted diene unit.

**TABLE 2:** Scope of [4+1] Silacyclization^a^.

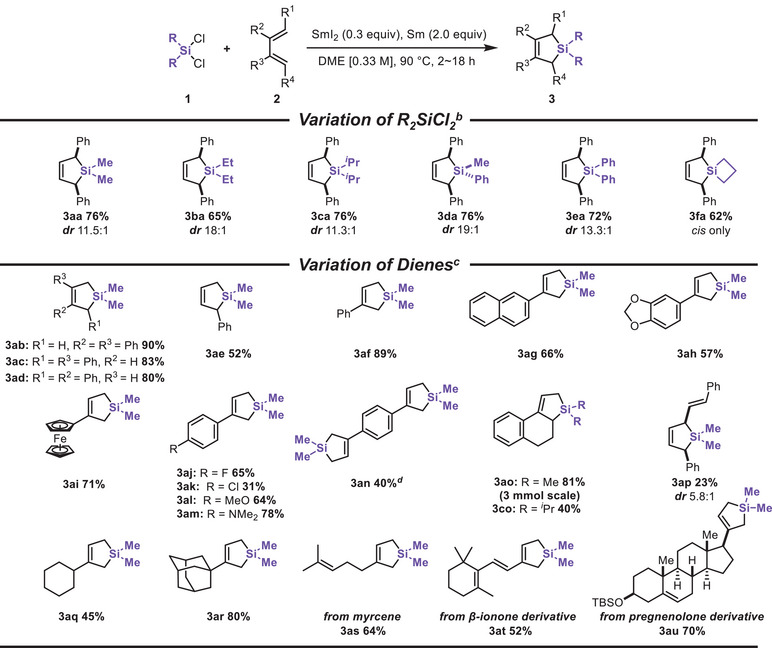

^a^ Reactions were conducted using dichlorosilanes **1** (1.2 equiv) and 1,3‐diene **2** (0.2‐3.0 mmol, 1.0 equiv).

^b^ (1*E*,3*E*)‐1,4‐Diphenyl‐1,3‐butadiene **2a** was used.

^c^ Me_2_SiCl_2_ **1a** was used.

^d^ **1a** (2.4 equiv) was used.

Cyclic disilanes bearing a Si─Si bond are valuable motifs for constructing large‐size cyclic organosilanes [39, 40, 41], yet the conventional methodologies for their synthesis rely strongly on alkali metals or organolithium reagents [42, 43]. In comparison with silacyclopent‐3‐enes, which are accessible via silylene transfer from specially designed precursors, the synthesis of 1,2‐disilacyclohex‐4‐enes remains challenging because of the few precursors available for “disilene” transfer [44]. Most recently, the Shu group [20] reported a Ni‐catalyzed [4+2] silacycloaddition using 1,3‐dienes and dichlorodisilanes, which are critically enabled by the use of phosphinooxazoline ligands. We envisioned that the corresponding transformation could be achieved under the reaction conditions using the SmI_2_/Sm system. Interestingly, 1,2‐disilacyclohex‐4‐enes were obtained in good yields (63%–98%) with excellent *cis‐*selectivity, while the weak Si─Si bonds remained intact (Table 3).

**TABLE 3:** Scope of [4+2] Silacyclization^a^.

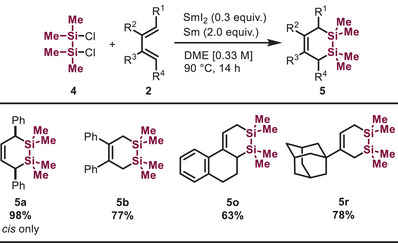

^a^ Reactions were conducted using 1,2‐dichloro‐1,1,2,2‐tetramethyldisilane **4** (1.2 equiv) and 1,3‐diene **2** (0.5‐1.0 mmol, 1.0 equiv).

A series of silacyclization reactions based on 1,3‐dienes was realized, motivating a deeper investigation into the actual reaction intermediate in this system. Ohmura and Suginome [45] reported that silacyclopent‐2‐enes can be formed through the isomerization of silirane derived from the reaction of **2a** and aminoborylsilane as a silylene precursor. Therefore, the selective formation of **3aa** in our system may constitute evidence against the intermediacy of a silylene. Considering the single‐electron reducing ability of SmI_2_, we postulated that a silyl radical [31, 32, 33, 34] could be generated *in situ* and further undergo addition to alkenes or dienes to give carbon‐centered radical intermediates. Thus, we examined the ring‐opening radical clock reaction using vinylcyclopropanes (VCPs), which have served as versatile C5 synthons in organic synthesis, significantly contributing to the construction of different ring systems [46, 47]. For example, the Oestreich group has reported a pioneering work on silylium‐ion‐promoted [5+1] cyclization using hydrosilanes, which gives saturated silacyclohexanes [48]. We found that *α*‐cyclopropylstyrene is an essential motif, acting as a C5 synthon to afford silacyclohex‐3‐enes and disilacyclohept‐4‐enes using dichlorosilanes or 1,2‐dichlorodisilanes with the SmI_2_/Sm system (Table 4). In contrast to conjugated dienes, VCPs exhibit lower reactivity under the present conditions, and higher loadings of samarium and dichlorosilanes are therefore required to achieve complete substrate conversion. The lack of a phenyl group on cyclopropane slightly decreased the yields of silacycles (**7a**). Vinylcyclopropane substrates bearing *para‐* (**7e**) and *ortho*‐tolyl VCP (**7f**) furnished silacyclohex‐3‐enes in good yields. Fluoro‐ (**7** **g**), dimethylamino (**7** **h**), and dimethoxy (**7i**) groups were well tolerated, producing the corresponding silacycles in good yields. Despite the low yield, 1,2‐disilacyclohept‐4‐enes **8a‐c** were obtained from VCP and 1,2‐dichlorodisilane **4**. The diminished yields are likely attributable to the competitive formation of silicon‐containing byproducts, including hexamethyldisilane, as well as other higher‐molecular‐weight or structurally complex species. These results demonstrate that the SmI_2_/Sm‐mediated reductive silacyclization can efficiently construct silacycles of varying ring sizes.

**TABLE 4:** Scope of [5+1] and [5+2] Silacyclization^a^.

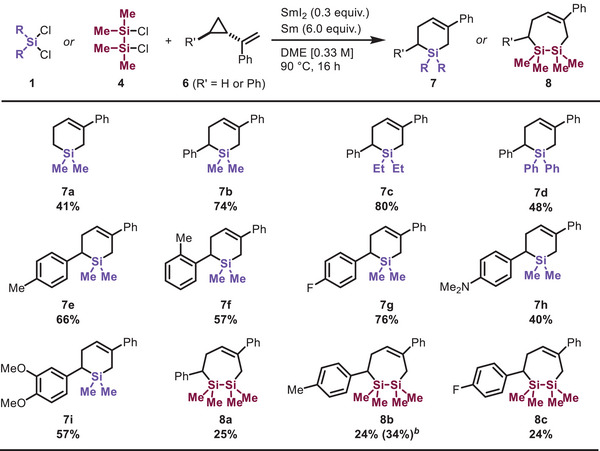

^a^ Reactions were conducted using dichlorosilanes **1** (4.0 equiv) or 1,2‐dichloro‐1,1,2,2‐tetramethyldisilane **4** (4.0 equiv) with VCPs **6** (1.0 equiv).

^b^ 8.0 equiv of **4** and 8.0 equiv of Sm were used at 110°C.

The reaction results with VCP indicate the intervention of a silyl radical intermediate generated through the reaction of R_2_SiCl_2_ or R_2_ClSiSiClR_2_ with SmI_2_/Sm. Given the nucleophilicity of silyl radicals, it can be inferred that the addition of silyl radicals to alkenes or 1,3‐butadienes is a crucial step in the reaction. However, the reaction using VCP without a phenyl group on the alkene moiety does not produce the desired product (Scheme S5). We considered that conjugated dienes or aryl‐substituted alkenes reduce the LUMO level and enable the reaction.

**SCHEME 2:**
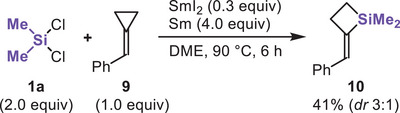
Reaction of dichlorodimethylsilane and benzylidenecyclopropane using the SmI_2_/Sm system.

When benzylidenecyclopropane **9** and Me_2_SiCl_2_ were reacted with SmI_2_/Sm, benzylidenesilacyclobutane **10** was obtained in 41% yield (Scheme 2). These findings suggest the involvement of silyl radical intermediates in the SmI_2_/Sm system and provide a new entry to silacyclobutanes, which are valuable building blocks in silicon‐containing compound synthesis [49, 50].

**FIGURE 2:**
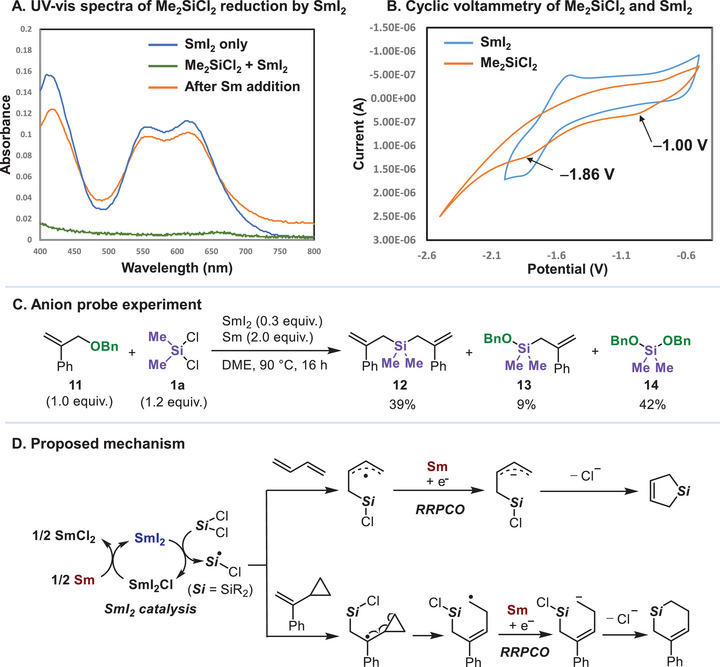
Mechanistic investigations. **A**: UV‐vis spectra of 1.0 mM SmI_2_ (blue), 1.0 mM SmI_2_ with 1.0 mM Me_2_SiCl_2_ at 90°C for 16 h (green) and the reaction mixture after addition of 2.0 equiv Sm powder addition (orange). **B**: Cyclic voltammograms of 2.5 mM SmI_2_ (blue) and 2.5 mM Me_2_SiCl_2_ (orange) with 0.01 M TBAPF_6_ in degassed THF at 50 mV/s. **C**: An anion probe experiment using allyl benzyl ether **11**. **D**. Summary of a proposed mechanism.

The unique reactivity arising from the SmI_2_/Sm system prompted us to investigate its function [51]. The UV‐vis spectra of a SmI_2_ with **1a**, heated at 90°C for 16 h, indicate that SmI_2_ reduces Si─Cl bonds, as evidenced by the disappearance of characteristic SmI_2_ absorbance peaks at 550 and 630 nm upon the addition of **1a** (Figure 2A). These specific peaks of SmI_2_ were recovered when Sm powder was added, indicating that Sm^3+^ is reduced back to Sm^2+^ by metallic Sm (Figure 2A) [30, 52, 53, 54]. These findings are in accordance with earlier reports on the electrochemical behaviors of chlorosilanes [55, 56, 57]. We further conducted the cyclic voltammetry of Me_2_SiCl_2_, detecting two irreversible signals corresponding to the sequential 1e^–^ reductions of the Si─Cl bonds [58]. The first cathodic potential was observed at −1.00 V, suggesting that Me_2_SiCl_2_ can indeed undergo one‐electron reduction by SmI_2_ (−1.55 V) [59, 60], although it remains ambiguous whether SmI_2_ is involved in the second reduction of the silicon species (Figure 2B).

To examine the necessity of both SmI_2_ and Sm in silacycle formation, we conducted reactions using butadiene **2a** or vinylcyclopropane **6b** without SmI_2_ or Sm (Tables S1 and S2). In the absence of SmI_2_, the reaction of **1a** with **2a** proceeded to some extent, while the reaction with **6b** did not afford the target silacycle. We suppose that Sm, being a stronger reductant than SmI_2_, can overreduce the Si─Cl bonds via a 2e^–^ pathway to afford silyl anions rather than radicals. These anions subsequently undergo conjugate addition to dienes to give silacyclopent‐3‐enes [17], whereas no [5+1] silacyclization product was formed with VCP. When Sm was omitted, no products were obtained in each case, which indicates that Sm plays a pivotal role in the cyclization process. We considered that silyl radicals add to alkenes to generate carbon‐centered radicals, which could be subsequently reduced to carbanions via a reductive radical‐polar crossover (RRPCO) pathway [61]. Allyl radicals (−1.99 V) [62] and benzyl radicals (−2.20 V) [63] can only be reduced by Sm (−2.62 V) [64], not by SmI_2_ (−1.55 V). To support this hypothesis further, we carried out the reaction of **1a** and allyl benzyl ether **11**, which can rapidly release BnO^–^ through *β*‐elimination from *β*‐carbanion [15]. The generated BnO^–^ is then trapped by Me_2_SiCl_2_ and the postulated intermediate ClMe_2_SiCH_2_C(Ph) = CH_2_, which ultimately gives **14** and **13**, along with adduct **12** (Figure 2C). These results clearly support the proposed RRPCO pathway and were further corroborated by the natural population charge analysis (NPA) from computational studies (Figure S6).

A plausible mechanism of the current reactions is depicted in Figure 2D. Dichlorosilanes are reduced by SmI_2_ to generate a silyl radical such as ClR_2_Si•[65], which subsequently adds to alkene or 1,3‐butadienes to form carbon‐centered radical intermediates, with possible contribution from the *β*‐silicon‐effect (Scheme S5) [66, 67]. Further single‐electron reduction with Sm yields the anionic intermediate, which may be further stabilized through the interaction with the Si─Cl σ* orbital (Scheme S7), ultimately leading to silacyclization via an RRPCO process.

**FIGURE 3:**
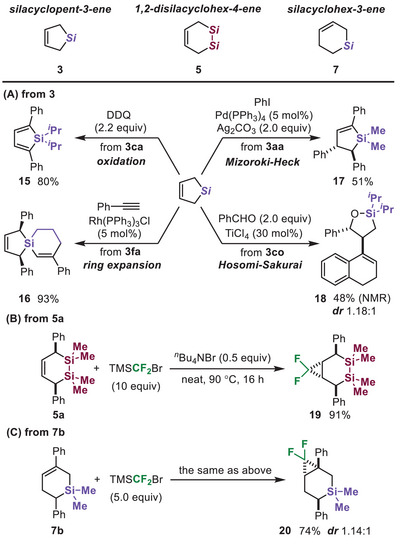
Derivatization of silacarbocycles.

Finally, we demonstrated the derivatization of the silacarbocycles (Figure 3). DDQ oxidation [10] of silacyclopent‐3‐ene **3ca** afforded silole **15** in 80% yield. The ring expansion of silacyclobutane in **3fa** by rhodium catalysis [68] afforded spirosilane **16** in 93% yield. Mizoroki–Heck‐type phenylation [69] was applicable to the derivatization of **3aa**, affording **17**. Taking advantage of a cyclic allylsilane structure, the Hosomi–Sakurai reaction of **3co** afforded 1‐oxa‐2‐silacyclopentene **18** in 48% yield. Moreover, difluorocyclopropanation of 1,2‐disilacyclohex‐4‐ene **5a** and silacyclohex‐3‐ene **7b** according to the reported method [70, 71] afforded **19** and **20** in good yields. These derivatizations highlight the versatility and synthetic potential of silacarbocycles, providing access to structurally diverse and functionally valuable silicon‐containing compounds.

## 3 Conclusion

In summary, we have discovered a novel silacyclization of conjugated dienes, VCPs, and methylenecyclopropanes with dichlorosilanes by SmI_2_/Sm, enabled by the independent role of samarium reductants. This reaction provides a convenient route to cyclic organosilanes of various ring sizes and demonstrates that an RRPCO process can be triggered through the reductive umpolung of electrophilic dichlorosilanes/disilanes, effectively exploiting the differences in reduction potentials among multiple reductants. This study not only introduces a new synthetic strategy in organosilicon chemistry, but also highlights the unique capability of mixed Sm reductants to promote multiple Si─C bond formations simultaneously under reductive conditions.

## Conflicts of Interest

The authors declare no conflict of interest.

## Supporting information




**Supporting File 1**: chem70674‐sup‐0001‐suppmat.pdf
