# Supplementary material for: SmI2/Sm‐Induced Reductive Silacyclization of Alkene/Diene Derivatives Using Dichlorosilanes or 1,2‐Dichlorodisilanes via Reductive Radical‐Polar Crossover
Source: Chemistry. 2026 Jan 16;32(12):e03424. doi: 10.1002/chem.202503424 (PMC13037352; doi:10.1002/chem.202503424)
Supplement: Supplementary file 1 — Supporting File 1: chem70674‐sup‐0001‐suppmat.pdf [file CHEM-32-e03424-s001.pdf]

SmI_2/Sm-Induced Reductive Silacyclization of Alkene/Diene Derivatives Using Dichlorosilanes or 1,2-Dichlorodisilanes via Reductive Radical-Polar Crossover

Zhengwei Chen,[a] Daigo Kondo,[a] Tsutomu Mizota,[a] Leo Onishi,[a] Huiying Mu,[a] Koji Miki,[a] Akiya Ogawa,[b] Kouichi Ohe*,[a]

[a]*Department of Energy and Hydrocarbon Chemistry, Graduate School of Engineering, Kyoto University, Katsura, Nishikyo-ku, Kyoto 615-8510, Japan*

[b]*Organization for Research Promotion, Osaka Metropolitan University, Sumiyoshi Ward, Osaka 558-0022, Japan*

Email: ohe@scl.kyoto-u.ac.jp

1. General information S1

2. Preparation of substrates S1

3. SmI_2/Sm-induced [4+1]-silacyclization using dienes and dichloro-silanes S1

4. SmI_2/Sm-induced [4+2]-silacyclization using dienes and 1,2-dichloro-tetramethyldisilane S14

5. SmI_2/Sm-induced [5+1]-silacyclization using vinylcyclopropanes and dichlorosilanes S16

6. SmI_2/Sm-induced [3+1]-silacyclization of benzylidenecyclopropane 9 and dichlorosilane 1a S23

7. Control experiments and mechanistic studies S24

8. Derivatization of Silacarbocycles S45

9. References S50

10. Copies of ^{1}H, ^{13}C and ^{29}Si NMR spectra S53

1. General information

NMR spectra were recorded on JEOL ECZ-400, JEOL ECX-400 (400 MHz for ^{1}H NMR, 100 MHz for ^{13}C NMR and 79 MHz for ^{29}Si NMR) and JEOL JNM-ECZ 600R (600 MHz for NOE). Chemical shifts are recorded in δ ppm referenced to $CDCl_3$ (δ 7.26 for ^{1}H NMR and δ 77.10 for ^{13}C NMR). ^{29}Si NMR spectra were calibrated using a tetramethylsilane standard at 0.00 ppm. Melting points (m.p.) were obtained using Micro Melting Pointapparatus (Yanaco) and the values are uncorrected. IR spectra were collected using Spectrum Two (Perkin Elmer) in cm^{-1}. HRMS was performed on Thermo Fisher Scientific Exactive Plus (ESI, APCI and DART).

Column chromatography was performed using silica gel (230-400 mesh) purchased from Silicycle (Canada). Reverse-phase column chromatography was performed using Wakogel 50C18 silica gel (38-63 μm, Wako Pure Chemical Industries). Gel permeation chromatography (GPC) was performed on a recycling preparative HPLC (Japan Analytical Industry, LC-5060) with a JAIGEL-2HR column.

Anhydrous 1,2-dimethoxyethane (DME) was purchased from Sigma-Aldrich. Chlorosilanes **1a**-**1f**, 1,2-dichloro-1,1,2,2-tetramethyldisilane **4**, samarium powder (99.9% REO, 40 mesh) was purchased from Strem. 1,2-Diiodoethane and 1,3-dienes **2a**, **2p**, **2s** were purchased from Tokyo Chemical Industry or Sigma-Aldrich and used without further purification.

2. Preparation of substrates

1,3-Dienes **2b**,[1] **2c**,[2] **2d**,[3] **2e**,[4] **2f**-**2o**,[5] **2q**,[5] **2r**,[5] **2t**,[5] **2u**[5], vinyl cyclopropane **6a**,[6] **6b-g**,[7] and benzylidenecyclopropane **9**[8] were prepared following reported procedures.

3. SmI_2/Sm-induced [4+1]-silacyclization using dienes and dichloro-silanes

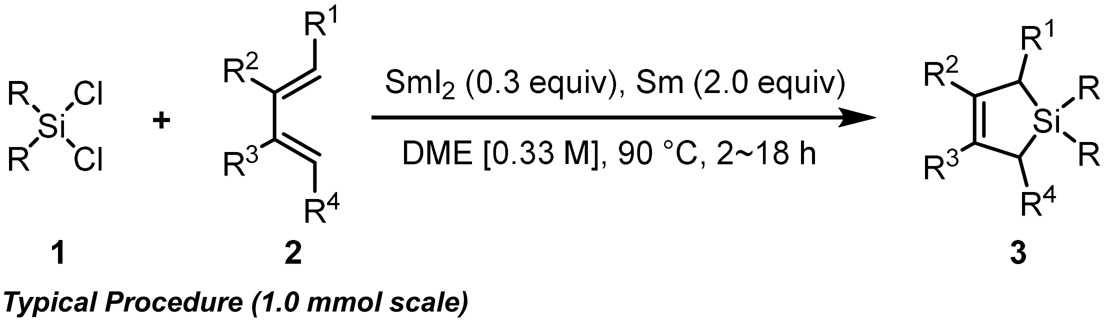

Typical Procedure (1.0 mmol scale)

To a flame-dried Schlenk tube were added samarium powder (2.3 mmol, 345.0 mg), 1,2-diiodoethane (0.3 mmol, 84.6 mg) and anhydrous 1,2-dimethoxyethane (3.0 mL) under nitrogen atmosphere. The reaction mixture was stirred vigorously at room temperature for 40 min until a deep blue suspension was formed. 1,3-Diene **2** (1.0 mmol) and dichlorosilane **1** (1.2 mmol) were added dropwise to the mixture under nitrogen atmosphere sequentially. The mixture was stirred at 90 °C for 2-18 hours until diene was all consumed (checked by

TLC or GC-MS). After cooling to room temperature, the mixture was carefully quenched by saturated aq. NH_4Cl solution (5.0 mL) and extracted with diethyl ether (3 × 25 mL). The organic layer was dried over $MgSO_4$ or Na_2SO_4. The solvent was removed under vacuum to afford the crude residue, which was subjected to silica gel column chromatography (hexane) to afford pure silacyclopent-3-enes **3**.

***cis*-1,1-dimethyl-2,5-diphenyl-1-silacyclopent-3-ene (3aa)**

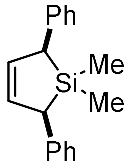

3aa

Following the typical procedure using *(1E,3E)*-1,4-diphenyl-1,3-butadiene **2a** (1.0 mmol, 206.0 mg, 1.0 equiv.), dichlorodimethylsilane **1a** (1.2 mmol, 154.9 mg, 1.2 equiv.), samarium powder (2.0 mmol, 300.7 mg) and freshly prepared SmI_2 (0.3 mmol in 3.0 mL DME). Reaction time: 2 h. Purification by column chromatography (hexane) using silica gel afforded pure **3aa** as a colorless liquid (*dr* = 11.5:1, 201.0 mg, 76%). All spectroscopic data matched those reported in the literature.[2]

***cis*-1,1-diethyl-2,5-diphenyl-1-silacyclopent-3-ene (3ba)**

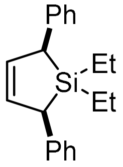

3ba

Following the typical procedure using *(1E,3E)*-1,4-diphenyl-1,3-butadiene **2a** (0.5 mmol, 103.0 mg, 1.0 equiv.), dichlorodiethylsilane **1b** (0.6 mmol, 94.3 mg, 1.2 equiv.), samarium powder (1.0 mmol, 150.4 mg) and freshly prepared SmI_2 (0.15 mmol in 1.5 mL DME). Reaction time: 2.5 h. Purification by column chromatography (hexane) using silica gel afforded pure **3ba** as a white solid (*dr* = 18:1, 94.9 mg, 65%). mp: 37.8-38.5 °C.

^{1}H NMR (400 MHz, $CDCl_3$): δ 7.30-7.26 (m, 4H), 7.14-7.10 (m, 6H), 6.14 (s, 2H), 3.37 (s, 2H), 1.22 (t, J = 7.8 Hz, 3H), 0.96 (d, J = 7.8 Hz, 2H), 0.14 (t, J = 7.8 Hz, 3H), –0.03 (d, J = 7.8 Hz, 2H).

^{13}C NMR (100 MHz, $CDCl_3$): δ 143.4, 135.2, 128.3, 126.6, 124.3, 37.8, 7.8, 5.6, 3.7, 1.3.

^{29}Si NMR (79 MHz, $CDCl_3$): δ 25.9.

Anal. Calcd. For $C_{20}H_{24}Si$: C, 82.13; H, 8.27. Found: C, 82.35; H, 8.40.

cis-1,1-diisopropyl-2,5-diphenyl-1-silacyclopent-3-ene (3ca)

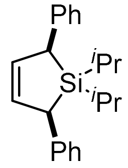

3ca

Following the typical procedure using *(1E,3E)*-1,4-diphenyl-1,3-butadiene **2a** (0.5 mmol, 103.0 mg, 1.0 equiv.), dichlorodiisopropylsilane **1c** (0.6 mmol, 111.1 mg, 1.2 equiv.), samarium powder (1.0 mmol, 150.4 mg) and freshly prepared SmI_2 (0.15 mmol in 1.5 mL DME). Reaction time: 2 h. Purification by column chromatography (hexane) using silica gel afforded pure **3ca** as a white solid (*dr* = 11.3:1, 122.0 mg, 76%). mp: 46.5-47.5 °C.

A mixture of two diastereomeric isomers were observed. The concentration of minor isomer (*trans*-isomer) was too dilute to be observed in ^{13}C and ^{29}Si NMR.

^1H NMR (400 MHz, $CDCl_3$):

cis-isomer (major): δ 7.29-7.25 (m, 4H), 7.19-7.15 (m, 4H), 7.13-7.09 (m, 2H), 6.14 (s, 2H), 3.34 (s, 2H), 1.32 (sep, *J* = 6.0 Hz, 1H), 1.23 (d, *J* = 6.0 Hz, 6H), 0.65 (sep, *J* = 7.4 Hz, 1H), 0.26 (d, *J* = 7.4 Hz, 6H).

trans-isomer (minor): 7.29-7.25 (m, 4H), 7.19-7.15 (m, 4H), 6.19 (*trans*-isomer, d, *J* = 0.9 Hz, 2H), 3.52 (*trans*-isomer, d, *J* = 0.9 Hz, 2H), 1.35 (*trans*-isomer, sep, *J* = 6.0 Hz, 2H), 0.72 (*trans*-isomer, d, *J* = 6.0 Hz, H).

^{13}C NMR (100 MHz, $CDCl_3$): δ 143.7, 135.6, 128.3, 127.3, 124.5, 36.8, 18.6, 17.2, 11.9, 11.0.

^{29}Si NMR (79 MHz, $CDCl_3$): δ 23.1.

Anal. Calcd. For $C_{22}H_{28}Si$: C, 82.43; H, 8.80. Found: C, 82.55; H, 9.01.

cis-1-methyl-1,2,5-triphenyl-1-silacyclopent-3-ene (3da)

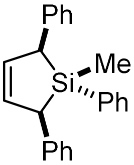

3da

Following the general procedure using *(1E,3E)*-1,4-diphenyl-1,3-butadiene **2a** (0.5 mmol, 103.0 mg, 1.0 equiv.), dichloromethylphenylsilane **1d** (0.6 mmol, 114.7 mg, 1.2 equiv.), samarium powder (1.0 mmol, 150.4 mg) and freshly prepared SmI_2 (0.15 mmol in 1.5 mL DME). Reaction time: 5 h. Purification by column chromatography (hexane) using silica gel afforded pure **3da** as a white solid (*dr* = 19:1, 123.9 mg, 76%). mp: 72.5-73.5 °C.

The scrambling of Me and Ph substituents on the Si atom may take place upon column chromatography on silica gel or during extended storage. The configuration of the pure product was determined by NOE NMR experiments.

^1H NMR (400 MHz, $CDCl_3$): δ 7.66-7.64 (m, 2H), 7.46-7.43 (m, 3H), 7.26-7.23 (m, 4H),

7.11-7.06 (m, 6H), 6.22 (s, 2H), 3.61 (s, 2H), −0.39 (s, 3H).
^{13}C NMR (100 MHz, CDCl$_3$): δ 143.0, 136.2, 135.1, 134.0, 129.8, 128.4, 128.2, 126.7, 124.5, 39.2, −8.3.
^{29}Si NMR (79 MHz, CDCl$_3$): δ 14.0.
Anal. Calcd. For $C_{23}H_{22}Si$: C, 84.61; H, 6.79. Found: C, 84.58; H, 6.83.

***cis*-1,1,2,5-tetraphenyl-1-silacyclopent-3-ene (3ea)**

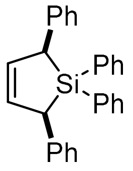

3ea

Following the typical procedure using *(1E,3E)*-1,4-diphenyl-1,3-butadiene **2a** (0.5 mmol, 103.0 mg, 1.0 equiv.), dichlorodiphenylsilane **1f** (0.6 mmol, 151.9 mg, 1.2 equiv.), samarium powder (1.0 mmol, 150.4 mg) and freshly prepared SmI_2 (0.15 mmol in 1.5 mL DME). Reaction time: 5 h. Purification by column chromatography (hexane) using silica gel afforded pure **3ea** as a white solid (*dr* = 13.3:1, 139.7 mg, 72%). mp: 107.1-107.9 °C.
^{1}H NMR (400 MHz, CDCl$_3$): δ 7.76-7.73 (m, 2H), 7.50-7.45 (m, 3H), 7.17-7.13 (m, 4H), 7.11-7.01 (m, 6H+1H), 6.82 (t, *J* = 7.8 Hz, 2H), 6.55 (d, *J* = 7.8 Hz, 2H), 6.36 (s, 2H), 3.87 (s, 2H).
^{13}C NMR (100 MHz, CDCl$_3$): δ 142.3, 136.2, 135.7, 135.1, 135.0, 130.2, 129.9, 129.0, 128.2, 128.2, 127.3, 126.7, 124.7, 39.7.
^{29}Si NMR (79 MHz, CDCl$_3$): δ 6.9.
Anal. Calcd. For $C_{28}H_{24}Si$: C, 86.55; H, 6.23. Found: C, 86.37; H, 6.39.

***cis*-5,8-diphenyl-4-silaspiro[3.4]oct-6-ene (3fa)**

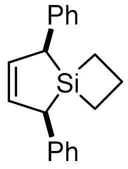

3fa

Following the typical procedure using *(1E,3E)*-1,4-diphenyl-1,3-butadiene **2a** (1.0 mmol, 206.0 mg, 1.0 equiv.), 1,1-dichlorosilacyclobutane **1f** (1.2 mmol, 169.3 mg, 1.2 equiv.), samarium powder (2.0 mmol, 300.7 mg) and freshly prepared SmI_2 (0.3 mmol in 3.0 mL DME). Reaction time: 3 h. Purification by column chromatography (hexane) using silica gel afforded pure **3fa** as a white solid (172.0 mg, 62%). mp: 39.0-40.0 °C.
^{1}H NMR (400 MHz, CDCl$_3$): δ 7.32-7.28 (m, 4H), 7.15-7.09 (m, 6H), 6.13 (s, 2H), 3.46 (s, 2H), 1.68 (tt, *J* = 8.2 Hz, 2H), 1.40 (t, *J* = 8.2 Hz, 2H), 0.44 (t, *J* = 8.2 Hz, 2H).
^{13}C NMR (100 MHz, CDCl$_3$): δ 143.1, 134.9, 128.5, 126.8, 124.7, 40.1, 17.9, 14.2, 12.2.
^{29}Si NMR (79 MHz, CDCl$_3$): δ 42.0.

Anal. Calcd. For $C_{19}H_{20}Si$: C, 82.55; H, 7.29. Found: C, 82.48; H, 7.42.

1,1-dimethyl-2,3-diphenyl-1-silacyclopent-3-ene (3ab)

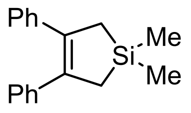

3ab

Following the typical procedure using 2,3-diphenyl-1,3-butadiene **2b** (1.0 mmol, 206.0 mg, 1.0 equiv.), dichlorodimethylsilane **1a** (1.2 mmol, 154.8 mg, 1.2 equiv.), samarium powder (2.0 mmol, 300.7 mg) and freshly prepared SmI_2 (0.3 mmol in 3.0 mL DME). Reaction time: 2 h. Purification by column chromatography (hexane) using silica gel afforded pure **3ab** as a white wax (237.6 mg, 90%). All spectroscopic data matched those reported in the literature.[2]

1,1-dimethyl-2,4-diphenyl-1-silacyclopent-3-ene (3ac)

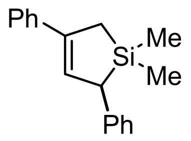

3ac

Following the typical procedure using 1,3-diphenyl-1,3-butadiene **2c** (0.3 mmol, 61.8 mg, 1.0 equiv.), dichlorodimethylsilane **1a** (0.36 mmol, 46.5 mg, 1.2 equiv.), samarium powder (0.6 mmol, 90.0 mg) and freshly prepared SmI_2 (0.09 mmol in 0.9 mL DME). Reaction time: 2 h. Purification by column chromatography (hexane) using silica gel afforded pure **3ac** as a colorless liquid (65.7 mg, 83%). All spectroscopic data matched those reported in the literature.[2]

1,1-dimethyl-2,3-diphenyl-1-silacyclopent-3-ene (3ad)

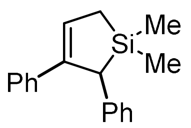

3ad

Following the typical procedure using 1,2-diphenyl-1,3-butadiene **2d** (0.3 mmol, 61.8 mg, 1.0 equiv.), dichlorodimethylsilane **1a** (0.36 mmol, 46.5 mg, 1.2 equiv.), samarium powder (0.6 mmol, 90.0 mg) and freshly prepared SmI_2 (0.09 mmol in 0.9 mL DME). Reaction time: 2 h. Purification by column chromatography (hexane) using silica gel afforded pure **3ad** as a colorless liquid (63.1 mg, 80%).

^{1}H NMR (400 MHz, $CDCl_3$): δ 7.41-7.39 (m, 2H), 7.21-7.17 (m, 4H), 7.14-7.10 (m, 1H), 7.04-6.98 (m, 3H), 6.68 (ddd, J = 4.6, 2.8, 0.9 Hz, 1H), 3.47 (br s, 1H), 1.66 (ddd, J = 18.8 Hz, 2.8 Hz, 1.8 Hz, 1H), 1.53 (dd, J = 18.8, 4.6 Hz, 1H), 0.26 (s, 3H), −0.21 (s, 3H).

^{13}C NMR (100 MHz, $CDCl_3$): δ 143.8, 143.1, 139.4, 129.7, 128.5, 128.2, 126.7, 126.4,

126.3, 124.1, 41.1, 17.7, −2.1, −3.8.
^{29}Si NMR (79 MHz, CDCl$_3$): δ 22.8.

1,1-dimethyl-2-phenyl-1-silacyclopent-3-ene (3ae)

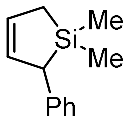

3ae

Following the typical procedure using *(E)*-1-phenyl-1,3-butadiene **2e** (0.5 mmol, 65.0 mg, 1.0 equiv.), dichlorodimethylsilane **1a** (0.6 mmol, 77.4 mg, 1.2 equiv.), samarium powder (1.0 mmol, 150.4 mg) and freshly prepared SmI_2 (0.15 mmol in 1.5 mL DME). Reaction time: 16 h. Purification by column chromatography (hexane) using silica gel afforded pure **3ae** as a colorless liquid (48.9 mg, 52%). All spectroscopic data matched those reported in the literature.[2]

1,1-dimethyl-3-phenyl-1-silacyclopent-3-ene (3af)

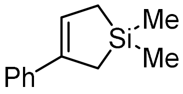

3af

Following the typical procedure using 2-phenyl-1,3-butadiene **2f** (0.5 mmol, 65.0 mg, 1.0 equiv.), dichlorodimethylsilane **1a** (0.6 mmol, 77.4 mg, 1.2 equiv.), samarium powder (1.0 mmol, 150.4 mg) and freshly prepared SmI_2 (0.15 mmol in 1.5 mL DME). Reaction time: 16 h. Purification by column chromatography (hexane) using silica gel afforded pure **3af** as a colorless liquid (84.0 mg, 89%). All spectroscopic data matched those reported in the literature.[2]

1,1-dimethyl-3-(2-naphthyl)-1-silacyclopent-3-ene (3ag)

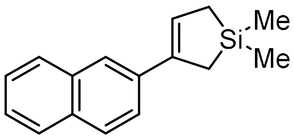

3ag

Following the typical procedure using 2-(buta-1,3-dien-2-yl)naphthalene **2g** (0.5 mmol, 90.0 mg, 1.0 equiv.), dichlorodimethylsilane **1a** (0.6 mmol, 77.4 mg, 1.2 equiv.), samarium powder (1.0 mmol, 150.4 mg) and freshly prepared SmI_2 (0.15 mmol in 1.5 mL DME). Reaction time: 4 h. Purification by column chromatography (hexane) using silica gel afforded pure **3ag** as a colorless liquid (78.6 mg, 66%).
^{1}H NMR (400 MHz, CDCl$_3$): δ 7.88-7.73 (m, 5H), 7.49-7.41 (m, 2H), 6.56 (tt, *J* = 3.6, 1.8 Hz, 1H), 1.84 (dt, *J* = 1.8 Hz, 2H), 1.62 (dt, *J* = 3.6, 1.8 Hz, 2H), 0.30 (s, 6H).
^{13}C NMR (100 MHz, CDCl$_3$): δ 141.5, 138.0, 133.6, 132.4, 128.1, 128.0, 127.5, 127.4,

125.9, 125.4, 124.4, 124.1, 19.4 (2C), −1.7.
^{29}Si NMR (79 MHz, CDCl$_3$): δ 18.4.
Anal. Calcd. for $C_{16}H_{18}Si$: C, 80.61; H, 7.61. Found: C, 80.46; H, 7.83.

1,1-dimethyl-3-(benzo[d][1,3]dioxol-5-yl)-1-silacyclopent-3-ene (3ah)

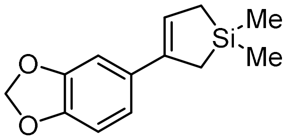

3ah

Following the typical procedure using 5-(buta-1,3-dien-2-yl)benzo[*d*][1,3]dioxole **2h** (0.5 mmol, 87.1 mg, 1.0 equiv.), dichlorodimethylsilane **1a** (0.6 mmol, 77.4 mg, 1.2 equiv.), samarium powder (1.0 mmol, 150.4 mg) and freshly prepared SmI_2 (0.15 mmol in 1.5 mL DME). Reaction time: 8 h. Purification by column chromatography (hexane) using silica gel afforded pure **3ah** as a white solid (66.1 mg, 57%). mp: 32.2-32.8 °C.
^{1}H NMR (400 MHz, CDCl$_3$): δ 7.03 (d, *J* = 1.8 Hz, 1H), 6.97 (dd, *J* = 7.8, 1.8 Hz, 1H), 6.76 (d, *J* = 7.8 Hz, 1H), 6.24 (tt, *J* = 3.6, 1.8 Hz, 1H), 5.94 (s, 2H), 1.64 (dt, *J* = 1.8 Hz, 2H), 1.52 (dt, *J* = 3.6, 1.8 Hz, 2H), 0.24 (s, 6H).
^{13}C NMR (100 MHz, CDCl$_3$): δ 147.5, 146.3, 141.1, 135.4, 126.0, 119.1, 107.8, 106.0, 100.8, 19.8, 19.1, −1.8.
^{29}Si NMR (79 MHz, CDCl$_3$): δ 18.3.
IR (neat, cm^{-1}): 3074, 3012, 2954, 2878, 2777, 1740, 1598, 1503, 1488, 1438, 1348, 1293, 1273, 1247, 1223, 1155, 1120, 1098, 1039, 998, 937, 840, 794, 733, 644, 568.
Anal. Calcd. for $C_{13}H_{16}OSi$: C, 67.20; H, 6.94. Found: C, 67.00; H, 7.07.

1,1-dimethyl-3-ferrocenyl-1-silacyclopent-3-ene (3ai)

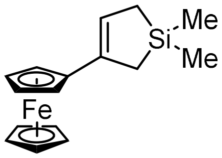

3ai

Following the typical procedure using 2-(buta-1,3-dien-yl)-ferrocene **2i** (0.5 mmol, 119.0 mg, 1.0 equiv.), dichlorodimethylsilane **1a** (0.6 mmol, 77.4 mg, 1.2 equiv.), samarium powder (1.0 mmol, 150.4 mg) and freshly prepared SmI_2 (0.15 mmol in 1.5 mL DME). Reaction time: 16 h. Purification by column chromatography (hexane) using silica gel afforded pure **3ai** as a red solid (108.1 mg, 71%). mp: 45.8-46.8 °C.
^{1}H NMR (400 MHz, CDCl$_3$): δ 5.98 (tt, *J* = 3.6, 1.8 Hz, 1H), 4.37 (t, *J* = 1.8 Hz, 2H), 4.17 (t, *J* = 1.8 Hz, 2H), 4.08 (s, 5H), 1.61 (dt, *J* = 1.8 Hz, 2H), 1.36 (dt, *J* = 3.6, 1.8 Hz, 2H), 0.25 (s, 6H).
^{13}C NMR (100 MHz, CDCl$_3$): δ 139.5, 123.3, 87.0, 68.9, 68.1, 65.9, 19.9, 18.7, −1.7.
^{29}Si NMR (79 MHz, CDCl$_3$): δ 17.7.
IR (neat, cm^{-1}): 3928, 3096, 3011, 2957, 2879, 1617, 1404, 1248, 1147, 1105, 1058, 1027,

1000, 963, 839, 809, 729, 703, 667, 644, 516.

Anal. Calcd. for $C_{16}H_{20}FeSi$: C, 64.87; H, 6.80. Found: C, 65.01; H, 6.87.

1,1-dimethyl-3-(4-fluorophenyl)-1-silacyclopent-3-ene (3aj)

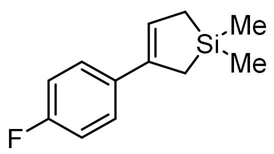

3aj

Following the typical procedure using 2-(4-fluorophenyl)-1,3-butadiene **2j** (0.5 mmol, 74.1 mg, 1.0 equiv.), dichlorodimethylsilane **1a** (0.6 mmol, 77.4 mg, 1.2 equiv.), samarium powder (1.0 mmol, 150.4 mg) and freshly prepared SmI_2 (0.15 mmol in 1.5 mL DME). Reaction time: 18 h. Purification by column chromatography (hexane) using silica gel afforded pure **3aj** as a colorless liquid (67.0 mg, 65%).

^{1}H NMR (400 MHz, $CDCl_3$): δ 7.46 (dd, J = 8.7, 5.5 Hz, 2H), 7.00 (t, J = 8.7 Hz, 2H), 6.31 (s, 1H), 1.67-1.66 (m, 2H), 1.55-1.54 (m, 2H), 0.26 (s, 6H).

^{13}C NMR (100 MHz, $CDCl_3$): δ 161.8 (d, $J_{C\text{-}F}$ = 244 Hz), 140.6, 136.9 (d, $J_{C\text{-}F}$ = 2.9 Hz), 127.0 (d, $J_{C\text{-}F}$ = 7.7 Hz), 126.9, 114.8 (d, $J_{C\text{-}F}$ = 21.0 Hz), 19.6, 19.2, –1.8.

^{29}Si NMR (79 MHz, $CDCl_3$): δ 18.6.

IR (neat, cm^{-1}): 3024, 2955, 2900, 2878, 2782, 1602, 1506, 1404, 1249, 1230, 1149, 1102, 998, 839, 801, 716, 679, 633, 575.

Anal. Calcd. For $C_{12}H_{15}FSi$: C, 69.85; H, 7.33. Found: C, 69.72; H, 7.52.

1,1-dimethyl-3-(4-chlorophenyl)-1-silacyclopent-3-ene (3ak)

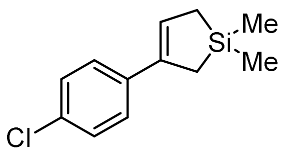

3ak

Following the typical procedure using 2-(4-chlorophenyl)-1,3-butadiene **2k** (0.5 mmol, 82.3 mg, 1.0 equiv.), dichlorodimethylsilane **1a** (0.6 mmol, 77.4 mg, 1.2 equiv.), samarium powder (1.0 mmol, 150.4 mg) and freshly prepared SmI_2 (0.15 mmol in 1.5 mL DME). Reaction time: 18 h. Purification by column chromatography (hexane) using silica gel afforded pure **3ak** as a colorless liquid (34.5 mg, 31%).

^{1}H NMR (400 MHz, $CDCl_3$): δ 7.41 (d, J = 8.7 Hz, 2H), 7.25 (d, J = 8.7 Hz, 2H), 6.35 (tt, J = 3.6, 1.8 Hz, 1H), 1.64 (dt, J = 1.8 Hz, 2H), 1.53 (dt, J = 3.6, 1.8 Hz, 2H), 0.24 (s, 6H).

^{13}C NMR (100 MHz, $CDCl_3$): δ 140.6, 139.2, 132.1, 128.1, 127.9, 126.8, 19.4, 19.3, –1.8.

^{29}Si NMR (79 MHz, $CDCl_3$): δ 18.1.

IR (neat, cm^{-1}): 3027, 2956, 2915, 2883, 1604, 1489, 1399, 1244, 1148, 1090, 1001, 947, 836, 799, 721, 665, 631.

Anal. Calcd. For $C_{12}H_{15}ClSi$: C, 64.69; H, 6.79. Found: C, 64.87; H, 6.85.

1,1-dimethyl-3-(4-methoxyphenyl)-1-silacyclopent-3-ene (3al)

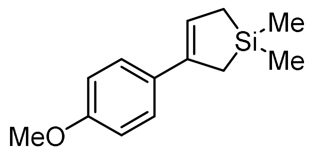

3al

Following the typical procedure using 2-(4-methoxyphenyl)-1,3-butadiene **2l** (0.5 mmol, 80.1 mg, 1.0 equiv.), dichlorodimethylsilane **1a** (0.6 mmol, 77.4 mg, 1.2 equiv.), samarium powder (1.0 mmol, 150.4 mg) and freshly prepared SmI_2 (0.15 mmol in 1.5 mL DME). Reaction time: 13 h. Purification by column chromatography (hexane) using silica gel afforded pure **3al** as a white solid (69.8 mg, 64%). mp: 52.5-53.5 °C.

^{1}H NMR (400 MHz, $CDCl_3$): δ 7.44 (d, *J* = 9.2 Hz, 2H), 6.86 (d, *J* = 9.2 Hz, 2H), 6.27 (tt, *J* = 3.6, 1.8 Hz, 1H), 3.82 (s, 3H), 1.67 (dt, *J* = 1.8 Hz, 2H), 1.53 (dt, *J* = 3.6, 1.8 Hz, 2H), 0.25 (s, 6H).

^{13}C NMR (100 MHz, $CDCl_3$): δ 158.4, 140.9, 133.5, 126.6, 125.2, 113.4, 55.3, 19.5, 19.1, –1.8.

^{29}Si NMR (79 MHz, $CDCl_3$): δ 18.7.

IR (neat, cm^{-1}): 3017, 2965, 2929, 2914, 2872, 1598, 1508, 1458, 1279, 1240, 1179, 1146, 1102, 1024, 996, 947, 826, 779, 735, 627, 584, 476.

Anal. Calcd. For $C_{13}H_{18}OSi$: C, 71.50; H, 8.31. Found: C, 71.27; H, 8.19.

1,1-dimethyl-3-(4-*N,N*-dimethylaminophenyl)-1-silacyclopent-3-ene (3am)

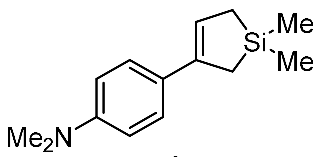

3am

Following the typical procedure using 2-(4-*N,N*-dimethylaminophenyl)-1,3-butadiene **2m** (0.3 mmol, 51.9 mg, 1.0 equiv.), dichlorodimethylsilane **1a** (0.36 mmol, 46.5 mg, 1.2 equiv.), samarium powder (0.6 mmol, 90.2 mg) and freshly prepared SmI_2 (0.09 mmol in 0.9 mL DME). Reaction time: 12 h. Purification by column chromatography (hexane/EtOAc = 6/1 + 0.2 v/v% NEt_3) using silica gel afforded pure **3am** as a pale-yellow liquid (54.1 mg, 78%).

^{1}H NMR (400 MHz, $CDCl_3$): δ 7.40 (d, *J* = 9.2 Hz, 2H), 6.70 (d, *J* = 9.2 Hz, 2H), 6.21 (tt, *J* = 3.6, 1.8 Hz, 1H), 2.94 (s, 6H), 1.65 (dt, *J* = 1.8 Hz, 2H), 1.50 (dt, *J* = 3.6, 1.8 Hz, 2H), 0.22 (s, 6H).

^{13}C NMR (100 MHz, $CDCl_3$): δ 149.5, 141.1, 129.4, 126.3, 123.3, 112.3, 40.7, 19.3, 19.0, –1.7.

^{29}Si NMR (79 MHz, $CDCl_3$): δ 18.1.

IR (neat, cm^{-1}): 2947, 2882, 2802, 1605, 1518, 1480, 1446, 1354, 1240, 1204, 1159, 1101, 1067, 997, 945, 854, 836, 796, 731, 672, 632, 573, 538.

HRMS (APCI): $[M+H]^+$ calcd for $C_{14}H_{22}NSi$: 232.1516, found: 232.1516.

1,4-bis(1,1-dimethyl-2,5-dihydro-1*H*-silol-3-yl)benzene (3an)

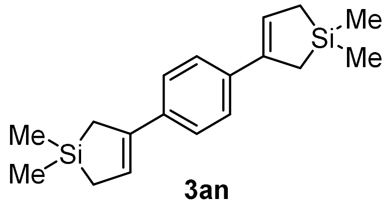

3an

Following the typical procedure using 1,4-di(buta-1,3-dien-2-yl)benzene **2n** (0.25 mmol, 45.5 mg, 1.0 equiv.), dichlorodimethylsilane **1a** (0.6 mmol, 77.4 mg, 2.4 equiv.), samarium powder (1.0 mmol, 150.4 mg, 4.0 equiv.) and freshly prepared SmI_2 (0.15 mmol in 1.5 mL DME, 0.6 equiv.). Reaction time: 13 h. Purification by column chromatography (hexane) using silica gel afforded pure **3an** as a white solid (29.8 mg, 40%). mp: 106.3-106.8 °C.
^{1}H NMR (400 MHz, CDCl$_3$): δ 7.44 (s, 4H), 6.37 (tt, *J* = 3.6, 1.8 Hz, 2H), 1.68 (dt, *J* = 1.8 Hz, 4H), 1.53 (dt, *J* = 3.6, 1.8 Hz, 4H), 0.24 (s, 12H).
^{13}C NMR (100 MHz, CDCl$_3$): δ 141.5, 139.2, 126.8, 125.4, 19.4, 19.3, −1.7.
^{29}Si NMR (79 MHz, CDCl$_3$): δ 18.4.
Anal. Calcd. for $C_{18}H_{26}Si_2$: C, 72.41; H, 8.78. Found: C, 72.19; H, 8.83.

3,3-dimethyl-3,3a,4,5-tetrahydro-2*H*-naphtho[2,1-*b*]silole (3ao)

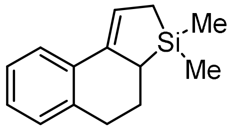

3ao

Following the typical procedure using 4-vinyl-1,2-dihydronaphthalene **2o** (3.0 mmol, 642.0 mg, 1.0 equiv.), dichlorodimethylsilane **1a** (3.6 mmol, 464.4 mg, 1.2 equiv.), samarium powder (6.0 mmol, 902.2 mg) and freshly prepared SmI_2 (0.9 mmol in 9 mL DME). Reaction time: 4 h. Purification by column chromatography (hexane) using silica gel afforded pure **3ao** as a colorless liquid (520.0 mg, 81%).
^{1}H NMR (400 MHz, CDCl$_3$): δ 7.67-7.65 (m, 1H), 7.18-7.12 (m, 3H), 6.45 (dt, *J* = 3.7, 3.2 Hz, 1H), 2.94 (ddd, *J* = 16.4, 12.3, 5.0 Hz, 1H), 2.86 (ddd, *J* = 16.4, 5.0, 2.3 Hz, 1H), 2.09-2.03 (m, 1H), 1.89-1.83 (m, 1H), 1.60 (qt, *J* = 12.3, 5.0 Hz, 1H), 1.52-1.50 (m, 2H), 0.34 (s, 3H), 0.12 (s, 3H).
^{13}C NMR (100 MHz, CDCl$_3$): δ 141.4, 136.7, 134.2, 129.4, 126.6, 125.7, 123.5, 122.0, 31.8, 29.4, 24.1, 17.7, −2.4, −2.5.
^{29}Si NMR (79 MHz, CDCl$_3$): δ 21.1.
HRMS (DART): $[M+H]^+$ calcd for $C_{14}H_{19}Si$: 215.1251, found: 215.1250.

3,3-diisopropyl-3,3a,4,5-tetrahydro-2*H*-naphtho[2,1-*b*]silole (3co)

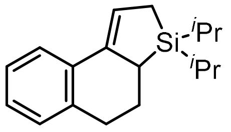

3co

Following the typical procedure of Sm/SmI_2-induced reaction of dienes and dichlorosilanes using **2o** (0.654 mmol, 110.0 mg, 1.0 equiv.), dichlorodiisopropylsilane **1c** (0.786 mmol, 145.6 mg, 1.2 equiv.), samarium powder (1.31 mmol, 196.2 mg, 2.0 equiv.) and freshly prepared SmI_2 (0.196 mmol in 1.96 mL DME, 0.3 equiv.). Reaction time: 3 h. Purification by column chromatography (hexane) using silica gel afforded pure **3co** as a colorless liquid (73.2 mg, 40%).

^{1}H NMR (400 MHz, $CDCl_3$): δ 7.65-7.61 (m, 1H), 7.14-7.08 (m, 3H), 6.44 (td, *J* = 3.7, 3.2 Hz, 1H), 2.89 (ddd, *J* = 16.5, 12.4, 5.0 Hz, 1H), 2.83 (ddd, *J* = 16.5, 5.0, 2.3 Hz, 1H), 2.12-2.07 (m, 1H), 2.03-1.97 (m, 1H), 1.77 (dtd, *J* = 13.8, 12.4, 5.0 Hz, 1H), 1.46-1.44 (m, 2H), 1.25-1.12 (m, 2H), 1.11-1.02 (m, 12H).

^{13}C NMR (100 MHz, $CDCl_3$): δ 141.8, 136.9, 134.2, 129.5, 126.6, 125.8, 123.6, 122.3, 32.3, 26.8, 24.9, 18.7, 18.6, 18.5, 18.2, 11.7, 11.0, 10.4.

^{29}Si NMR (79 MHz, $CDCl_3$): δ 28.7.

HRMS (EI): $[M+H]^+$ calcd for $C_{18}H_{27}Si$: 271.1877, found: 271.1877.

***cis*-1,1-dimethyl-2-phenyl-5-((*E*)-styryl)-1-silacyclopent-3-ene (3ap)**

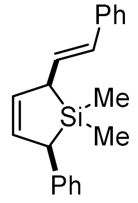

3ap

Following the typical procedure using *(1E,3E,5E)*-1,6-diphenyl-1,3,5-hexatriene **2p** (0.5 mmol, 116.2 mg, 1.0 equiv.), dichlorodimethylsilane **1a** (0.6 mmol, 77.4 mg, 1.2 equiv.), samarium powder (1.0 mmol, 150.4 mg) and freshly prepared SmI_2 (0.15 mmol in 1.5 mL DME). Reaction time: 18 h. Purification by column chromatography (hexane) using silica gel afforded pure **3ap** as a yellow oil (*dr* = 5.3:1, 32.2 mg, 23%).

^{1}H NMR (400 MHz, $CDCl_3$): δ 7.36-7.25 (m, 6H), 7.19-7.16 (m, 1H), 7.12-7.08 (m, 1H), 7.06-7.02 (m, 2H), 6.33-6.29 (m, 2H), 6.11-6.03 (m, 2H), 3.27 (s, 1H, *trans*-isomer), 3.22 (s, 1H), 2.88-2.85 (m, 1H, *trans*-isomer), 2.83-2.81 (m, 1H), 0.38 (s, 3H), 0.21 (s, 3H, *trans*-isomer), –0.17 (s, 3H, *trans*-isomer), –0.32 (s, 3H).

^{13}C NMR (100 MHz, $CDCl_3$): δ 143.3, 143.0 (*trans*-isomer), 138.3, 135.2 (*trans*-isomer), 135.1 (*trans*-isomer), 134.7, 134.6, 131.8, 131.4 (*trans*-isomer), 128.5, 128.3, 126.4, 126.3, 126.3 (*trans*-isomer), 126.2 (*trans*-isomer), 126.0, 125.6, 124.3, 40.0, 39.2 (*trans*-isomer),

36.9, 36.3 (*trans*-isomer), –2.6, –4.3 (*trans*-isomer), –4.6 (*trans*-isomer), –6.5. (Some peaks of *cis*- and *trans*-isomer overlapped).
^{29}Si NMR (79 MHz, CDCl$_3$): δ 23.8 (*cis*-isomer), 21.7 (*trans*-isomer).
HRMS (DART): $[M+H]^+$ calcd for $C_{20}H_{23}Si$: 291.1564, found: 291.1560.

1,1-dimethyl-3-cyclohexyl-1-silacyclopent-3-ene (3aq)

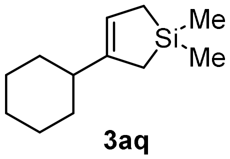

3aq

Following the typical procedure using 2-cyclohexyl-1,3-butadiene **2q** (0.5 mmol, 68.0 mg, 1.0 equiv.), dichlorodimethylsilane **1a** (0.6 mmol, 77.4 mg, 1.2 equiv.), samarium powder (1.0 mmol, 150.4 mg, 2.0 equiv.) and freshly prepared SmI_2 (0.15 mmol in 1.5 mL DME, 0.3 equiv.). Reaction time: 10 h. Purification by column chromatography (hexane + 0.5% triethylamine) using silica gel afforded pure **3aq** as a colorless liquid (43.9 mg, 45%).
^{1}H NMR (400 MHz, CDCl$_3$): δ 5.48 (m, 1H), 1.94 (tt, J = 11.4, 3.2 Hz, 1H), 1.76-1.64 (m, 5H), 1.28-1.12 (m, 9H), 0.14 (s, 6H).
^{13}C NMR (100 MHz, CDCl$_3$): δ 149.5, 121.6, 44.6, 32.0, 26.7, 26.6, 18.3, 18.0, –1.8.
^{29}Si NMR (79 MHz, CDCl$_3$): δ 17.4.
Anal. Calcd. for $C_{12}H_{22}Si$: C, 74.14; H, 11.41. Found: C, 75.99; H, 11.95.
HRMS (APCI): $[M+H]^+$ calcd for $C_{12}H_{23}Si$: 195.1564, found: 195.1564.

1,1-dimethyl-3-(1-adamantyl)-1-silacyclopent-3-ene (3ar)

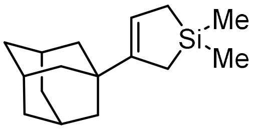

3ar

Following the typical procedure using 2-(1-adamantyl)-1,3-butadiene **2r** (0.5 mmol, 94.0 mg, 1.0 equiv.), dichlorodimethylsilane **1a** (0.6 mmol, 77.4 mg, 1.2 equiv.), samarium powder (1.0 mmol, 150.4 mg) and freshly prepared SmI_2 (0.15 mmol in 1.5 mL DME). Reaction time: 6 h. Purification by column chromatography (hexane) using silica gel afforded pure **3ar** as a colorless liquid (98.4 mg, 80%).
^{1}H NMR (400 MHz, CDCl$_3$): δ 5.51 (tt, J = 3.6, 1.8 Hz, 1H), 1.99-1.97 (m, 3H), 1.73-1.65 (m, 12H), 1.27 (dt, J = 3.6, 1.8 Hz, 2H), 1.23 (dt, J = 1.8 Hz, 2H), 0.13 (s, 6H).
^{13}C NMR (100 MHz, CDCl$_3$): δ 153.1, 120.2, 41.3, 37.2, 36.8, 28.8, 17.8, 15.2, –2.1.
^{29}Si NMR (79 MHz, CDCl$_3$): δ 17.8.
Anal. Calcd. for $C_{16}H_{26}Si$: C, 77.97; H, 10.63. Found: C, 78.28; H, 10.60.

1,1-dimethyl-3-(4-methylpent-3-en-1-yl)-1-silacyclopent-3-ene (3as)

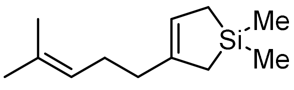

3as

Following the typical procedure using commercially available myrcene **2s** (0.4 mmol, 54.5 mg, 1.0 equiv.), dichlorodimethylsilane **1a** (0.48 mmol, 61.9 mg, 1.2 equiv.), samarium powder (0.8 mmol, 120.3 mg) and freshly prepared SmI_2 (0.12 mmol in 1.2 mL DME). Reaction time: 14 h. Purification by column chromatography (hexane) using silica gel afforded pure **3as** as a colorless liquid (50.0 mg, 64%).
All spectroscopic data matched those reported in the literature.[2]

(*E*)-1,1-dimethyl-3-(2-(2,6,6-trimethylcyclohex-1-en-1-yl)vinyl)-1-silacyclopent-3-ene (3at)

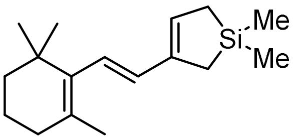

3at

Following the typical procedure using **2t** (0.5 mmol, 101.1 mg, 1.0 equiv.), dichlorodimethylsilane **1a** (0.6 mmol, 77.4 mg, 1.2 equiv.), samarium powder (1.0 mmol, 150.4 mg) and freshly prepared SmI_2 (0.15 mmol in 1.5 mL DME). Reaction time: 13 h. Purification by column chromatography (hexane) using silica gel afforded pure **3at** as a colorless liquid (67.0 mg, 52%).
^{1}H NMR (400 MHz, $CDCl_3$): δ 6.23 (d, J = 16.0 Hz, 1H), 6.05 (d, J = 16.0 Hz, 1H), 5.82-5.81 (m, 1H), 2.00 (t, J = 6.0 Hz, 2H), 1.71 (s, 3H), 1.64-1.57 (m, 2H), 1.47-1.42 (m, 6H), 1.01 (s, 6H), 0.21 (s, 6H).
^{13}C NMR (100 MHz, $CDCl_3$): δ 142.6, 137.8, 134.5, 130.6, 128.5, 126.8, 39.6, 33.0, 28.9, 27.4, 21.7, 19.3, 18.8, 16.4, −1.6.
^{29}Si NMR (79 MHz, $CDCl_3$): δ 18.2.
HRMS (APCI): $[M+H]^+$ calcd for $C_{17}H_{29}Si$: 261.2033, found: 261.2028.

Silacyclopent-3-ene (3au)

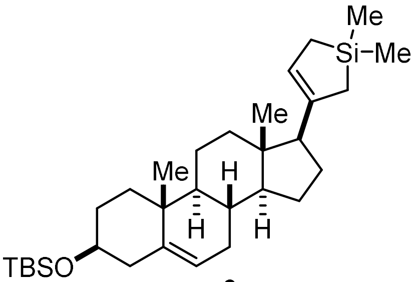

3au

Following the typical procedure using diene **2u** (0.2 mmol, 88.1 mg, 1.0 equiv.),

dichlorodimethylsilane **1a** (0.24 mmol, 31.0 mg, 1.2 equiv.), samarium powder (0.4 mmol, 60.2 mg) and freshly prepared SmI_2 (0.06 mmol in 0.6 mL DME). Reaction time: 24 h. Purification by column chromatography (hexane:EtOAc = 20:1) using silica gel afforded pure **3au** as a white solid (69.7 mg, 70%). mp: 121.5-122.8 °C.

^{1}H NMR (400 MHz, $CDCl_3$): δ 5.57-5.55 (m, 1H), 5.34-5.31 (m, 1H), 3.49 (tt, J = 16.0, 5.0 Hz, 1H), 2.31-2.24 (m, 1H), 2.17 (ddd, J = 13.3, 5.0, 2.3 Hz, 1H), 2.11-2.06 (m, 1H), 2.03-1.95 (m, 1H), 1.91-1.79 (m, 3H), 1.74-1.62 (m, 3H), 1.58-1.34 (m, 5H), 1.29-1.28 (m, 2H), 1.22-1.14 (m, 3H), 1.08-1.04 (m, 2H), 1.00 (s, 3H+1H), 0.98-0.92 (m, 1H), 0.89 (s, 9H), 0.54 (s, 3H), 0.16 (s, 3H), 0.13 (s, 3H), 0.06 (s, 6H).

^{13}C NMR (100 MHz, $CDCl_3$): δ 143.8, 141.6, 124.7, 121.1, 72.6, 56.6, 56.3, 50.4, 43.6, 42.8, 38.8, 37.4, 36.6, 32.1, 32.0, 31.9, 25.9, 25.4, 24.4, 22.2, 21.0, 19.4, 18.3, 17.8, 12.9, −1.8, −2.0, −4.6.

^{29}Si NMR (79 MHz, $CDCl_3$): δ 17.4, 16.9.

IR (neat, cm^{-1}): 3030, 2958, 2929, 2904, 2883, 2855, 2824, 1471, 1462, 1429, 1382, 1367, 1249, 1150, 1135, 1079, 1028, 1006, 957, 940, 886, 873, 837, 803, 777, 731, 702, 673, 636.

Anal. Calcd. for $C_{31}H_{54}OSi_2$: C, 74.63; H, 10.91. Found: C, 74.55; H, 10.99.

4. SmI_2/Sm-induced [4+2]-silacyclization using dienes and 1,2-dichlorotetramethyldisilane

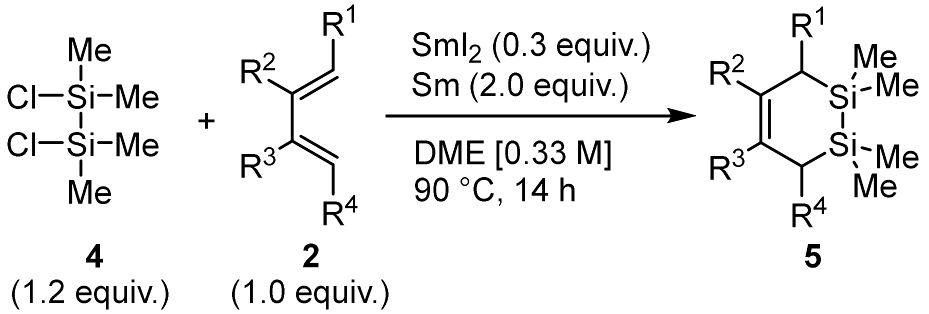

Following the same procedure as reactions using dichlorosilanes.

***cis*-1,1,2,2-tetramethyl-3,6-diphenyl-1,2,3,6-tetrahydro-1,2-disiline (5a)**

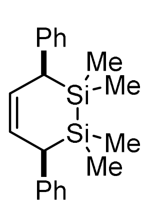

5a

Following the typical procedure using diene **2a** (0.5 mmol, 103.0 mg, 1.0 equiv.), 1,2-dichlorotetramethyldisilane **4** (0.6 mmol, 112.3 mg, 1.2 equiv.), samarium powder (1.0 mmol, 150.4 mg) and freshly prepared SmI_2 (0.15 mmol in 1.5 mL DME). Reaction time:

14 h. Purification by column chromatography (hexane) using silica gel afforded pure **5a** as a white solid (157.8 mg, 98%). mp: 178.5-179.5 °C.

^{1}H NMR (400 MHz, CDCl$_3$): δ 7.33-7.29 (m, 4H), 7.16-7.10 (m, 6H), 5.94 (d, J = 3.2 Hz, 2H), 3.05 (d, J = 3.2 Hz, 2H), 0.05 (s, 6H), –0.07 (s, 6H).

^{13}C NMR (100 MHz, CDCl$_3$): δ 142.3, 128.9, 128.3, 127.0, 124.4, 38.4, –4.9, –6.1.

^{29}Si NMR (79 MHz, CDCl$_3$): δ –17.3.

Anal. Calcd. for $C_{20}H_{26}Si_2$: C, 74.46; H, 8.12. Found: C, 74.53; H, 8.20.

1,1,2,2-tetramethyl-4,5-diphenyl-1,2,3,6-tetrahydro-1,2-disiline (5b)

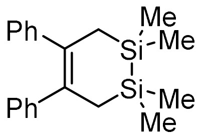

5b

Following the typical procedure using diene **2b** (1.0 mmol, 206.0 mg, 1.0 equiv.), 1,2-dichlorotetramethyldisilane **4** (1.2 mmol, 224.7 mg, 1.2 equiv.), samarium powder (2.0 mmol, 300.8 mg) and freshly prepared SmI_2 (0.3 mmol in 3.0 mL DME). Reaction time: 14 h. Purification by column chromatography (hexane) using silica gel afforded pure **5b** as a white solid (247.9 mg, 77%). mp: 76.5-77.5 °C.

^{1}H NMR (400 MHz, CDCl$_3$): δ 7.07-7.03 (m, 4H), 7.01-6.99 (m, 2H), 6.96-6.93 (m, 4H), 2.00 (s, 4H), 0.18 (s, 12H).

^{13}C NMR (100 MHz, CDCl$_3$): δ 145.9, 133.8, 129.6, 127.4, 125.1, 26.6, –3.8.

^{29}Si NMR (79 MHz, CDCl$_3$): δ –23.1.

Anal. Calcd. for $C_{20}H_{26}Si_2$: C, 74.46; H, 8.12. Found: C, 74.40; H, 8.04.

3,3,4,4-tetramethyl-2,3,4,4a,5,6-hexahydronaphtho[2,1-*c*][1,2]disiline (5o)

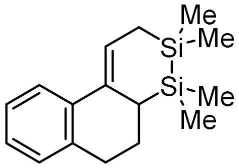

5o

Following the typical procedure using diene **2o** (0.5 mmol, 78.2 mg, 1.0 equiv.), 1,2-dichlorotetramethyldisilane **4** (0.6 mmol, 112.3 mg, 1.2 equiv.), samarium powder (1.0 mmol, 150.4 mg) and freshly prepared SmI_2 (0.15 mmol in 1.5 mL DME). Reaction time: 14 h. Purification by column chromatography (hexane) using silica gel afforded pure **5o** as a colorless oil (85.5 mg, 63%).

^{1}H NMR (400 MHz, CDCl$_3$): δ 7.39 (d, J = 7.8 Hz, 1H), 7.13 (td, J = 7.8, 1.8 Hz, 1H), 7.10-7.03 (m, 2H), 6.13 (ddd, J = 8.7, 6.0, 2.8 Hz, 1H), 2.74 (ddd, J = 14.7, 8.2, 4.1 Hz, 1H), 2.55 (ddd, J = 14.7, 11.4, 3.6 Hz, 1H), 2.13-2.06 (m, 1H), 2.00-1.95 (m, 1H), 1.80 (dd, J = 13.8, 8.7 Hz, 1H), 1.64-1.53 (m, 1H), 1.38 (ddd, J = 13.8, 6.0, 1.8 Hz, 1H), 0.18 (s, 3H), 0.16 (s, 3H), 0.09 (s, 3H), –0.18 (s, 3H).

^{13}C NMR (100 MHz, CDCl$_3$): δ 138.4, 138.0, 136.1, 127.4, 126.2, 125.6, 123.8, 120.6, 31.6, 27.4, 23.0, 18.0, –3.5, –3.8, –5.4, –6.5.
^{29}Si NMR (79 MHz, CDCl$_3$): δ –19.9, –20.8.
Anal. Calcd. for $C_{16}H_{24}Si_2$: C, 70.51; H, 8.88. Found: C, 70.38; H, 8.92.

4-((3*r*,5*r*,7*r*)-adamantan-1-yl)-1,1,2,2-tetramethyl-1,2,3,6-tetrahydro-1,2-disiline (5r)

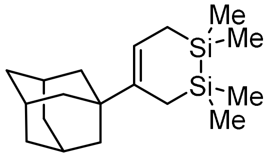

5r

Following the typical procedure using diene **2r** (0.5 mmol, 94.0 mg, 1.0 equiv.), 1,2-dichlorotetramethyldisilane **4** (0.6 mmol, 112.3 mg, 1.2 equiv.), samarium powder (1.0 mmol, 150.4 mg) and freshly prepared SmI_2 (0.15 mmol in 1.5 mL DME). Reaction time: 14 h. Purification by column chromatography (hexane) using silica gel afforded pure **5r** as a colorless oil (118.6 mg, 78%).
^{1}H NMR (400 MHz, CDCl$_3$): δ 5.31 (t, J = 7.3 Hz, 1H), 1.98 (s, 3H), 1.71-1.58 (m, 12H), 1.43 (s, 2H), 1.33 (d, J = 7.3 Hz, 2H), 0.06 (s, 6H), 0.04 (s, 6H).
^{13}C NMR (100 MHz, CDCl$_3$): δ 146.1, 114.9, 41.4, 37.6, 37.1, 28.8, 17.2, 16.3, –3.4, –4.1.
^{29}Si NMR (79 MHz, CDCl$_3$): δ –22.0, –22.1.
HRMS (DART): $[M+H]^+$ calcd for $C_{18}H_{33}Si_2$: 305.2115, found: 305.2112.

5. SmI$_2$/Sm-induced [5+1]-silacyclization using vinylcyclopropanes and dichlorosilanes

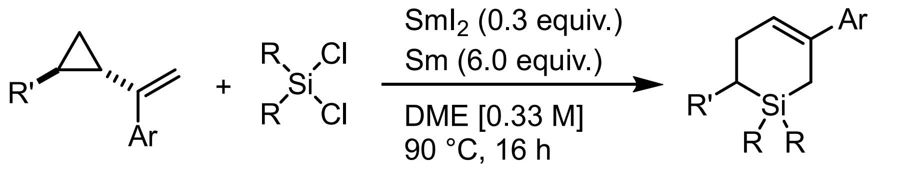

6 (1.0 equiv.) **1** (4.0 equiv.) **7**

Typical Procedure
To a flame-dried Schlenk tube were added samarium powder (6.3 equiv), 1,2-diiodoethane (0.3 equiv) and anhydrous 1,2-dimethoxyethane [0.33 M SmI_2] under nitrogen atmosphere. The reaction mixture was stirred vigorously at room temperature for 40 min until a deep blue suspension was formed. Next, vinylcyclopropane **6** (1.0 equiv) and dichlorosilane **1** (4.0 equiv) were added dropwise to the mixture under nitrogen atmosphere sequentially. The mixture was stirred at 90 °C and checked by TLC or GC-MS until **6** was all consumed. After working up by the same procedure as before, the product was purified by column chromatography using silica gel to afford pure **7**.

1,1-dimethyl-5-phenyl-1,2,3,6-tetrahydrosiline (7a)

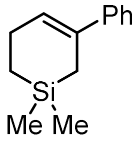

7a

Following the typical procedure using vinylcyclopropane **6a** (0.5 mmol, 74.0 mg, 1.0 equiv.), dichlorosilane **1a** (2.0 mmol, 258.0 mg, 4.0 equiv), samarium powder (3.0 mmol, 451.8 mg, 6.0 equiv) and freshly prepared SmI_2 (0.15 mmol in 1.5 mL DME). Reaction time: 16 h. Purification by column chromatography (hexane) using silica gel afforded pure **7a** as a colorless liquid (41.3 mg, 41%).

^{1}H NMR (400 MHz, CDCl$_3$): δ 7.40-7.37 (m, 2H), 7.31-7.29 (m, 2H), 7.22-7.18 (m, 1H), 6.07 (tt, J = 6.4, 1.4 Hz, 1H), 2.37 (tdt, J = 6.8, 6.4, 1.4 Hz, 2H), 1.64 (q, J = 1.4 Hz, 2H), 0.68 (t, J = 6.8 Hz, 2H), 0.09 (s, 6H).
^{13}C NMR (100 MHz, CDCl$_3$): δ 145.6, 137.4, 128.1, 127.8, 126.3, 125.6, 23.3, 16.6, 9.5, –2.3.
^{29}Si NMR (79 MHz, CDCl$_3$): δ –1.9.
Anal. Calcd. for $C_{13}H_{18}Si$: C, 77.16; H, 8.97. **Found:** C, 77.02; H, 8.84.
HRMS (APCI): $[M+H]^+$ calcd for $C_{13}H_{19}Si$: 203.1251, found: 203.1250.

1,1-dimethyl-2,5-diphenyl-1,2,3,6-tetrahydrosiline (7b)

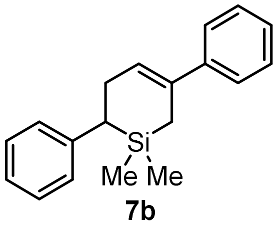

7b

Following the typical procedure using vinylcyclopropane **6b** (0.3 mmol, 66.0 mg, 1.0 equiv.), dichlorosilane **1a** (1.2 mmol, 154.8 mg, 4.0 equiv), samarium powder (1.8 mmol, 270.6 mg, 6.0 equiv) and freshly prepared SmI_2 (0.09 mmol in 0.9 mL DME). Reaction time: 16 h. Purification by column chromatography (hexane) using silica gel afforded pure **7b** as a colorless oil (61.5 mg, 74%).
^{1}H NMR (400 MHz, CDCl$_3$): δ 7.44-7.40 (m, 2H), 7.34-7.30 (m, 2H), 7.25-7.21 (m, 3H), 7.12-7.09 (m, 3H), 6.20 (t, J = 5.0 Hz, 1H), 2.77-2.74 (m, 2H), 2.35 (t, J = 7.8 Hz, 1H), 1.81-1.76 (m, 1H), 1.74-1.70 (m, 1H), 0.11 (s, 3H), –0.10 (s, 3H).
^{13}C NMR (100 MHz, CDCl$_3$): δ 145.4, 144.8, 136.9, 128.3, 128.2, 127.0, 126.8, 126.6, 125.7, 124.4, 31.4, 30.4, 16.4, –2.8, –5.0.
^{29}Si NMR (79 MHz, CDCl$_3$): δ –0.5.
Anal. Calcd. for $C_{19}H_{22}Si$: C, 81.95; H, 7.96. **Found:** C, 81.82; H, 8.08.

1,1-diethyl-2,5-diphenyl-1,2,3,6-tetrahydrosiline (7c)

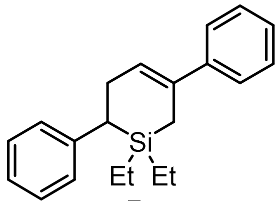

7c

Following the typical procedure using vinylcyclopropane **6b** (0.3 mmol, 66.0 mg, 1.0 equiv.), dichlorosilane **1b** (1.2 mmol, 188.5 mg, 4.0 equiv), samarium powder (1.8 mmol, 270.6 mg, 6.0 equiv) and freshly prepared SmI_2 (0.09 mmol in 0.9 mL DME). Reaction time: 16 h. Purification by column chromatography (hexane) using silica gel afforded pure **7c** as a colorless oil (73.6 mg, 80%).

^{1}H NMR (400 MHz, $CDCl_3$): δ 7.46-7.44 (m, 2H), 7.35-7.31 (m, 2H), 7.27-7.22 (m, 3H), 7.14-7.08 (m, 3H), 6.19 (t, *J* = 5.5 Hz, 1H), 2.76-2.72 (m, 2H), 2.44 (t, *J* = 7.8 Hz, 1H), 1.81-1.77 (m, 1H), 1.75-1.70 (m, 1H), 0.97 (t, *J* = 7.8 Hz, 3H), 0.75 (t, *J* = 7.8 Hz, 3H), 0.64 (q, *J* = 7.8 Hz, 2H), 0.50-0.35 (m, 2H).

^{13}C NMR (100 MHz, $CDCl_3$): δ 145.3, 137.5, 128.3, 128.2, 127.1, 127.0, 126.6, 125.8, 124.4, 30.8, 29.5, 12.5, 7.5, 7.2, 4.0, 2.6.

^{29}Si NMR (79 MHz, $CDCl_3$): δ 3.0.

HRMS (APCI): $[M+H]^+$ calcd for $C_{21}H_{27}Si$: 307.1877, found: 307.1877.

1,1,2,5-tetraphenyl-1,2,3,6-tetrahydrosiline (7d)

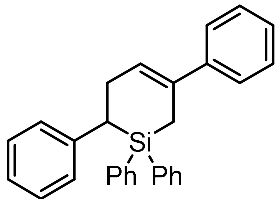

7d

Following the typical procedure using vinylcyclopropane **6b** (0.3 mmol, 66.0 mg, 1.0 equiv.), dichlorosilane **1e** (1.2 mmol, 303.8 mg, 4.0 equiv), samarium powder (1.8 mmol, 270.6 mg, 6.0 equiv) and freshly prepared SmI_2 (0.09 mmol in 0.9 mL DME). Reaction time: 16 h. Purification by column chromatography (hexane:EtOAc = 20:1) using silica gel afforded pure **7d** as a white solid (58.2 mg, 48%).

^{1}H NMR (400 MHz, $CDCl_3$): δ 7.57-7.53 (m, 3H), 7.44-7.34 (m, 6H), 7.31-7.25 (m, 5H), 7.17-7.13 (m, 2H), 7.11-7.07 (m, 1H), 6.98-6.96 (m, 2H), 6.32-6.30 (m, 1H), 2.99 (dd, *J* = 9.6, 5.5 Hz, 1H), 2.93-2.78 (m, 2H), 2.41-2.36 (m, 1H), 2.33-2.28 (m, 1H).

^{13}C NMR (100 MHz, $CDCl_3$): δ 145.1, 143.7, 136.4, 135.8, 135.0, 133.4, 129.7, 129.6, 128.3, 128.1, 128.0, 127.9, 127.8, 127.6, 126.8, 125.8, 124.9, 32.1, 30.2, 14.5.

^{29}Si NMR (79 MHz, $CDCl_3$): δ −13.5.

HRMS (APCI): $[M+H]^+$ calcd for $C_{29}H_{27}Si$: 403.1877, found: 403.1873.

1,1-dimethyl-5-phenyl-2-(*p*-tolyl)-1,2,3,6-tetrahydrosiline (7e)

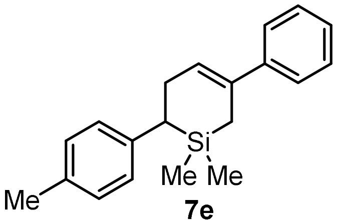

7e

Following the typical procedure using vinylcyclopropane **6c** (0.25 mmol, 58.5 mg, 1.0 equiv.), dichlorosilane **1a** (1.0 mmol, 129.0 mg, 4.0 equiv), samarium powder (1.5 mmol, 225.5 mg, 6.0 equiv) and freshly prepared SmI_2 (0.075 mmol in 0.75 mL DME). Reaction time: 16 h. Purification by column chromatography (hexane) using silica gel afforded pure **7e** as a colorless oil (48.4 mg, 66%).

^{1}H NMR (400 MHz, $CDCl_3$): δ 7.46-7.43 (m, 2H), 7.35-7.31 (m, 2H), 7.25-7.22 (m, 1H), 7.08 (d, J = 8.2 Hz, 2H), 7.00 (d, J = 8.2 Hz, 2H), 6.20 (t, J = 5.0 Hz, 1H), 2.76-2.72 (m, 2H), 2.32 (s, 3H), 2.31 (t, J = 7.3 Hz, 1H), 1.81-1.76 (m, 1H), 1.75-1.70 (m, 1H), 0.12 (s, 3H), –0.08 (s, 3H).

^{13}C NMR (100 MHz, $CDCl_3$): δ 145.4, 141.6, 136.9, 133.7, 129.0, 128.2, 127.2, 126.8, 126.6, 125.7, 30.8, 30.7, 21.0, 16.4, –2.8, –5.0.

^{29}Si NMR (79 MHz, $CDCl_3$): δ –0.8.

HRMS (APCI): $[M+H]^+$ calcd for $C_{20}H_{25}Si$: 293.1720, found: 293.1723.

1,1-dimethyl-5-phenyl-2-(*o*-tolyl)-1,2,3,6-tetrahydrosiline (7f)

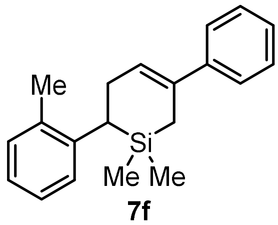

7f

Following the typical procedure using vinylcyclopropane **6d** (0.25 mmol, 58.5 mg, 1.0 equiv.), dichlorosilane **1a** (1.0 mmol, 129.0 mg, 4.0 equiv), samarium powder (1.5 mmol, 225.5 mg, 6.0 equiv) and freshly prepared SmI_2 (0.075 mmol in 0.75 mL DME). Reaction time: 16 h. Purification by column chromatography (hexane) using silica gel afforded pure **7f** as a colorless oil (41.4 mg, 57%).

^{1}H NMR (400 MHz, $CDCl_3$): δ 7.46-7.41 (m, 2H), 7.35-7.31 (m, 2H), 7.25-7.22 (m, 1H), 7.16-7.13 (m, 3H), 7.03-6.99 (m, 1H), 6.23 (t, J = 5.0 Hz, 1H), 2.74-2.71 (m, 2H), 2.57 (dd, J = 8.7, 6.4 Hz, 1H), 2.28 (s, 3H), 1.83-1.74 (m, 2H), 0.13 (s, 3H), –0.10 (s, 3H).

^{13}C NMR (100 MHz, $CDCl_3$): δ 145.2, 143.6, 137.2, 134.8, 130.1, 128.3, 127.2, 126.6, 126.2, 126.0, 125.7, 124.1, 31.4, 26.7, 20.7, 16.8, –1.9, –4.5.

^{29}Si NMR (79 MHz, $CDCl_3$): δ 0.2.

HRMS (APCI): $[M+H]^+$ calcd for $C_{20}H_{25}Si$: 293.1720, found: 293.1723.

2-(4-fluorophenyl)-1,1-dimethyl-5-phenyl-1,2,3,6-tetrahydrosiline (7g)

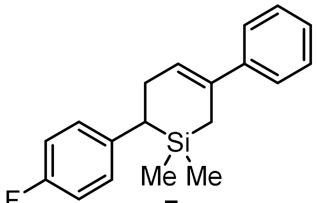

7g

Following the typical procedure using vinylcyclopropane **6e** (0.25 mmol, 59.5 mg, 1.0 equiv.), dichlorosilane **1a** (1.0 mmol, 129.0 mg, 4.0 equiv), samarium powder (1.5 mmol, 225.5 mg, 6.0 equiv) and freshly prepared SmI_2 (0.075 mmol in 0.75 mL DME). Reaction time: 16 h. Purification by column chromatography (hexane) using silica gel afforded pure **7g** as a pale-yellow oil (56.3 mg, 76%).

^{1}H NMR (400 MHz, $CDCl_3$): δ 7.45-7.40 (m, 2H), 7.35-7.31 (m, 2H), 7.25-7.22 (m, 1H), 7.06-7.03 (m, 2H), 6.98-6.94 (m, 2H), 6.19 (dd, *J* = 6.0, 4.6 Hz, 1H), 2.79-2.65 (m, 2H), 2.32 (dd, *J* = 9.1, 6.0 Hz, 1H), 1.81-1.70 (m, 2H), 0.11 (s, 3H), –0.10 (s, 3H).

^{13}C NMR (100 MHz, $CDCl_3$): δ 160 (d, $J_{C\text{-}F}$ = 241 Hz), 145.3, 140.3 (d, $J_{C\text{-}F}$ = 2.9 Hz), 137.0, 128.3, 127.9 (d, $J_{C\text{-}F}$ = 6.7 Hz), 126.8, 126.7, 125.7, 115.0 (d, $J_{C\text{-}F}$ = 20 Hz), 30.7, 30.5, 16.2, –2.8, –5.0.

^{29}Si NMR (79 MHz, $CDCl_3$): δ –0.6.

HRMS (APCI): $[M+H]^+$ calcd for $C_{19}H_{22}FSi$: 297.1467, found: 297.1472.

2-(4-dimethylaminophenyl)-1,1-dimethyl-5-phenyl-1,2,3,6-tetrahydrosiline (7h)

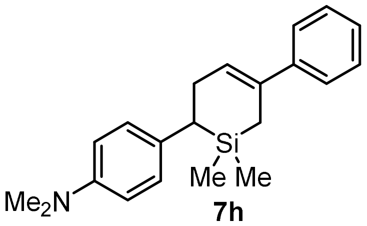

7h

Following the typical procedure using vinylcyclopropane **6f** (0.25 mmol, 65.8 mg, 1.0 equiv.), dichlorosilane **1a** (1.0 mmol, 129.0 mg, 4.0 equiv), samarium powder (1.5 mmol, 225.5 mg, 6.0 equiv) and freshly prepared SmI_2 (0.075 mmol in 0.75 mL DME). Reaction time: 16 h. Purification by column chromatography (hexane) using silica gel afforded pure **7h** as a yellow oil (31.6 mg, 40%).

^{1}H NMR (400 MHz, $CDCl_3$): δ 7.45-7.40 (m, 2H), 7.34-7.30 (m, 2H), 7.24-7.20 (m, 1H), 6.99 (d, *J* = 8.7 Hz, 2H), 6.71 (d, *J* = 8.7 Hz, 2H), 6.18 (dd, *J* = 5.5, 4.1 Hz, 1H), 2.91 (s, 6H), 2.74-2.68 (m, 2H), 2.23 (dd, *J* = 9.6, 6.4 Hz, 1H), 1.78-1.68 (m, 2H), 0.10 (s, 3H), –0.10 (s, 3H).

^{13}C NMR (100 MHz, $CDCl_3$): δ 145.6, 136.8, 128.2, 127.5, 127.4, 126.5, 125.7, 125.7, 114.5, 113.4, 41.1, 31.0, 29.8, 16.5, –2.8, –4.9.

^{29}Si NMR (79 MHz, $CDCl_3$): δ –1.1.

HRMS (APCI): $[M+H]^+$ calcd for $C_{21}H_{28}NSi$: 322.1986, found: 322.1987.

2-(3,4-dimethoxyphenyl)-1,1-dimethyl-5-phenyl-1,2,3,6-tetrahydrosiline (7i)

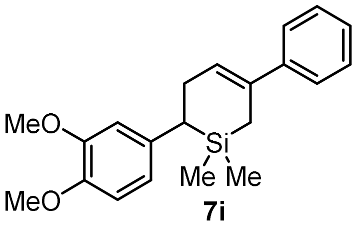

7i

Following the typical procedure using vinylcyclopropane **6g** (0.25 mmol, 70.0 mg, 1.0 equiv.), dichlorosilane **1a** (1.0 mmol, 129.0 mg, 4.0 equiv), samarium powder (1.5 mmol, 225.5 mg, 6.0 equiv) and freshly prepared SmI_2 (0.075 mmol in 0.75 mL DME). Reaction time: 16 h. Purification by column chromatography (hexane) using silica gel afforded pure **7h** as a white solid (47.6 mg, 57%).

^{1}H NMR (400 MHz, $CDCl_3$): δ 7.44-7.41 (m, 2H), 7.34-7.30 (m, 2H), 7.25-7.21 (m, 1H), 6.79 (d, *J* = 8.7 Hz, 1H), 6.65 (d, *J* = 1.8 Hz, 1H), 6.63 (dd, *J* = 8.7, 1.8 Hz, 1H), 6.18 (dd, *J* = 5.5, 4.1 Hz, 1H), 3.86 (s, 3H), 3.85 (s, 3H), 2.79-2.70 (m, 2H), 2.28 (dd, *J* = 9.6, 6.0 Hz, 1H), 1.77-1.69 (m, 2H), 0.11 (s, 3H), –0.08 (s, 3H).

^{13}C NMR (100 MHz, $CDCl_3$): δ 148.8, 146.2, 145.5, 137.5, 137.0, 128.2, 127.0, 126.6, 125.7, 118.4, 111.4, 110.5, 56.0, 55.9, 30.9, 30.6, 16.4, –2.7, –2.8.

^{29}Si NMR (79 MHz, $CDCl_3$): δ –0.9.

HRMS (APCI): $[M+H]^+$ calcd for $C_{21}H_{27}O_2Si$: 339.1775, found: 339.1771.

1,2-disilacyclohept-4-ene (8a)

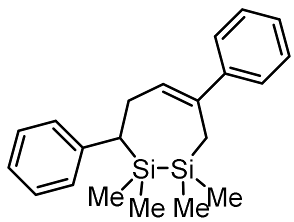

8a

Following the typical procedure using vinylcyclopropane **6d** (0.3 mmol, 66.0 mg, 1.0 equiv.), dichlorodisilane **4** (1.2 mmol, 224.7 mg, 4.0 equiv), samarium powder (1.8 mmol, 270.6 mg, 6.0 equiv) and freshly prepared SmI_2 (0.09 mmol in 0.9 mL DME). Reaction time: 16 h. Purification by column chromatography (hexane) using silica gel afforded pure **8a** as a white solid (25.3 mg, 25%). In some cases, $(SiMe_2)_4O$ remained as an impurity after column chromatography, but it could be removed by Kugelrohr distillation under vacuum at temperatures above 100 °C.

^{1}H NMR (400 MHz, $CDCl_3$): δ 7.42-7.40 (m, 2H), 7.33-7.29 (m, 2H), 7.27-7.20 (m, 3H), 7.12-7.08 (m, 1H), 7.02-7.00 (m, 2H), 5.91 (t, *J* = 9.2 Hz, 1H), 2.89 (ddd, *J* = 13.3-13.7, 11.2-11.4, 9.2 Hz, 1H), 2.53-2.47 (m, 2H), 2.29 (dd, *J* = 11.2-11.4m 1.4 Hz, 1H), 1,95 (d, *J* = 13.7 Hz, 1H), 0.15 (s, 3H), 0.01 (s, 6H), –0.16 (s, 3H).

^{13}C NMR (100 MHz, $CDCl_3$): δ 145.4, 144.7, 139.6, 128.2, 128.0, 127.2, 126.7, 126.4, 125.2, 124.3, 35.0, 31.5, 19.5, –2.1, –3.3, –3.8, –6.3.

^{29}Si NMR (79 MHz, CDCl$_3$): δ −13.1, −19.2.
H/^{29}Si HMQC NMR (400/79MHz, CDCl$_3$, TMS-free, 298K, optimized for *J* = 7 Hz): δ −19.17/−0.16, −19.17/0.01, −19.17/0.15, −19.17/1.95, −19.17/2.29, −19.17/2.89, −13.11/−0.16, −13.11/0.01, −13.11/0.15, −13.11/1.95, −13.11/2.50.
HRMS (APCI): $[M+H]^+$ calcd for $C_{21}H_{29}Si_2$: 337.1802, found: 337.1800.

1,2-disilacyclohept-4-ene (8b)

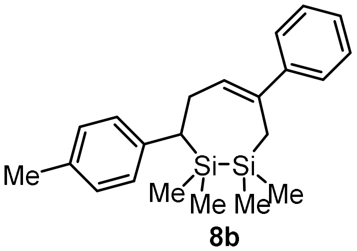

8b

Following the typical procedure using vinylcyclopropane **6e** (0.25 mmol, 58.5 mg, 1.0 equiv.), dichlorodisilane **4** (1.0 mmol, 187.2 mg, 4.0 equiv), samarium powder (1.5 mmol, 225.5 mg, 6.0 equiv) and freshly prepared SmI_2 (0.075 mmol in 0.75 mL DME). Reaction time: 16 h. Purification by column chromatography (hexane) using silica gel afforded pure **8b** as a colorless oil (19.7 mg, 24%). In some cases, $(SiMe_2)_4O$ remained as an impurity after column chromatography, but it could be removed by Kugelrohr distillation under vacuum at temperatures above 100 °C.
^{1}H NMR (400 MHz, CDCl$_3$): δ 7.42-7.39 (m, 2H), 7.33-7.29 (m, 2H), 7.24-7.20 (m, 1H), 7.08 (d, *J* = 7.8 Hz, 2H), 6.90 (d, *J* = 7.8 Hz, 2H), 5.91 (ddd, *J* = 9.2, 7.3, 1.4 Hz, 1H), 2.87 (ddd, *J* = 12.8, 11.0, 9.1 Hz, 1H), 2.50-2.46 (m, 2H), 2.31 (s, 3H), 2.45 (dd, *J* = 11.0, 1.4 Hz, 1H), 1.95 (d, *J* = 13.7 Hz, 1H), 0.15 (3H), 0.00 (6H), −0.15 (3H).
^{13}C NMR (100 MHz, CDCl$_3$): δ 144.7, 142.2, 139.5, 133.6, 128.7, 128.2, 127.1, 126.6, 126.4, 125.2, 34.5, 31.7, 21.0, 19.5, −2.1, −3.3, −3.8, −6.3.
^{29}Si NMR (79 MHz, CDCl$_3$): δ −13.4, −19.1.
HRMS (APCI): $[M+H]^+$ calcd for $C_{22}H_{31}Si_2$: 351.1959, found: 351.1956.

1,2-disilacyclohept-4-ene (8c)

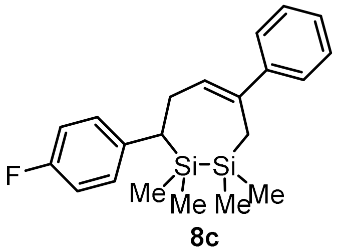

8c

Following the typical procedure using vinylcyclopropane **6g** (0.25 mmol, 60.0 mg, 1.0 equiv.), dichlorodisilane **4** (1.0 mmol, 187.2 mg, 4.0 equiv), samarium powder (1.5 mmol, 225.5 mg, 6.0 equiv) and freshly prepared SmI_2 (0.075 mmol in 0.75 mL DME). Reaction time: 16 h. Purification by column chromatography (hexane) using silica gel afforded pure

8c as a colorless oil (21.2 mg, 24%). In some cases, $(SiMe_2)_4O$ remained as an impurity after column chromatography, but it could be removed by Kugelrohr distillation under vacuum at temperatures above 100 °C.

^{1}H NMR (400 MHz, $CDCl_3$): δ 7.42-7.40 (m, 2H), 7.33-7.29 (m, 2H), 7.24-7.20 (m, 1H), 6.99-6.92 (m, 4H), 5.90 (t, J = 9.2 Hz, 1H), 2.86 (ddd, J = 12.4, 11.4, 9.2 Hz, 1H), 2.46 (m, 2H), 2.27 (dd, J = 11.4, 1.4 Hz, 1H), 1.96 (d, J = 13.7 Hz, 1H), 0.15 (3H), 0.01 (3H), 0.00 (3H), −0.17 (3H).

^{13}C NMR (100 MHz, $CDCl_3$): δ 160.0 (d, $J_{C\text{-}F}$ = 241 Hz), 144.6, 141.0 (d, $J_{C\text{-}F}$ = 2.9 Hz), 139.7, 128.2, 128.1 (d, $J_{C\text{-}F}$ = 7.7 Hz), 126.7, 126.4, 124.9, 114.7 (d, $J_{C\text{-}F}$ = 21.0 Hz), 34.2, 31.7, 19.5, −2.1, −3.3, −3.8, −6.3.

^{29}Si NMR (79 MHz, $CDCl_3$): δ −12.6, −18.5.

HRMS (APCI): $[M+H]^+$ calcd for $C_{21}H_{28}FSi_2$: 355.1708, found: 355.1706.

6. SmI_2/Sm-induced [3+1]-silacyclization of benzylidenecyclopropane 9 and dichlorosilane 1a

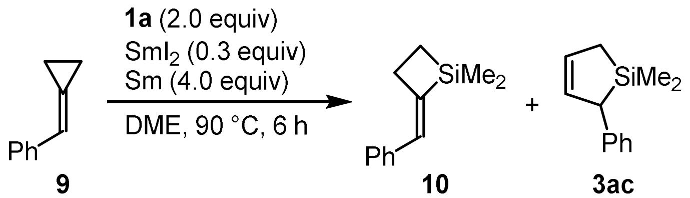

9 10 3ac

Following the general procedure using **9** (0.5 mmol, 65.0 mg, 1.0 equiv.), dichlorodisilane **1** (1.0 mmol, 129.0 mg), samarium powder (2.0 mmol, 300.7 mg, 4.0 equiv) and freshly prepared SmI_2 (0.15 mmol in 1.5 mL DME). Reaction time: 5 h. Purification by column chromatography (hexane) using silica gel afforded **10** as a colorless liquid (38.0 mg, 41% *dr* 2.1:1). A side product, identified as **3ac**, could not be separated by silica gel chromatography. The mixture of isomers was obtained without assignment of their configurations.

^{1}H NMR (400 MHz, $CDCl_3$): (major isomer) δ 7.33-7.28 (m, 2H), 7.24-7.15 (m, 3H), 6.75 (t, J = 2.3 Hz, 1H), 2.93-2.88 (m, 2H), 1.13-1.09 (m, 2H), 0.43 (s, 6H). (minor isomer) δ 7.33-7.28 (m, 2H), 7.24-7.15 (m, 3H), 6.52 (t, J = 2.8 Hz, 1H), 3.16-3.11 (m, 2H), 1.17-1.13 (m, 2H), 0.39 (s, 6H).

^{13}C NMR (100 MHz, $CDCl_3$): (diastereomer mixture) δ 152.6, 151.4, 139.3, 138.8, 134.4, 134.3, 132.8, 128.6, 128.4, 126.7, 126.6, 126.5, 32.1, 31.7, 12.1, 10.7, −0.1, −0.3.

^{9}Si NMR (79 MHz, $CDCl_3$): δ 15.6, 12.9.

HRMS (APCI): $[M+H]^+$ calcd for $C_{12}H_{17}Si$: 189.1094, found: 189.1097.

7. Control experiments and mechanistic studies

(A) Reaction using dichlorodimethylsilane and styrene using the SmI_2/Sm system

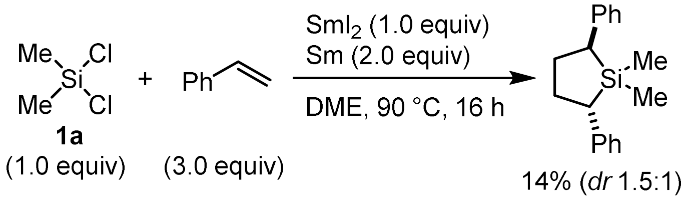

To a flame-dried Schlenk tube were added samarium powder (1.5 mmol, 225.5 mg), 1,2-diiodoethane (0.5 mmol, 140.9 mg) and anhydrous 1,2-dimethoxyethane (5.0 mL) under nitrogen atmosphere. The reaction mixture was stirred vigorously at room temperature for 40 min until a deep blue suspension was formed. Styrene (1.5 mmol, 156.2 mg) and dichlorodimethylsilane **1a** (0.5 mmol, 64.5 mg) were added dropwise to the mixture under nitrogen atmosphere sequentially. The mixture was stirred at 90 °C for 16 hours. After cooling to room temperature, the mixture was carefully quenched by saturated aq. NH_4Cl solution (5.0 mL) and extracted with diethyl ether (3 × 25 mL). The organic layer was dried over $MgSO_4$ or Na_2SO_4. The solvent was removed under reduced pressure to give the crude residue. A mixture of silacyclopentanes was detected, and the spectra were consistent with those reported in the literature.[9] The NMR yield of silacyclopentanes was determined using 1,3,5-trimethoxybenzene as an internal standard, giving 14% yield (*dr* = 1.5:1).

GC–MS analysis showed that, aside from the starting material and the desired product, neither residual Me_2SiCl_2 nor low-molecular-weight silane oligomers were detected (retention time of $(SiMe_2)_6$ = 20.4 min), suggesting the presence of higher-molecular-weight silicon-containing species.

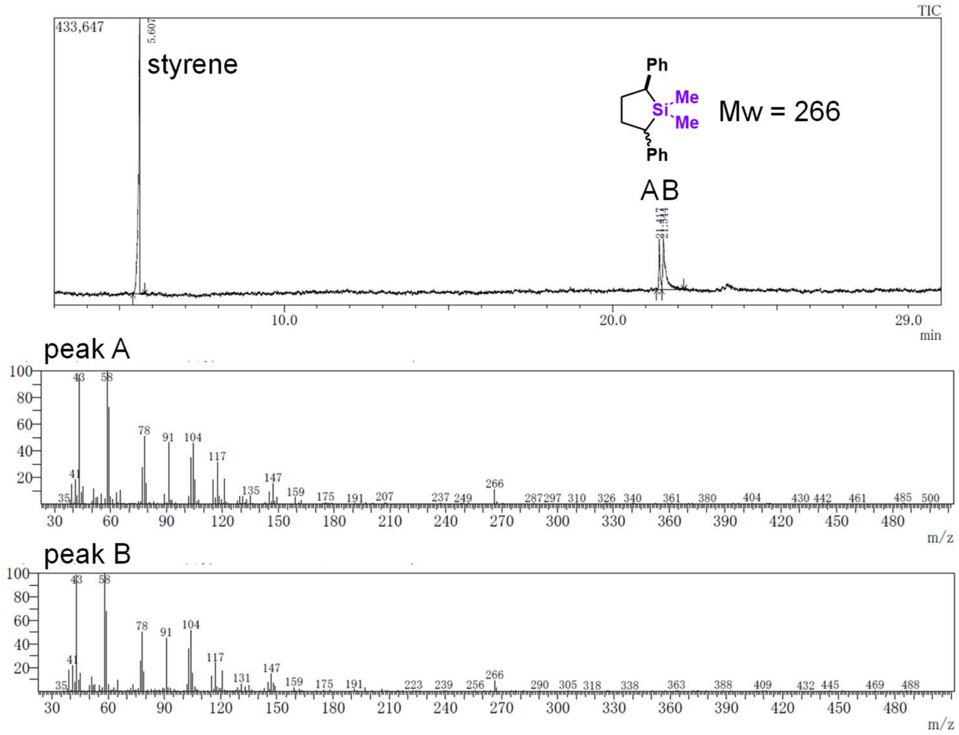

Figure S1. GC-MS spectra of the reaction mixture.

HRMS analysis of the crude mixtures revealed a series of peaks with an approximately constant mass interval of ca. 58 Da, indicative of polysilane.

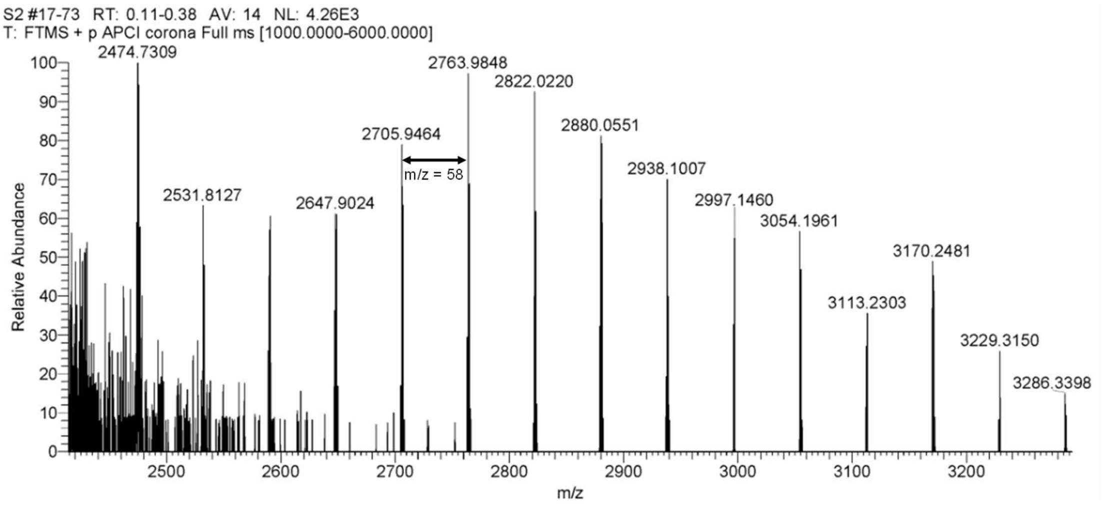

Figure S2. HRMS spectra of the polysilane.

(B) Reaction using Mg instead of Sm

Rieke's prior study suggested that Mg-promoted cyclization of 1,3-diene and dichlorosilanes proceeds via initial reduction of the diene into dianion.[10] Treatment of 2,3-diphenyl-1,3-butadiene with magnesium powder led to the quantitative formation of a Mg–diene complex, as confirmed by ^{1}H NMR spectroscopy (*vide infra*). In contrast, no analogous species was detected when samarium powder was used, highlighting a pronounced mechanistic divergence between the two systems.

For comparison, reactions employing Mg instead of Sm were performed under identical conditions as described before. Before the reaction, Mg powder was vigorously stirred under N_2 atmosphere for at least 1 h for activation. SmI_2 was freshly prepared in another Schlenk tube. Before being carefully transferred to the reaction mixture, the SmI_2 suspension was filtered to avoid participation of Sm metal.

Scheme S1. Control experiments using Mg instead of Sm

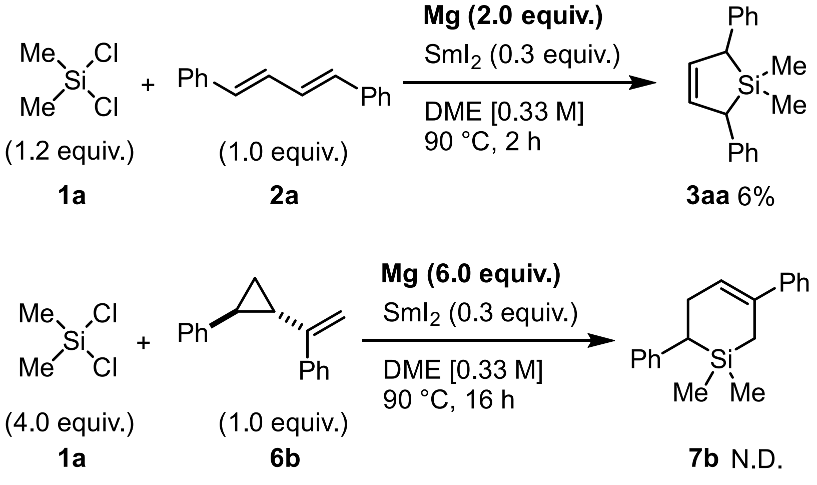

Scheme S2. Reaction of 2,3-diphenyl-1,3-butadiene with Mg

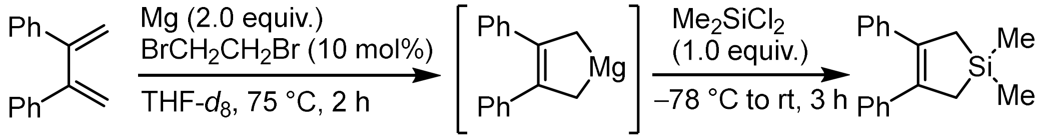

2b detected by ^{1}H NMR **3ab** 66%

Before the reaction, Mg powder was vigorously stirred under N_2 atmosphere for at least 1h for activation. To a flame-dried Schlenk tube was added dry THF-d_8 (0.6 mL), diene **2b** (0.15 mmol, 30.9 mg), Mg powder (0.3 mmol, 7.2 mg) and 1,2-dibromodiethane (0.015 mmol, 2.8 mg) under N_2 atmosphere. The mixture was stirred at 75 °C for 2 h. After the reaction finished, the Schlenk tube was moved into the glovebox and the clear deep red solution was transferred to an NMR tube for measurement.

The ^{1}H NMR spectrum of the mixture was shown below. After NMR measurement, the red solution was transferred to a flame-dried Schlenk tube under positive N_2 atmosphere. Then, Me_2SiCl_2 (0.15 mmol, 19.4 mg) was added dropwise at –78 °C to the solution until the red color gradually faded. The cold bath was removed and the mixture was stirred for another 3 h. Saturated NH_4Cl aq. was added to quench the reaction, followed by extraction with ether for three times. The organic layer was collected and dried by $MgSO_4$, filtrated and evaporated to give the crude mixture. The NMR yield was measured by using 1,3,5-trimethoxybenzene as standard (66%).

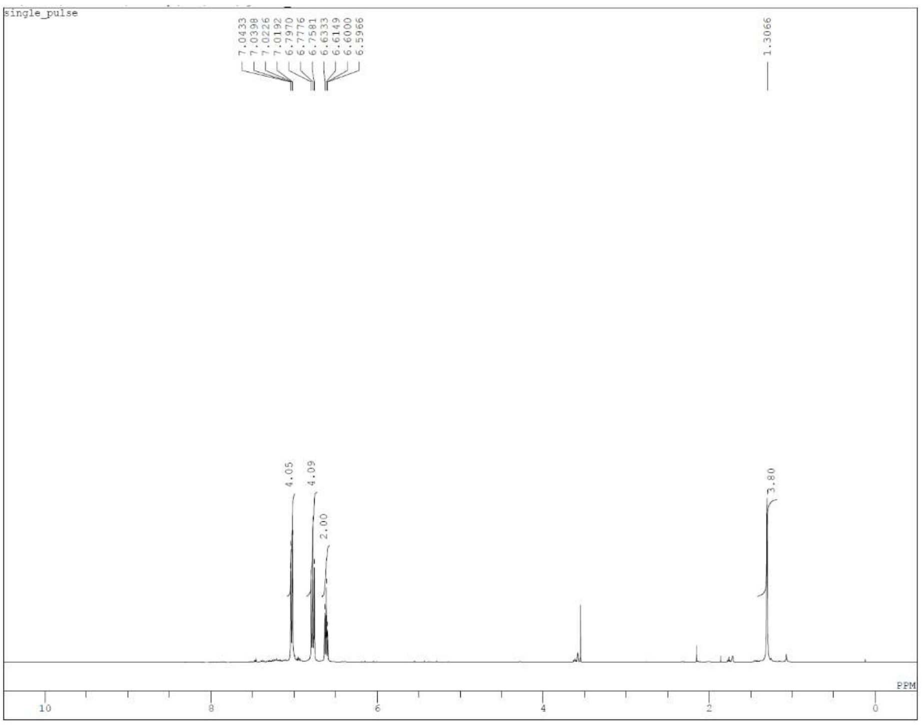

Figure S3. ^{1}H NMR of the Mg-diene complex (THF-d_8, 400 MHz, rt): 7.04-7.02 (m, 4H), 6.80-6.76 (m, 4H), 6.63-6.60 (m, 4H), 1.31 (s, 4H).

(C) UV-vis spectra

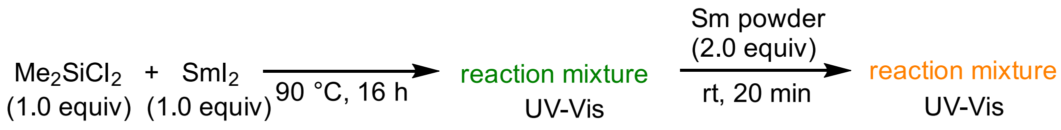

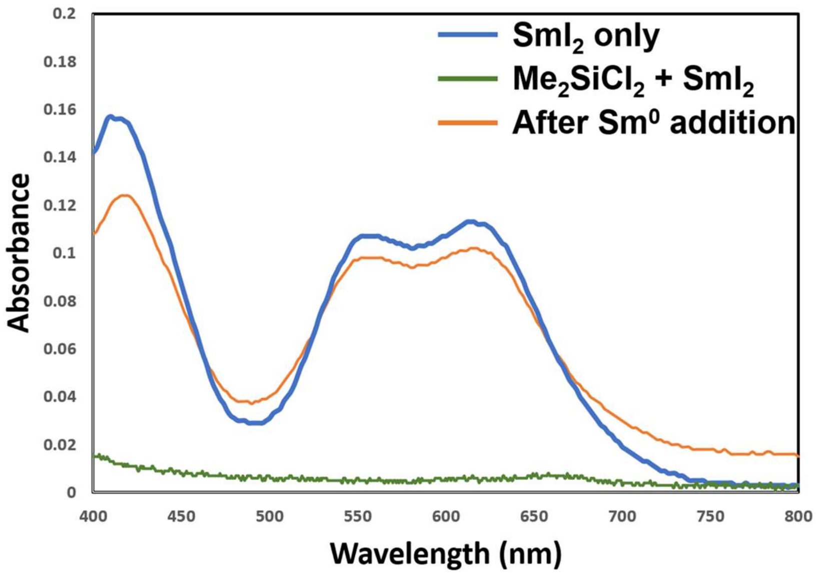

Figure S4. UV-vis spectra of 1.0 mM SmI_2 (blue), 1.0 mM SmI_2 with 1.0 mM Me_2SiCl_2 at 90 °C for 16 h (green) and the reaction mixture after addition of 2.0 equiv Sm powder addition (orange).

Me_2SiCl_2 (0.2 mmol) was added dropwise to a freshly-prepared SmI_2 solution (0.2 mmol, 0.1 M) in DME under positive N_2 flowing in a flame-dried Schlenk tube. After stirring at 90 °C for 16 h, the resulting colorless solution was transferred into a glovebox. Inside the glovebox, the reaction mixture was diluted to 1.0 mM using a volumetric flask and transferred to a screw-cap quartz cuvette for UV-vis measurements. The Schlenk tube was then removed from the glovebox, and Sm powder (0.4 mmol) was added. After 20 min stir at rt, the solution turned deep blue and UV-vis measurement was performed following the same procedure as described above.

(D) Cyclic voltammetry studies

All CV studies were conducted by electrochemical analyzer (610C-S, ALS Co., Ltd., Japan) under N_2 atmosphere. Tetrabutylammonium hexafluorophosphate ($TBAPF_6$) was used as supporting electrolyte. THF (anhydrous, inhibitor-free) was purchased from Sigma-Aldrich and degassed by N_2 bubbling for 40 min prior to use. The working electrode is a glassy carbon disk and was polished by aluminum oxide, sonicated in Milli-Q water and dried in vacuo for 16 h before use. RE-7N Non Aqueous reference electrode with Ag wire was used as the reference electrode. The reference electrode was soaked in a 0.01 M $TBAPF_6$ and saturated $AgNO_3$ solution in THF overnight before the experiment. The counter electrode is a Pt wire, which was dried in an oven at 150 °C for 6 h.

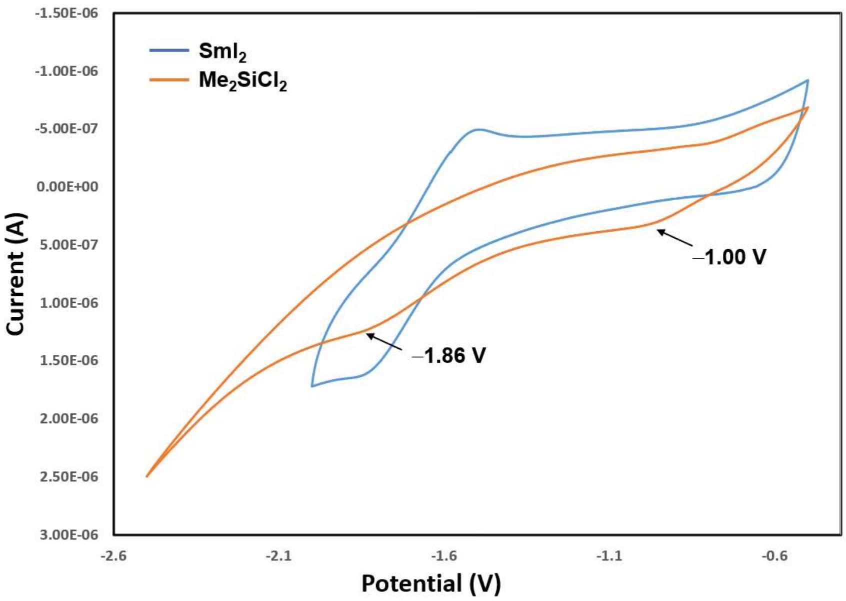

Figure S5. Cyclic voltammograms of 2.5 mM SmI_2 (blue) and 2.5 mM Me_2SiCl_2 (orange) with 0.01 M $TBAPF_6$ in degassed THF at 50 mV/s. Half wave potential of SmI_2 (–1.62 V) matches reported value in THF (–1.55 V).[11] Two reductive peaks of Me_2SiCl_2 were observed at –1.00 V and –1.86 V (vs. Ag/$AgNO_3$).

Several studies have reported the reduction potentials of chlorosilanes.[12,13] However, the reported values are sometimes contradictory, leading to ambiguity in mechanistic interpretations. Hengge,[14] Duchek,[15] Jammegg,[16] and Hoddenbagh[17] reported low cathodic potentials for chlorosilanes, whereas Corriu,[18] Zhuikov,[19] and Wang[20] reported higher values. The reduction potentials measured by cyclic voltammetry (CV) can vary significantly depending on the experimental conditions. Our measurements revealed two irreversible reduction events for Me_2SiCl_2, corresponding to the sequential reduction of the Si–Cl bonds.[14] In combination with our UV–vis spectra, these results suggest that SmI_2 reduces the Si–Cl bond via a one-electron process to generate silyl radicals, whereas over-reduction to form a silyl anion is unlikely under these conditions.

(E) Reaction outcomes in the absence of SmI_2 or Sm

Table S1. Reactions between **1a** and **1b** under different conditions.

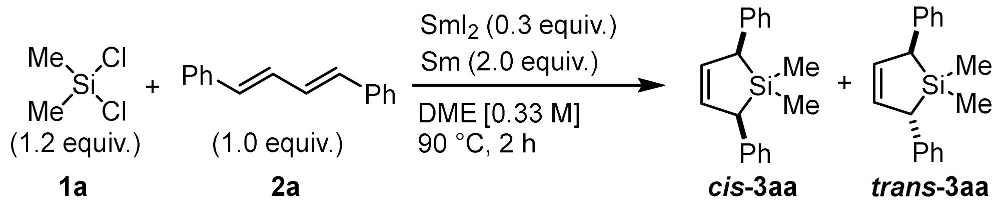

Deviation from standard condition	**3aa** (%)
none	76 (*cis:trans* = 92:8)
no SmI_2 (16 h)	37 (*cis:trans* = 93:7)
no Sm	0

Table S2. Reactions between **1a** and **6b** under different conditions.

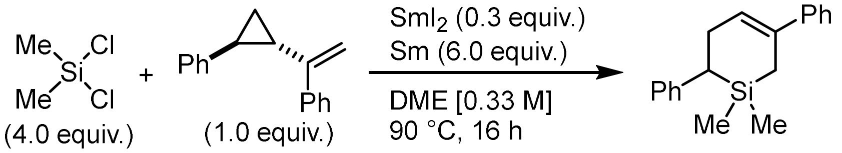

1a **6b** **7b**

Deviation from standard condition	**7c** (%)
none	74
no SmI_2	0
no Sm	0

Both Sm and SmI_2 are essential for the reaction. In the case of 1,3-butadiene, the diminished yield of silacyclopent-3-ene formation can be attributed to the over-reduction of Si-Cl bond by Sm alone, which generates a silyl anion that undergoes conjugate addition to butadiene to give the product. A similar mechanism has been previously reported by Ishikawa using an electroreduction method.[21]

However, no such product was obtained in the case of vinylcyclopropane, suggesting that a radical-mediated pathway operates instead.

(F) Anion probe experiment

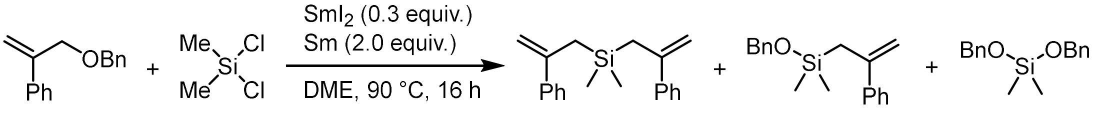

11 (1.0 equiv.) **1a** (1.2 equiv.) **12** 39% **13** 9% **14** 42%

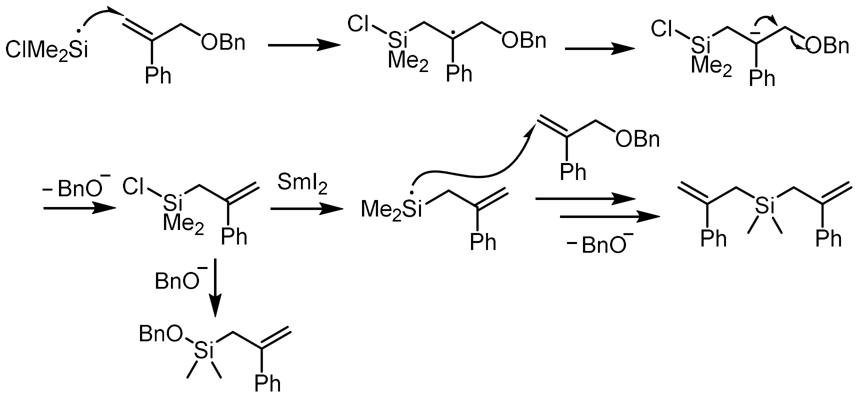

To a flame-dried Schlenk tube were added 1,2-diiodoethane (0.3 equiv, 0.09 mmol, 25.4 mg) and Sm powder (2.3 equiv, 0.69 mmol, 103.5 mg). Anhydrous DME was then introduced, and the suspension was vigorously stirred at rt for 40 min. Allyl benzyl ether **11** (0.3 mmol, 67.2 mg)[22] and Me_2SiCl_2 **1a** (0.36 mmol, 46.6 mg) was subsequently added, and the reaction mixture was stirred at 90 °C for 16 h. Upon completion, the mixture was quenched with saturated NH_4Cl aq., extracted with ether, dried over $MgSO_4$ and filtered. The organic layer was concentrated under reduced pressure, and the yields of were determined by ^1H NMR using 1,3,5-trimethoxybenzene as an internal standard. The crude product was further purified by silica gel column chromatography (hexane) to afford a

mixture of **12** and **13** as a colorless oil.

Dimethylbis(2-phenylallyl)silane (12)

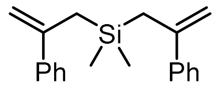

12

^{1}H NMR (400 MHz, CDCl$_3$): δ 7.38-7.33 (m, 4H), 7.31-7.22 (m, 6H), 5.11 (d, J = 1.4 Hz, 2H), 4.82 (dt, J = 1.4, 0.9 Hz, 2H), 1.96 (d, J =0.9 Hz, 4H), –0.26 (s, 6H).
^{13}C NMR (100 MHz, CDCl$_3$): δ 146.3, 142.7, 128.2, 127.4, 126.4, 110.6, 24.8, –2.9.
HRMS (APCI): [M+H]$^+$ calcd for $C_{20}H_{25}Si$: 293.1720, found: 293.1722.

(Benzyloxy)dimethyl(2-phenylallyl)silane (13)

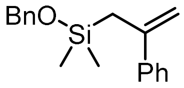

13

^{1}H NMR (400 MHz, CDCl$_3$): δ 7.38-7.22 (m, 10H), 5.15 (d, J = 1.4 Hz, 1H), 4.88 (dt, J = 1.4, 0.9 Hz, 1H), 2.04 (d, J = 0.9 Hz, 2H), 2.02 (s, 2H), –0.16 (s, 6H).

(G) Investigation of background reactions: reduction of butadiene and vinylcyclopropane by SmI$_2$/Sm

Table S3. Reduction of **1b** under different conditions.

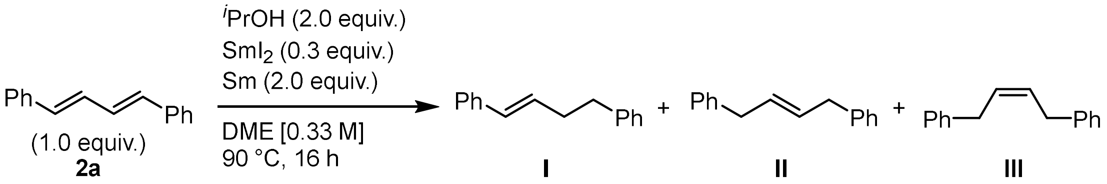

Deviation from standard condition	Yield (%)
none	**I** : 19, **II** : 10, **III** : 10
no SmI$_2$	0
no Sm	0

Table S4. Reduction of **6b** under different conditions.

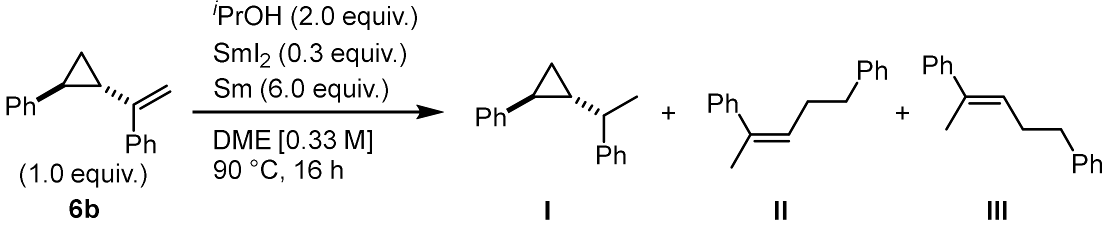

Deviation from standard condition	Yield (%)
none	**I** : 0, **II** : 0, **III** : 23
no SmI$_2$	0
no Sm	0

Under the standard conditions, the reduction of BDE or VCP could also occur but is regarded as a minor pathway. The alkene reduction followed by nucleophilic attack on dichlorosilanes may generate identical carbon-centered radicals (Scheme S9, minor pathway). In contrast, the predominant pathway involves reduction of the Si–Cl bond to furnish the desired products.

(H) DFT studies

General All DFT calculations were carried out with Gaussion 09.[23] Allyl radicals **INT1** and anions **INT2** were computed at the UB3LYP/6-311+G(d,p) level.[24]

(1) Formation of *cis*-3aa and *trans*-3aa

Scheme S3. Formation of ***cis*-3aa** and ***trans*-3aa** from **INT1-3aa**.

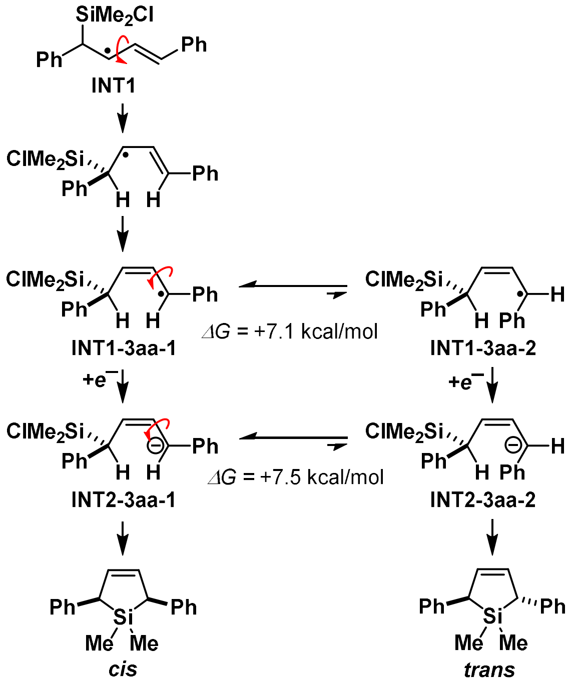

INT1-3aa-1 is 7.1 kcal/mol lower in energy than **INT1-3aa-2**, which is the dominant intermediate controlled by allylic strain. The maximum along the rotational coordinate corresponds to a rotational energy barrier of approximately 11.0 kcal·mol^{-1}.

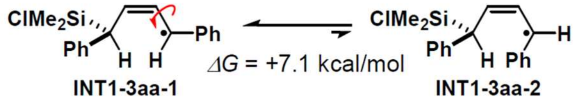

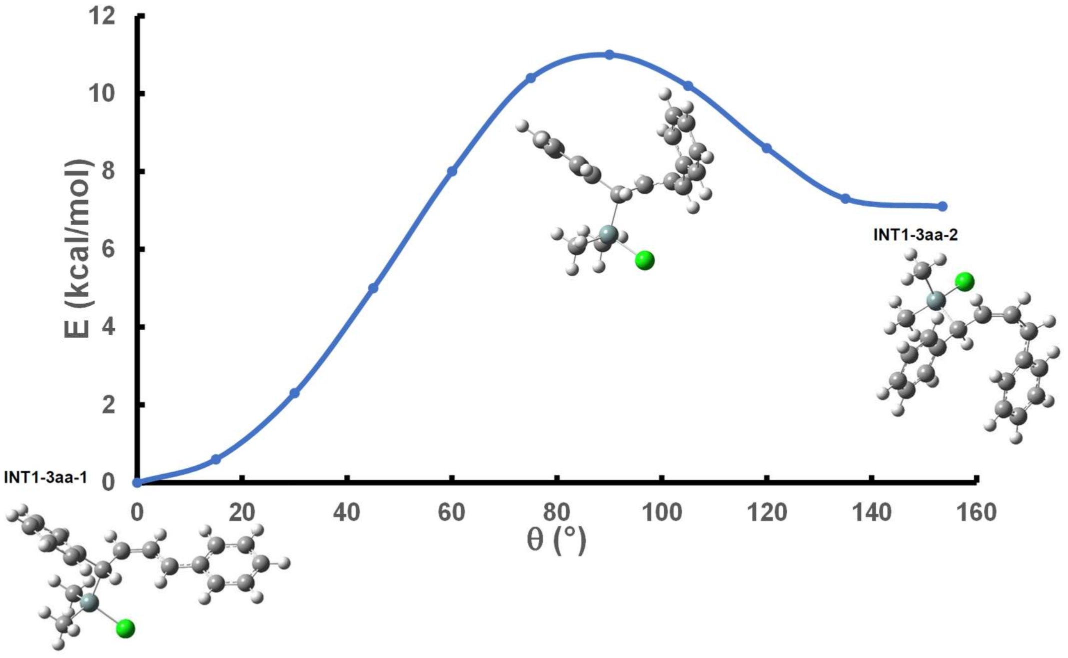

INT1-3aa-1

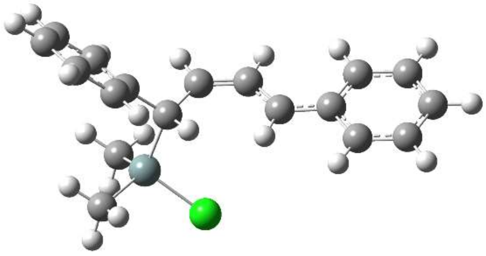

Atom	Coordinates X	(Angstroms) Y	Z
C	-1.562718	0.539299	0.000353
C	-1.561895	0.536949	1.376733
C	-0.366770	0.535993	2.281922
C	-0.447041	0.530186	-0.849290
Si	0.031257	-1.227885	2.953350
C	1.453188	-1.180299	4.174412
C	-1.472638	-2.098334	3.656602
Cl	0.686878	-2.362967	1.301458
C	-0.465860	0.555090	-2.287467
C	0.762778	0.524996	-2.990476
C	0.805799	0.549743	-4.376933
C	-0.377421	0.604387	-5.116966
C	-1.603257	0.634034	-4.445507
C	-1.651769	0.609764	-3.059517
C	-0.480202	1.508175	3.449749
C	0.573712	2.387405	3.729530
C	0.509015	3.273616	4.802680
C	-0.616689	3.300242	5.623410
C	-1.674583	2.433198	5.357169
C	-1.606044	1.546950	4.283787
H	-2.545233	0.553820	-0.462760
H	-2.530285	0.544395	1.866023
H	0.527204	0.808067	1.712587
H	0.539868	0.488703	-0.400641
H	1.728690	-2.195784	4.471955
H	2.336393	-0.706591	3.738462
H	1.176332	-0.620721	5.072068
H	-1.213627	-3.123486	3.934891
H	-2.284231	-2.142874	2.926737
H	-1.837017	-1.588738	4.553215
H	1.687263	0.479208	-2.424192
H	1.763194	0.524744	-4.885722
H	-0.346356	0.623425	-6.200202
H	-2.527368	0.676489	-5.011736
H	-2.616630	0.635187	-2.567990
H	1.452765	2.384000	3.093046
H	1.337006	3.947740	4.992501
H	-0.671209	3.990951	6.456991

H	-2.558673	2.446267	5.985149
H	-2.444766	0.885918	4.096246

Sum of electronic and zero-point Energies= -1447.611107
Sum of electronic and thermal Energies= -1447.590121
Sum of electronic and thermal Enthalpies= -1447.589177
Sum of electronic and thermal Free Energies= -1447.664551

INT1-3aa-2

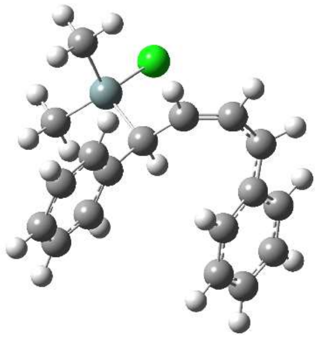

	Coordinates	(Angstroms)	
Atom	X	Y	Z
C	1.103561	-0.652755	0.777962
C	0.296139	-1.708709	1.130257
C	0.031193	-2.976384	0.377053
C	2.060231	-0.484572	-0.248042
Si	-1.731286	-2.913267	-0.406080
C	-2.086891	-4.455460	-1.411413
C	-3.071446	-2.586258	0.863966
Cl	-1.743492	-1.296207	-1.757937
C	2.874719	-1.451582	-0.944549
C	3.458737	-1.096067	-2.183396
C	4.280588	-1.975865	-2.873200
C	4.569086	-3.234700	-2.340154
C	4.030195	-3.594266	-1.102801
C	3.197370	-2.721788	-0.413740
C	0.164345	-4.260313	1.187216
C	0.702888	-5.406322	0.585107
C	0.816240	-6.604929	1.285919
C	0.391272	-6.686202	2.610736
C	-0.143892	-5.555469	3.224114
C	-0.256237	-4.357093	2.520904
H	0.930376	0.251232	1.360775
H	-0.321932	-1.545160	2.008402
H	0.696940	-3.048024	-0.484925
H	2.253267	0.548949	-0.524573
H	-3.052385	-4.357727	-1.915437
H	-1.323944	-4.616007	-2.177238
H	-2.121160	-5.341027	-0.771260

H	-4.044469	-2.517558	0.369988
H	-2.901627	-1.648581	1.398026
H	-3.120523	-3.398576	1.594977
H	3.243713	-0.118581	-2.602428
H	4.702944	-1.681121	-3.827558
H	5.217905	-3.919395	-2.874240
H	4.273841	-4.556421	-0.665295
H	2.822701	-3.000909	0.562912
H	1.045805	-5.354680	-0.442918
H	1.242123	-7.474179	0.796717
H	0.481178	-7.616667	3.159500
H	-0.472747	-5.602187	4.256547
H	-0.667523	-3.491110	3.025932

Sum of electronic and zero-point Energies= -1447.600346
Sum of electronic and thermal Energies= -1447.579444
Sum of electronic and thermal Enthalpies= -1447.578499
Sum of electronic and thermal Free Energies= -1447.653289

INT2-3aa-1

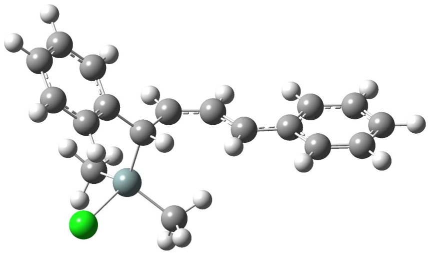

	Coordinates	(Angstroms)	
Atom	X	Y	Z
C	1.673026	1.852104	2.183592
C	0.766435	0.945720	1.653895
C	-0.538148	0.535684	2.291042
C	1.637090	2.582695	3.374178
Si	0.056094	-1.118583	3.001177
C	0.638264	-2.326924	1.674700
C	1.284315	-0.893013	4.404219
Cl	-1.559468	-2.274179	3.962198
C	2.624829	3.513178	3.830338
C	2.435871	4.214945	5.057349
C	3.359236	5.125087	5.546374
C	4.539768	5.404178	4.848858
C	4.754997	4.731962	3.640610
C	3.837109	3.819559	3.143445
C	-1.776466	0.496223	1.418284
C	-3.043159	0.312913	1.996839
C	-4.196663	0.282731	1.221237
C	-4.118383	0.427064	-0.164929

C	-2.871501	0.610043	-0.754598
C	-1.716292	0.648012	0.027262
H	2.557813	1.997359	1.562974
H	1.012706	0.485970	0.703419
H	-0.750571	1.180471	3.149843
H	0.772061	2.472209	4.024509
H	0.749181	-3.325815	2.105113
H	-0.093124	-2.388631	0.864014
H	1.596394	-2.016513	1.254049
H	1.850364	-1.815886	4.564818
H	0.763406	-0.656503	5.335315
H	1.975239	-0.075680	4.182023
H	1.528346	4.021162	5.623359
H	3.157474	5.628541	6.489157
H	5.263019	6.116663	5.230960
H	5.662721	4.925584	3.073190
H	4.054427	3.325757	2.202435
H	-3.118004	0.186080	3.071165
H	-5.161200	0.145263	1.700170
H	-5.017317	0.400368	-0.772455
H	-2.792768	0.734406	-1.830601
H	-0.755966	0.829391	-0.438709

Sum of electronic and zero-point Energies= -1447.658503
Sum of electronic and thermal Energies= -1447.637319
Sum of electronic and thermal Enthalpies= -1447.636375
Sum of electronic and thermal Free Energies= -1447.710956

INT2-3aa-2

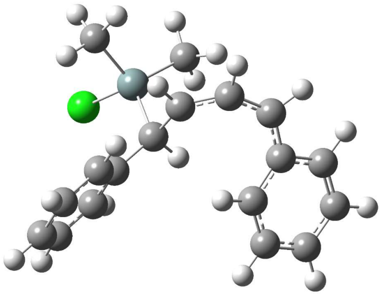

	Coordinates	(Angstroms)	
Atom	X	Y	Z
C	0.725978	-0.965466	-1.224043
C	0.532137	0.055013	-0.299921
C	-0.647745	0.287543	0.611965
C	0.098802	-2.188559	-1.514560
Si	-1.543407	1.521810	-0.514100
C	-0.537397	3.069979	-0.908514
C	-2.263820	0.687430	-2.034558
Cl	-3.325339	2.407625	0.435944

C	-0.727879	-3.041364	-0.711819
C	-1.466516	-4.098103	-1.320996
C	-2.242729	-4.981285	-0.589311
C	-2.332504	-4.878426	0.804789
C	-1.595747	-3.873111	1.435222
C	-0.812780	-2.983118	0.709670
C	-0.374033	0.726510	2.034846
C	-1.418323	0.777931	2.973205
C	-1.191144	1.168625	4.288009
C	0.091209	1.528704	4.705915
C	1.137806	1.480387	3.790311
C	0.908761	1.079739	2.473336
H	1.530510	-0.732282	-1.928556
H	1.260292	0.860239	-0.329384
H	-1.297757	-0.590738	0.641786
H	0.347090	-2.604295	-2.489671
H	-1.188620	3.836968	-1.336407
H	-0.090018	3.478428	0.002098
H	0.262706	2.851451	-1.618338
H	-2.519129	1.431333	-2.795788
H	-3.176697	0.146992	-1.772696
H	-1.551564	-0.032038	-2.447072
H	-1.420021	-4.197828	-2.402578
H	-2.793614	-5.761481	-1.109727
H	-2.947875	-5.564270	1.377536
H	-1.618770	-3.786858	2.518773
H	-0.203951	-2.263647	1.241835
H	-2.420729	0.509139	2.659894
H	-2.018412	1.191106	4.990671
H	0.269330	1.836478	5.731406
H	2.144021	1.746023	4.101193
H	1.736639	1.008673	1.778706

Sum of electronic and zero-point Energies= -1447.647407
Sum of electronic and thermal Energies= -1447.626441
Sum of electronic and thermal Enthalpies= -1447.625497
Sum of electronic and thermal Free Energies= -1447.699052

(2) Reduction of INT1-3aa to INT2-3aa

Scheme S4. Reduction of **INT1-3aa** to **INT2-3aa**.

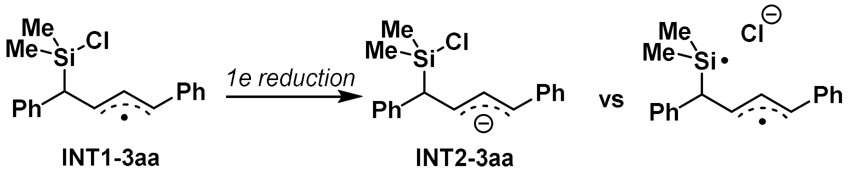

The NPA charge distributions are presented in **Figure S6**, and the SOMO of **INT1-3aa** is shown in **Figure S8**. The computational results reveal a pronounced increase in electron density on the allyl carbons (from −0.165, −0.141 to −0.422, −0.370), corresponding to the site of the SOMO. On the other hand, no considerable difference in both NPA charge and Si-Cl bond length suggests that the reduction of allyl radical is prior to which of Si-Cl bond, which aligns with both experimental data and reported studies.[25] Besides, the β-silicon effect can be rationalized in terms of a σ(Si-C)–π hyperconjugative interaction, which leads to stabilization of an occupied bonding combination (HOMO-1) and a relative elevation of the SOMO, as shown in **Figures S8** and **S9**.

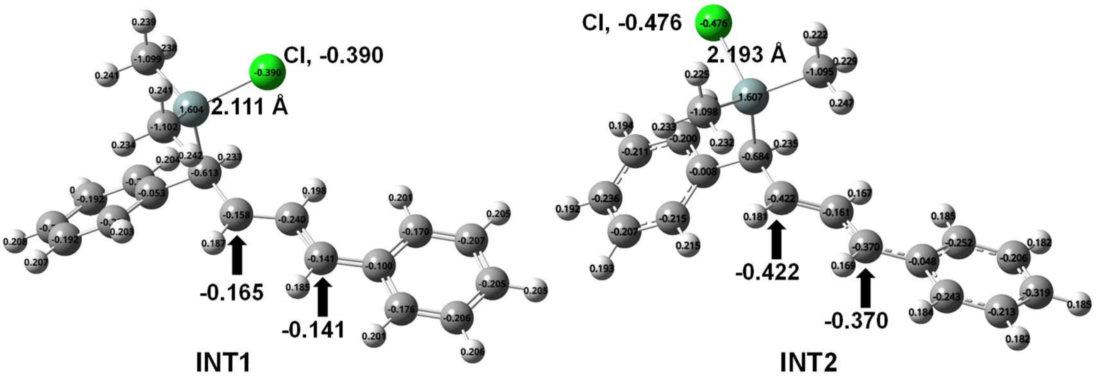

Figure S6. NPA charge computed at UB3LYP/6-311+G(d,p) level.

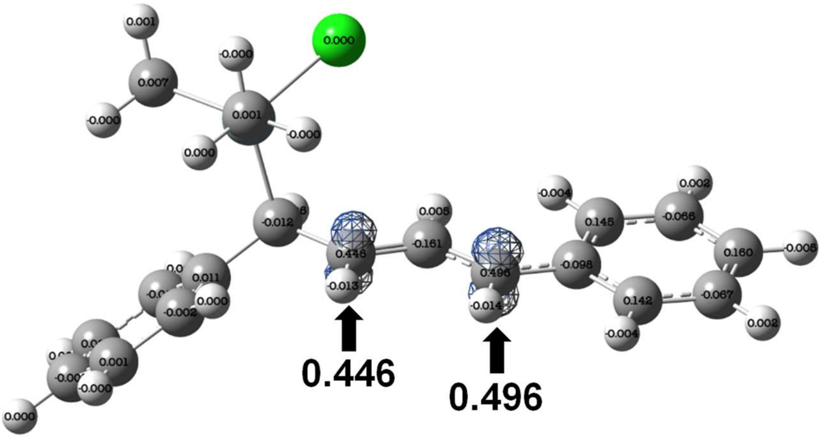

Figure S7. Spin density of **INT1-3aa**. Isovalue = 0.025.

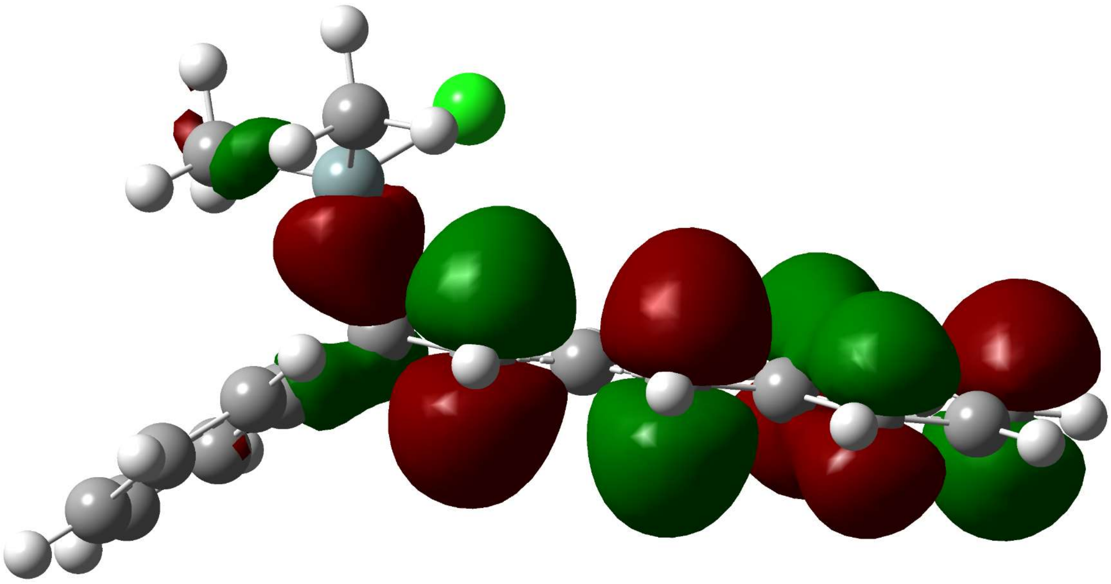

Figure S8. SOMO of **INT1-3aa**.

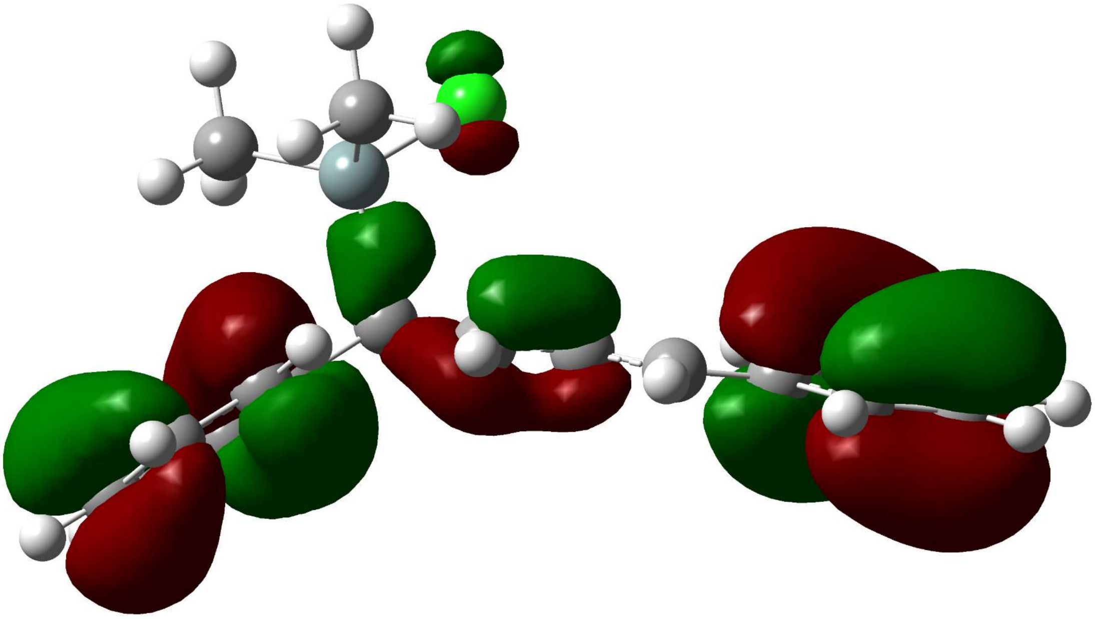

Figure S9. HOMO-1 of **INT1-3aa**.

INT1-3aa

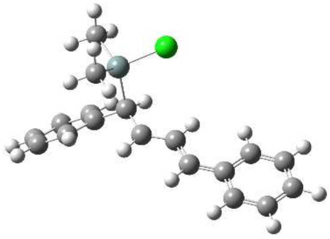

	Coordinates	(Angstroms)	
Atom	X	Y	Z

C	-0.705632	0.621972	0.904378
C	-0.154632	0.240255	-0.293773
C	0.589768	-1.039793	-0.519778
C	-1.387714	1.831198	1.106531
Si	2.489059	-0.810513	-0.259981
C	3.428839	-2.383654	-0.654848
C	3.174940	0.674894	-1.173676
Cl	2.749913	-0.448337	1.803189
C	-1.989765	2.293204	2.326579
C	-2.683812	3.527609	2.329335
C	-3.285109	4.018277	3.479277
C	-3.216469	3.296028	4.672872
C	-2.533784	2.076087	4.695737
C	-1.930095	1.580907	3.549501
C	0.329870	-1.694332	-1.867793
C	0.015623	-3.057745	-1.934554
C	-0.205393	-3.688241	-3.157159
C	-0.116785	-2.965521	-4.345234
C	0.193298	-1.607863	-4.295609
C	0.415830	-0.980599	-3.071145
H	-0.604371	-0.062672	1.742604
H	-0.243929	0.917814	-1.138822
H	0.311558	-1.753079	0.264436
H	-1.483655	2.492700	0.248601
H	4.487264	-2.263938	-0.407812
H	3.043661	-3.231454	-0.082786
H	3.347864	-2.626828	-1.717869
H	4.228399	0.818765	-0.918905
H	2.636427	1.587387	-0.907860
H	3.107522	0.532684	-2.256249
H	-2.743366	4.095660	1.406567
H	-3.810239	4.966690	3.448661
H	-3.685286	3.677640	5.572465
H	-2.471394	1.510323	5.618985
H	-1.401860	0.636592	3.599475
H	-0.065870	-3.630634	-1.016359
H	-0.453403	-4.743720	-3.179540
H	-0.291644	-3.452778	-5.297549
H	0.261362	-1.032514	-5.212382
H	0.653011	0.077530	-3.059510

Sum of electronic and zero-point Energies= -1447.613768

Sum of electronic and thermal Energies= -1447.592652

Sum of electronic and thermal Enthalpies= -1447.591708

Sum of electronic and thermal Free Energies= -1447.667368

INT2-3aa

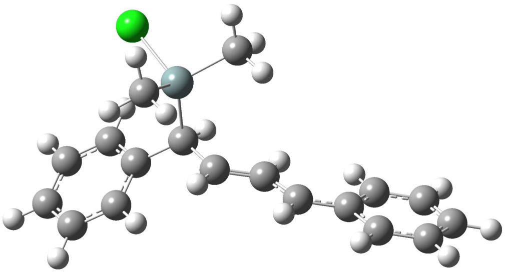

Atom	Coordinates X	(Angstroms) Y	Z
C	-1.865993	-0.260440	-2.754038
C	-0.994096	0.166438	-1.767300
C	-1.227233	-0.054243	-0.294019
C	-1.813934	-0.019248	-4.131144
Si	-1.815884	1.689520	0.177439
C	-0.496491	3.009297	-0.083889
C	-3.447384	2.134634	-0.643662
Cl	-2.287696	1.888423	2.310559
C	-2.715551	-0.513380	-5.121031
C	-2.539673	-0.161844	-6.494629
C	-3.389915	-0.607872	-7.494338
C	-4.480603	-1.438367	-7.202938
C	-4.677736	-1.805158	-5.864862
C	-3.831491	-1.366386	-4.857471
C	-0.097158	-0.624792	0.539814
C	-0.336985	-1.083767	1.845205
C	0.685324	-1.615662	2.625217
C	1.986937	-1.692256	2.124587
C	2.241948	-1.238722	0.832758
C	1.211699	-0.716264	0.048844
H	-2.708687	-0.858591	-2.398832
H	-0.126191	0.754043	-2.056614
H	-2.105087	-0.699831	-0.166085
H	-0.995963	0.598513	-4.500122
H	-0.823502	3.954256	0.358729
H	0.442523	2.719039	0.395499
H	-0.307773	3.170162	-1.147762
H	-3.807433	3.094758	-0.262435
H	-4.209162	1.380927	-0.424632
H	-3.335077	2.196533	-1.728109
H	-1.704160	0.482787	-6.755322
H	-3.202975	-0.304336	-8.521809
H	-5.145876	-1.787941	-7.984794
H	-5.511670	-2.452781	-5.604057
H	-4.026513	-1.688366	-3.840235
H	-1.339785	-1.016159	2.252948
H	0.466570	-1.970326	3.627677
H	2.786512	-2.103211	2.732359

H	3.246339	-1.299678	0.424599
H	1.413351	-0.399617	-0.967090

Sum of electronic and zero-point Energies= -1447.670522
Sum of electronic and thermal Energies= -1447.649136
Sum of electronic and thermal Enthalpies= -1447.648192
Sum of electronic and thermal Free Energies= -1447.723539

(3) Computational studies on the β-radical-effect and anion stabilization effect by the Si–Cl σ* orbital

A conformation in which the Si–C bond is oriented perpendicular to the allyl π system—thus minimizing σ(Si–C)–π(allyl) orbital interaction—was optimized (**Scheme S5**). This structure is higher in energy by 3.4 kcal/mol compared to the β-effect-stabilized geometry, indicating the energetic importance of the β- silicon-effect.

Scheme S5. β- silicon-effect in **INT-3aa-1**

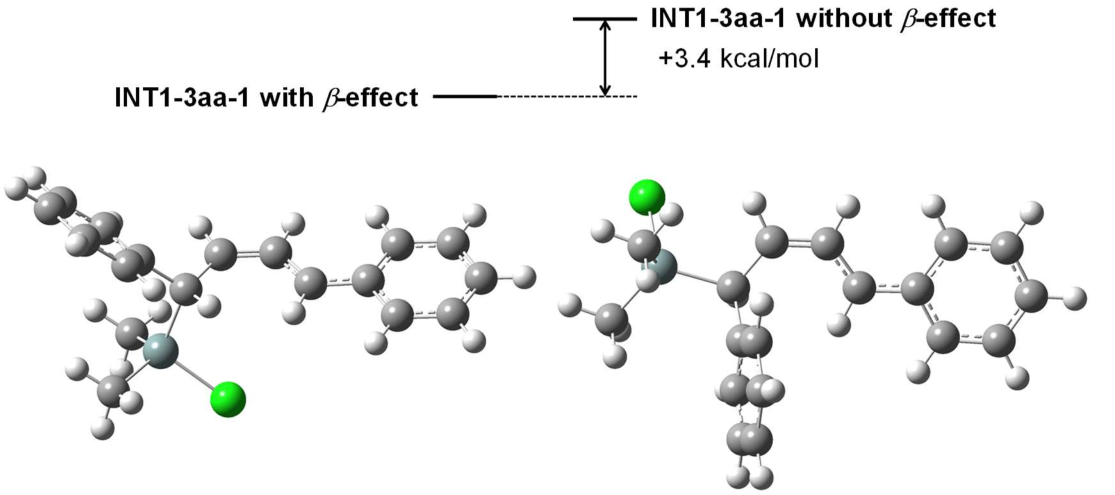

INT1-3aa-1 with β-effect **INT1-3aa-1 without β-effect**

Optimization of **INT1-3aa** by enforcing an overlap between the Si–Cl σ* orbital and the allyl radical and compared the relative energies of the resulting conformations. No significant energy differences were observed, indicating that the interaction effect is negligible.

Scheme S6. The effect of Si-Cl σ* orbital interaction with allyl radical in **INT1-3aa**

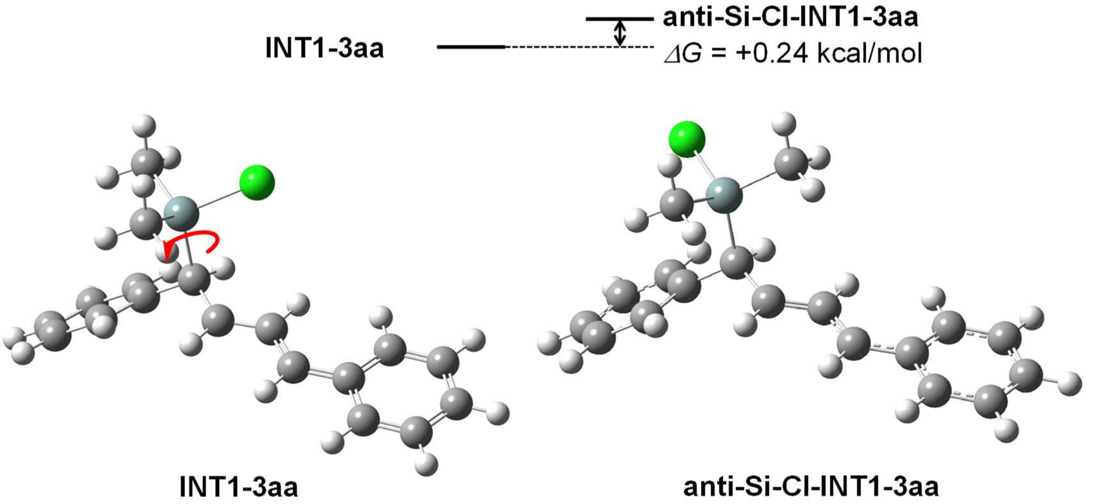

In contrast, when the same analysis was applied to the anionic intermediate **INT2**, the Si–Cl σ*-overlapping conformation (**anti-Si-Cl-INT2-3aa**) was 4.8 kcal/mol lower in energy, indicating a stabilization between the allyl anion and the Si–Cl σ* orbital.

Scheme S7. The effect of Si-Cl σ* orbital interaction with allyl anion in **INT2-3aa**

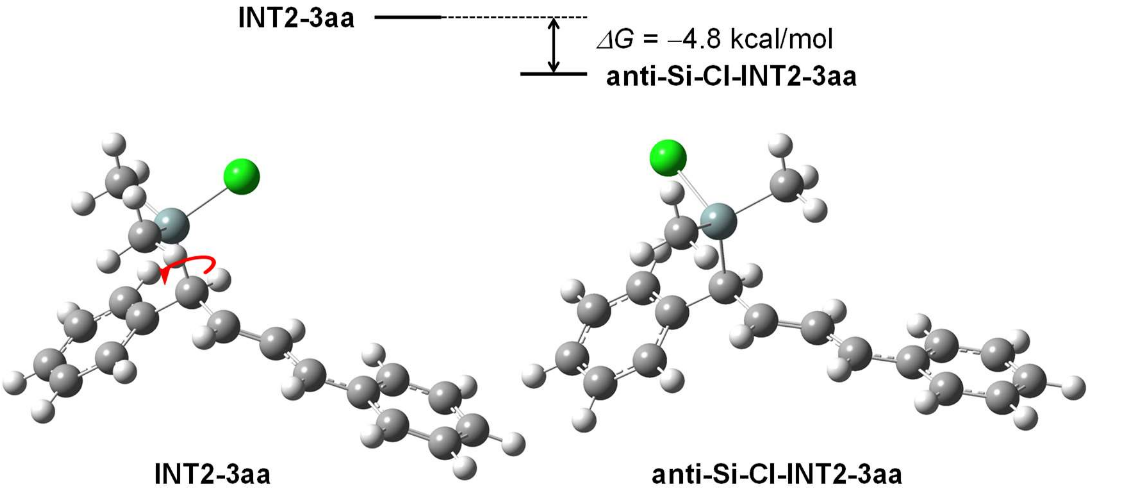

(I) Unsuccessful substrate of VCP

Scheme S8. Unsuccessful substrate for [5+1]-silacyclization reaction

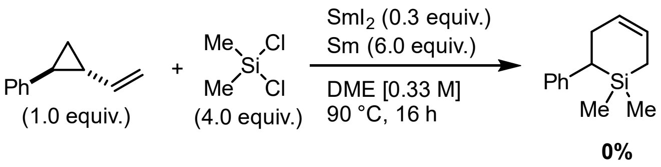

The reaction was performed following the typical procedure using VCP and dichlorosilanes.

(J) Plausible mechanisms

Scheme S9. Possible pathways.

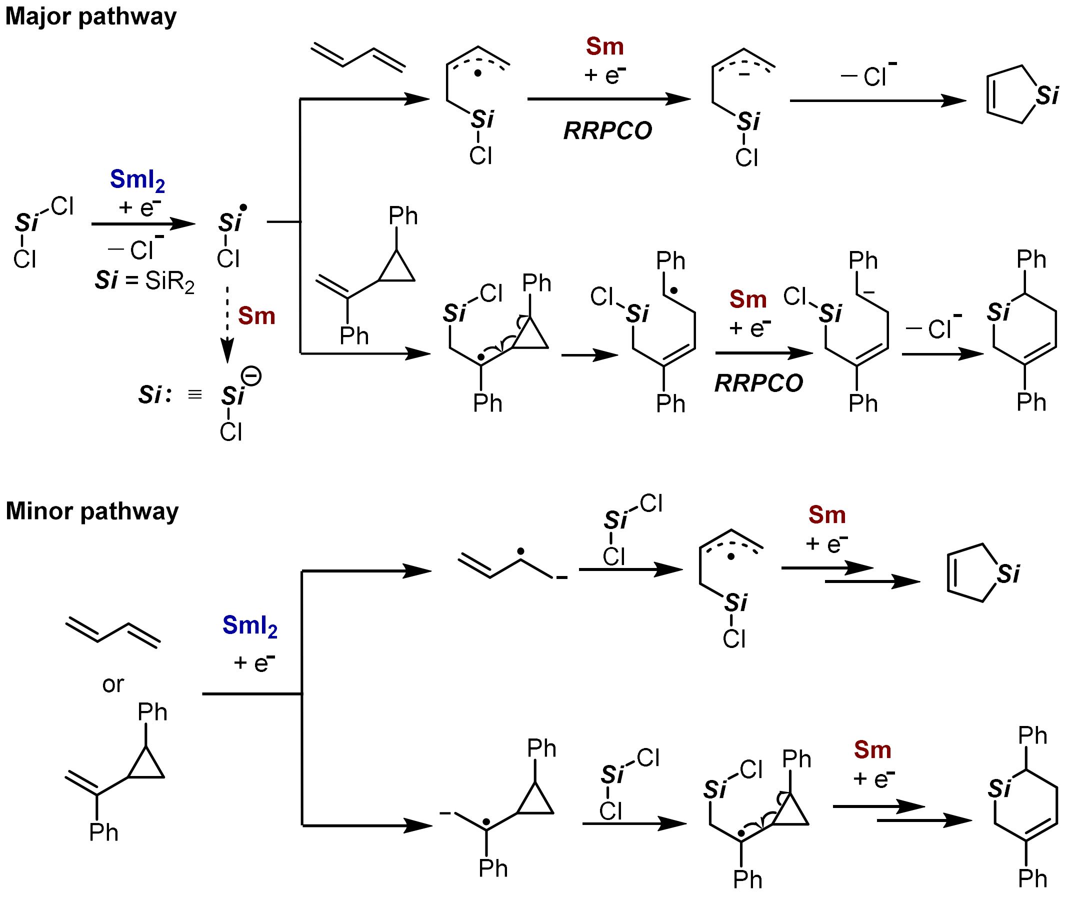

8. Derivatization of Silacarbocycles

(A) DDQ oxidation of 3ca

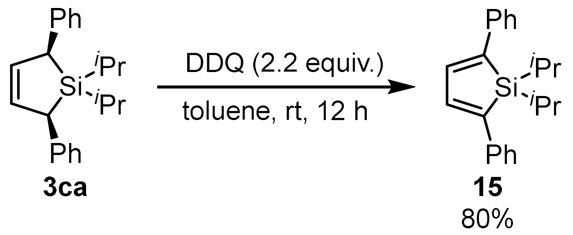

The procedure was modified from reported literature.[26] A Schlenk tube was flame-dried and refilled with N_2 prior to use. To a stirred solution of **3ca** (0.097 mmol, 31.0 mg, 1.0 equiv) in 0.9 mL dry toluene, DDQ (0.22 mmol, 48.4 mg, 2.2 equiv.) was added. The mixture was stirred at rt for 13 h until starting material was all consumed (monitored by TLC in Hex and GC-MS). Freshly distilled NEt_3 (0.58 mmol, 6.0 equiv) was added to the reaction mixture in 10 min. After addition of NEt_3, the solvent was evaporated and the residue was purified by column chromatography (Hexane) using silica gel to afford **15** (24.5 mg, 88%) as a yellow solid.

^{1}H NMR (400 MHz, $CDCl_3$): δ 7.42-7.39 (m, 4H), 7.34-7.31 (m, 4H), 7.27 (s, 2H), 7.23-7.19 (m, 2H), 1.58 (sept, J = 7.3 Hz, 2H), 1.05 (d, J = 7.3 Hz, 12H).

^{13}C NMR (100 MHz, $CDCl_3$): δ 143.1, 140.8, 140.5, 128.6, 126.8, 126.7, 17.8, 11.8.

Anal. Calcd. for $C_{22}H_{26}Si$: C, 82.96; H, 8.23. Found: C, 82.87; H, 8.37.

(B) Rh-catalyzed ring expansion of 3fa

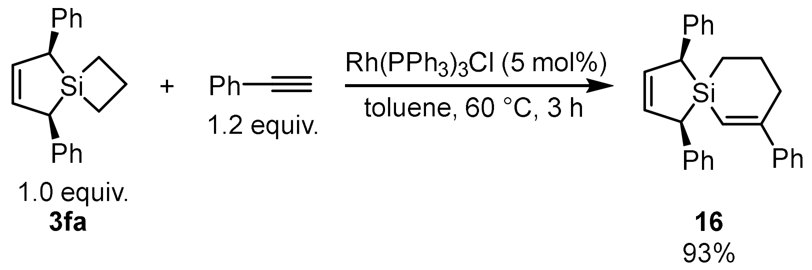

Compound **16** was prepared according to a reported procedure.[27] A Schlenk tube was flame-dried and refilled with N_2 prior to use. A mixture of **3fa** (0.2 mmol, 55.2 mg), phenylacetylene (0.24 mmol, 24.5 mg, 1.2 equiv), $Rh(PPh_3)_3Cl$ (0.01 mmol, 9.3 mg) in dry toluene (1.0 mL) was stirred at 60 °C for 3 h until all starting material was consumed (monitored by TLC Hexane/EtOAc = 10/1). The solvent was evaporated and the residue was purified by column chromatography (Hexane/EtOAc = 20/1) to afford **16** (70.5 mg, 93%) as a pale-yellow oil.

^{1}H NMR (400 MHz, $CDCl_3$): δ 7.47-7.45 (m, 2H), 7.37-7.34 (m, 2H), 7.31-7.24 (m, 5H), 7.12-7.07 (m, 6H), 6.30 (s, 1H), 6.17 (br s, 2H), 3.46 (s, 2H), 2.29 (t, J = 5.5 Hz, 2H), 1.28-1.24 (m, 2H), 0.05-0.02 (m, 2H).

^{13}C NMR (100 MHz, $CDCl_3$): δ 162.1, 144.3, 142.9, 135.1, 128.3, 128.2, 127.8, 126.7, 125.5, 124.4, 119.1, 38.8, 32.3, 21.2, 4.7.

^{9}Si NMR (79 MHz, $CDCl_3$): δ 8.8.

HRMS (EI): $[M+H]^+$ calcd for $C_{27}H_{27}Si$: 379.1877, found: 379.1896.

(C) Mizoroki-Heck reaction of 3aa

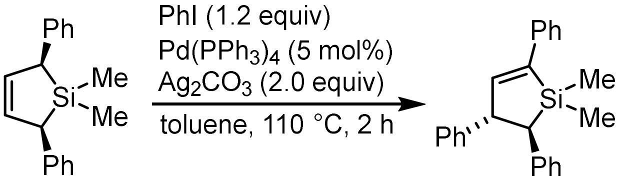

1.0 equiv. **3aa** → **17** 51%

The procedure was modified from reported literature.[28] To a flame-dried Schlenk tube were added $Pd(PPh_3)_4$ (5 mol%, 0.005 mmol, 5.8 mg), Ag_2CO_3 (2.0 equiv, 0.2 mmol, 55.1 mg) and anhydrous toluene (1.5 mL). Subsequently, **3aa** (0.1 mmol, 26.4 mg) and PhI (0.12 mmol, 24.5 mg) were added sequentially. The suspension was stirred at 110 °C until **3aa** was all consumed (monitored by TLC, hexane). The product exhibited fluorescence under blue light. Upon completion, the mixture was concentrated under reduced pressure and purified by silica gel column chromatography (hexane) to afford **17** (17.4 mg, 51%) as a colorless oil.

^{1}H NMR (400 MHz, $CDCl_3$): δ 7.46-7.43 (m, 2H), 7.38-7.33 (m, 2H), 7.28-7.24 (m, 4H), 7.21-7.09 (m, 7H), 7.03 (d, J = 2.8 Hz, 1H), 4.31 (dd, J = 6.9, 2.8 Hz, 1H), 2.58 (d, J = 6.9 Hz, 1H), 0.43 (s, 3H), 0.09 (s, 3H).

^{13}C NMR (100 MHz, $CDCl_3$): δ 147.7, 146.1, 143.4, 143.0, 130.8, 128.8, 128.6, 128.5, 127.2, 127.2, 127.0, 126.7, 126.3, 124.6, 56.4, 45.9, –0.6, –2.9.

^{9}Si NMR (79 MHz, $CDCl_3$): δ 18.5.

HRMS (APCI): $[M+H]^+$ calcd for $C_{24}H_{25}Si$: 341.1720, found: 341.1722.

(D) Hosomi-Sakurai reaction of 3co

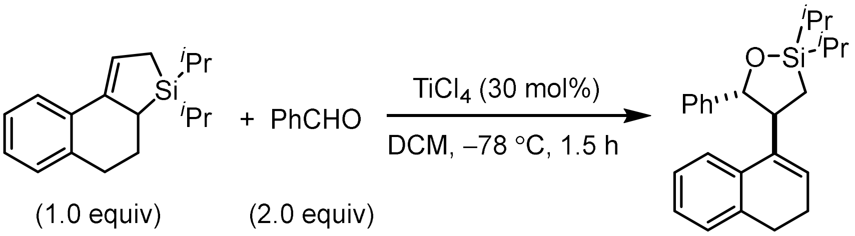

(1.0 equiv) **3co** (2.0 equiv) → **18** 48% ***dr*** 1.18:1

To a flame-dried Schlenk tube were added freshly-distilled benzaldehyde (0.2 mmol, 21.2 mg) anhydrous DCM (0.8 mL). The solution was cooled down to –78 °C and $TiCl_4$ (1.0 M in toluene solution) was added dropwise carefully. The reaction mixture was stirred at –78 °C for 5 min, and **3co** (0.1 mmol, 27.0 mg) was added dropwise. The mixture was stirred under –78 °C until complete consumption of **3co** monitored by TLC (TLC Silica gel 60 RP-18 F_{254}s) using MeCN as an eluent. The reaction mixture was quenched by anhydrous EtOH at –78 °C until the yellow color completely turned colorless. The suspension was then concentrated under reduced pressure and purified by reverse-phase column chromatography (MeCN) to afford a crude mixture containing **18**. The yield was determined by ^{1}H NMR using 1,3,5-trimethoxybenzene as an internal standard (48%, *anti:syn* = 1.18:1). Isomers of **18** could be further separated and purified by GPC.

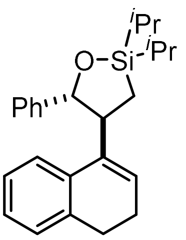

***anti*-18**

^{1}H NMR (400 MHz, $CDCl_3$): δ 7.33-7.31 (m, 2H), 7.21-7.17 (m, 2H), 7.15-7.11 (m, 1H), 7.08-7.03 (m, 3H), 6.94-6.92 (m, 1H), 6.04 (t, *J* = 4.6 Hz, 1H), 4.95 (d, *J* = 10.0 Hz, 1H), 3.07 (ddd, *J* = 12.4, 10.0, 6.9 Hz, 1H), 2.66 (sep, *J* = 7.3 Hz, 1H), 2.64 (sep, *J* = 7.3 Hz, 1H), 2.22 (td, *J* = 7.8, 4.6 Hz, 2H), 1.41 (dd, *J* = 15.1, 6.9 Hz, 1H), 1.26-1.18 (m, 9H), 1.12-1.04 (m, 6H).

^{13}C NMR (100 MHz, $CDCl_3$): δ 143.1, 139.0, 136.8, 135.1, 128.1, 127.5, 127.3, 126.4, 126.3, 126.2, 124.6, 122.4, 84.6, 48.6, 28.6, 23.2, 17.6, 17.6, 17.4, 17.3, 16.3, 12.8, 12.7.

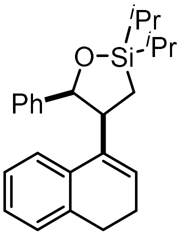

***syn*-18**

^{1}H NMR (400 MHz, $CDCl_3$): δ 7.42-7.41 (m, 1H), 7.37-7.35 (m, 2H), 7.24-7.22 (m, 2H), 7.19-7.15 (m, 1H), 7.12-7.03 (m, 2H), 6.92-6.90 (m, 1H), 6.30 (ddd, *J* = 10.5, 4.1, 1.4 Hz), 5.57 (d, *J* = 2.8 Hz, 1H), 3.03-3.00 (m, 1H), 2.62-2.47 (m, 2H), 2.24 (ddd, *J* = 15.6, 4.1, 1.8 Hz, 1H), 1.80-1.65 (m, 2H),1.60 (dd, *J* = 15.6, 10.5 Hz, 1H), 1.23-1.16 (m, 2H), 1.08-1.07 (m, 6H), 1.03-1.00 (m, 6H).

^{13}C NMR (100 MHz, $CDCl_3$): δ 143.1, 139.7, 136.7, 136.4, 128.2, 127.7, 126.7, 126.6, 126.4, 125.8, 124.8, 121.6, 75.2, 46.9, 29.1, 23.0, 17.8, 17.7, 17.6, 17.5, 14.1, 12.9, 11.3.

HRMS (EI) of 18: M^+ calcd for $C_{25}H_{32}OSi$: 376.2222, found: 376.2222.

(E) Difluoromethylation

General procedure for difluoromethylation modified from reported literature.[29] Tetrabutylammonium bromide (TBAB), $TMSCF_2Br$, and corresponding silacarbocycles were stirred at 90 °C for 16 h. The reaction mixture was diluted with DCM and quenched by saturated $NaHCO_3$ aq. solution, further extracted with DCM for three times. The organic layer was collected, dried with anhydrous $MgSO_4$, filtered and concentrated under reduced pressure. The crude product was purified by silica gel column chromatography.

from 5a

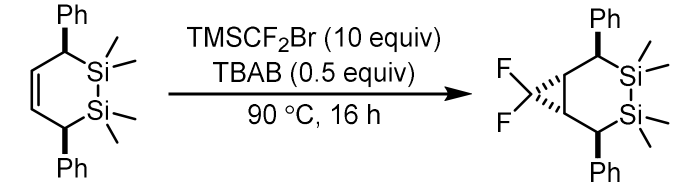

5a **19** 91%

Following the general procedure using **5a** (0.125 mmol, 40.5 mg), $TMSCF_2Br$ (1.3 mmol, 264.0 mg), and TBAB (0.063 mmol, 20.1 mg). Purification by column chromatography (hexane + 0.5% triethylamine) using silica gel afforded pure **19** as a white solid (42.5 mg, 91%).

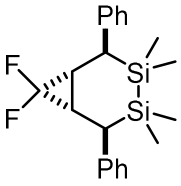

19

Only single isomer was obtained, whose configuration was confirmed by NOE.

^{1}H NMR (400 MHz, CDCl$_3$): δ 7.32-7.28 (m, 4H), 7.17-7.13 (m, 2H), 7.10-7.08 (m, 4H), 2.28-2.15 (m, 4H), 0.11 (s, 6H), 0.04 (s, 6H).

^{13}C NMR (100 MHz, CDCl$_3$): δ 141.1, 128.5, 127.2, 125.1, 113.8 (dd, $J_{C\text{-}F}$ = 298, 284 Hz), 26.9 (t, $J_{C\text{-}F}$ = 3.8 Hz), 25.1 (dd, $J_{C\text{-}F}$ = 12.5, 9.6 Hz), –4.5, –6.7.

^{29}Si NMR (79 MHz, CDCl$_3$): δ –14.8.

HRMS (APCI) of 20: $[M+H]^+$ calcd for $C_{21}H_{27}F_2Si_2$: 373.1614, found: 373.1616.

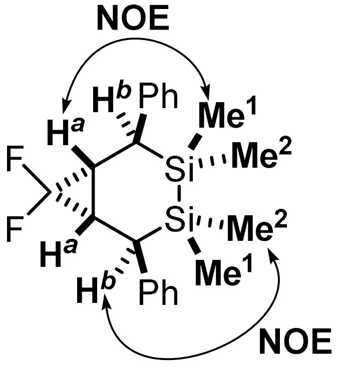

from 7b

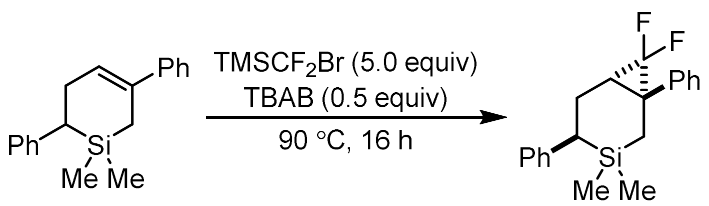

7b **20**
74% ***dr*** 1.14:1

Following the general procedure using **7b** (0.15 mmol, 41.7 mg), $TMSCF_2Br$ (0.75 mmol,

142.2 mg), and TBAB (0.075 mmol, 24.2 mg). Purification by column chromatography (hexane/EtOAc = 40/1 + 0.5% triethylamine) using silica gel afforded pure **20** as a white solid (36.4 mg, 74%).

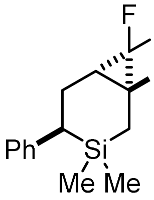

20

^{1}H NMR (400 MHz, CDCl$_3$):

Isomer **20a** (major): δ 7.36-7.22 (m, 7H), 7.16-7.09 (m, 3H), 2.67-2.50 (m, 2H, C*H*$_2$CHPh), 2.48-2.43 (m, 1H, C*H*Ph), 2.08-2.03 (m, 1H, C*H*CF$_2$),1.54 (dd, *J* = 15.5, 4.0 Hz, 1H, C*H*SiMe$_2$), 1.17 (ddd, *J* = 15.5, 5.5, 1.4 Hz, 1H, C*H'*SiMe$_2$), 0.00 (s, 3H, SiC*H*$_3$), –0.08 (s, 3H, SiC*H'*$_3$).

Isomer **20b** (minor): δ 7.37-7.22 (m, 7H), 7.16-7.11 (m, 1H), 7.09-7.03 (m, 2H), 2.37-2.31 (m, 2H, C*H*CHPh + C*H*Ph), 2.12-2.08 (m, 1H, C*H*CF$_2$), 1.81 (td, *J* = 14.6, 10.0 Hz, 1H, C*H'*CHPh), 1.28 (dd, *J* = 15.5, 1.4 Hz, 1H, C*H*SiMe$_2$) 1.11 (dd, *J* = 15.5, 4.0 Hz, 1H, C*H'*SiMe$_2$), 0.03 (s, 3H, SiC*H*$_3$), –0.37 (s, 3H, SiC*H'*$_3$).

^{13}C NMR (100 MHz, CDCl$_3$): 143.9, 143.7, 128.9, 128.8, 128.7, 128.6, 128.5, 128.4, 128.1, 128.0, 127.1, 127.0 (Ph^1, *o*-*C*H, **20a**), 126.9, 126.4 (Ph^1, *o*-*C*H, **20b**), 124.8 (**20b**), 124.6 (**20a**), 115.7 (dd, J_{C-F} = 294, 289 Hz, *C*F$_2$), 114.4 (dd, J_{C-F} = 293, 291 Hz, *C*F$_2$), 32.7 (dd, J_{C-F} = 11.5, 10.5 Hz, *C*PhCF$_2$, **20a**), 32.2 (t, J_{C-F} = 10.5 Hz, *C*PhCF$_2$, **20b**), 31.7 (d, J_{C-F} = 3.8 Hz, *C*HPh, **20b**), 28.2 (dd, J_{C-F} = 11.5, 9.6 Hz, *C*HCF$_2$, **20a**), 28.0 (d, J_{C-F} = 4.8 Hz, *C*HPh, **20a**), 27.0 (dd, J_{C-F} = 9.6, 8.6 Hz, *C*HPh, **20b**), 23.3 (*C*H$_2$CHPh, **20a**), 20.5 (d, J_{C-F} = 3.8 Hz, *C*H$_2$CHPh, **20b**), 15.4 (dd, J_{C-F} = 7.7, 2.9 Hz, *C*HSiMe$_2$, **20a**), 12.8 (dd, J_{C-F} = 6.7, 2.9 Hz, *C*HSiMe$_2$, **20b**), –1.5 (Si*C*H$_3$, **20b**), –3.2 (Si*C*H$_3$, **20a**), –4.1 (Si*C'*H$_3$, **20b**), –5.1 (Si*C'*H$_3$, **20a**).

^{29}Si NMR (79 MHz, CDCl$_3$): δ 1.6, –4.6.

HRMS (APCI) of 21: [M+H]$^+$ calcd for C$_{20}$H$_{23}$F$_2$Si: 329.1532, found: 329.1538.

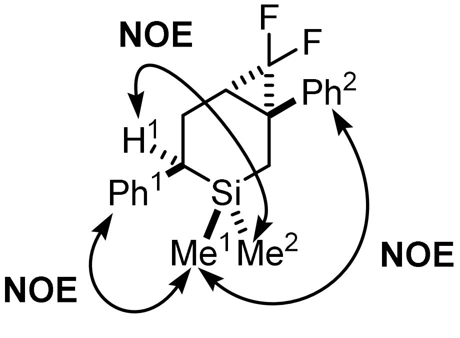

20a

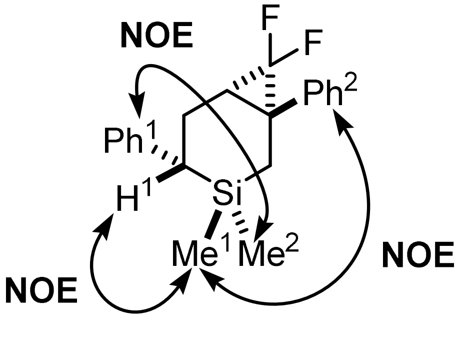

20b

9. References

[1] A. Hirao, Y. Sakano, K. Takenaka, S. Nakahama, "Anionic Living Polymerization of 2,3-Diphenyl-1,3-butadiene." *Macromolecules* **1998**, *31*, 9141–9145.

[2] T. Ohmura, K. Masuda, I. Takase, M. Suginome, "Palladium-Catalyzed Silylene-1,3-Diene [4+1] Cycloaddition with Use of (Aminosilyl)boronic Esters as Synthetic Equivalents of Silylene." *J. Am. Chem. Soc.* **2009**, *131*, 16624–16625.

[3] M. Ohashi, I. Takeda, M. Ikawa, S. Ogoshi, "Nickel-Catalyzed Dehydrogenative [4 + 2] Cycloaddition of 1,3-Dienes with Nitriles" *J. Am. Chem. Soc.* **2011**, *133*, 18018–18021.

[4] Y. Jiang, X. Kang, Z. Zhang, S. Li, D. Cui, "Syndioselective 3,4-Polymerization of 1-Phenyl-1,3-Butadiene by Rare-Earth Metal Catalysts." *ACS Catal.* **2020**, *10*, 5223–5229.

[5] D. Fiorito, S. Folliet, Y. Liu, C. Mazet, "A General Nickel-Catalyzed Kumada Vinylation for the Preparation of 2-Substituted 1,3-Dienes." *ACS Catal.* **2018**, *8*, 1392–1398.

[6] P. Bellotti, H.-M. Huang, T. Faber, R. Laskar, F. Glorius, "Catalytic Defluorinative Ketyl-Olefin Coupling by Halogen-Atom Transfer." *Chem. Sci.* **2022**, *13*, 7855–7862.

[7] C. Chen, X. Shen, J. Chen, X. Hong, Z. Lu, "Iron-Catalyzed Hydroboration of Vinylcyclopropanes." *Org. Lett.* **2017**, *19*, 5422–5425.

[8] F.-P. Wu, X.-F. Wu, "Catalyst-controlled selective borocarbonylation of benzylidenecyclopropanes: regiodivergent synthesis of *γ*-vinylboryl ketones and *β*-cyclopropylboryl ketones." *Chem. Sci.* **2022**, *13*, 4321–4326.

[9] D. Seyferth, D. P. Duncan, M. L. Shannon, E. W. Goldman, "Hexamethylsilirane. 5. Conversion to five-membered ring silicon compounds by two-atom insertion reactions of aryl olefins, 1,3-dienes and conjugated acetylenes." *Organometallics* **1984**, *3*, 574–578.

[10] R. D. Rieke, H. Xiong, "Chemistry of substituted (2-butene-1,4-diyl)magnesium: a facile approach to complex carbocycles, functionalized ketones and alcohols, and silicon-containing heterocycles." *J. Org. Chem.* **1991**, *56*, 3109–3118.

[11] A. Dahlén, Å. Nilsson, G. Hilmersson, "Estimating the Limiting Reducing Power of SmI_2/H_2O/Amine and YbI_2/H_2O/Amine by Efficient Reduction of Unsaturated Hydrocarbons." *J. Org. Chem.* **2006**, *71*, 1576–1580.

[12] V. V. Jouikov, "Electrochemical Reactions of Organosilicon Compounds." *Rus. Chem. Rev.* **1997**, *66*, 509–540.

[13] A. D. Beck, S. Haufe, S. R. Waldvogel, "General Concepts and Recent Advances in the Electrochemical Transformation of Chloro- and Hydrosilanes." *ChemElectroChem* **2023**, *10*, e202201149.

[14] E. Hengge, H. Firgoi, "An Electrochemical Method for the Synthesis of Silicon-Silicon Bonds." *J. Organomet. Chem.* **1981**, *212*, 155–161.

[15] P. Duchek, R. Ponec, V. Chvalovsky, "Experimental and Theoretical Study of Electrochemical Reduction of Halosilanes." *J. Organomet. Chem.* **1984**, *271*, 101–106.

[16] Ch. Jammegg, S. Graschy, E. Hengge, "New Aspects in the Electrochemical Polymerization of Organosilicon Compounds." *Organometallics* **1993**, *13*, 2397–2400.

[17] M. Hoddenbagh, D. Foucher, D. Worsfold, "Electrochemical Studies of Chlorine-Containing Silanes." *ChemRxiv*, Cambridge Open Engage, 2021. https://doi.org/10.26434/chemrxiv-2021-0hjxl

[18] R. J. P. Corriu, G. Dabosi, M. Martineau, "Electrochemical Reduction of

Triorganohalosilanes and -germanes." *J. Organomet. Chem.* **1980**, *188*, 63–72.
[19] V. V. Zhuikov, "Stepwise and Dissociative Mechanisms of the Electron Transfer in Electrochemical Reactions Involving Organosilicon Compounds: Molecular-Thermodynamic Approach." *Russ. J. Electrochem.* **2000**, *36*, 117–127.
[20] X. Wang, Y. Yuan, I. Cabasso, "Cyclic Voltammetric Study of Electroreduction of Dichlorosilanes." *J. Electrochem. Soc.* **2005**, *152*, E259–E264.
[21] A. Kunai, T. Ueda, E. Toyoda, M. Ishikawa, "Electrochemical Reduction of Dichlorosilanes in the Presence of 2,3-Dimethylbutadiene." *Bull. Chem. Soc. Jpn.* **1994**, *67*, 287–289.
[22] V. L. Tweedie, M. Cuscurida, "Hydrogenolysis by Metal Hydrides I. Hydrogenolysis of Aryl Allyl Ethers by Lithium Aluminum Hydride." *J. Am. Chem. Soc.* **1957**, *79*, 5463–5466.
[23] M. J. Frisch, G. W. Trucks, H. B. Schlegel, G. E. Scuseria, M. A. Robb, J. R. Cheeseman, G. Scalmani, V. Barone, G. A. Petersson, H. Nakatsuji, X. Li, M. Caricato, A. Marenich, J. Bloino, B. G. Janesko, R. Gomperts, B. Mennucci, H. P. Hratchian, J. V. Ortiz, A. F. Izmaylov, J. L. Sonnenberg, D. Williams-Young, F. Ding, F. Lipparini, F. Egidi, J. Goings, B. Peng, A. Petrone, T. Henderson, D. Ranasinghe, V. G. Zakrzewski, J. Gao, N. Rega, G. Zheng, W. Liang, M. Hada, M. Ehara, K. Toyota, R. Fukuda, J. Hasegawa, M. Ishida, T. Nakajima, Y. Honda, O. Kitao, H. Nakai, T. Vreven, K. Throssell, J. A. Montgomery, Jr., J. E. Peralta, F. Ogliaro, M. Bearpark, J. J. Heyd, E. Brothers, K. N. Kudin, V. N. Staroverov, T. Keith, R. Kobayashi, J. Normand, K. Raghavachari, A. Rendell, J. C. Burant, S. S. Iyengar, J. Tomasi, M. Cossi, J. M. Millam, M. Klene, C. Adamo, R. Cammi, J. W. Ochterski, R. L. Martin, K. Morokuma, Ö. Farkas, J. B. Foresman, D. J. Fox, "Gaussian 09, Revision A.02." Gaussian, Inc., Wallingford CT, 2016.
[24] (a) J. C. Slater, *Quantum Theory of Molecules and Solids, Vol. 4: The Self-Consistent Field for Molecules and Solids*; McGraw-Hill: New York, **1974**. (b) S. H. Vosko, L. Wilk, M. Nusair, *Can. J. Phys.* **1980**, *58*, 1200. (c) A. D. Becke, *Phys. Rev. A* **1988**, *38*, 3098. (d) C. Lee, W. Yang, R. G. Parr, *Phys. Rev. B* **1988**, *37*, 785. (e) A. D. Becke, *J. Chem. Phys.* **1993**, *98*, 5648.
[25] L. Lu, J. C. Siu, Y. Lai, S. Lin, "An Electroreductive Approach to Radical Silylation via the Activation of Strong Si-Cl Bond." *J. Am. Chem. Soc.* **2020**, *142*, 21272–21278.
[26] Y.-F. Zhang, L. Li, Y. Tang, Y.-Y. Zhou, "Catalytic Reductive (Double) [4+1] Sila-cycloaddition of 1,3-Dienes with Di-, Tri-, and Tetrachlorosilane(s) Enabled by the Pyridine–Diimine–Nickel Complex." *Org. Lett.* **2025**, *27*, 4032–4038.
[27] L. Qi, Q.-Q. Pan, X.-X. Wei, X. Pang, Z. Liu, X.-Z. Shu, "Nickel-Catalyzed Reductive [4+1] Silacycloaddition of 1,3-Dienes with Dichlorosilanes." *J. Am. Chem. Soc.* **2023**, *145*, 13008–13014.
[28] W.-S. Huang, F.-Y. Ling, X.-J. Cui, F. Ye, Z. Xu, J. Cao, H. Yang, L.-W. Xu, "Synthesis of 2,3-Dihydrosiloles via Palladium-Catalyzed Heck-type Arylation of Silacyclopentenes with Aryl Iodides." *Synthesis* **2023**, *55*, 1577–1585.
[29] (a) L. Li, F. Wang, C. Ni, J. Hu, "Synthesis of gem-Difluorocyclopropa(e)nes and O-, S-, N-, and P-Difluoromethylated Compounds with $TMSCF_2Br$." *Angew. Chem. Int. Ed.* **2013**, *52*,12390–12394. (b) E. Oftadeh, M. J. Wong, J. Yu, X. Li, Y. Cao, F. Gallou, L. Heinz, B. H. Lipshutz, "Reactions of In Situ-Generated Difluorocarbene ($:CF_2$) with Aromatic/Heteroaromatic Alcohols, Thiols, Olefins, and Alkynes under Environmentally

Responsible Conditions." *J. Org. Chem.* **2024**, *89*, 17331–17337.

10. Copies of ^{1}H, ^{13}C and ^{29}Si NMR spectra

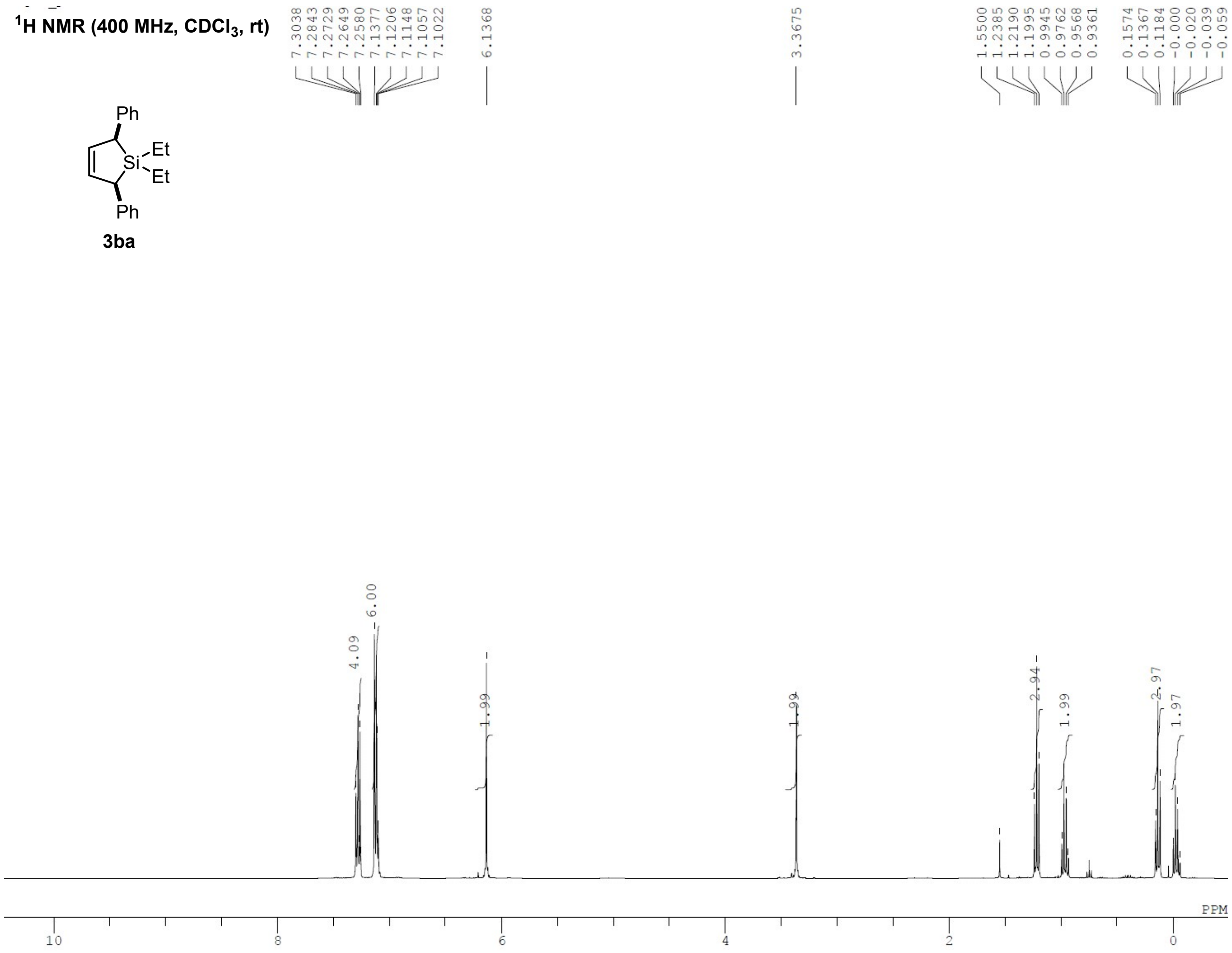

^{13}C NMR (100 MHz, $CDCl_3$, rt)

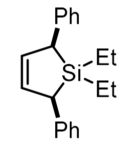

3ba

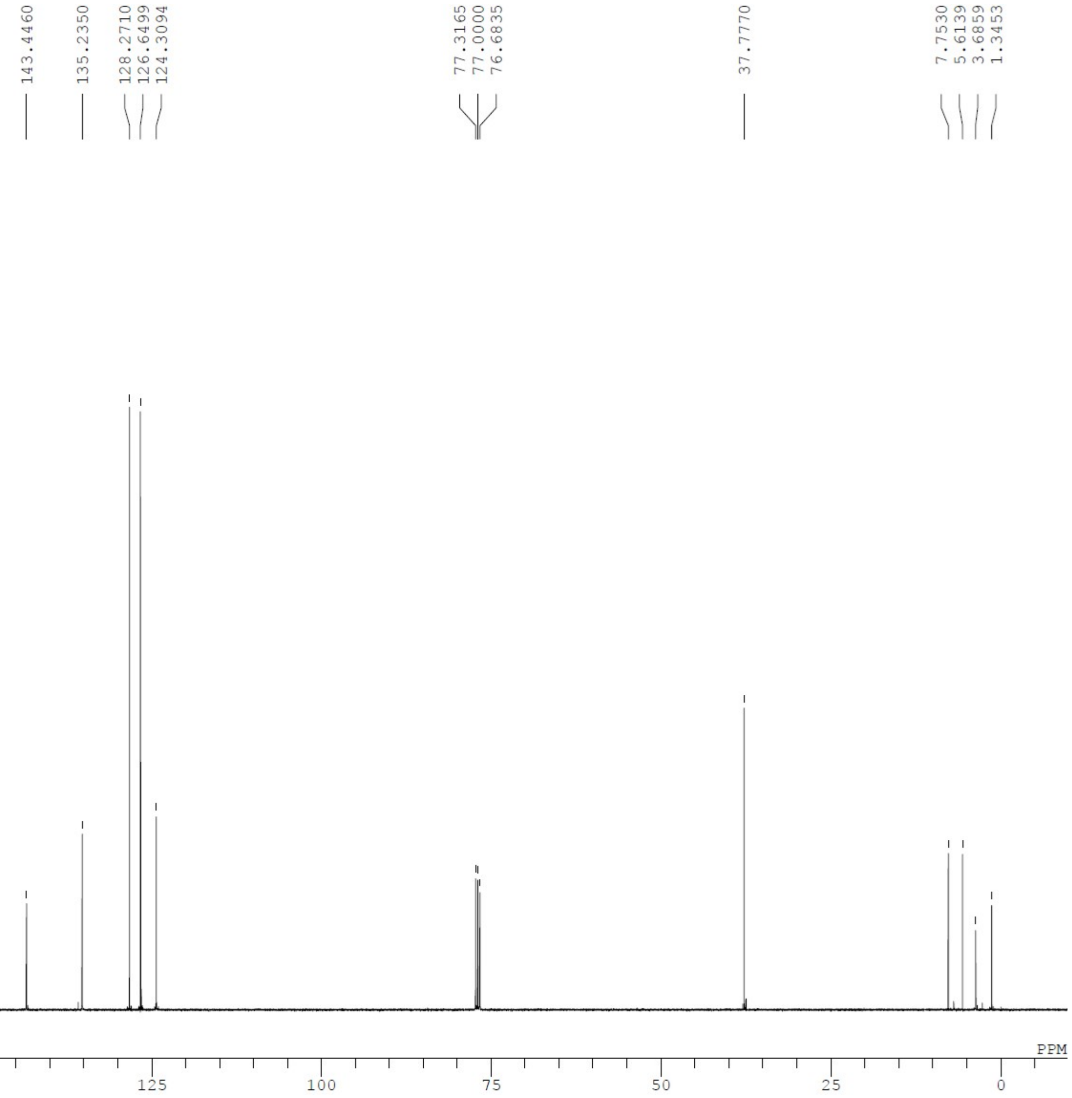

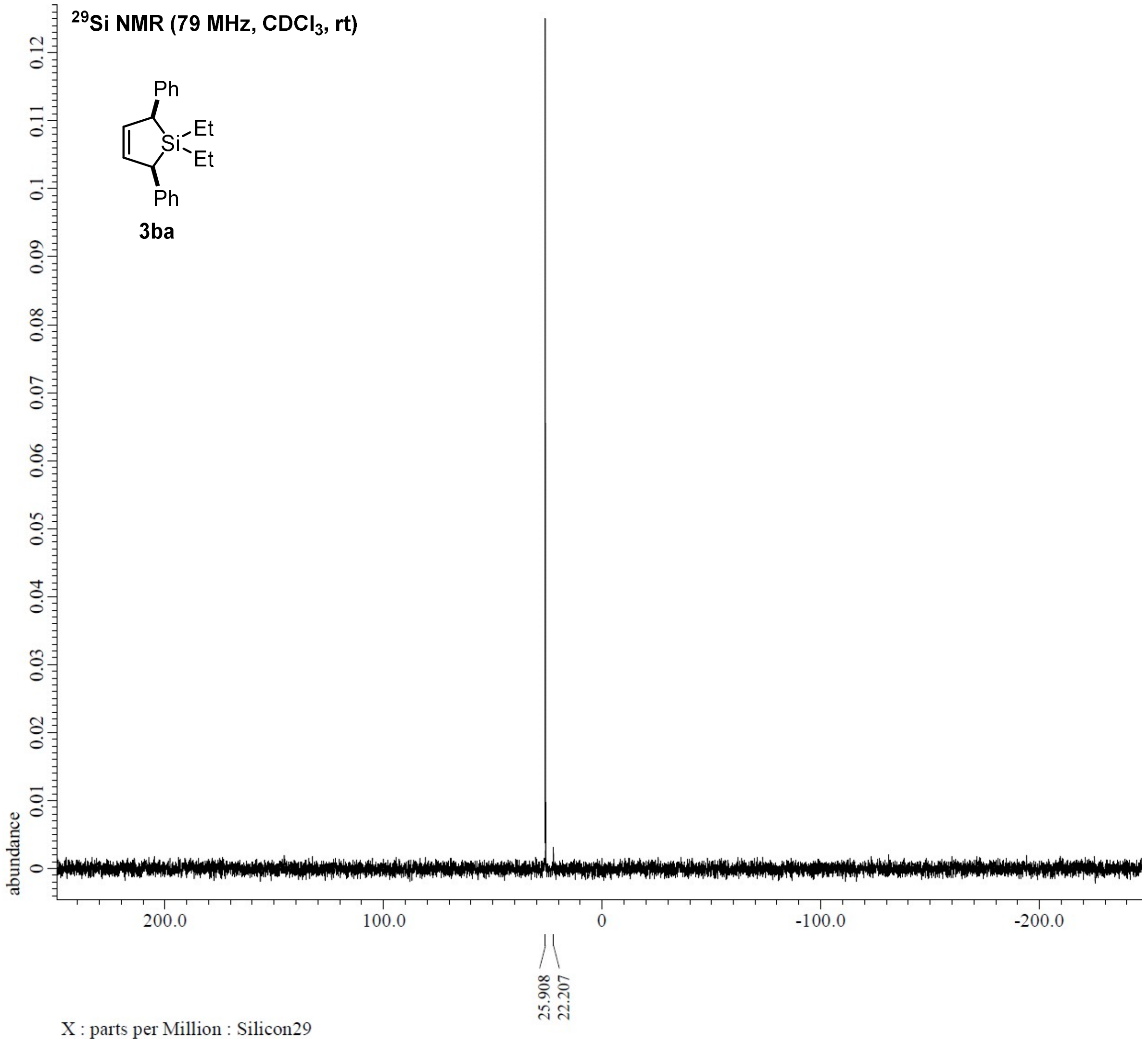

29Si NMR (79 MHz, CDCl3, rt)
Ph
Si
Et
Et
Ph
3ba
abundance
0.12
0.11
0.1
0.09
0.08
0.07
0.06
0.05
0.04
0.03
0.02
0.01
0
200.0
100.0
0
-100.0
-200.0
25.908
22.207
X : parts per Million : Silicon29

^1H NMR (400 MHz, $CDCl_3$, rt)

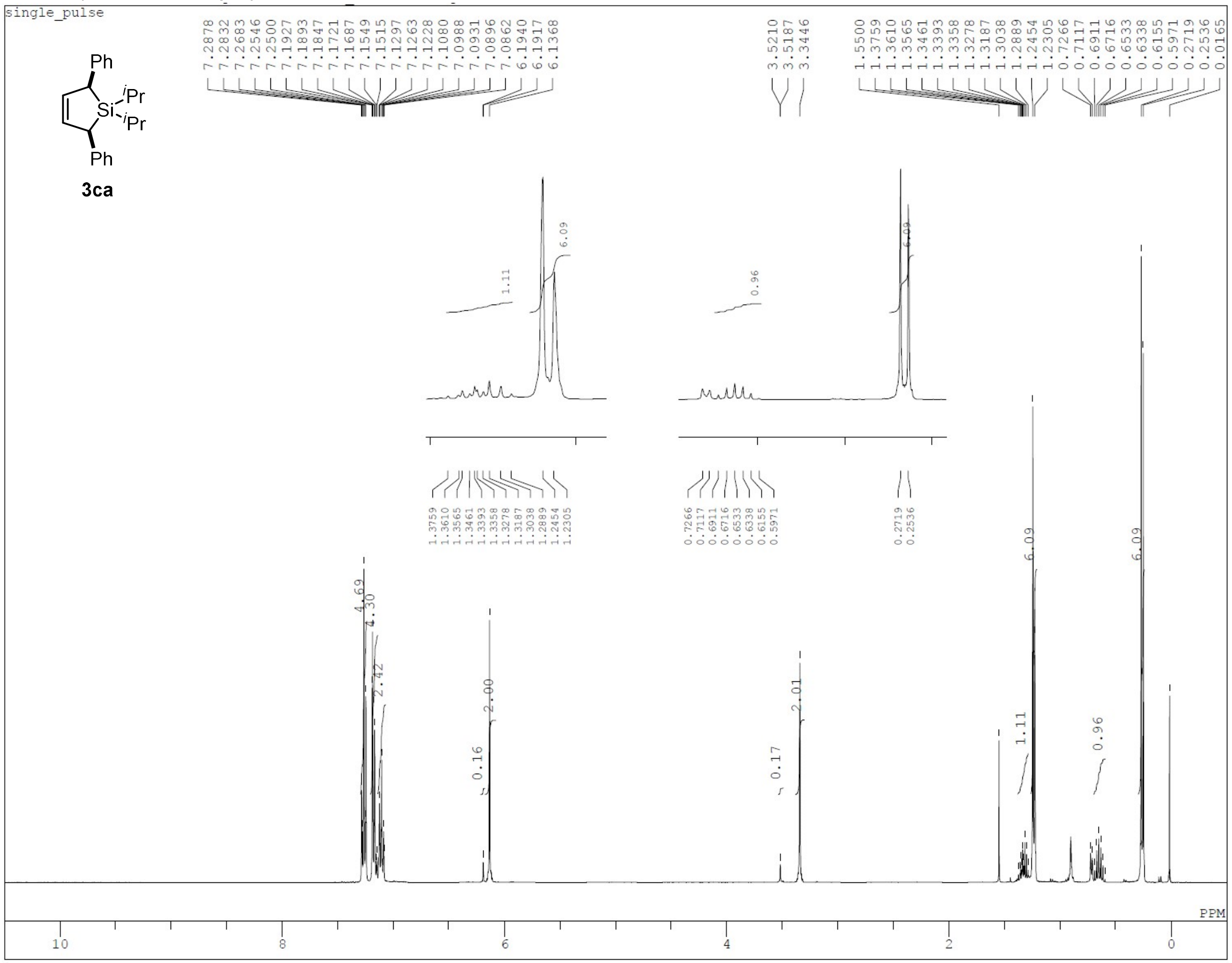

^{13}C NMR (100 MHz, $CDCl_3$, rt)

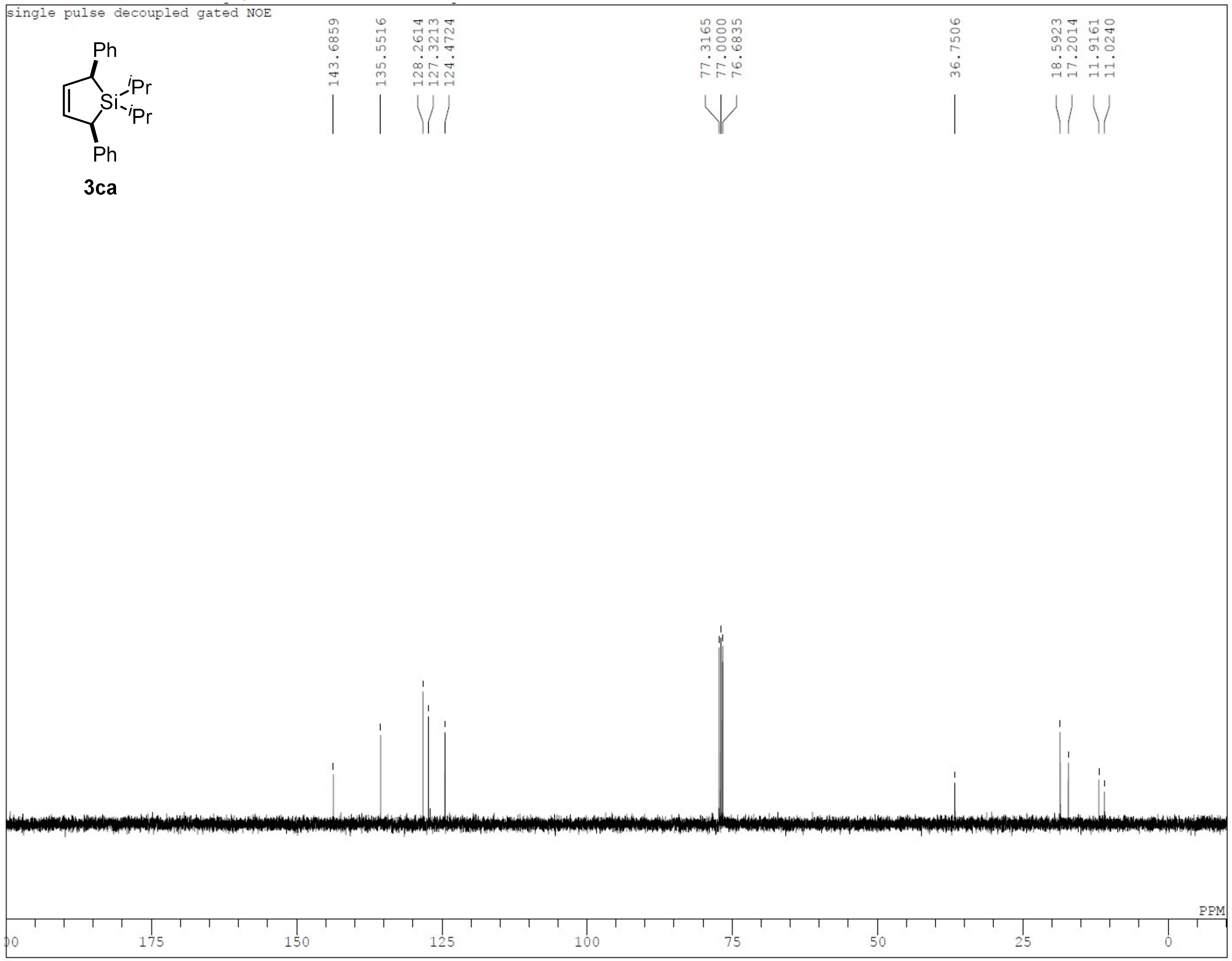

^{29}Si NMR (79 MHz, $CDCl_3$, rt)

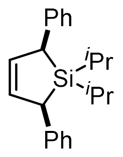

3ca

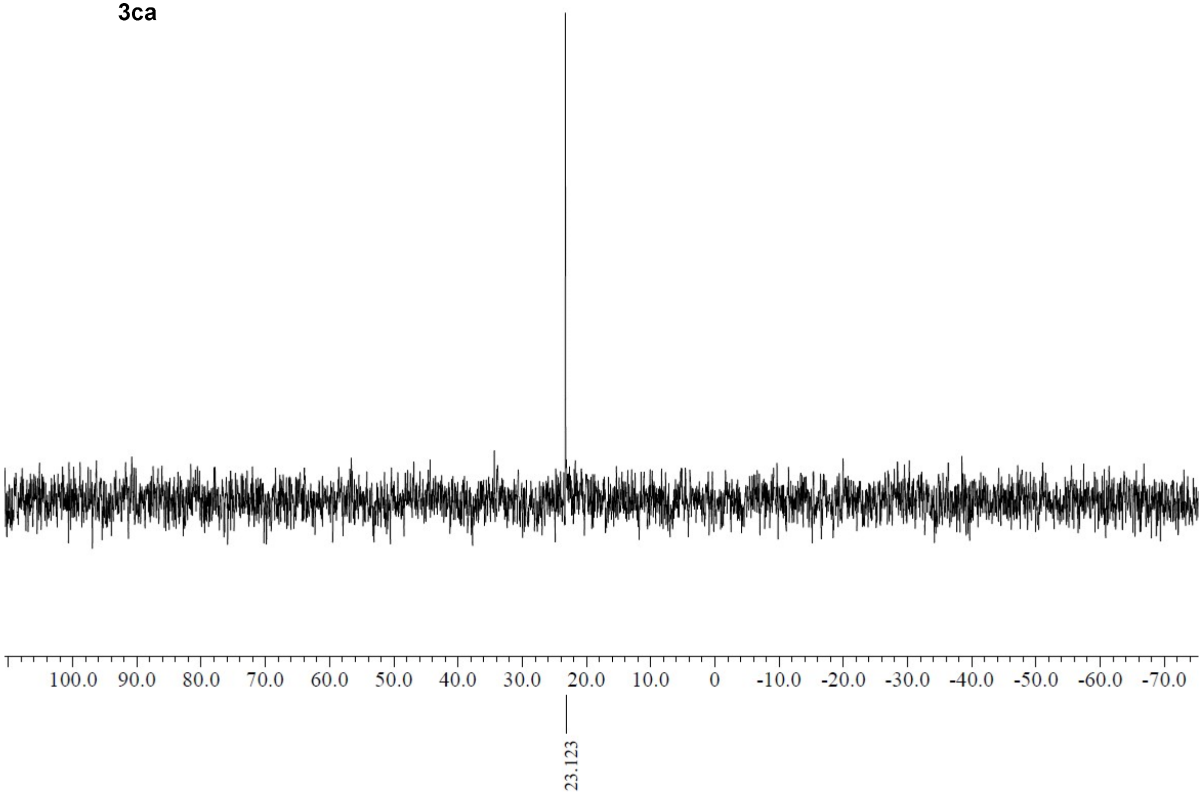

^1H NMR (400 MHz, $CDCl_3$, rt)

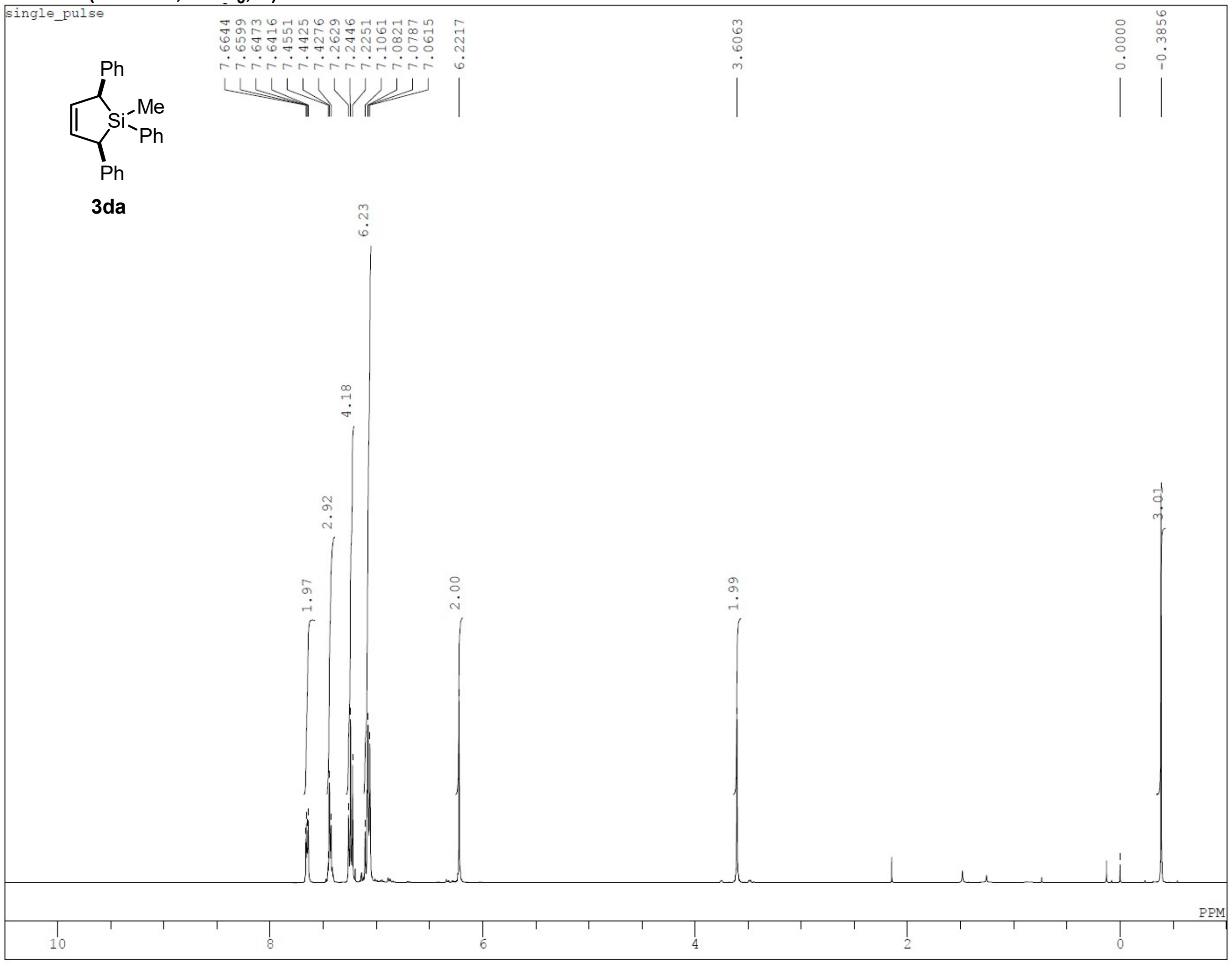

^{13}C NMR (100 MHz, $CDCl_3$, rt)

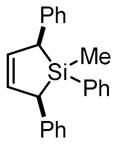

3da

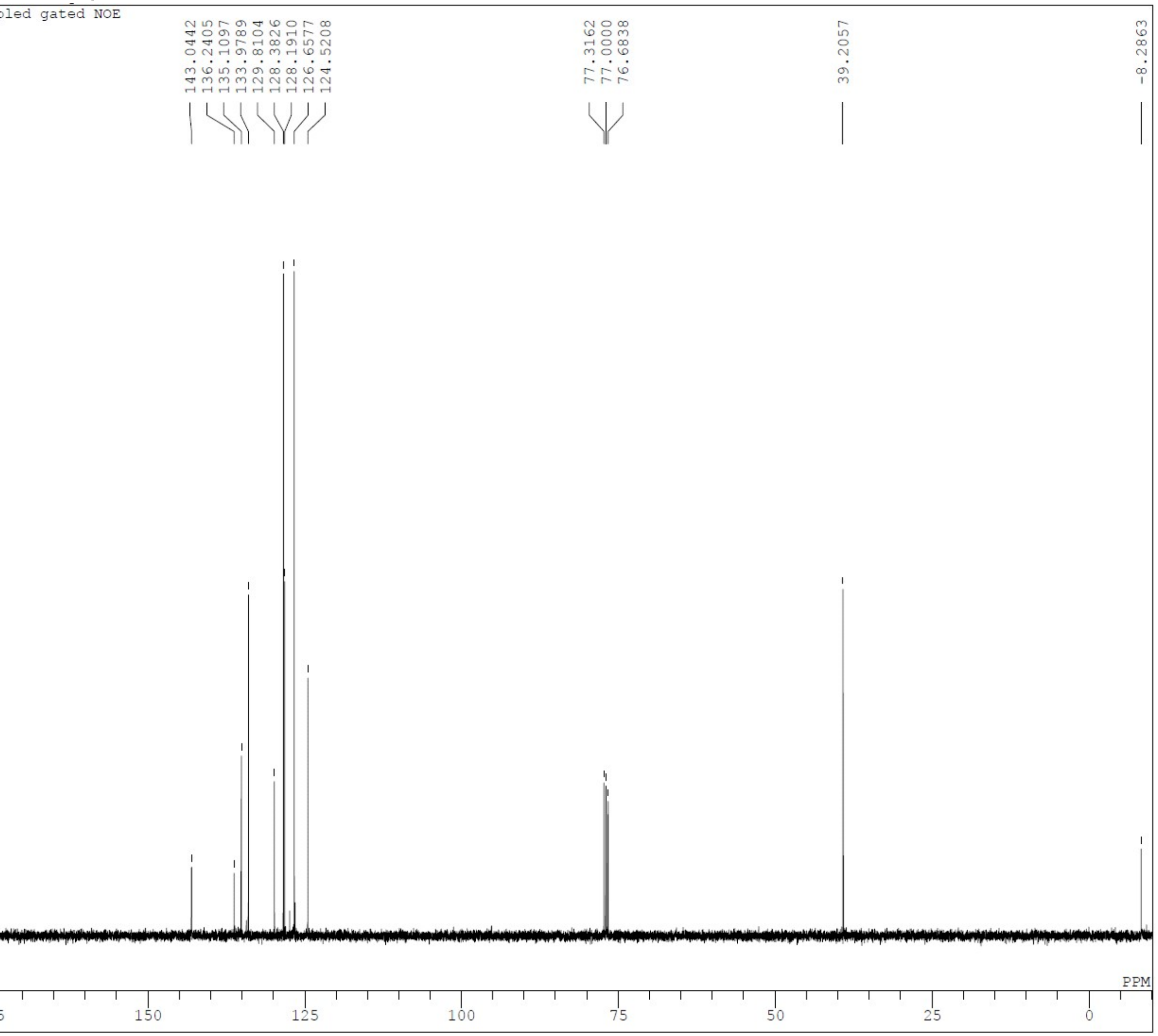

^{29}Si NMR (79 MHz, $CDCl_3$, rt)

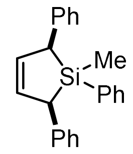

3da

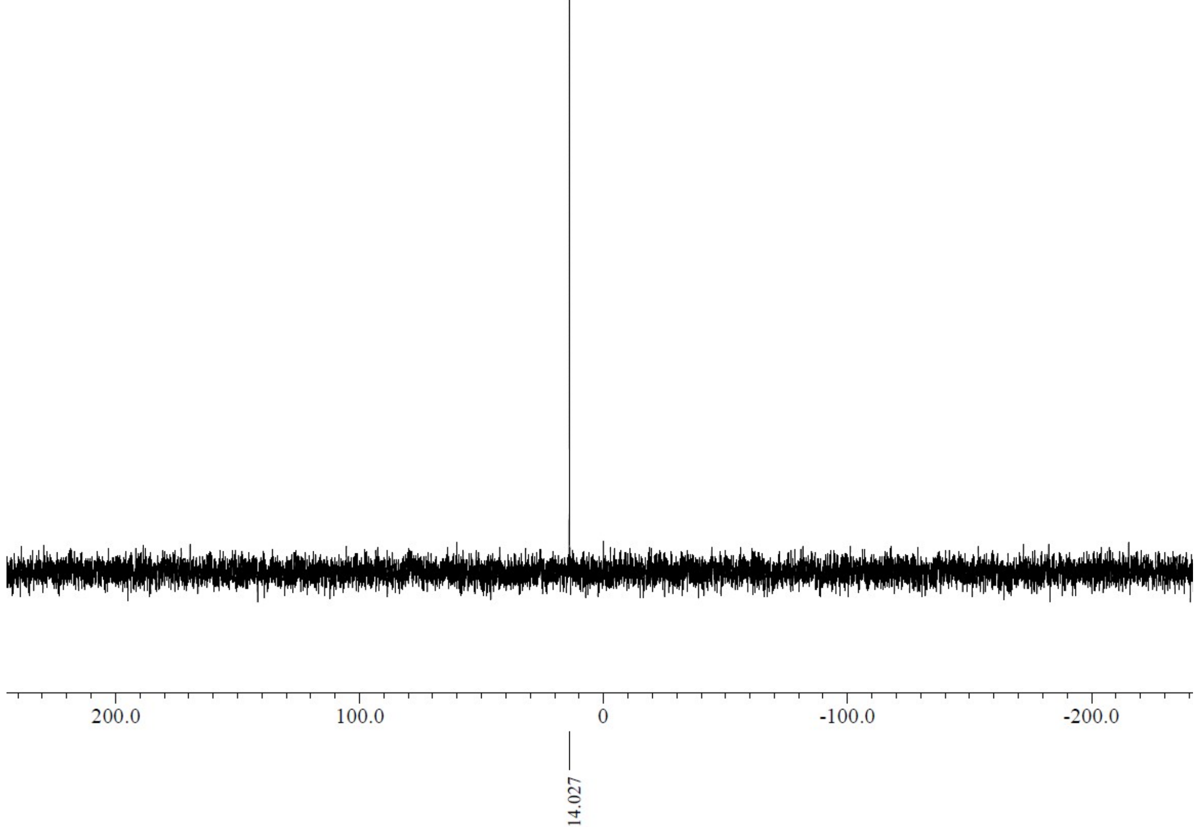

3da; NOE

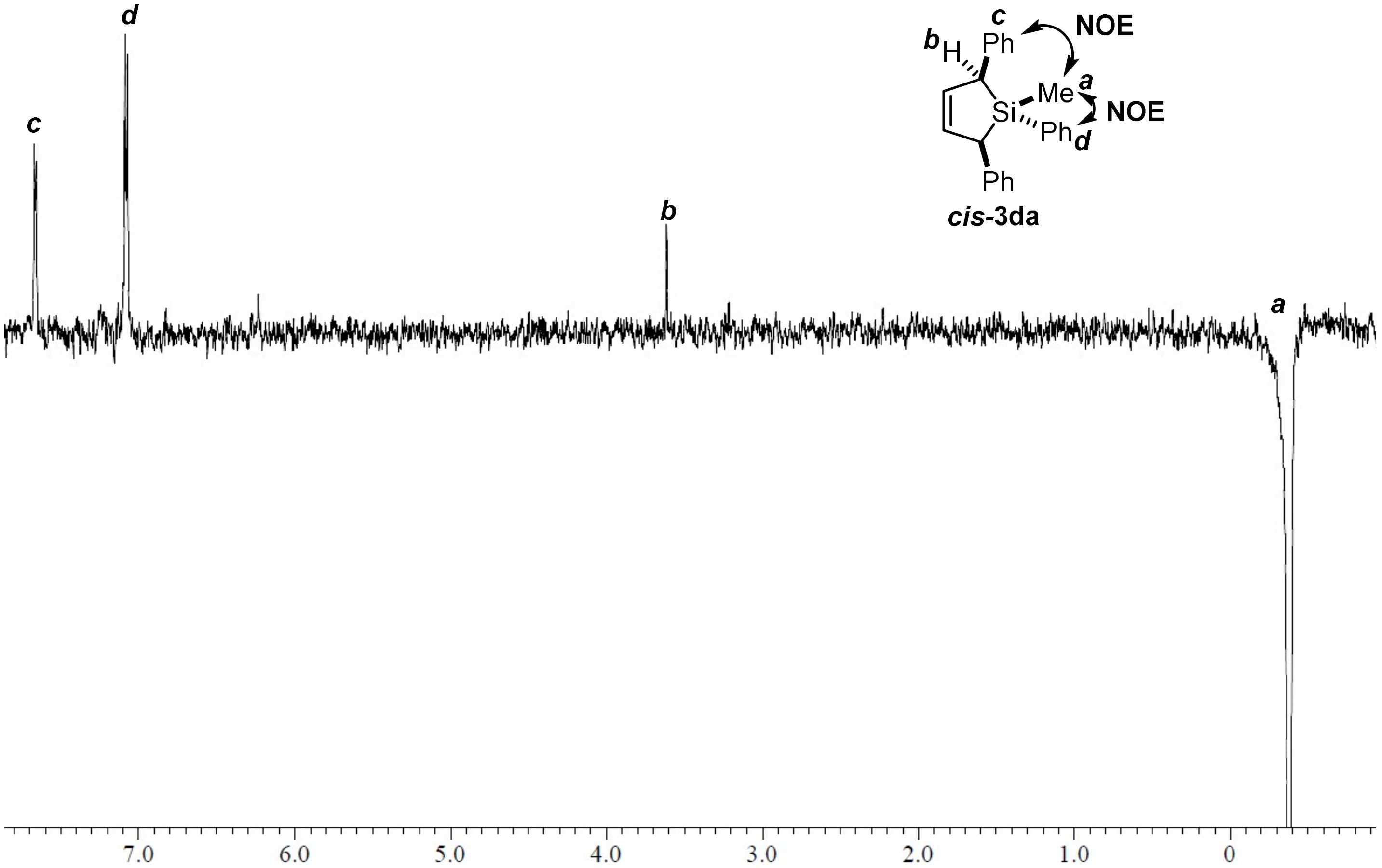

^{1}H NMR (400 MHz, $CDCl_3$, rt)

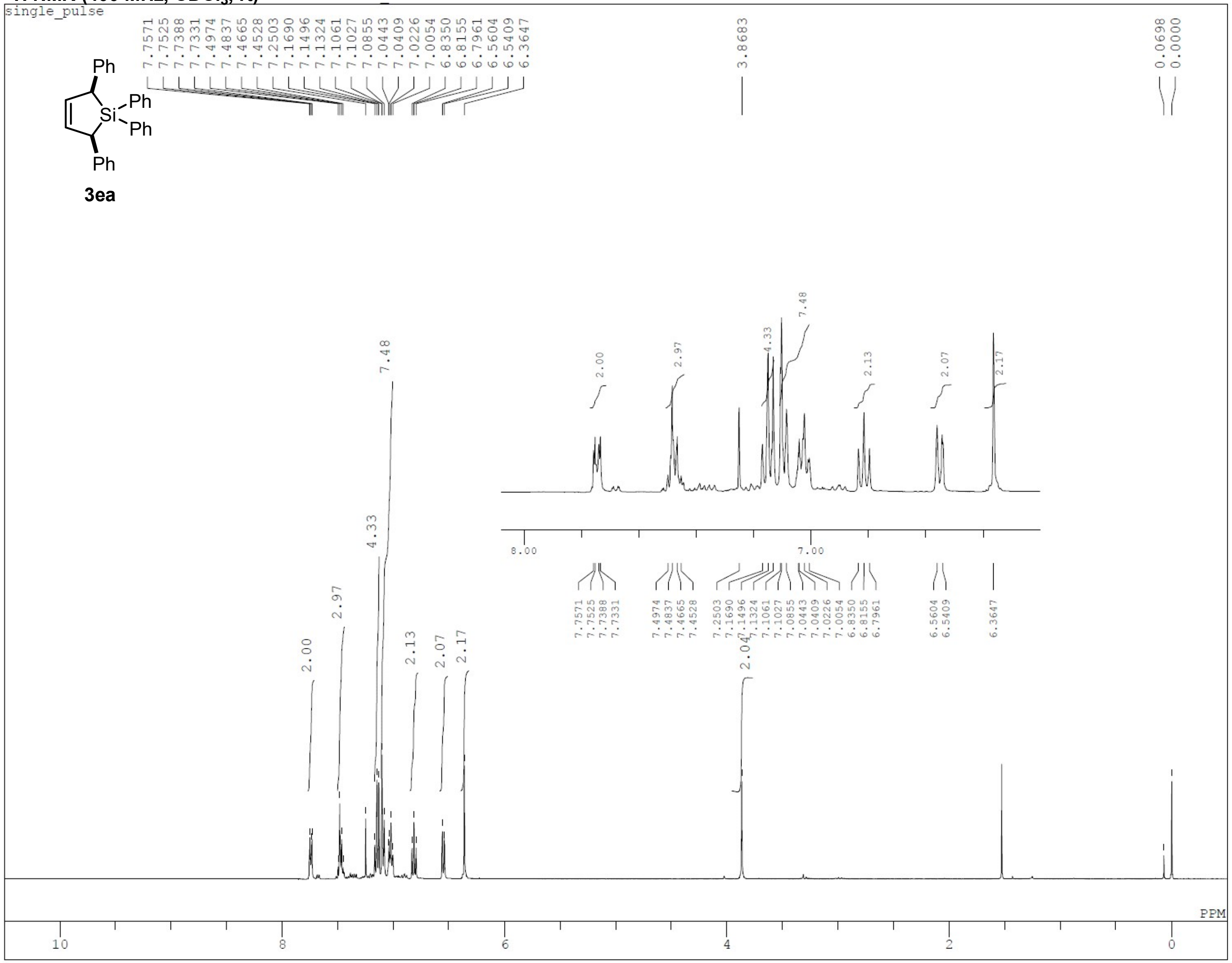

^{13}C NMR (100 MHz, $CDCl_3$, rt)

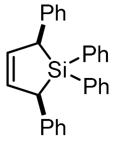

3ea

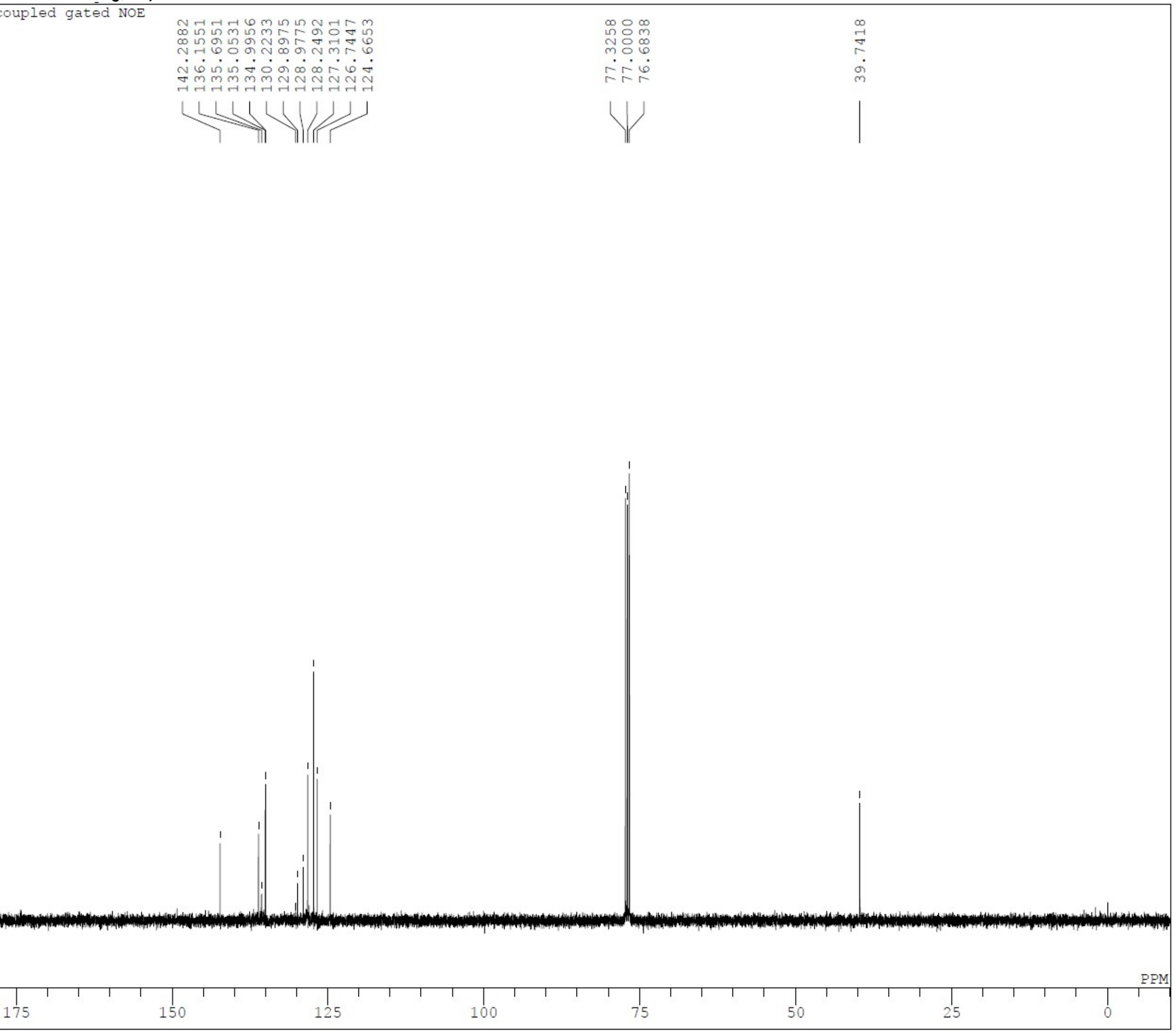

^{29}Si NMR (79 MHz, $CDCl_3$, rt)

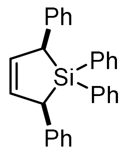

3ea

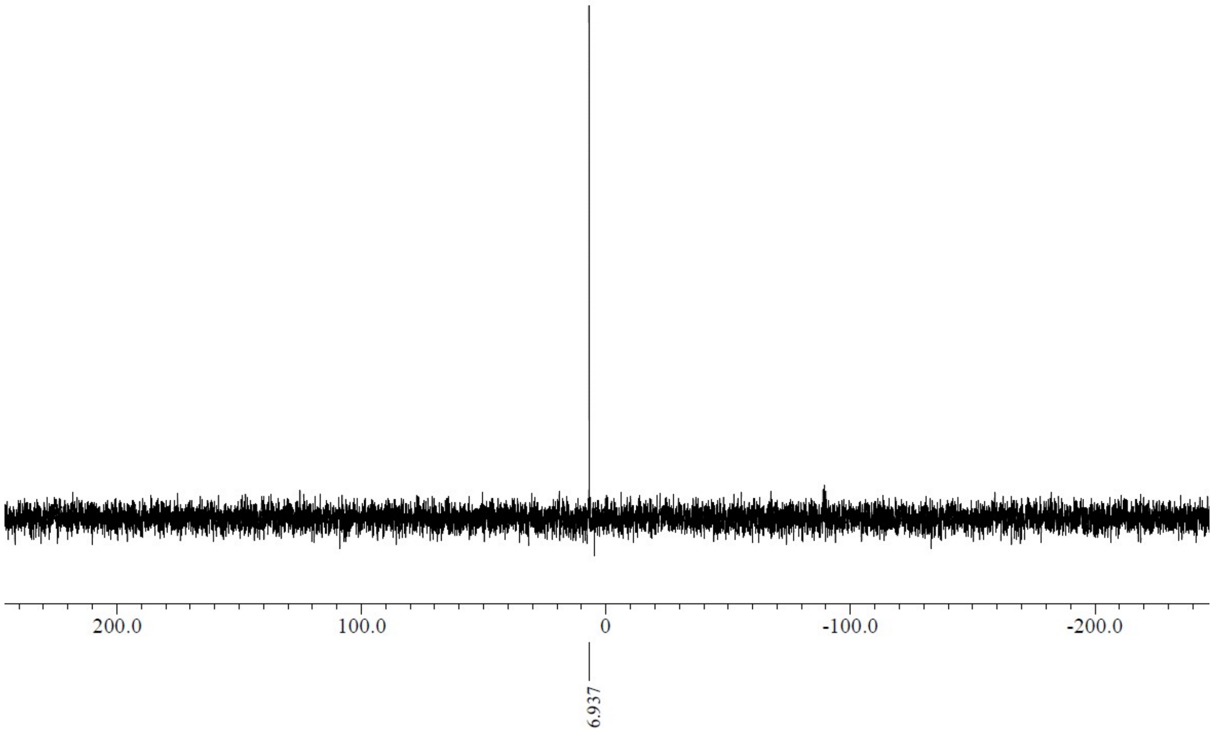

^{1}H NMR (400 MHz, $CDCl_3$, rt)

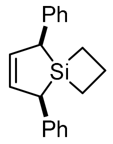

3fa

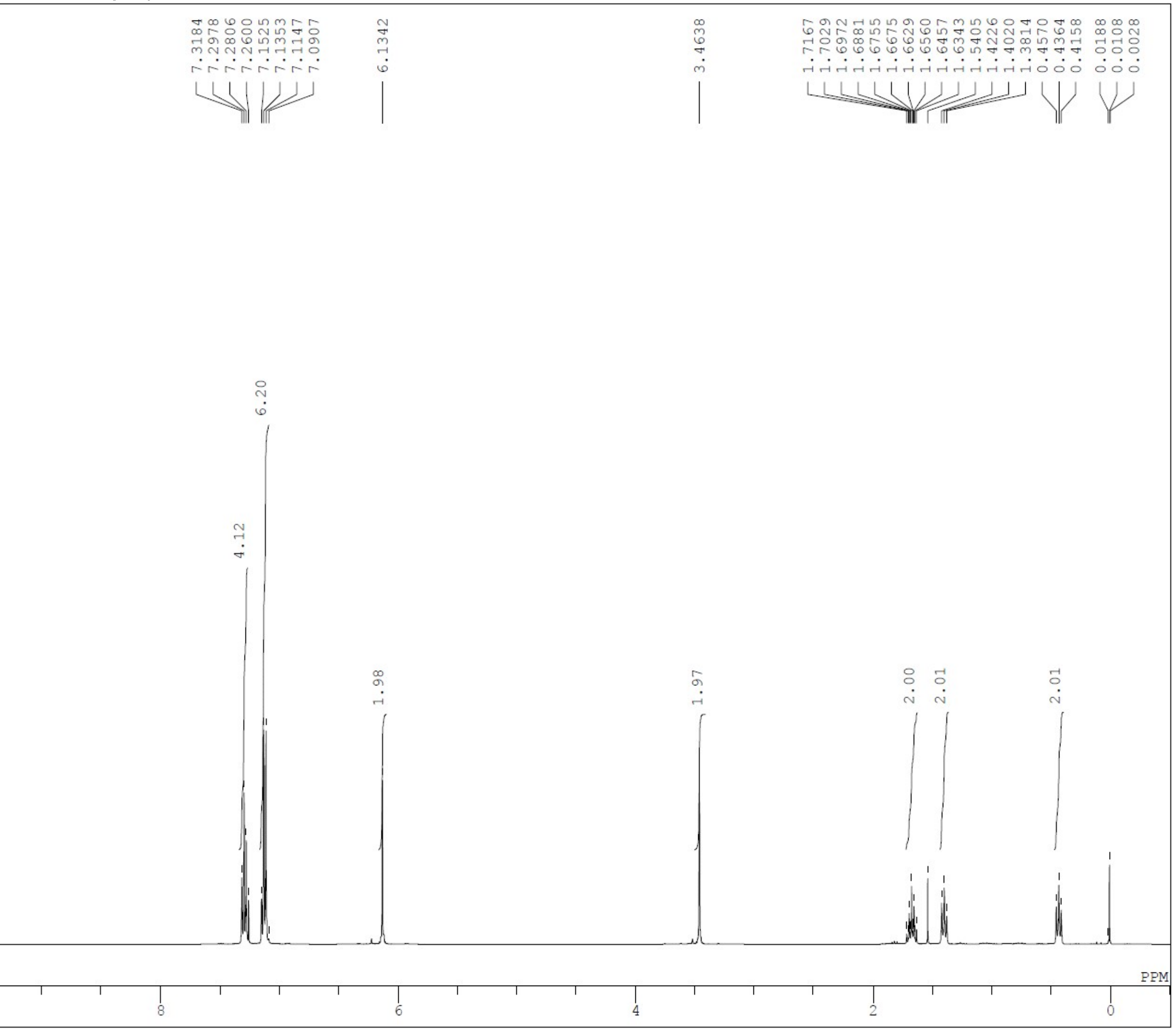

^{13}C NMR (100 MHz, $CDCl_3$, rt)

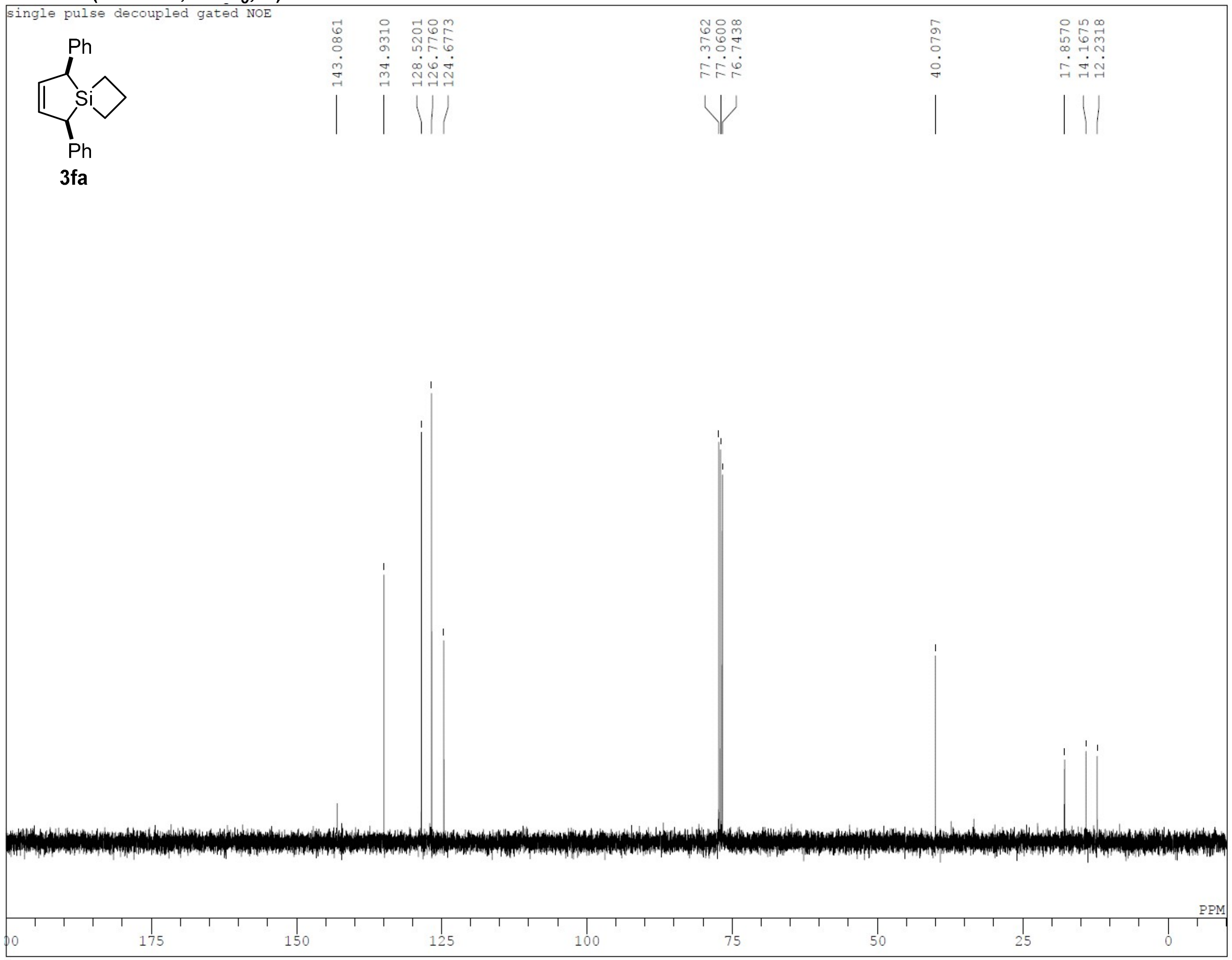

^{29}Si NMR (79 MHz, $CDCl_3$, rt)

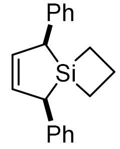

3fa

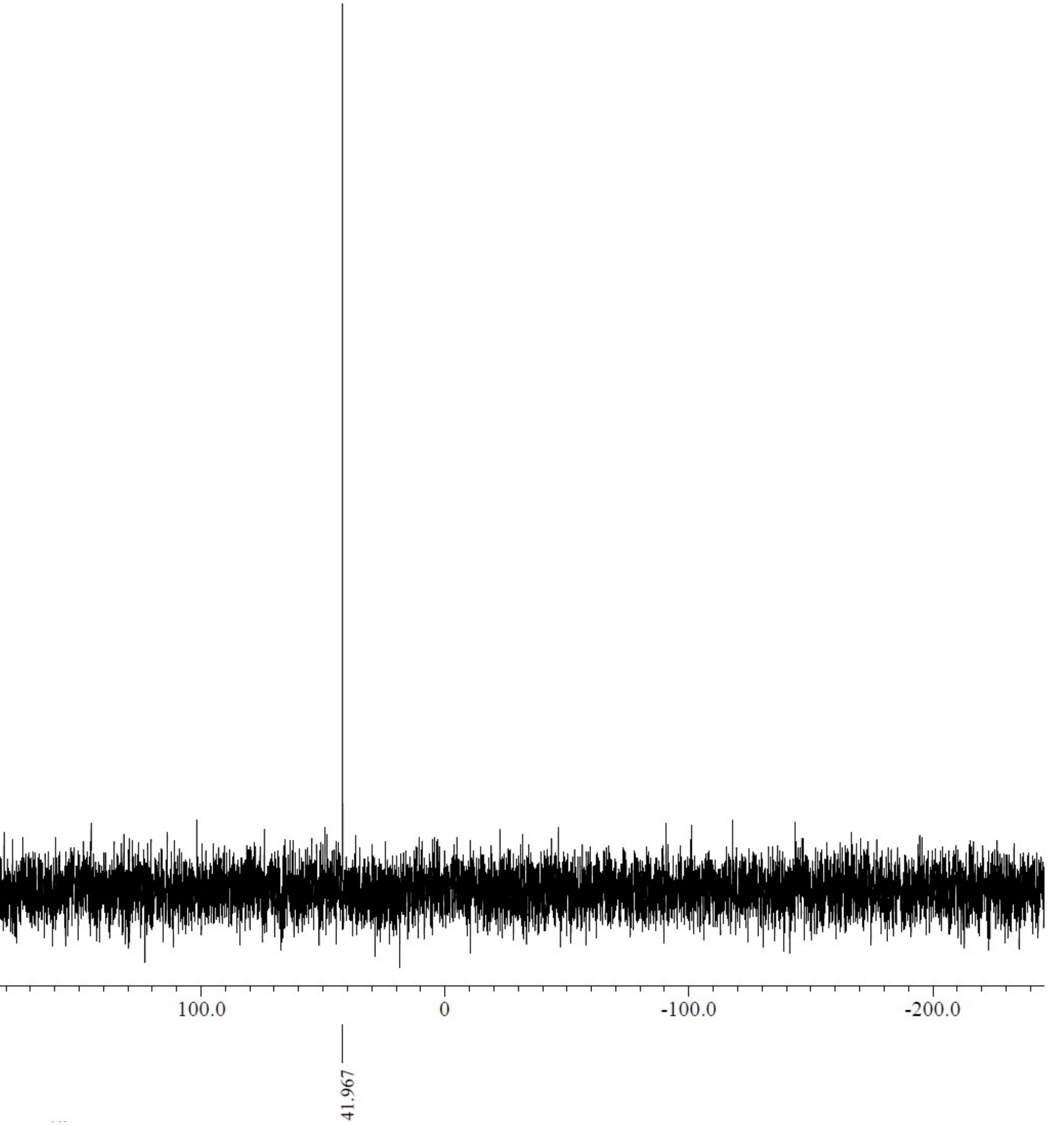

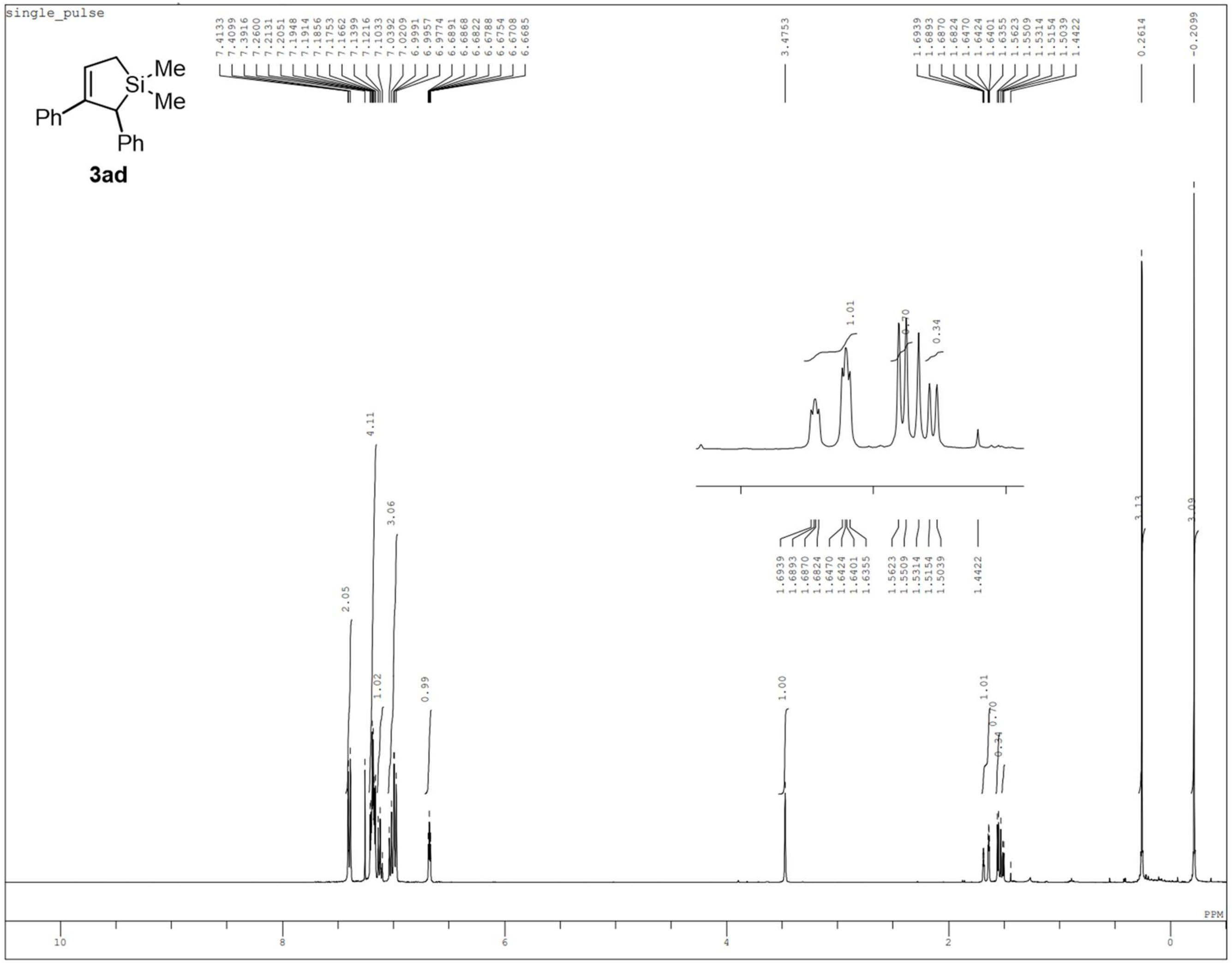

single_pulse
Me
Si
Me
Ph
Ph
3ad
7.4133
7.4099
7.3916
7.2600
7.2131
7.2051
7.1948
7.1914
7.1856
7.1753
7.1662
7.1399
7.1216
7.1033
7.0392
7.0209
6.9991
6.9957
6.9774
6.6891
6.6868
6.6822
6.6788
6.6754
6.6708
6.6685
3.4753
1.6939
1.6893
1.6870
1.6824
1.6470
1.6424
1.6401
1.6355
1.5623
1.5509
1.5314
1.5154
1.5039
1.4422
0.2614
-0.2099
2.05
4.11
1.02
3.06
0.99
1.00
1.01
0.70
0.34
3.13
3.09
PPM
10
8
6
4
2
0

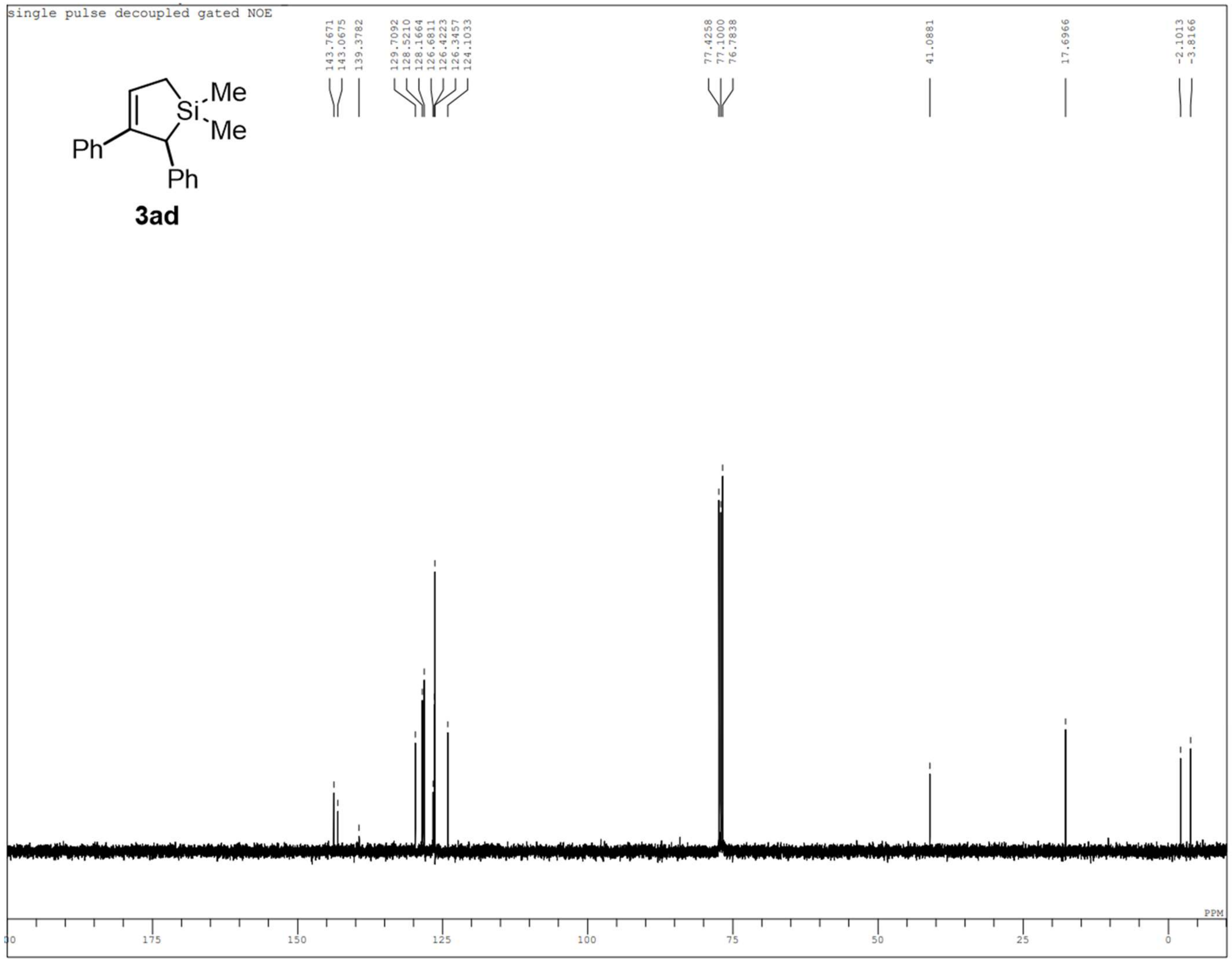
single pulse decoupled gated NOE
143.7671
143.0675
139.3782
129.7092
128.5210
128.1664
126.6811
126.4223
126.3457
124.1033
77.4258
77.1000
76.7838
41.0881
17.6966
-2.1013
-3.8166
Me
Si
Me
Ph
Ph
3ad
PPM
175
150
125
100
75
50
25
0

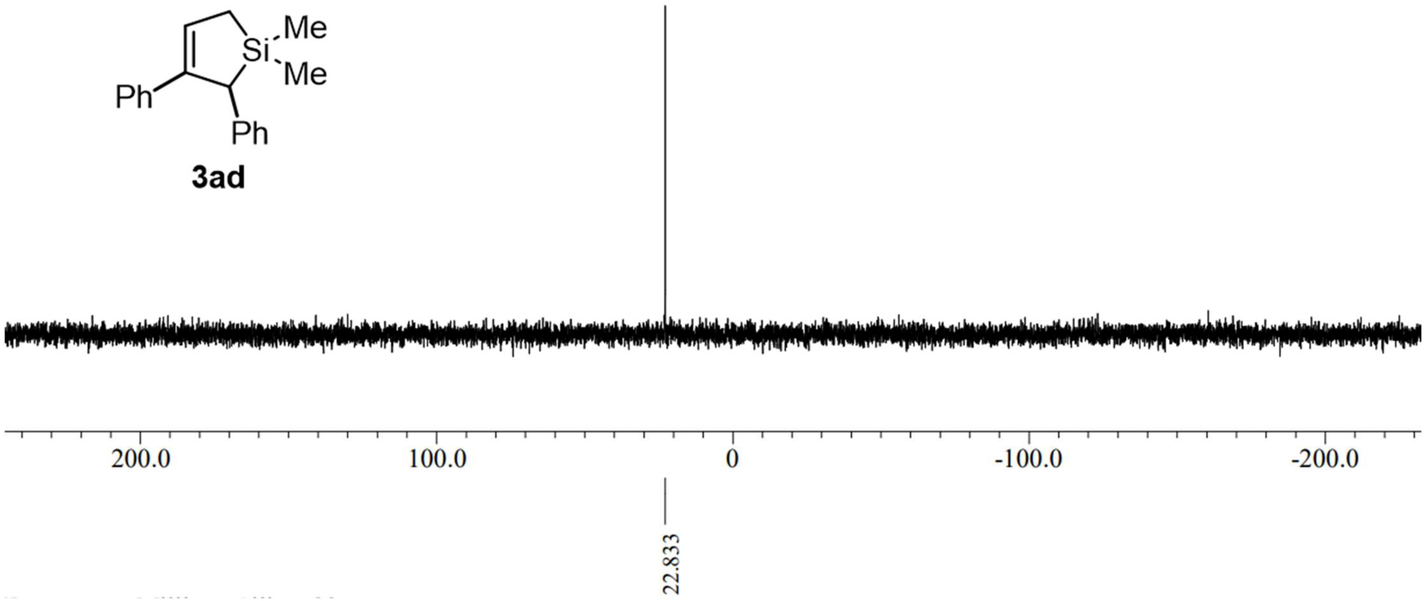
Me
Si
Me
Ph
Ph
3ad
200.0
100.0
0
-100.0
-200.0
22.833

^{1}H NMR (400 MHz, $CDCl_3$, rt)

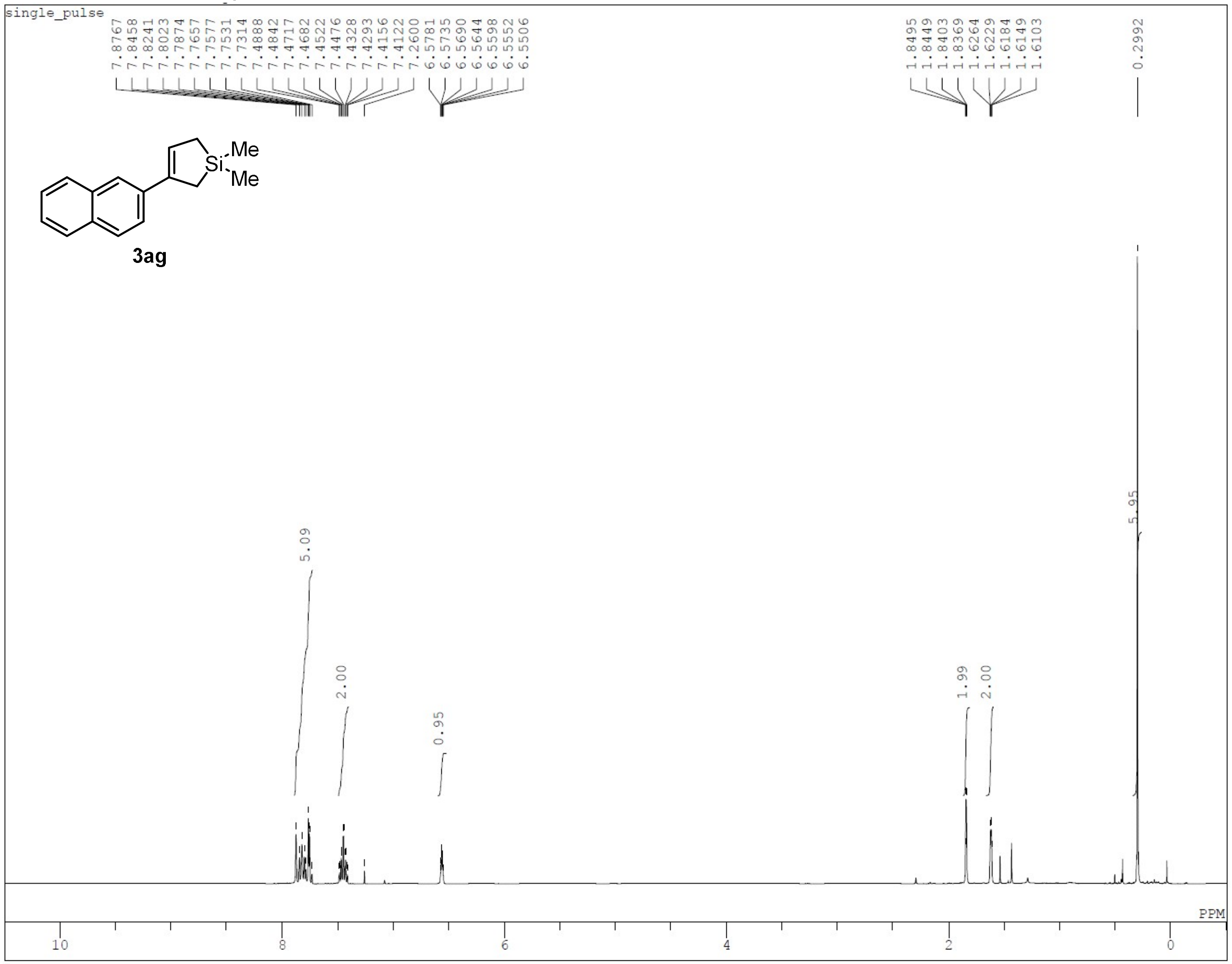

^{13}C NMR (100 MHz, $CDCl_3$, rt)

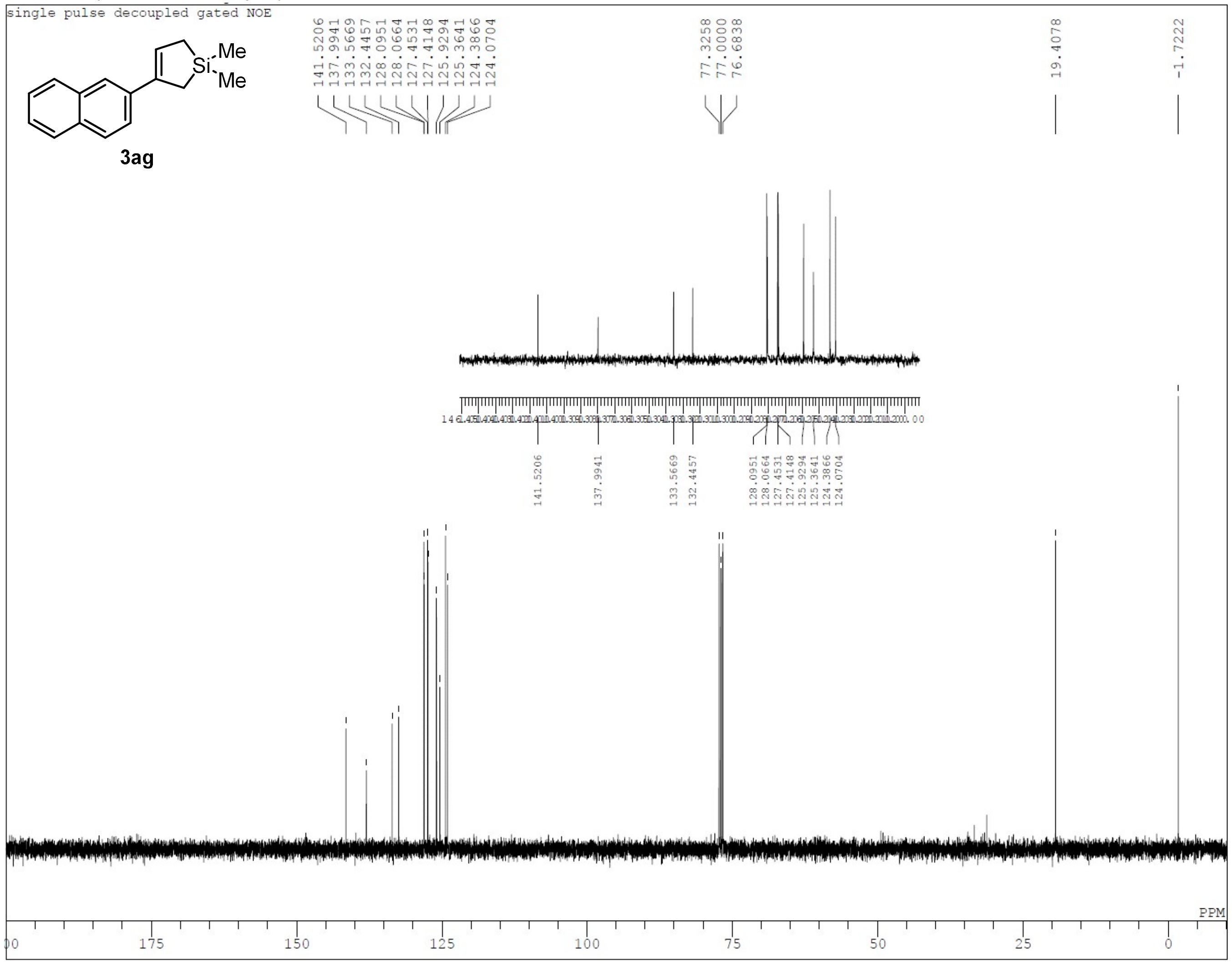

^{29}Si NMR (79 MHz, $CDCl_3$, rt)

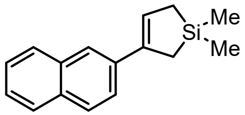

3ag

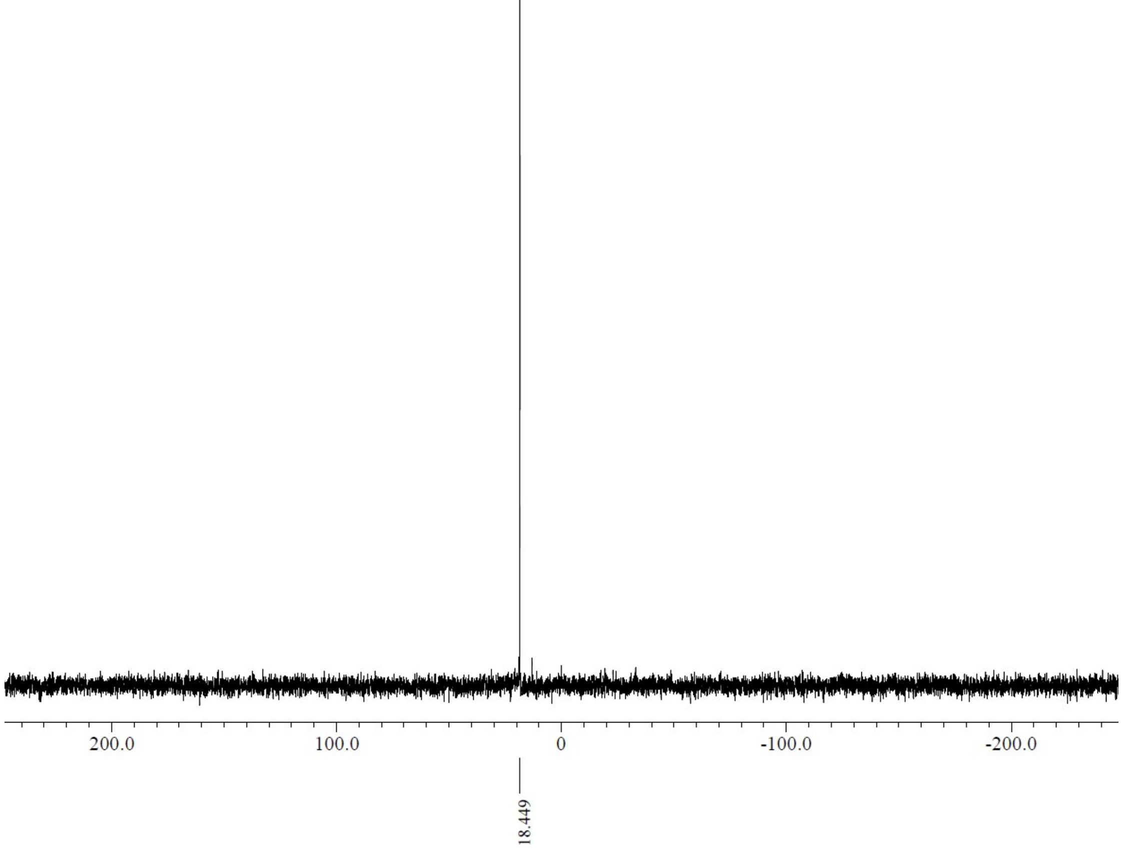

^1H NMR (400 MHz, $CDCl_3$, rt)

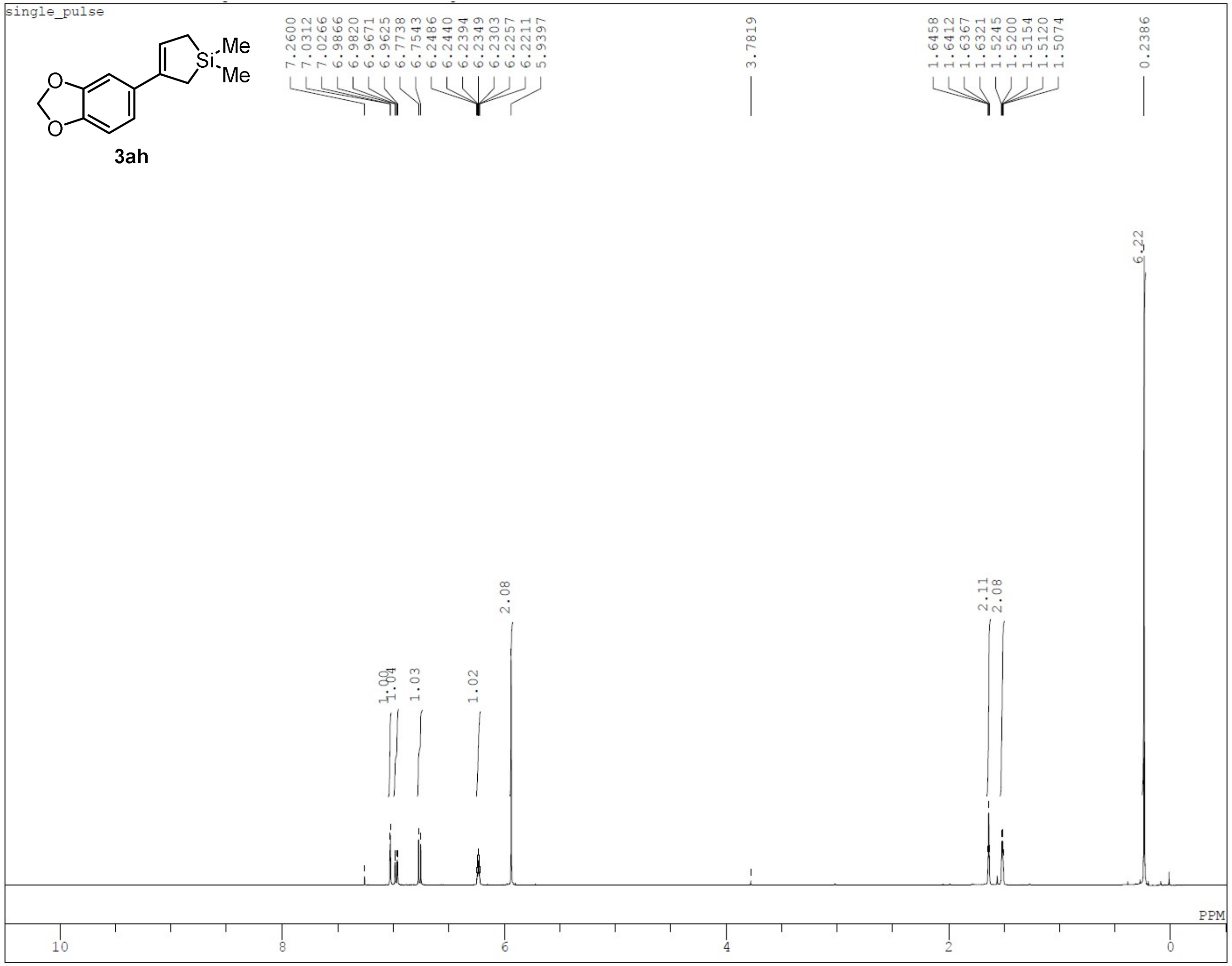

^{13}C NMR (100 MHz, $CDCl_3$, rt)

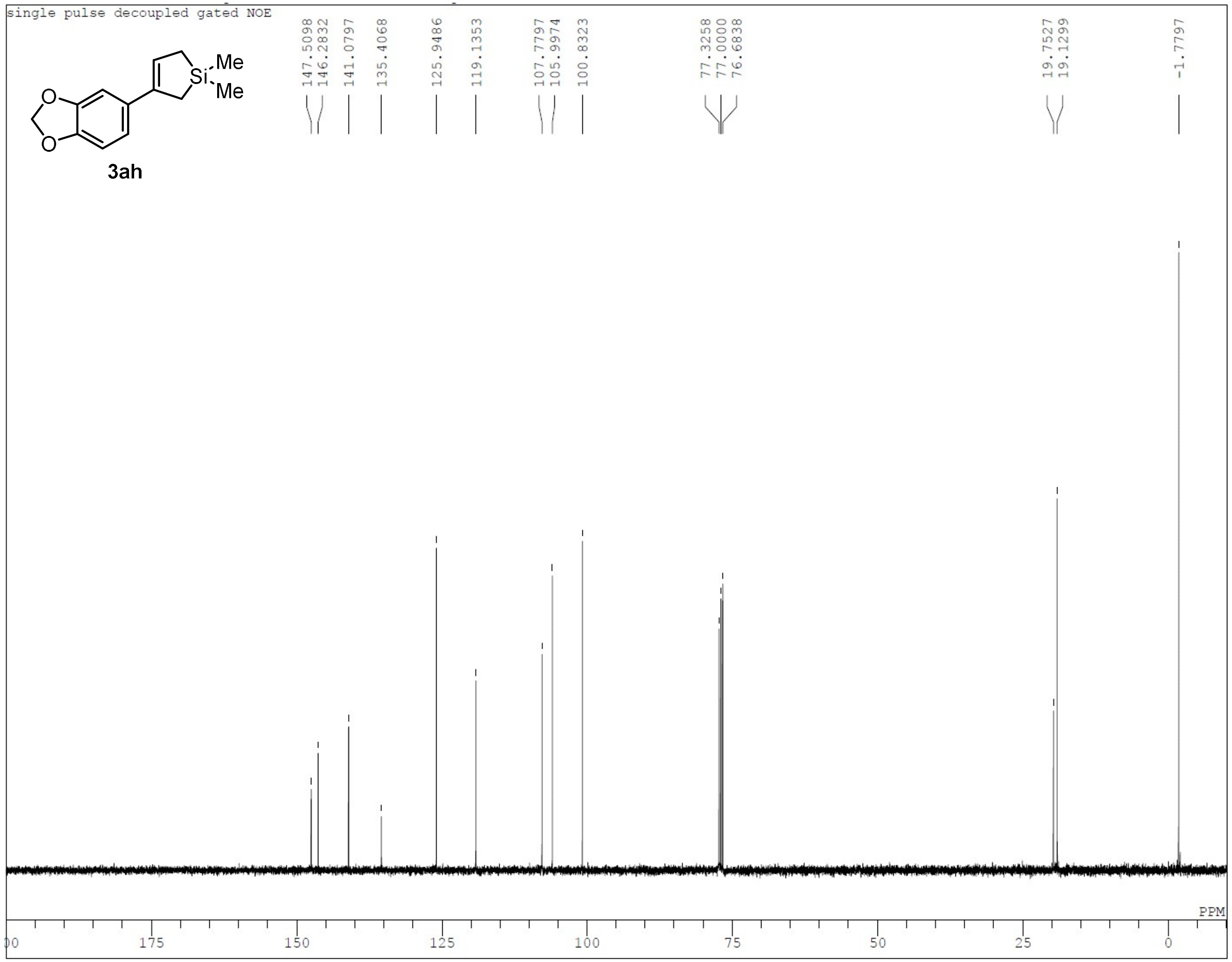

^{29}Si NMR (79 MHz, $CDCl_3$, rt)

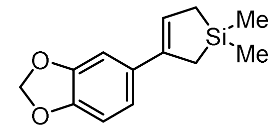

3ah

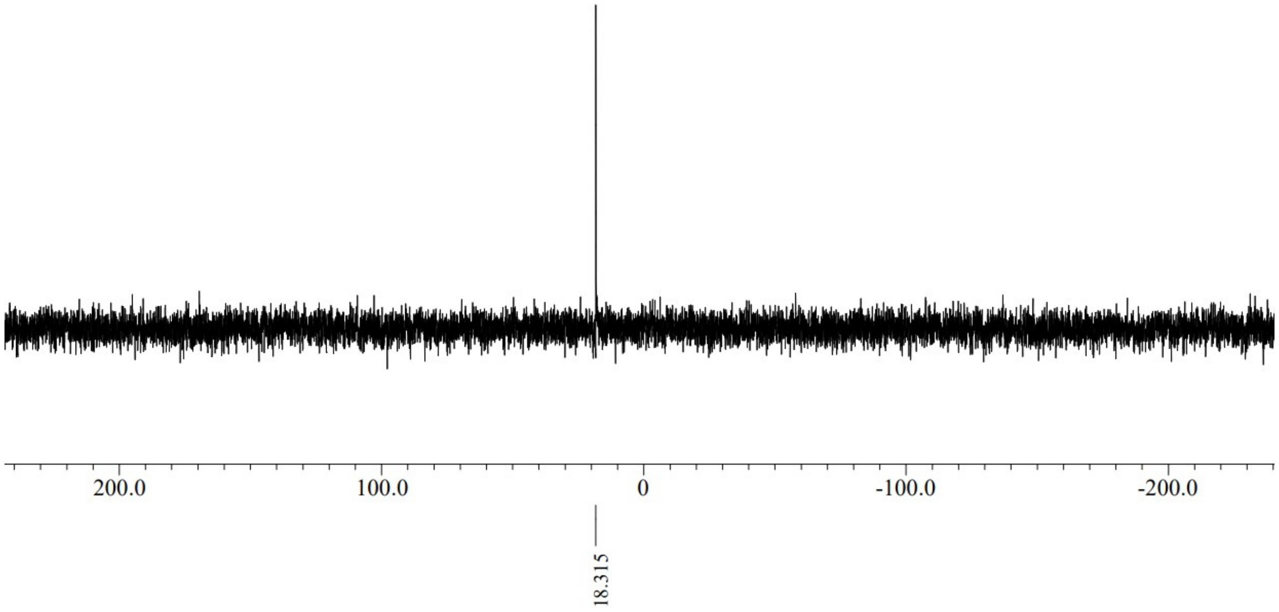

^{1}H NMR (400 MHz, $CDCl_3$, rt)

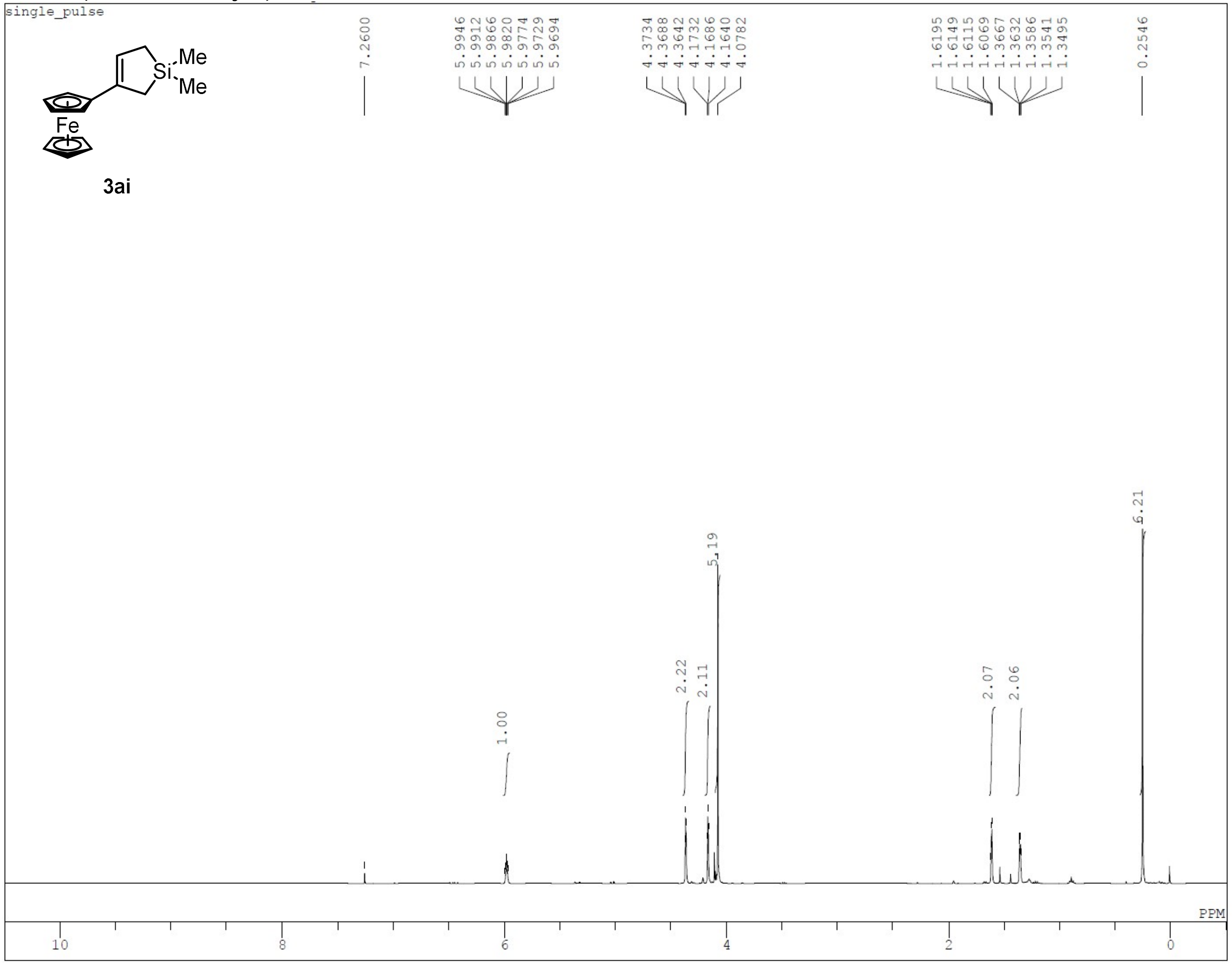

3ai

^{13}C NMR (100 MHz, $CDCl_3$, rt)

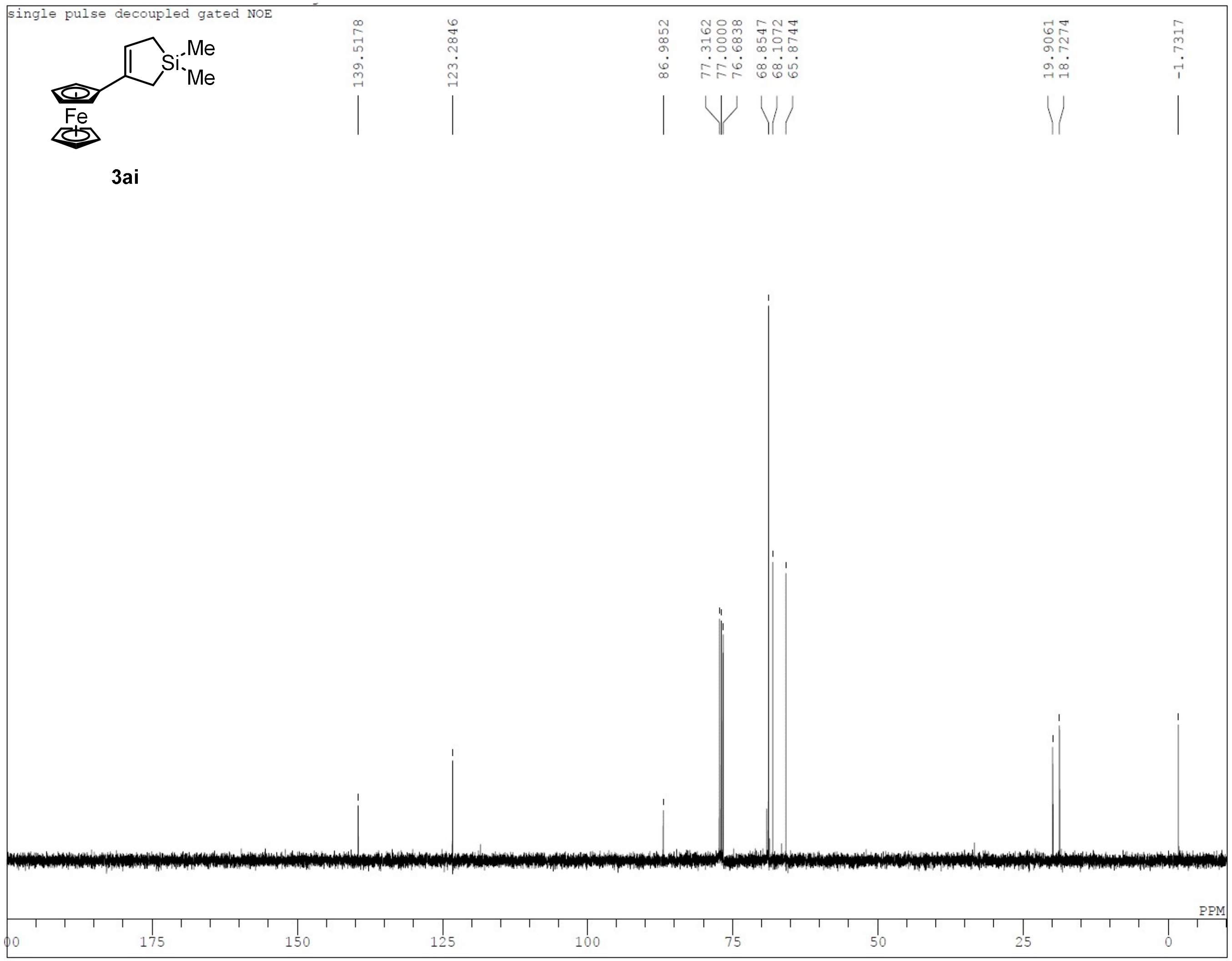

^{29}Si NMR (79 MHz, $CDCl_3$, rt)

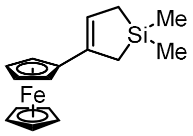

3ai

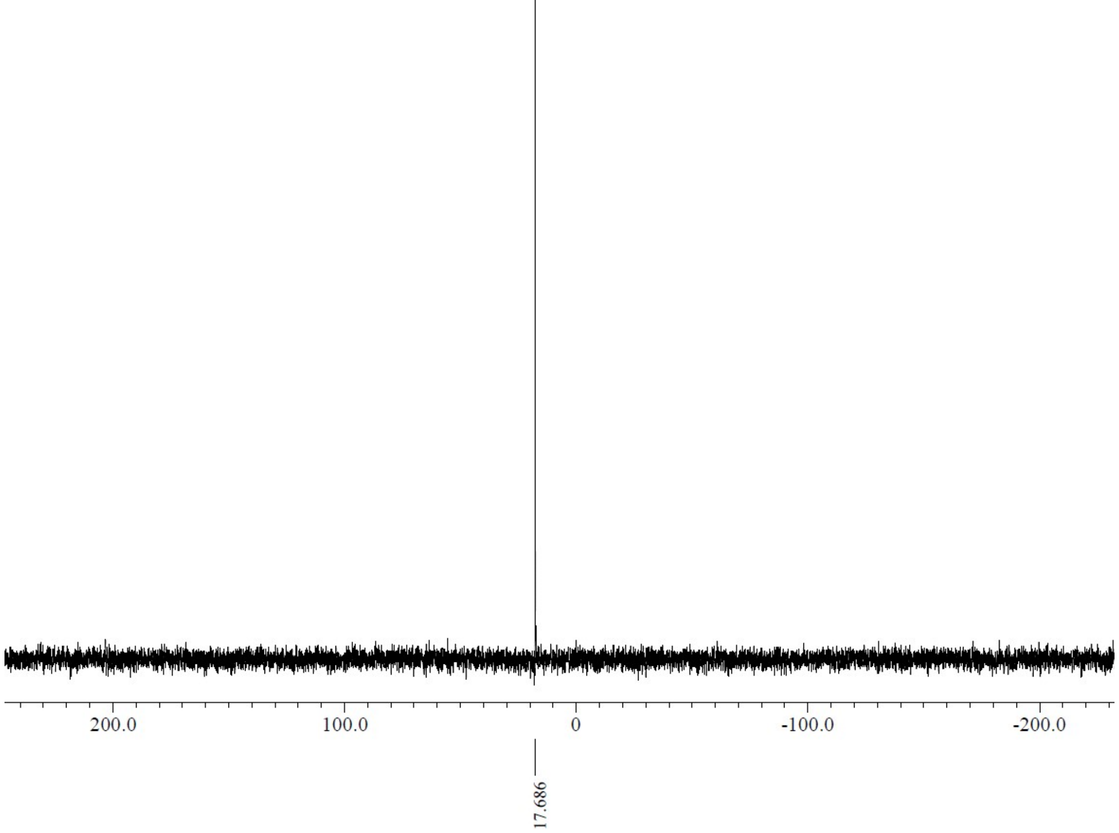

^{1}H NMR (400 MHz, $CDCl_3$, rt)

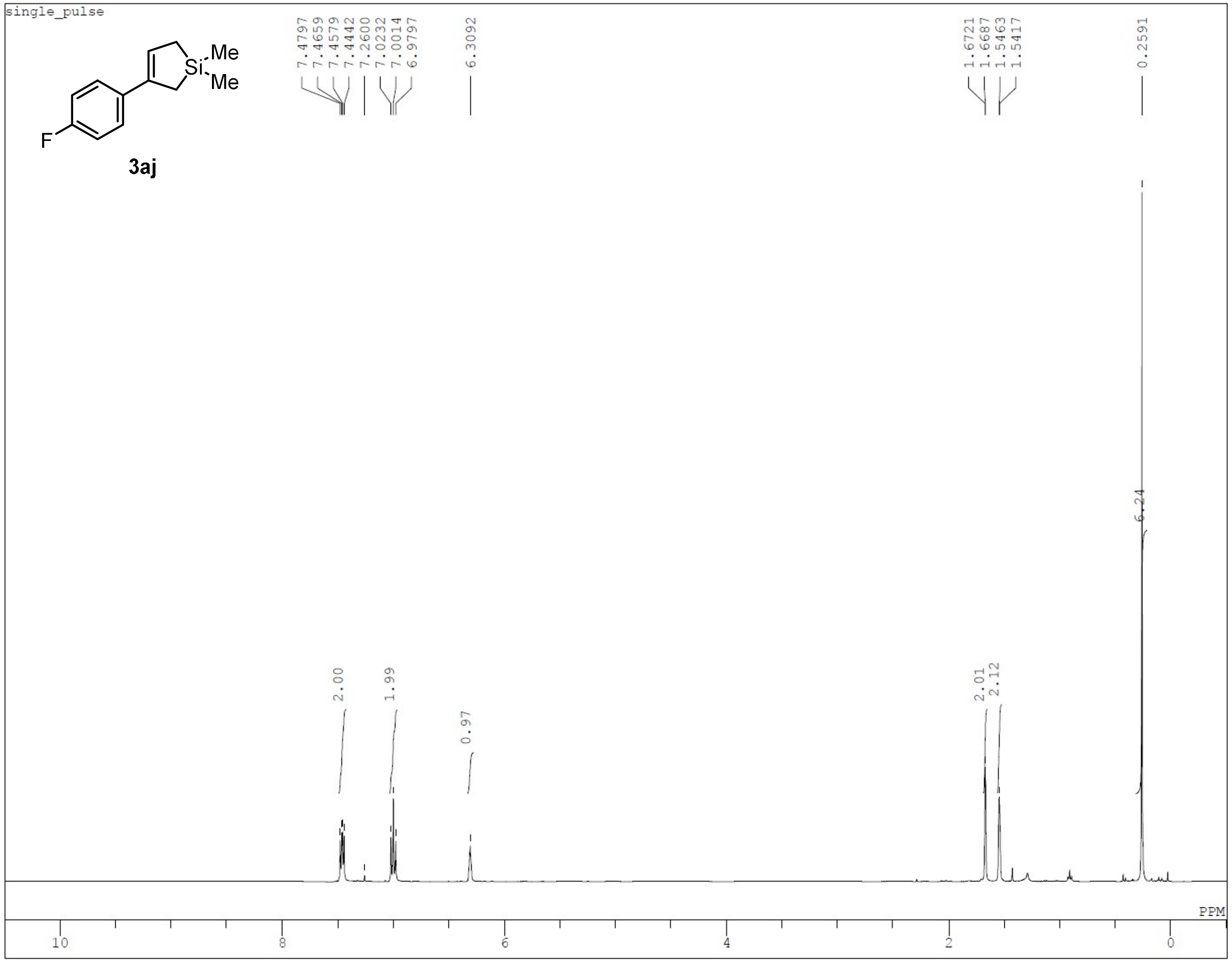

3aj

^{13}C NMR (100 MHz, $CDCl_3$, rt)

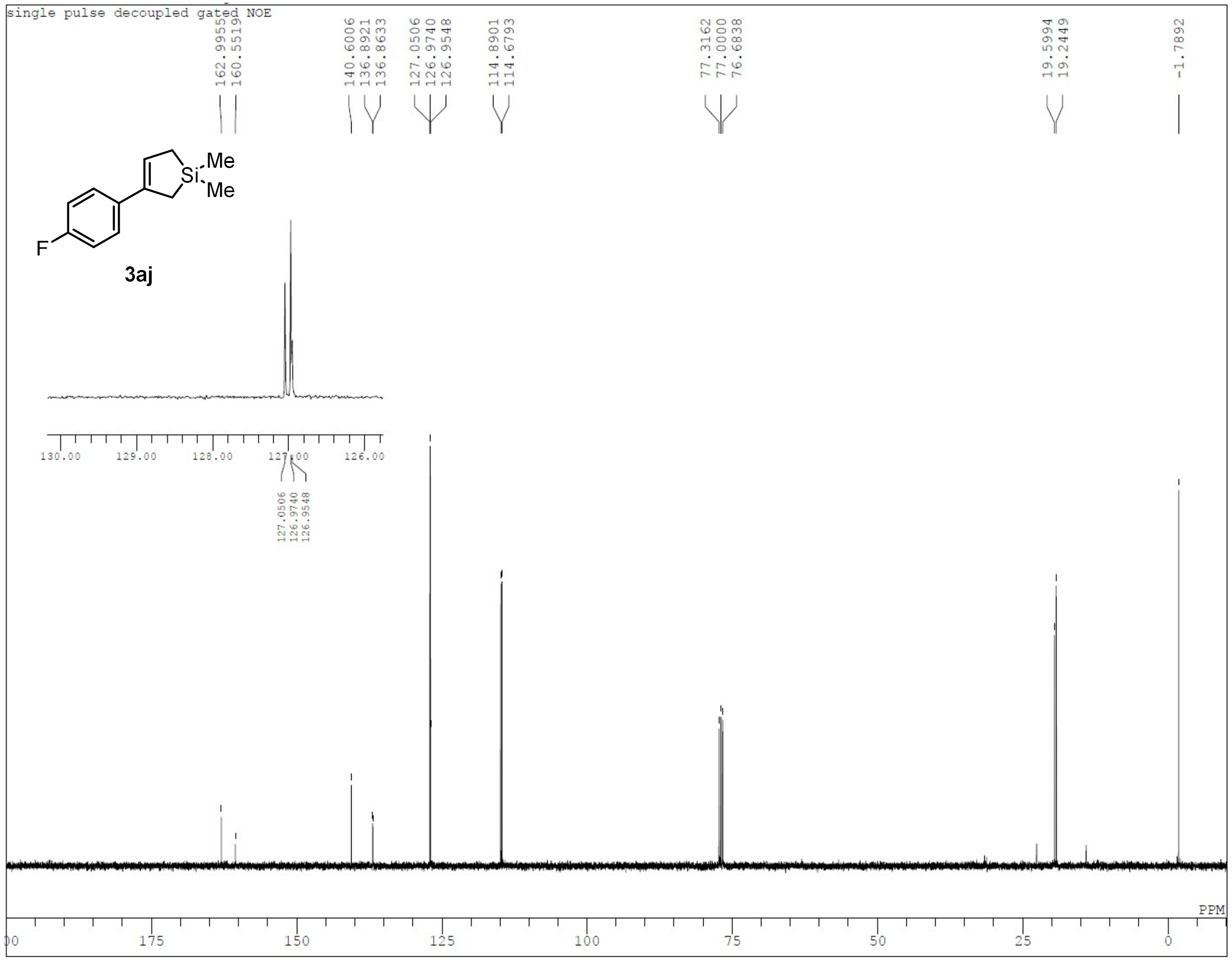

^{29}Si NMR (79 MHz, $CDCl_3$, rt)

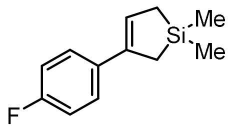

3aj

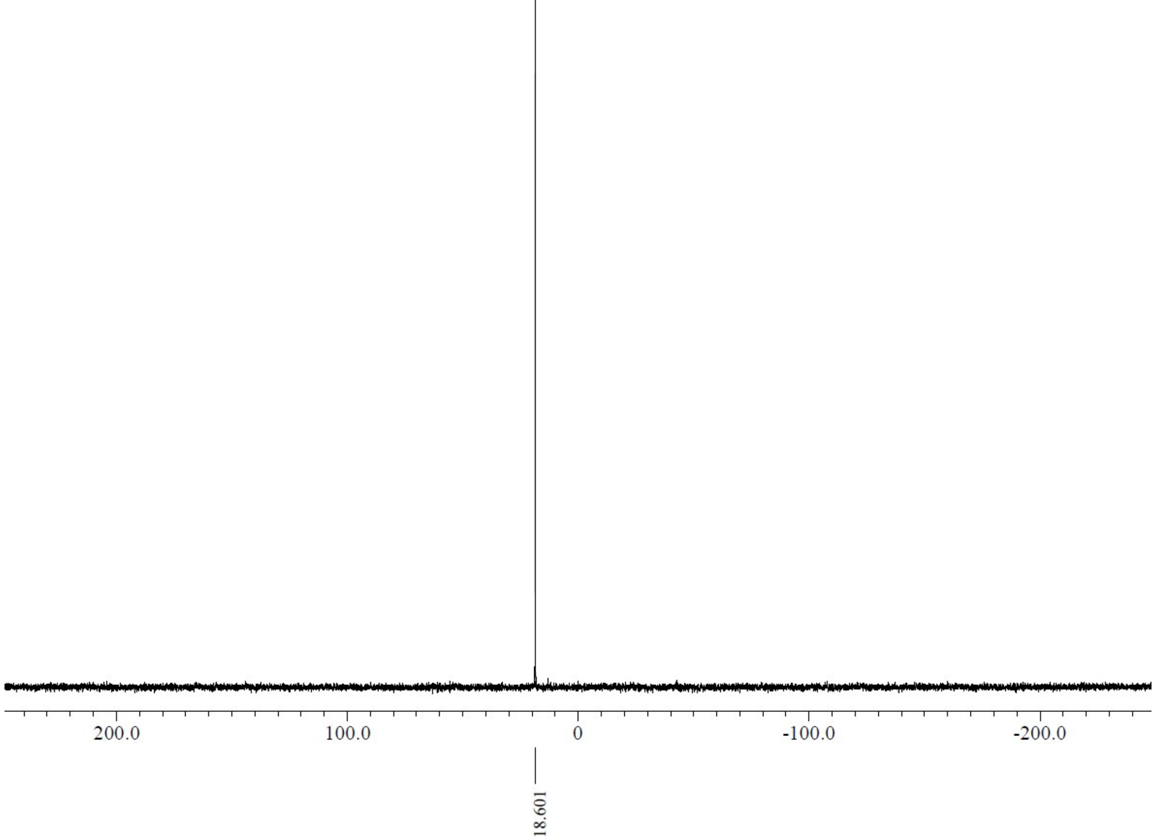

^{1}H NMR (400 MHz, $CDCl_3$, rt)

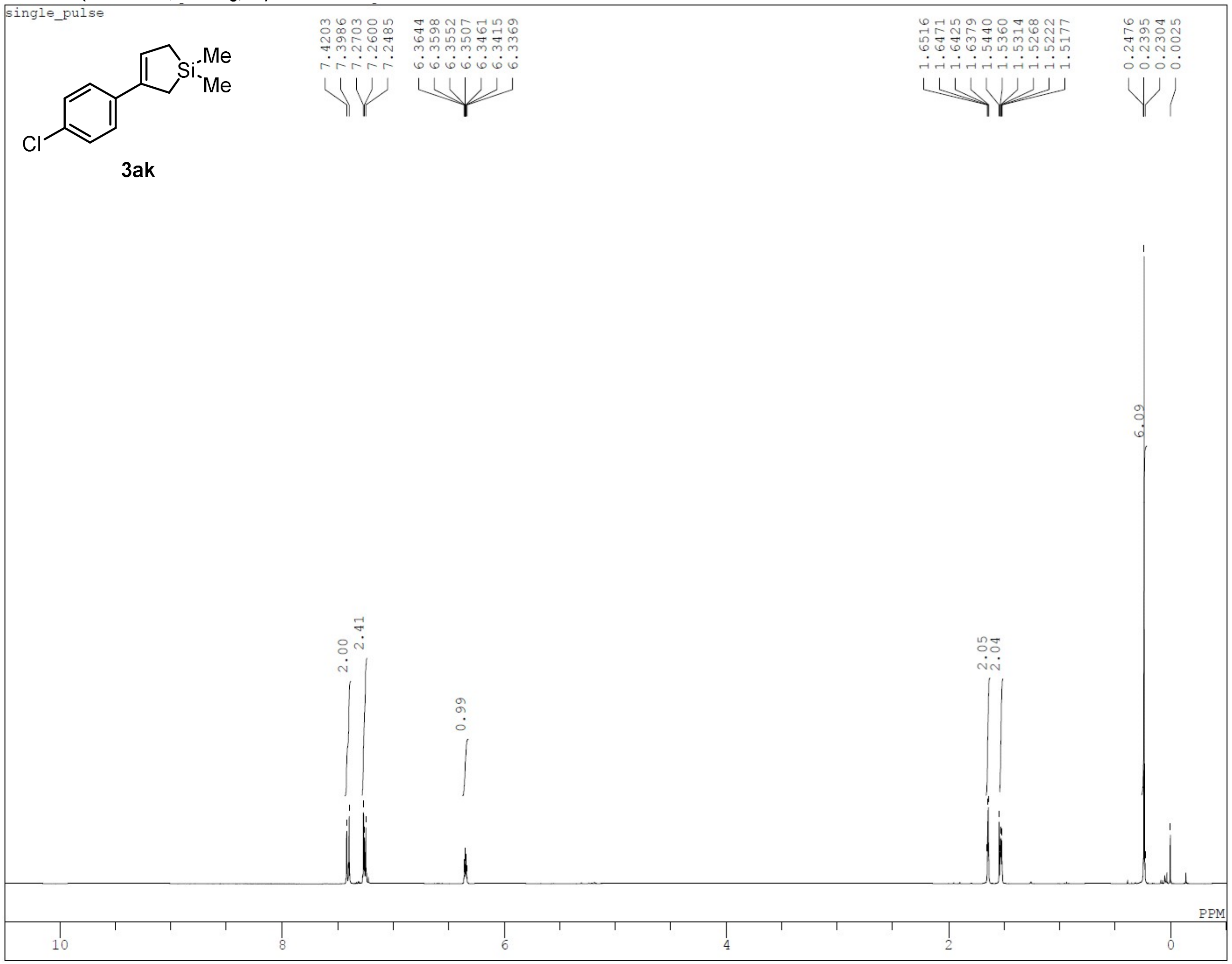

^{13}C NMR (100 MHz, $CDCl_3$, rt)

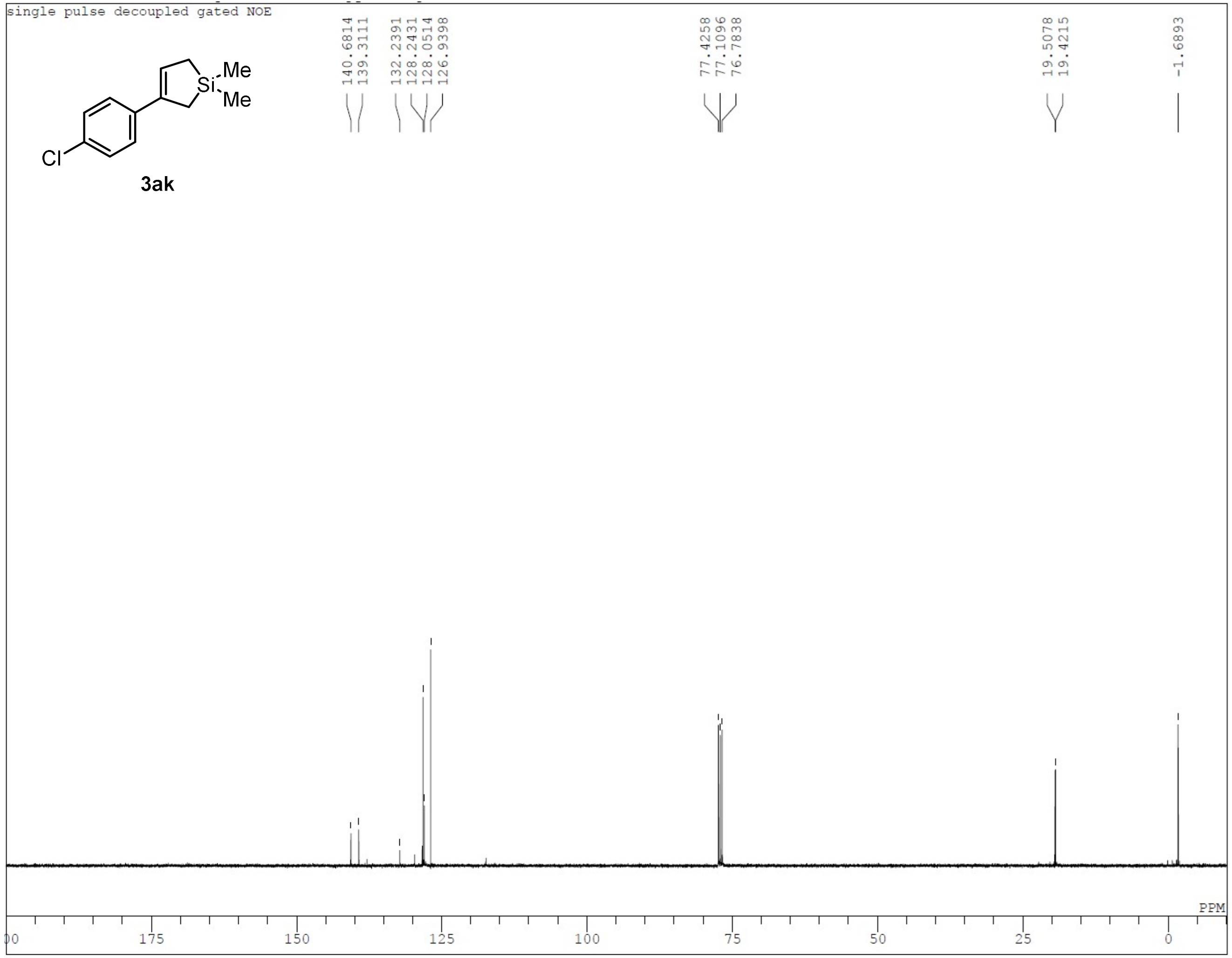

^{29}Si NMR (79 MHz, CDCl$_3$, rt)

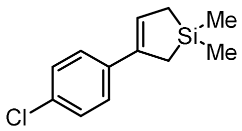

3ak

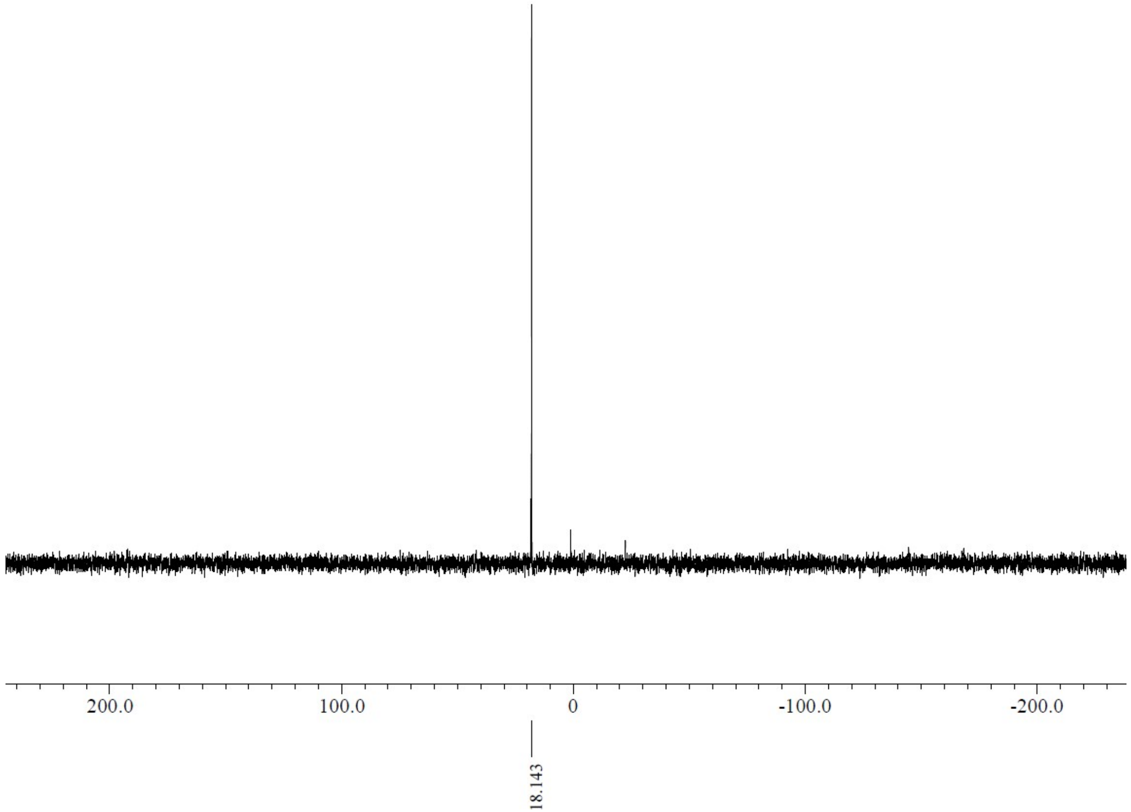

^{1}H NMR (400 MHz, $CDCl_3$, rt)

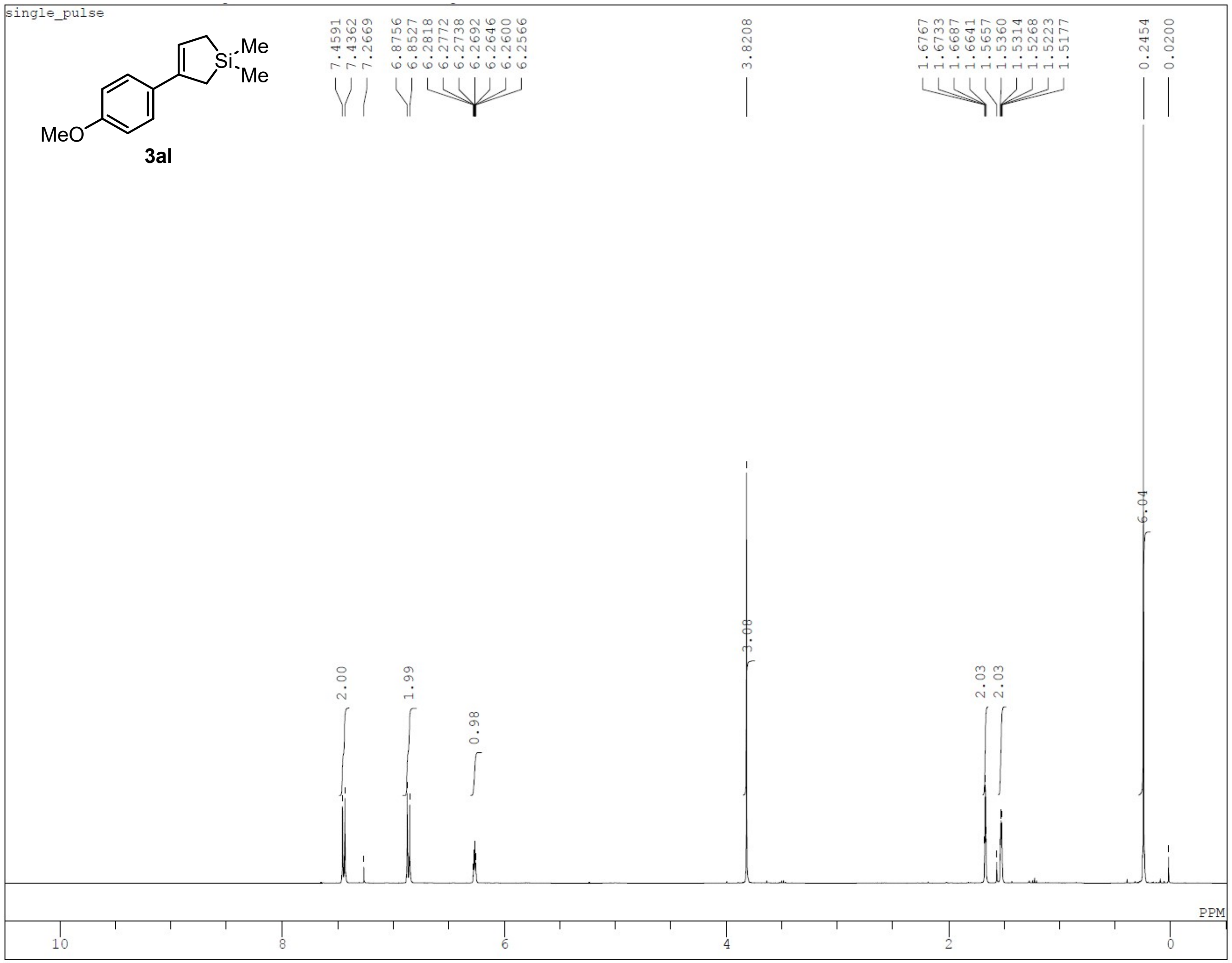

^{13}C NMR (100 MHz, $CDCl_3$, rt)

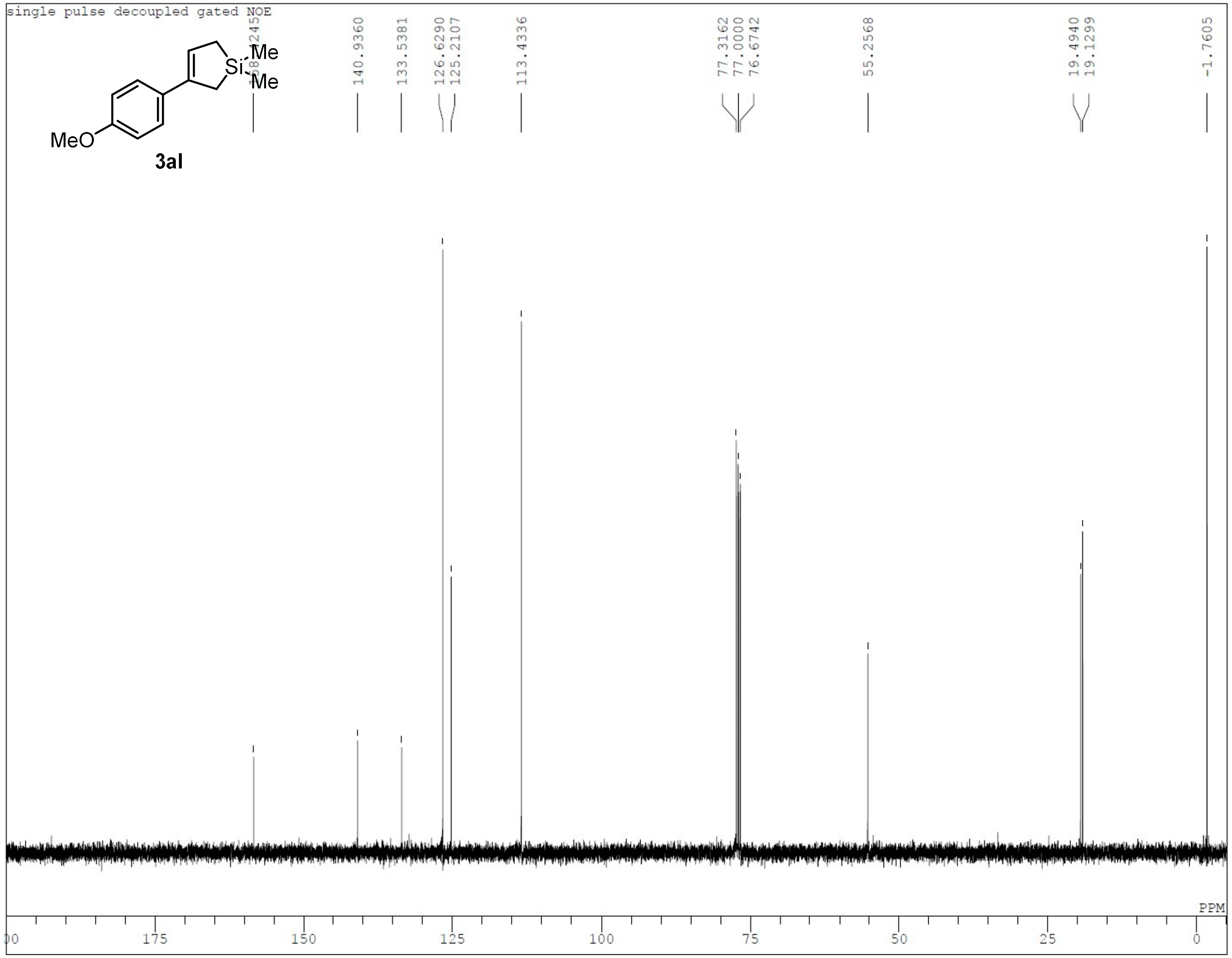

^{29}Si NMR (79 MHz, CDCl$_3$, rt)

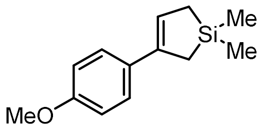

3al

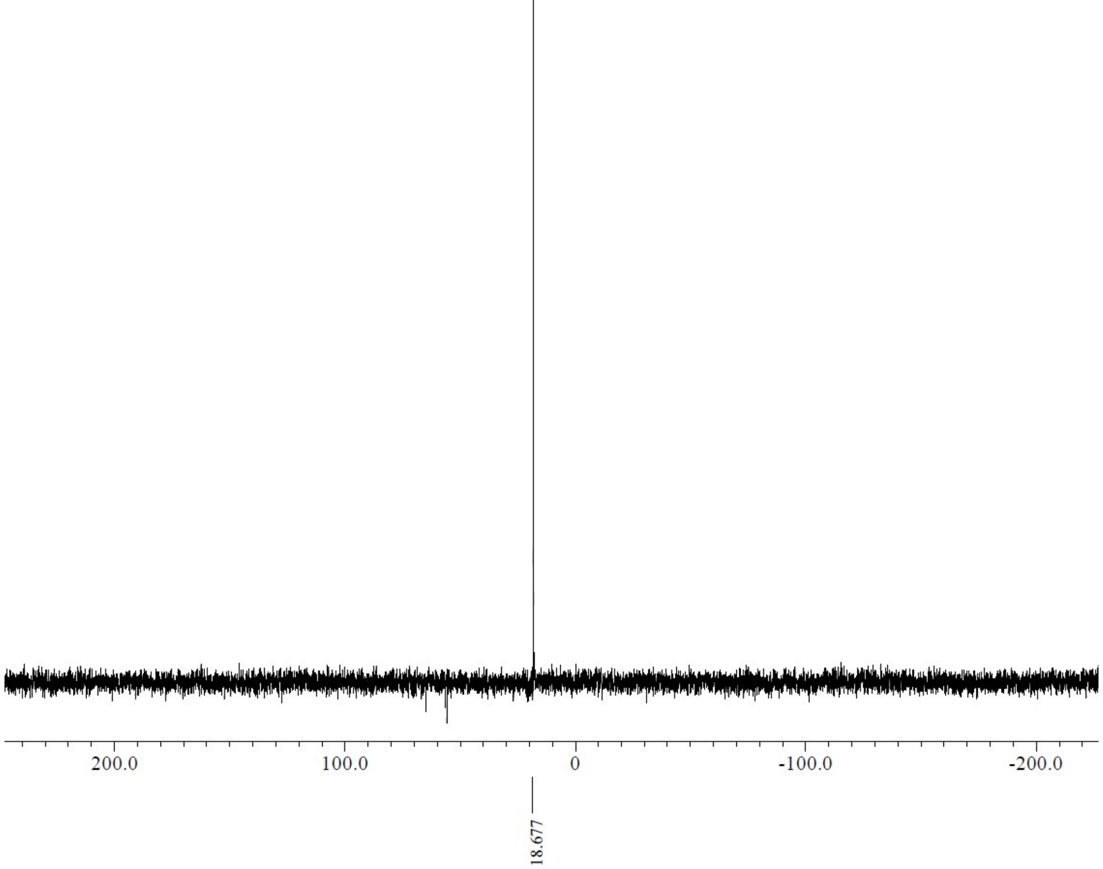

^{1}H NMR (400 MHz, $CDCl_3$, rt)

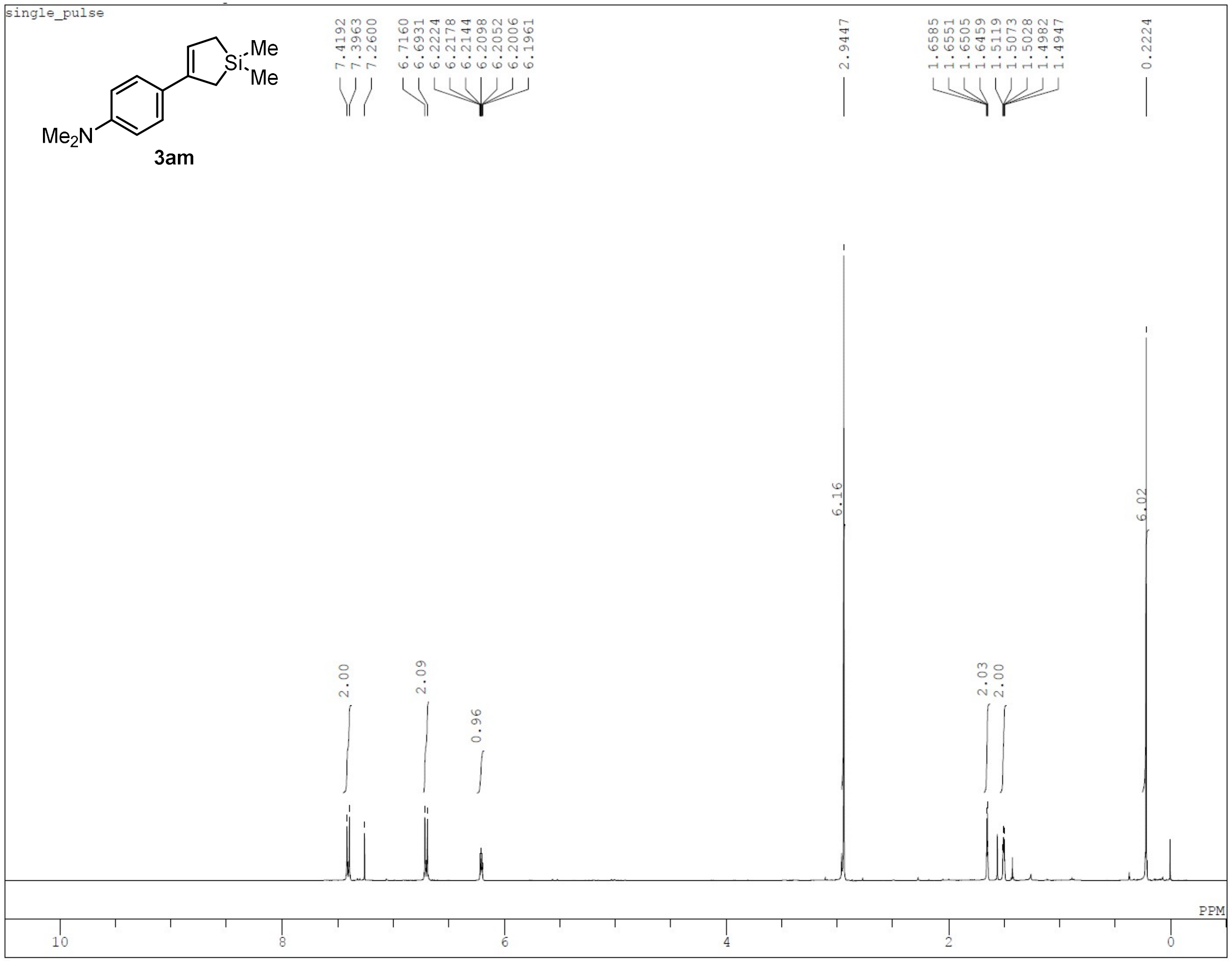

^{13}C NMR (100 MHz, $CDCl_3$, rt)

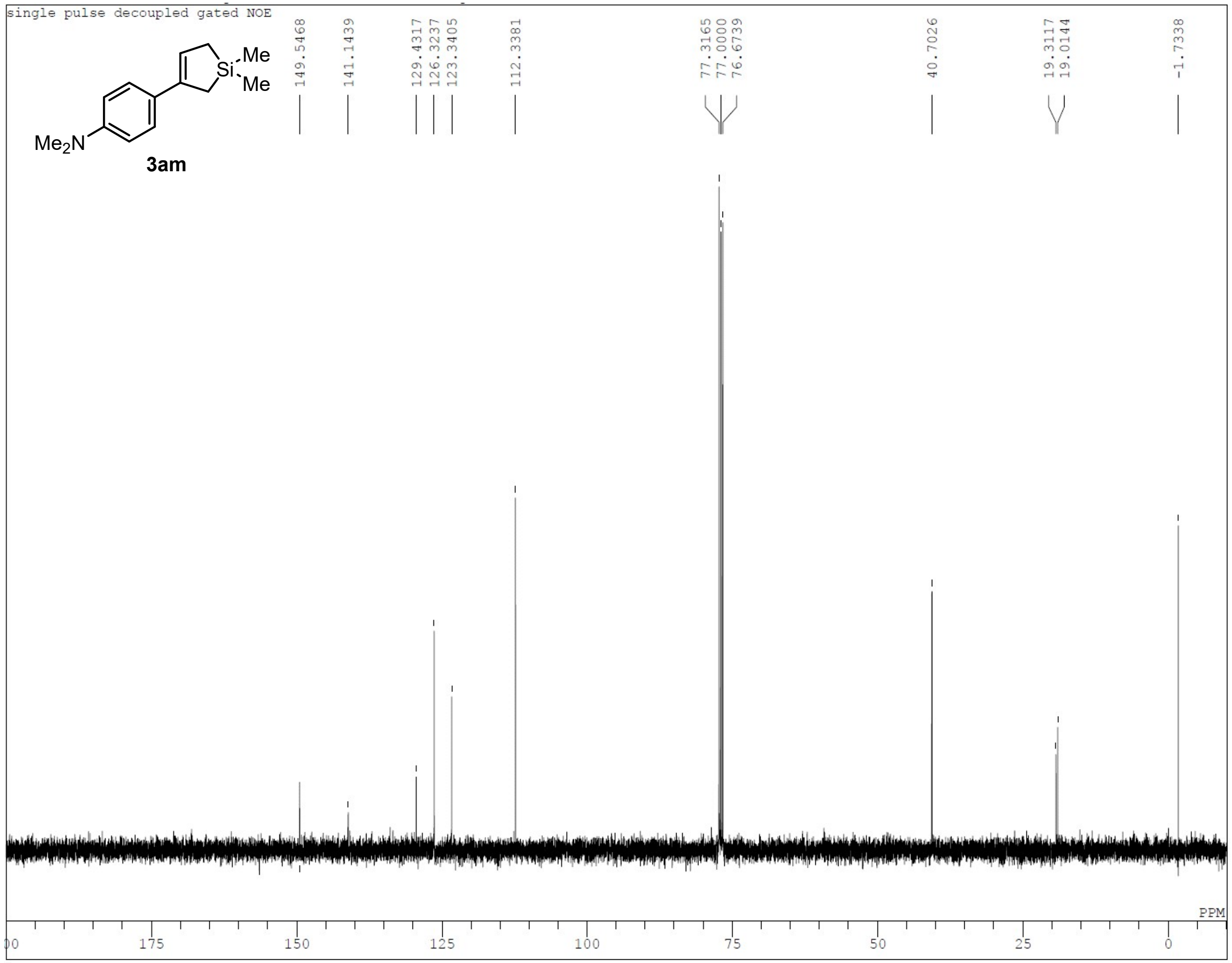

^{29}Si NMR (79 MHz, $CDCl_3$, rt)

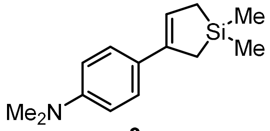

3am

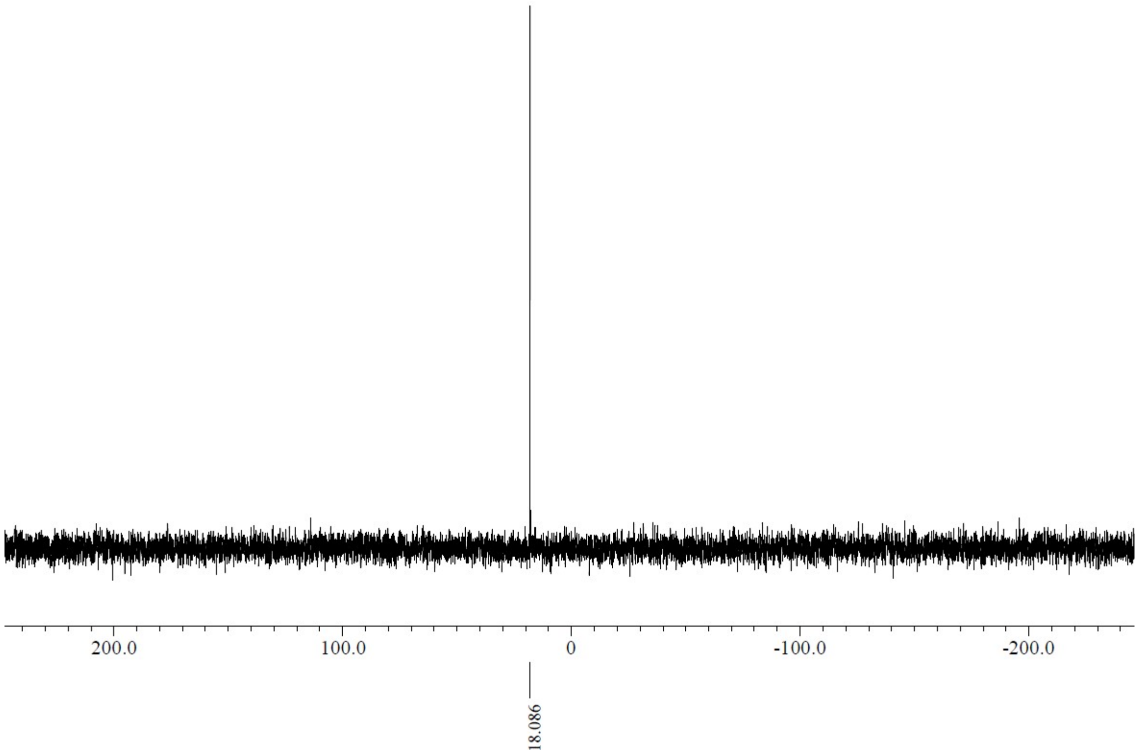

^{1}H NMR (400 MHz, $CDCl_3$, rt)

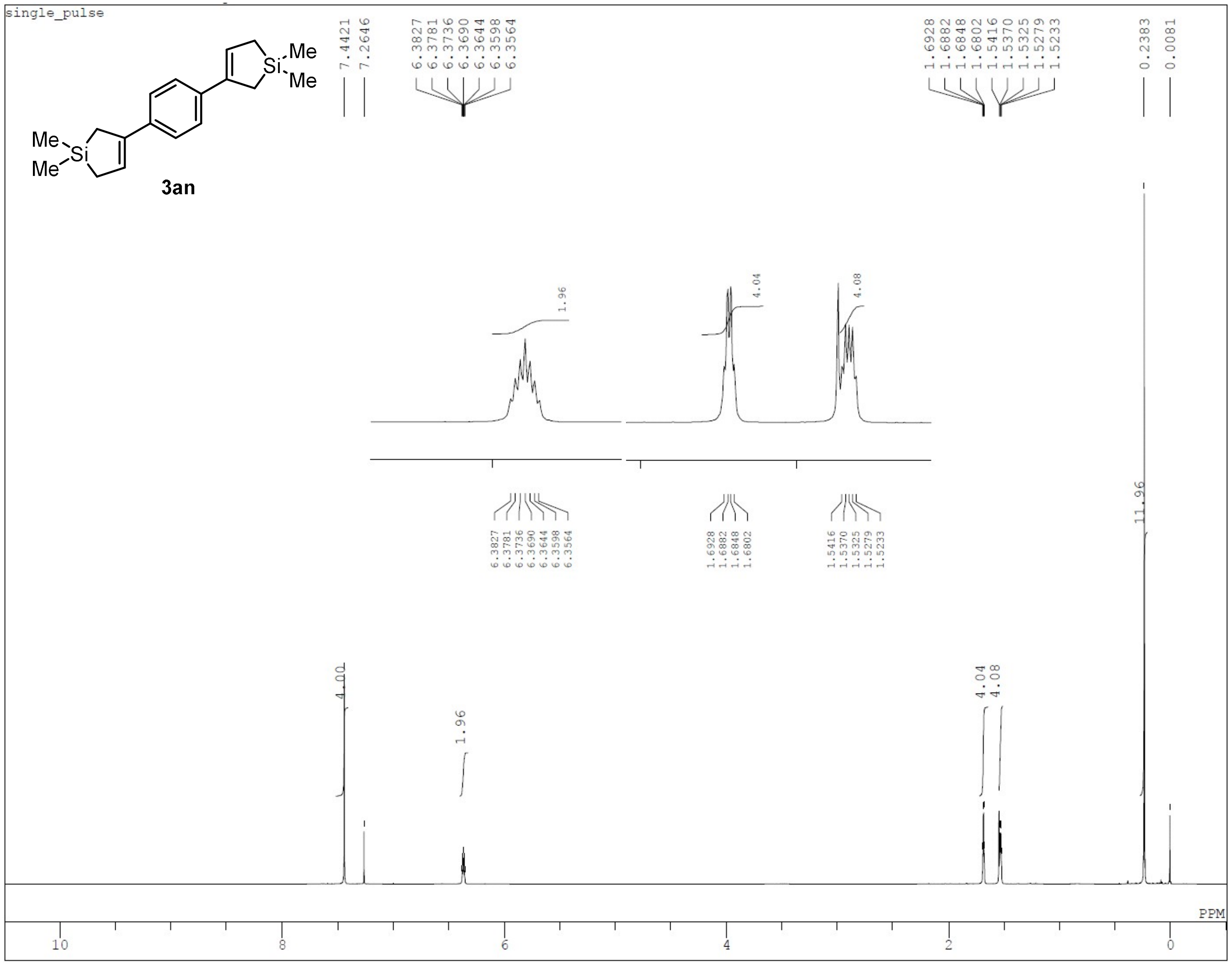

^{13}C NMR (100 MHz, $CDCl_3$, rt)

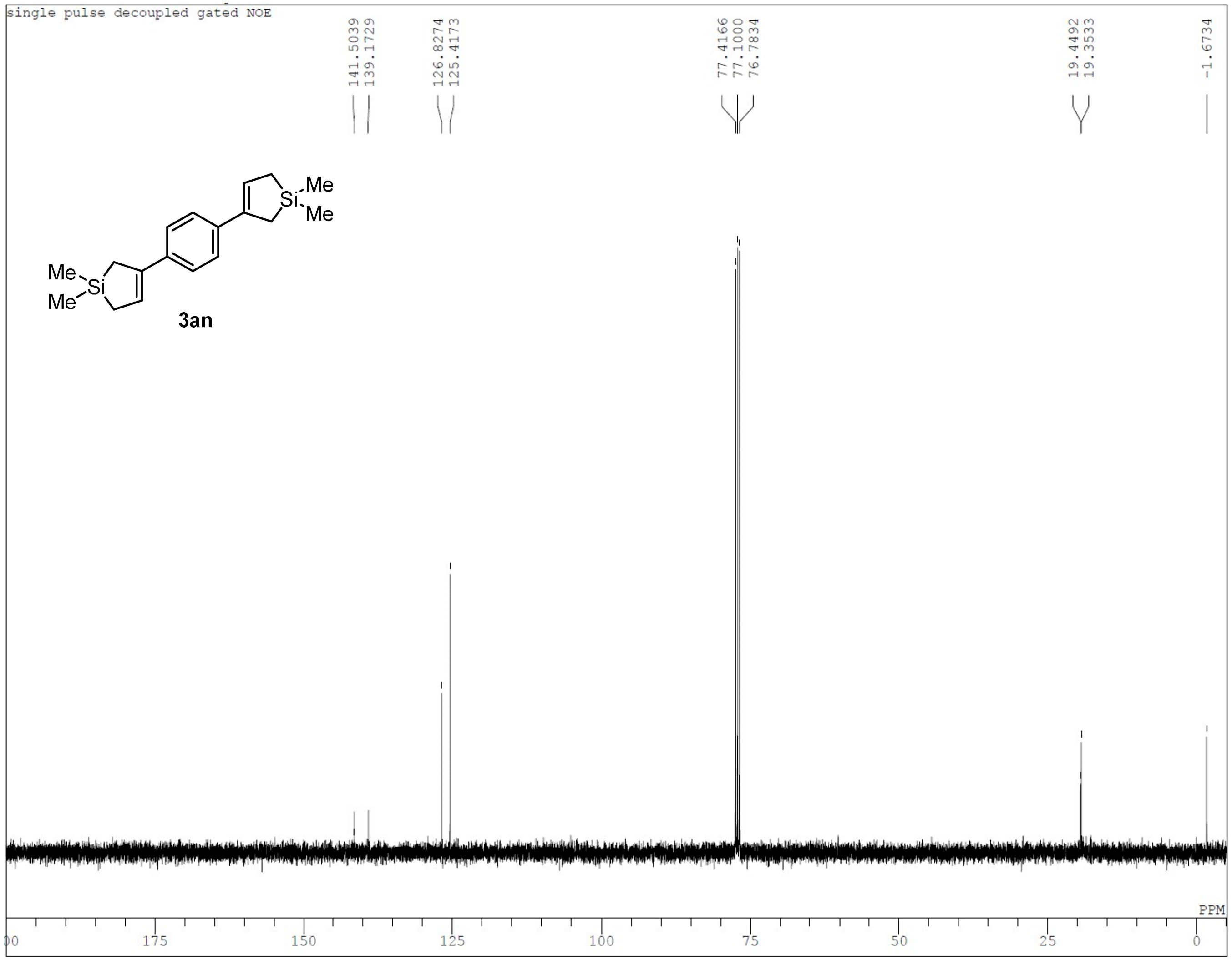

^{29}Si NMR (79 MHz, CDCl$_3$, rt)

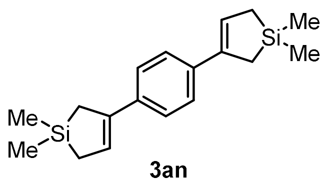

3an

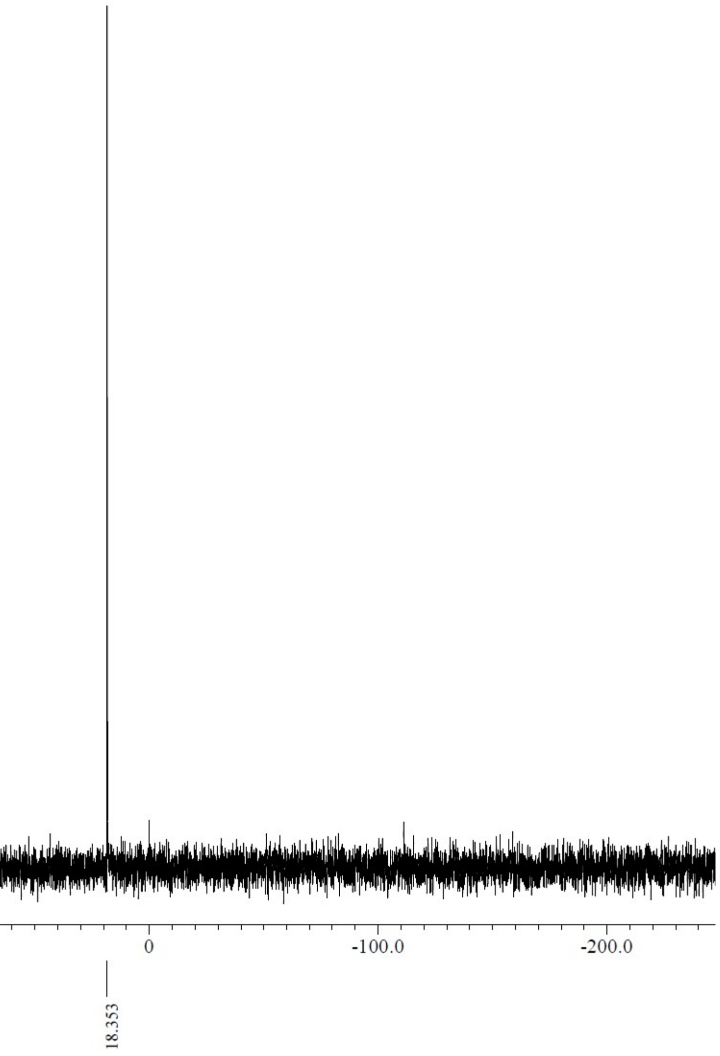

^{1}H NMR (400 MHz, $CDCl_3$, rt)

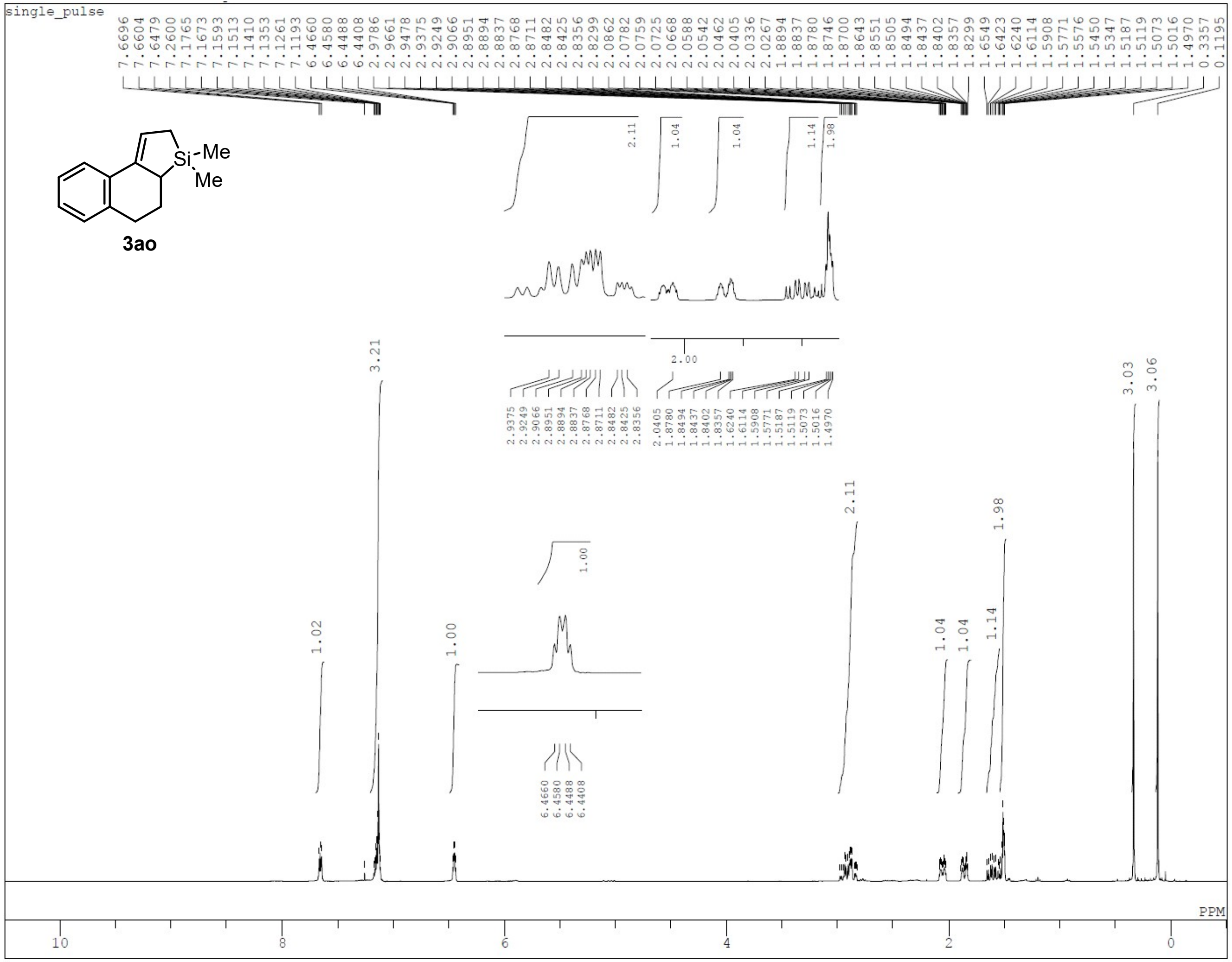

^{13}C NMR (100 MHz, $CDCl_3$, rt)

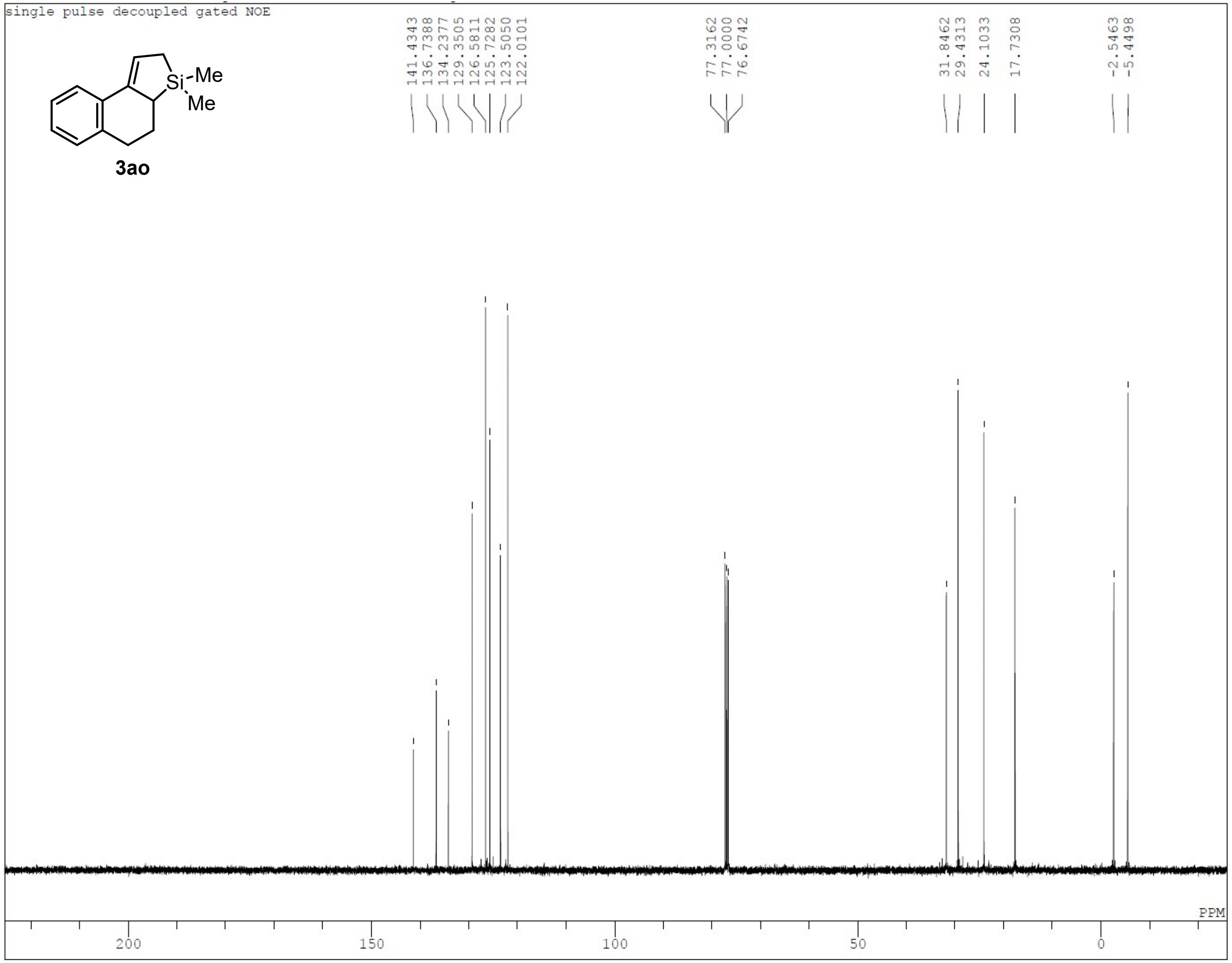

^{29}Si NMR (79 MHz, $CDCl_3$, rt)

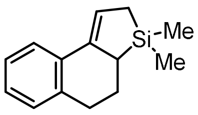

3ao

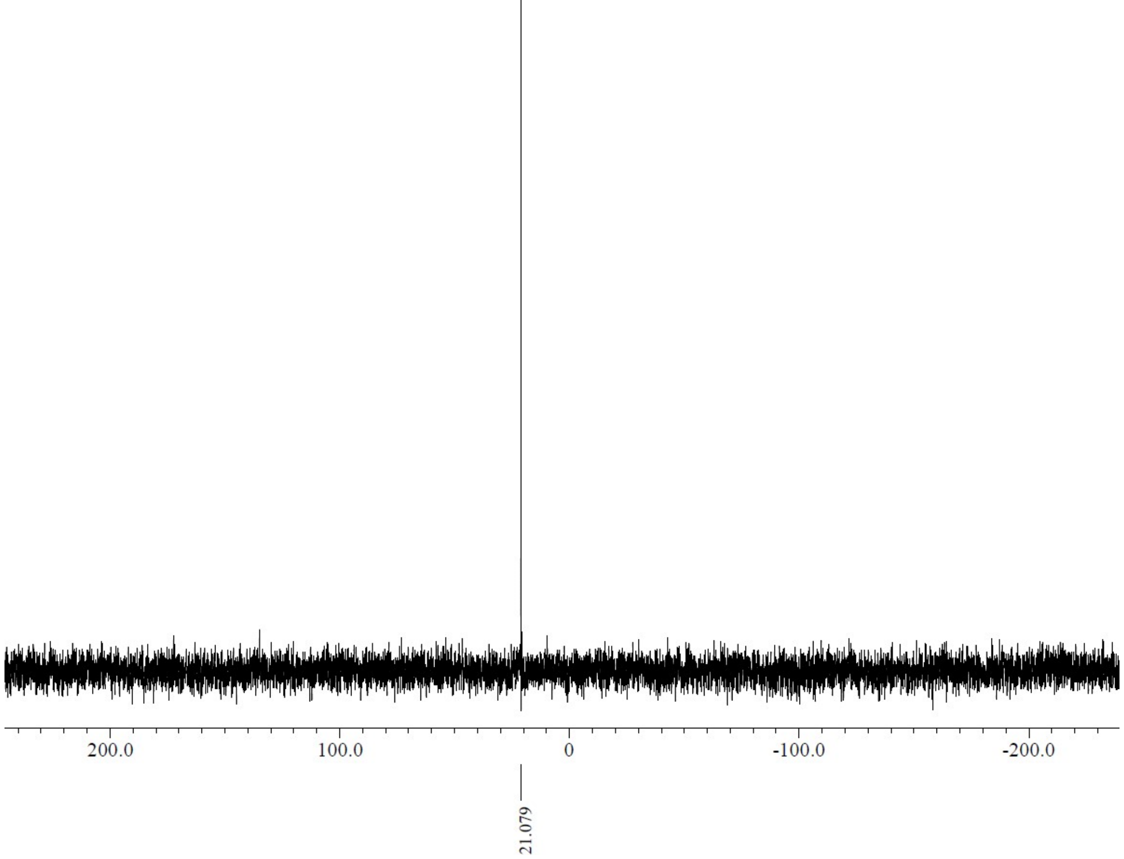

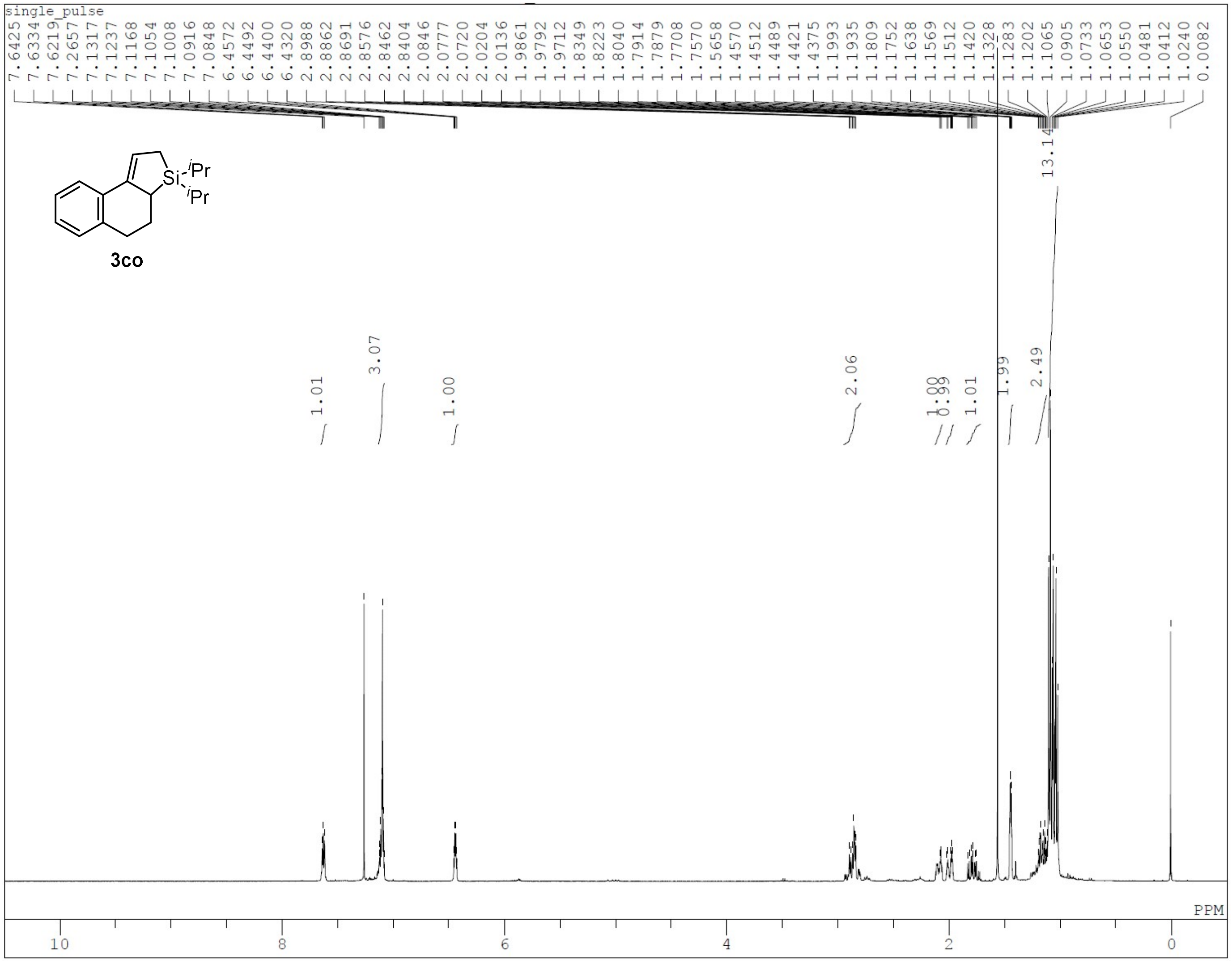
single_pulse
7.6425
7.6334
7.6219
7.2657
7.1317
7.1237
7.1168
7.1054
7.1008
7.0916
7.0848
6.4572
6.4492
6.4400
6.4320
2.8988
2.8862
2.8691
2.8576
2.8462
2.8404
2.0846
2.0777
2.0720
2.0204
2.0136
1.9861
1.9792
1.9712
1.8349
1.8223
1.8040
1.7914
1.7879
1.7708
1.7570
1.5658
1.4570
1.4512
1.4489
1.4421
1.4375
1.1993
1.1935
1.1809
1.1752
1.1638
1.1569
1.1512
1.1420
1.1328
1.1283
1.1202
1.1065
1.0905
1.0733
1.0653
1.0550
1.0481
1.0412
1.0240
0.0082
13.14
1.01
3.07
1.00
2.06
1.00
0.99
1.01
1.99
2.49
Si
iPr
iPr
3co
PPM
10
8
6
4
2
0

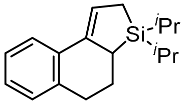

3co

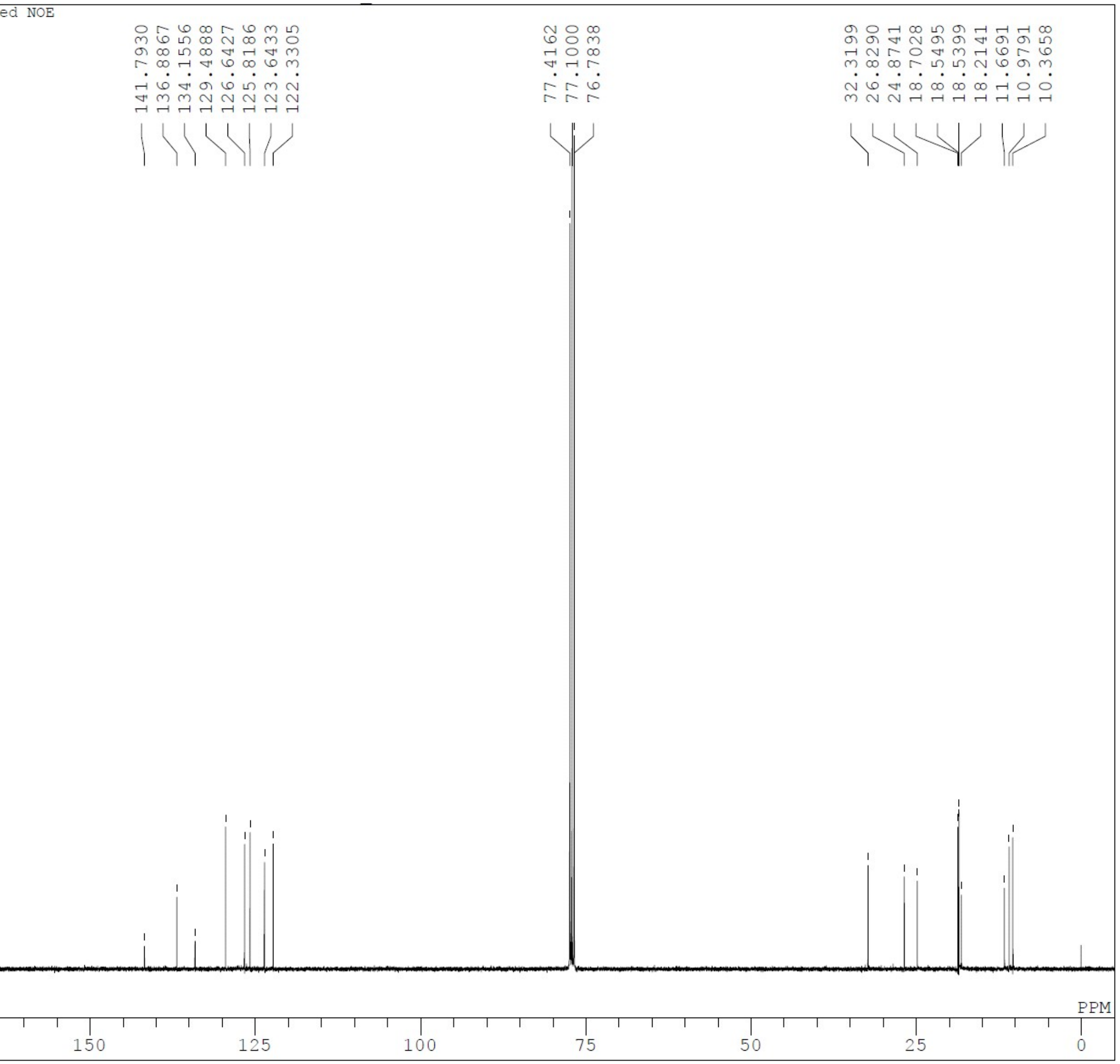

single pulse decoupled gated NOE
141.7930
136.8867
134.1556
129.4888
126.6427
125.8186
123.6433
122.3305
77.4162
77.1000
76.7838
32.3199
26.8290
24.8741
18.7028
18.5495
18.5399
18.2141
11.6691
10.9791
10.3658
PPM
175
150
125
100
75
50
25
0

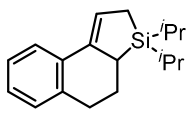

Si
ⁱPr
ⁱPr

3co

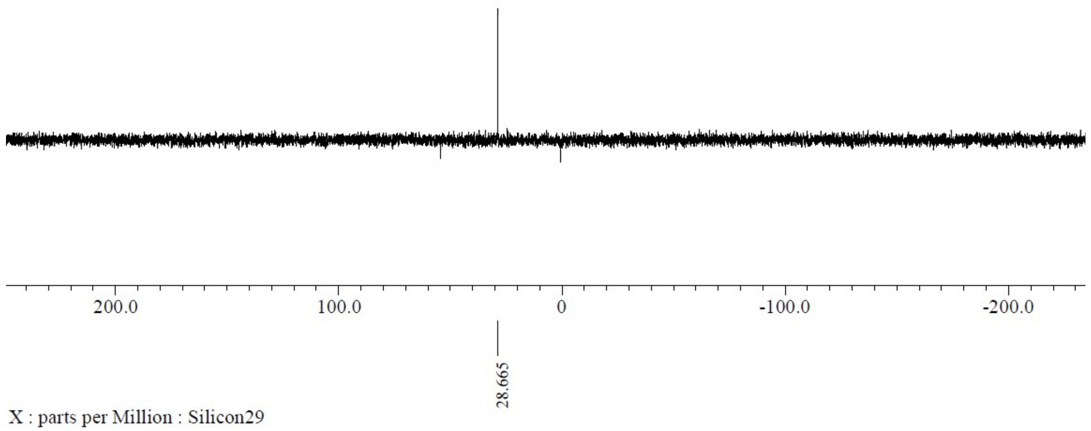

200.0
100.0
0
-100.0
-200.0
28.665
X : parts per Million : Silicon29

^{1}H NMR (400 MHz, $CDCl_3$, rt)

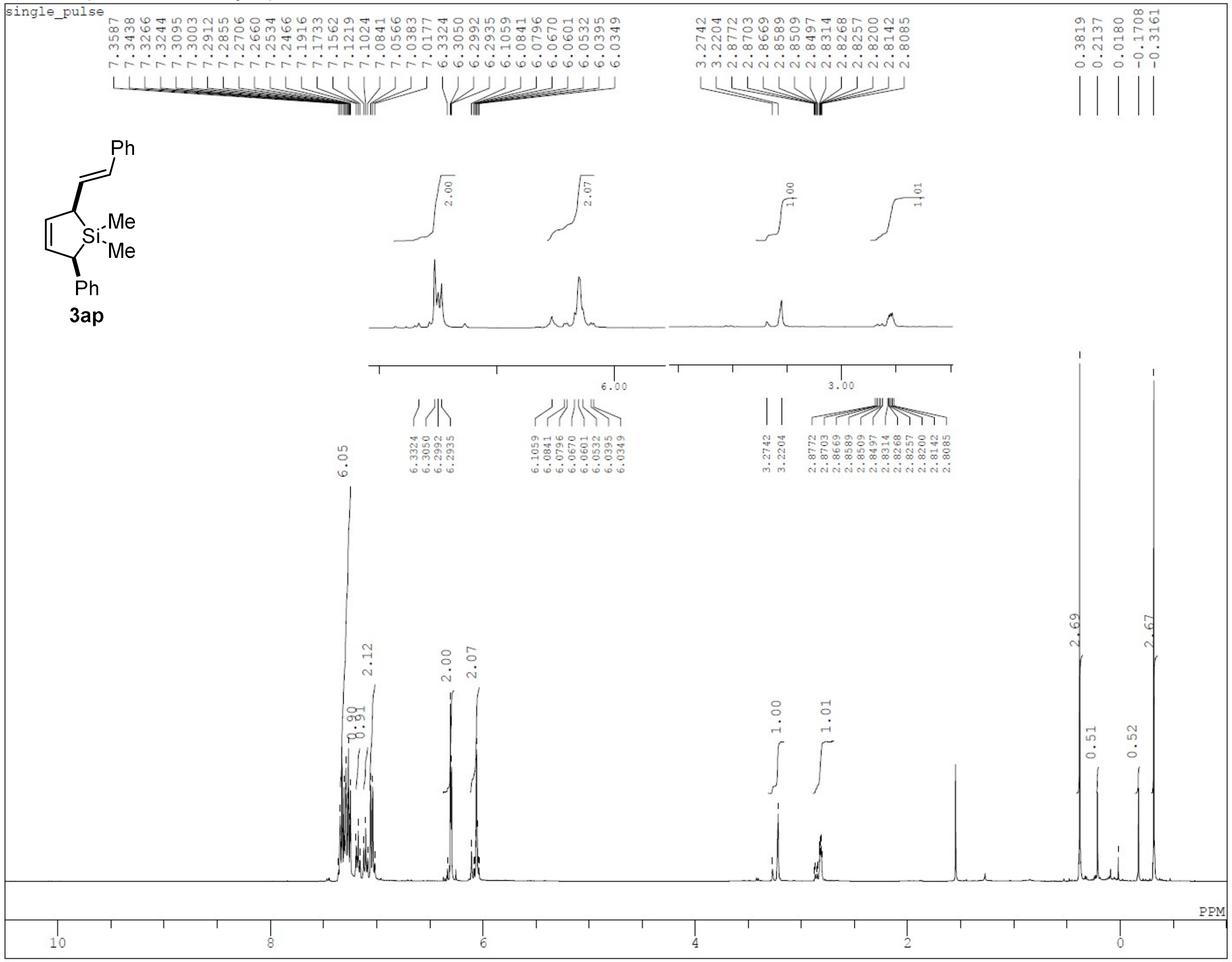

^{13}C NMR (100 MHz, $CDCl_3$, rt)

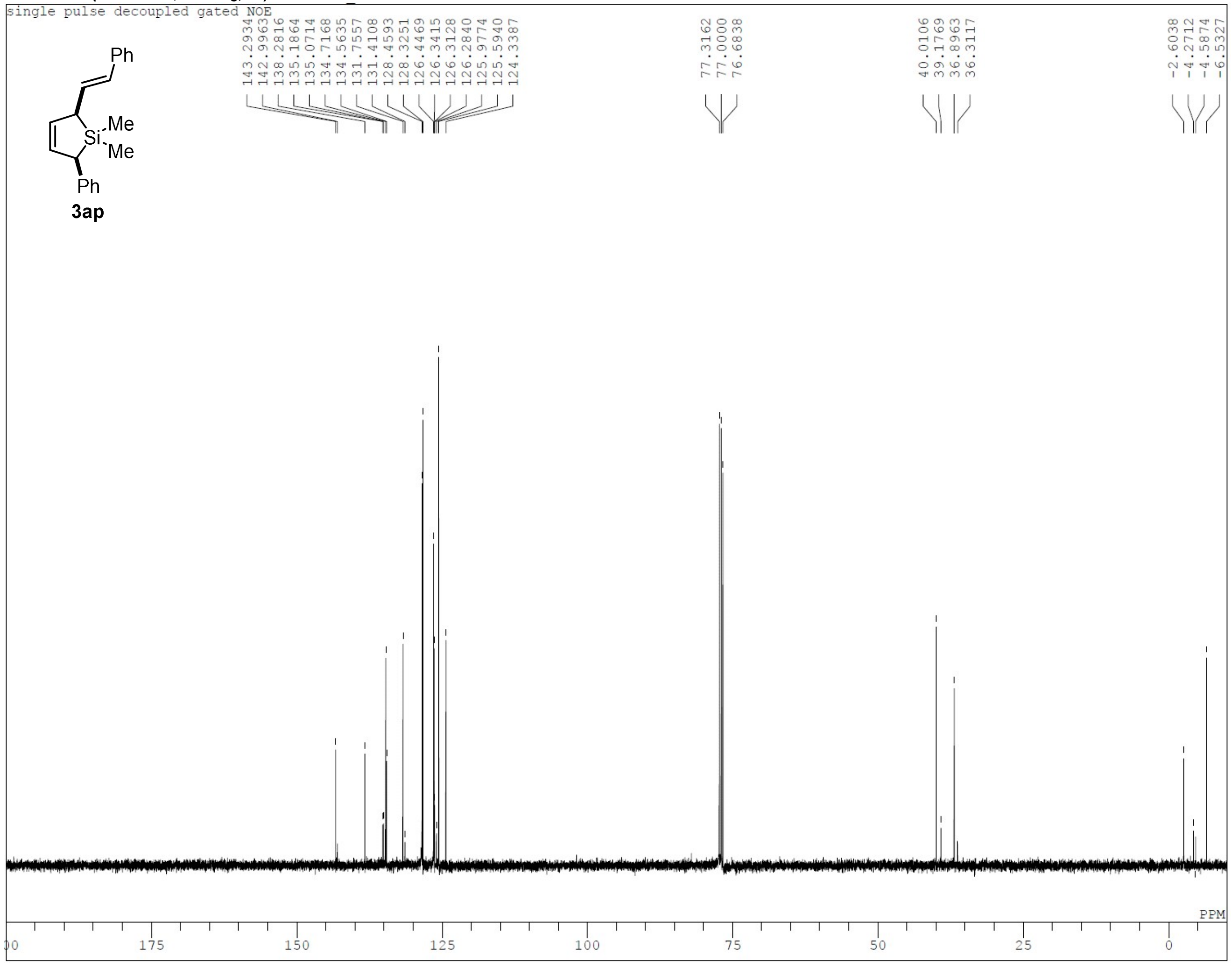

^{29}Si NMR (79 MHz, $CDCl_3$, rt)

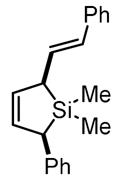

3ap

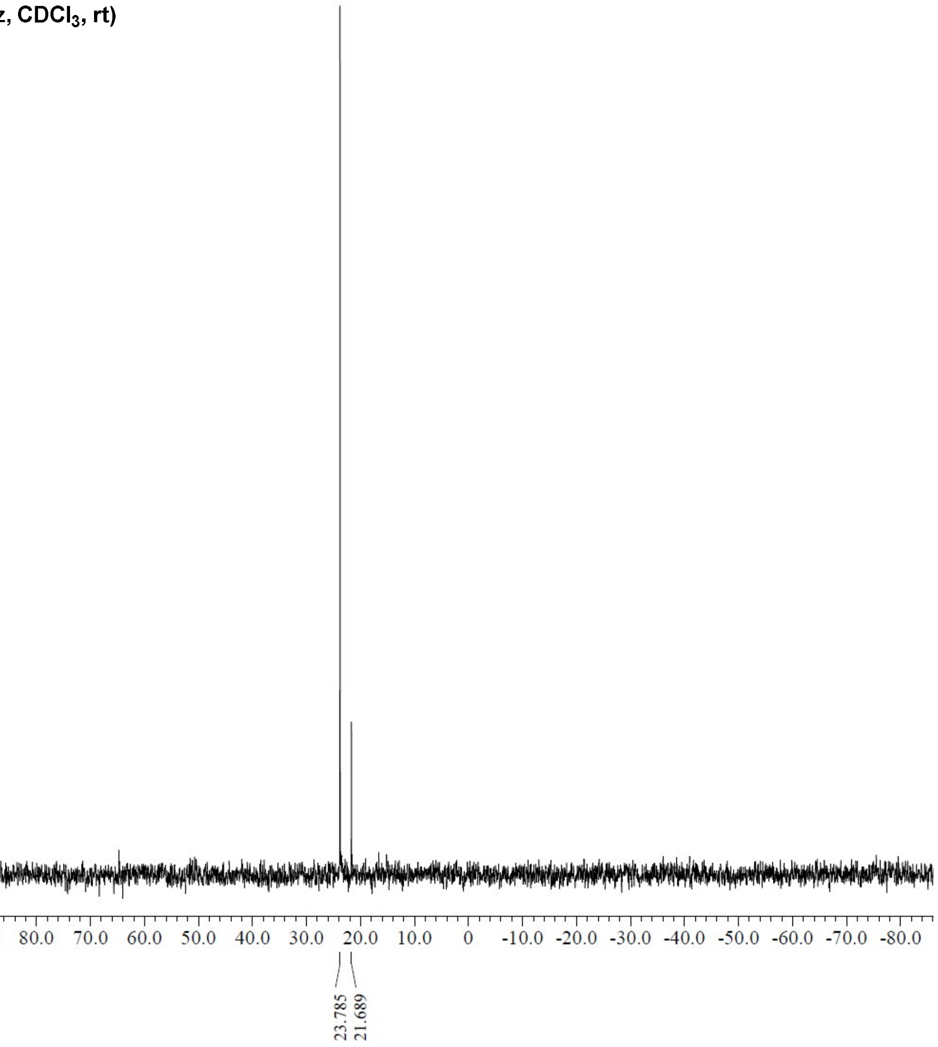

^{1}H NMR (400 MHz, $CDCl_3$, rt)

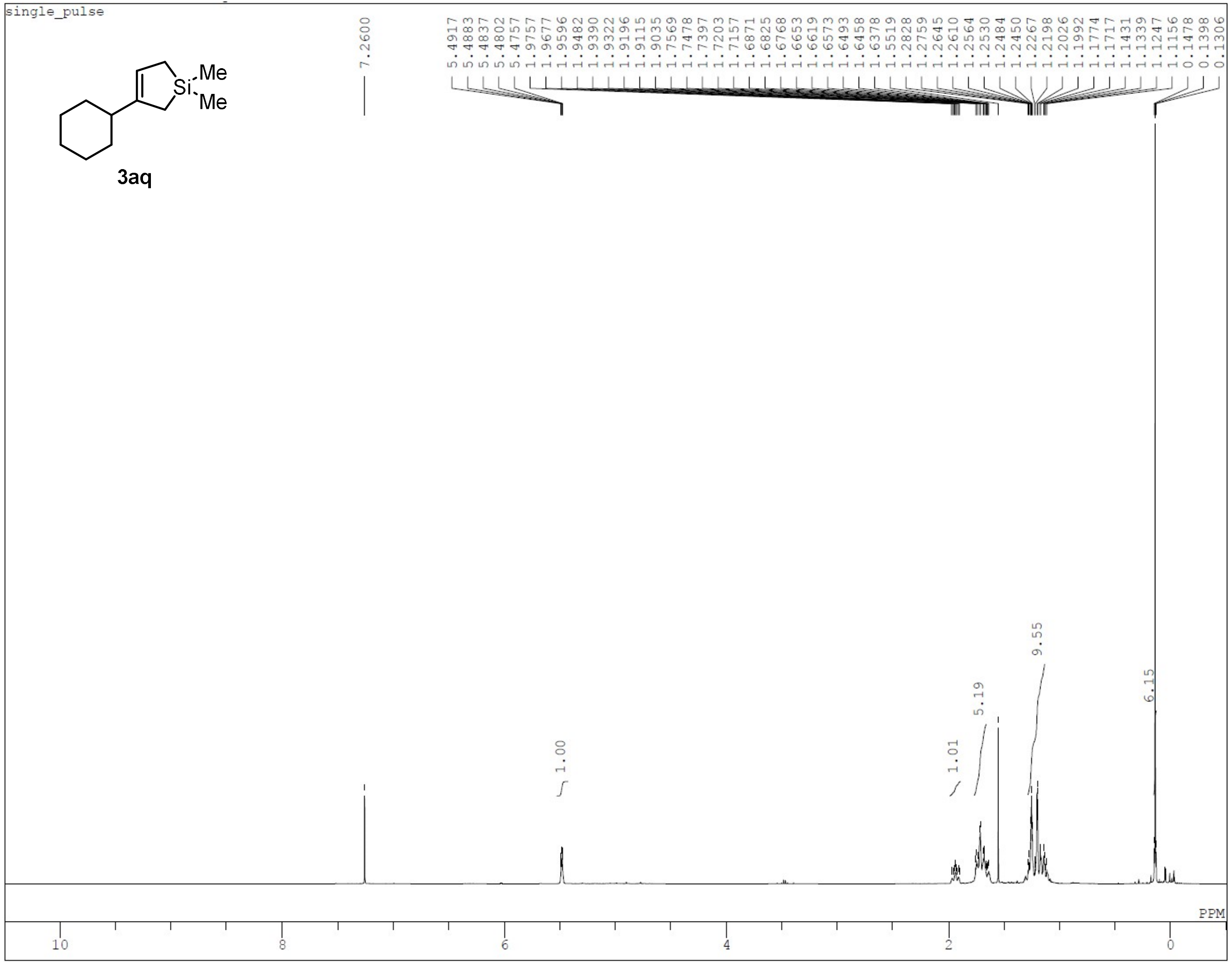

^{13}C NMR (100 MHz, $CDCl_3$, rt)

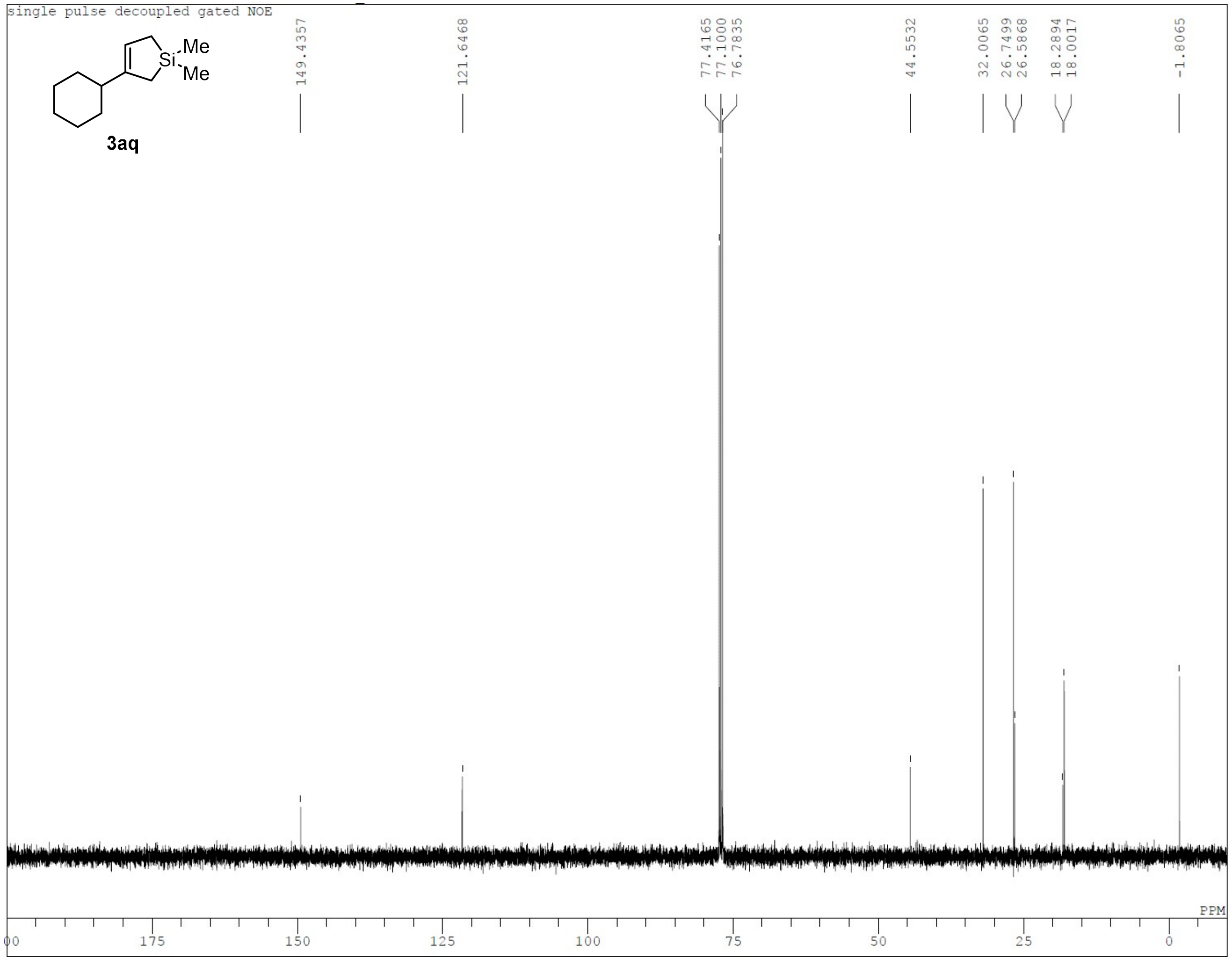

^{29}Si NMR (79 MHz, $CDCl_3$, rt)

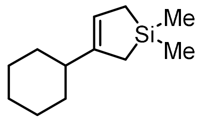

3aq

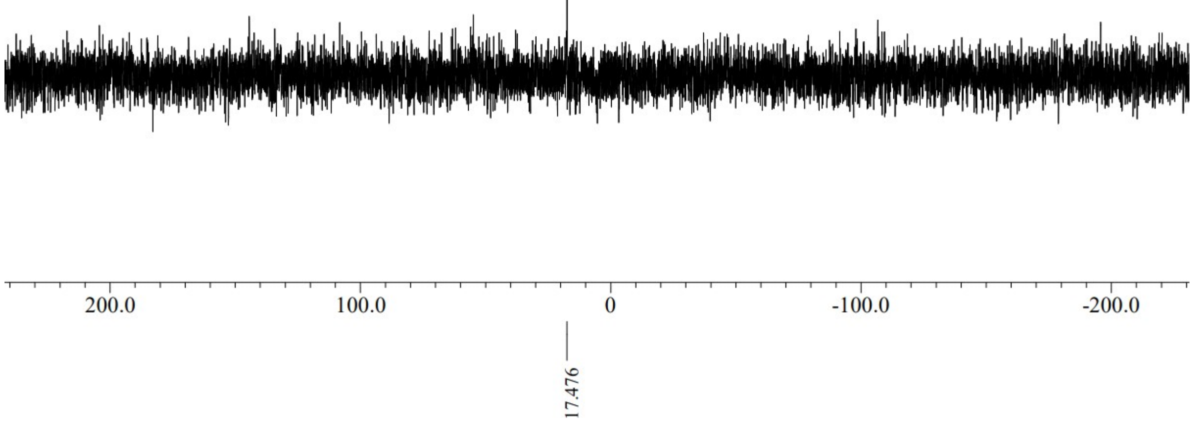

^{1}H NMR (400 MHz, $CDCl_3$, rt)

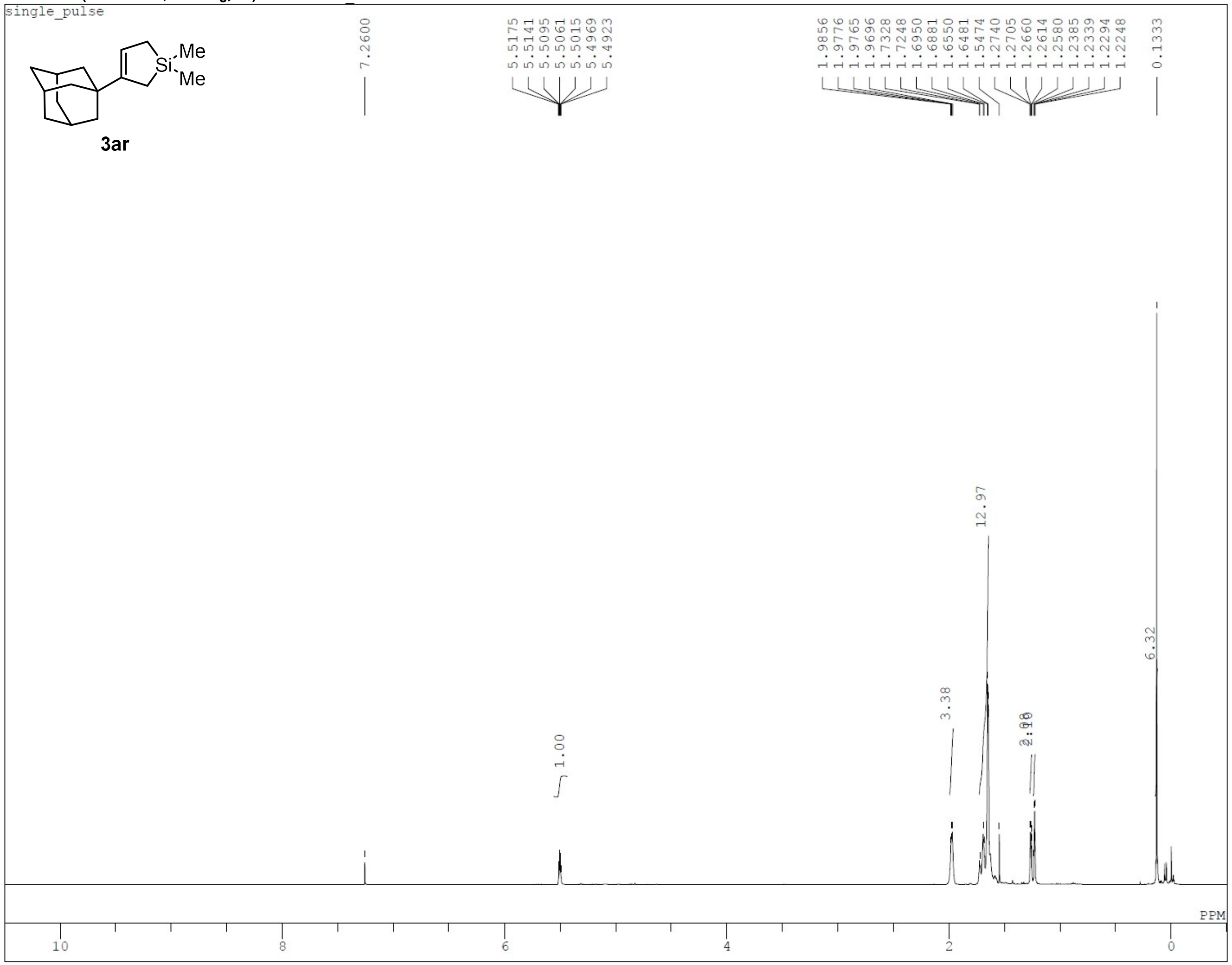

^{13}C NMR (100 MHz, $CDCl_3$, rt)

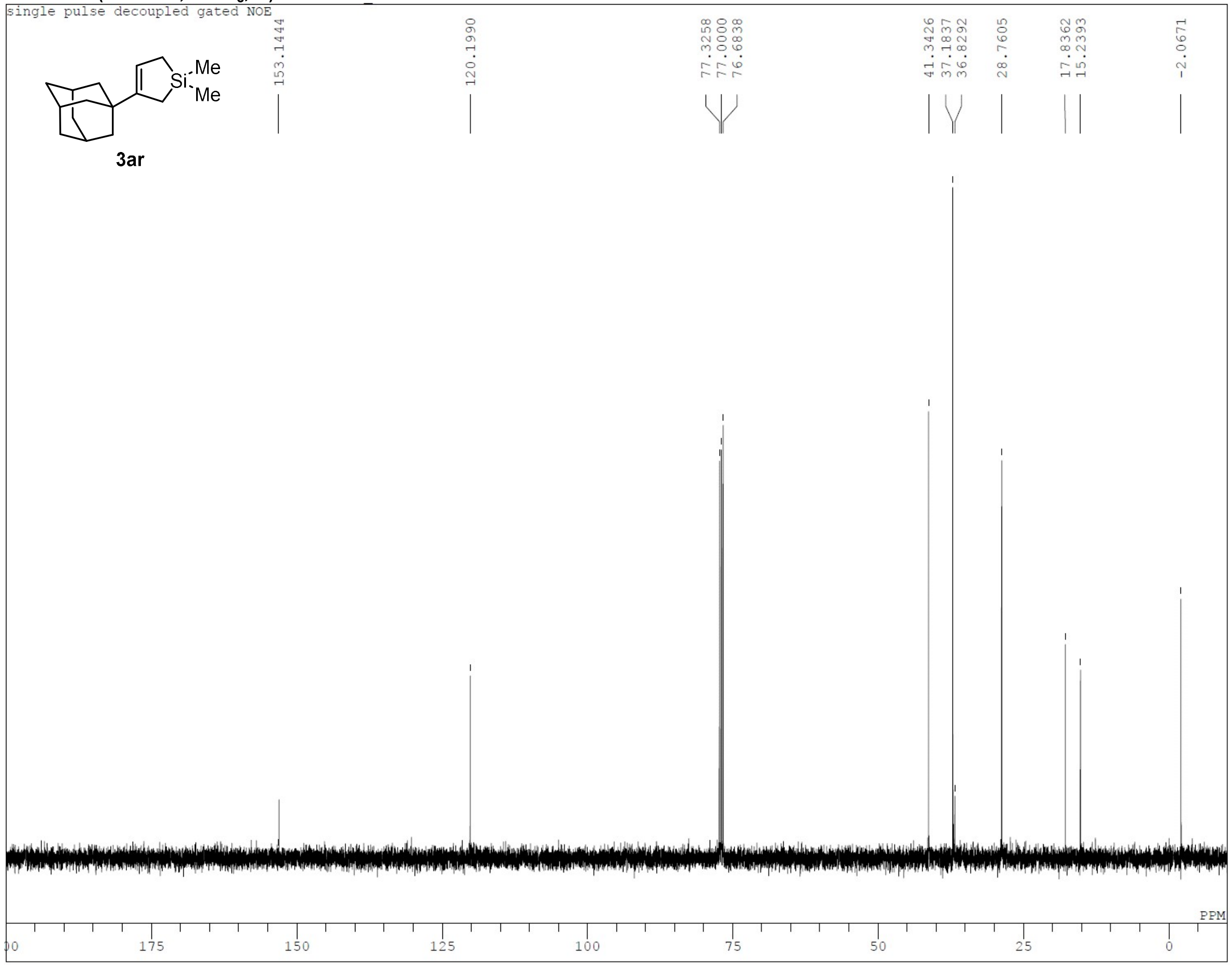

^{29}Si NMR (79 MHz, $CDCl_3$, rt)

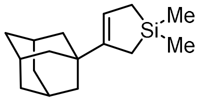

3ar

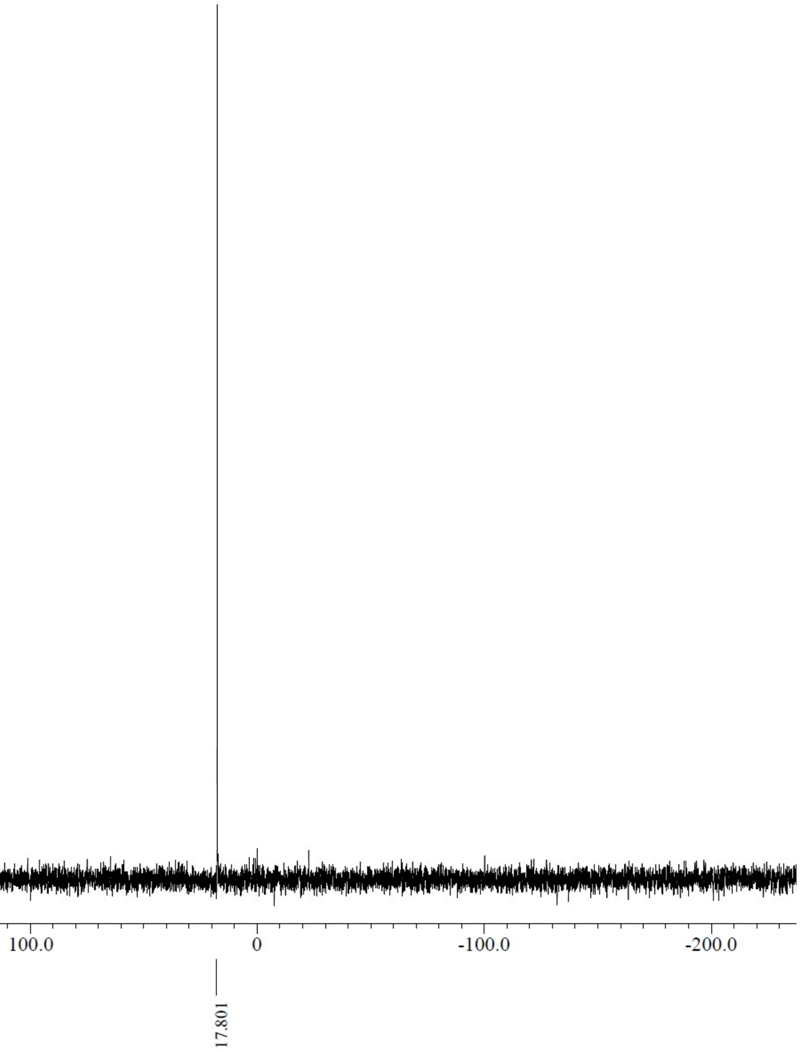

^{1}H NMR (400 MHz, CDCl$_3$, rt)

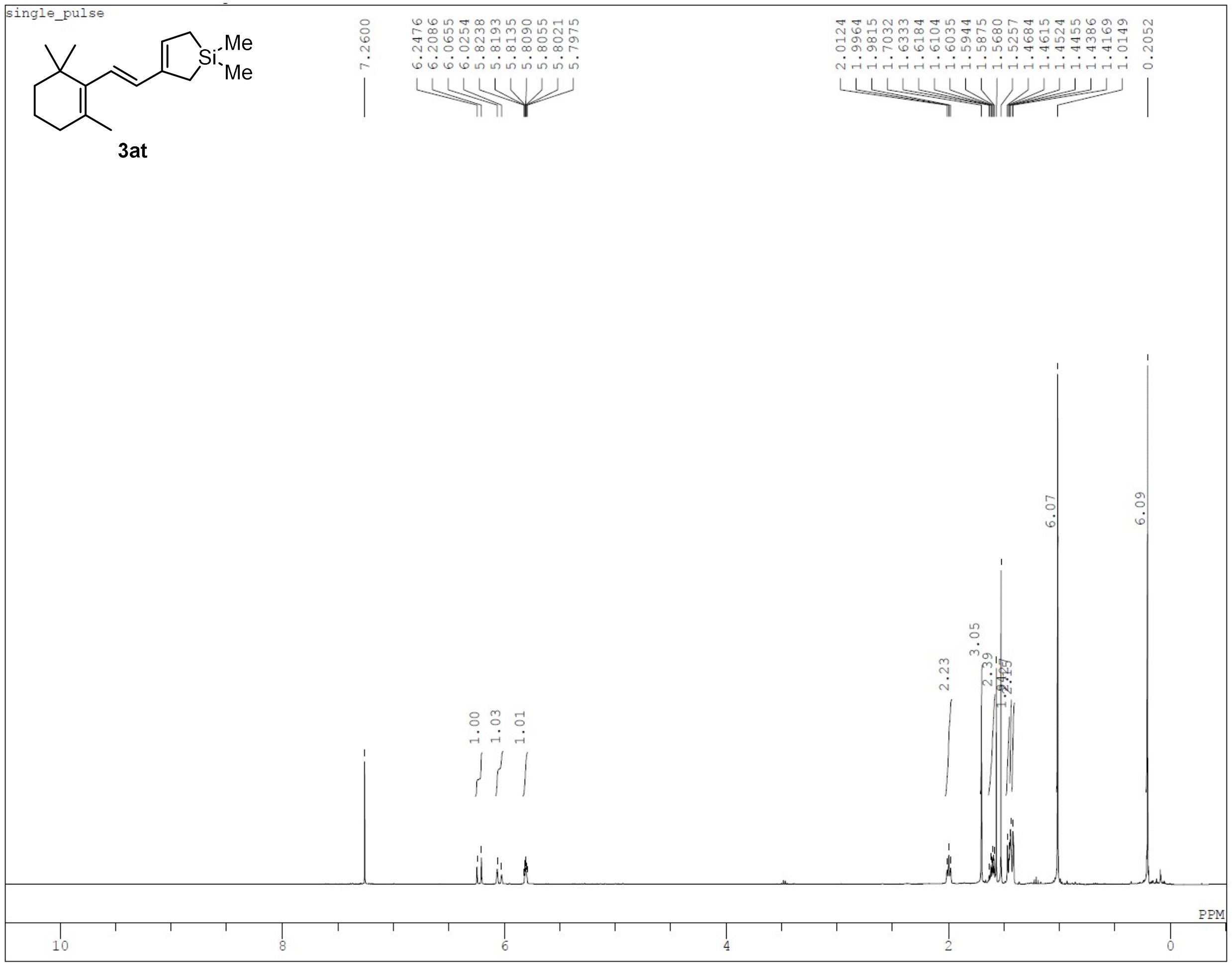

^{13}C NMR (100 MHz, $CDCl_3$, rt)

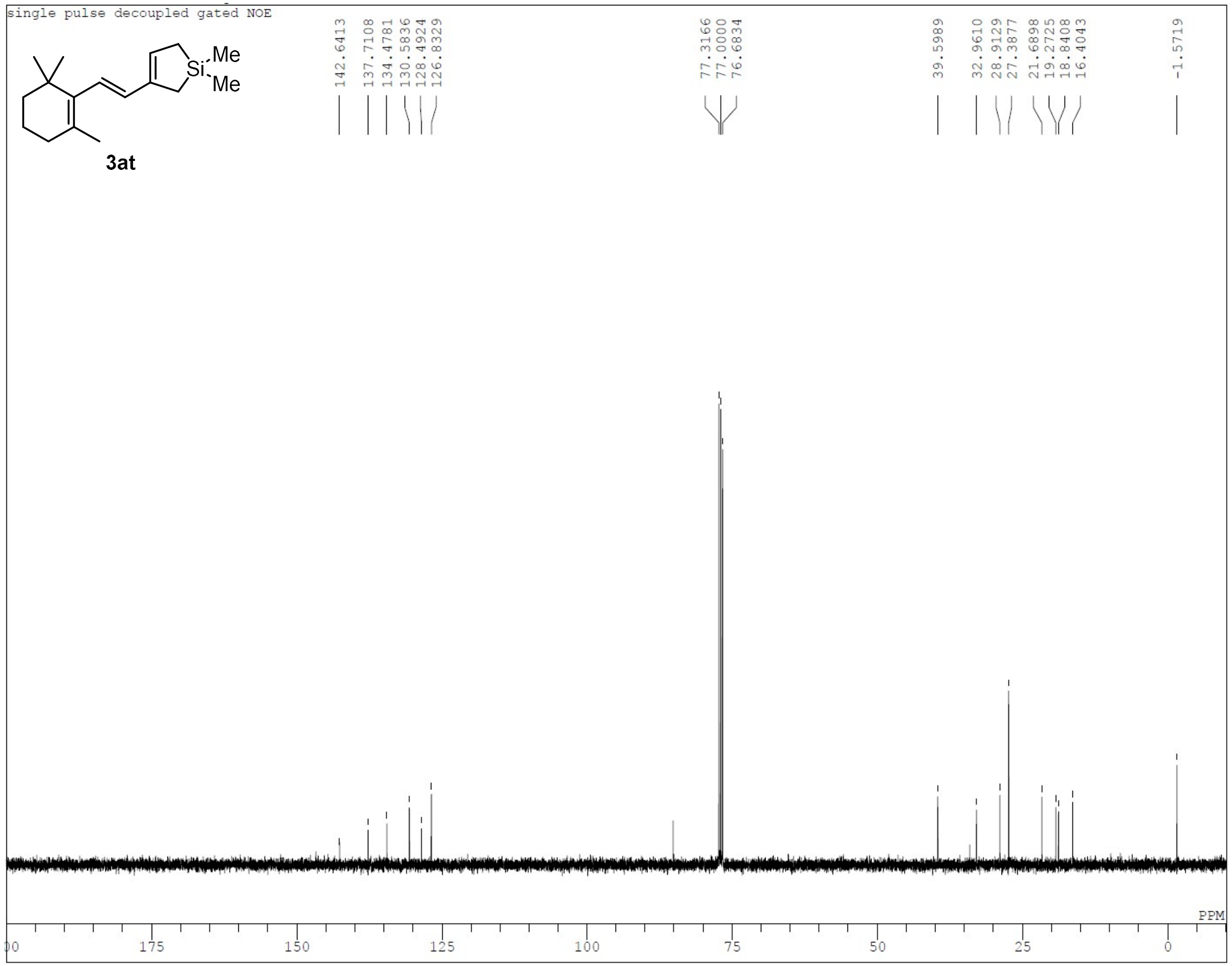

^{29}Si NMR (79 MHz, $CDCl_3$, rt)

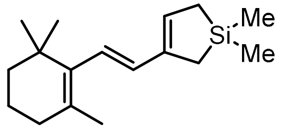

3at

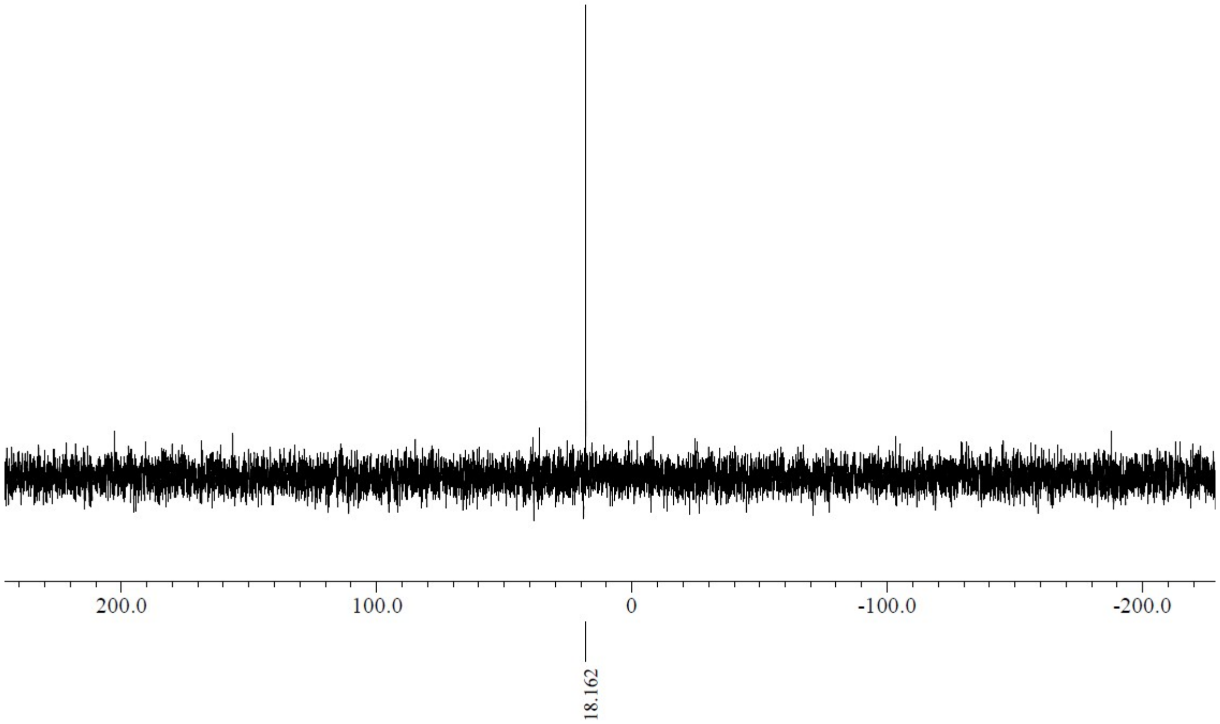

^{1}H NMR (400 MHz, $CDCl_3$, rt)

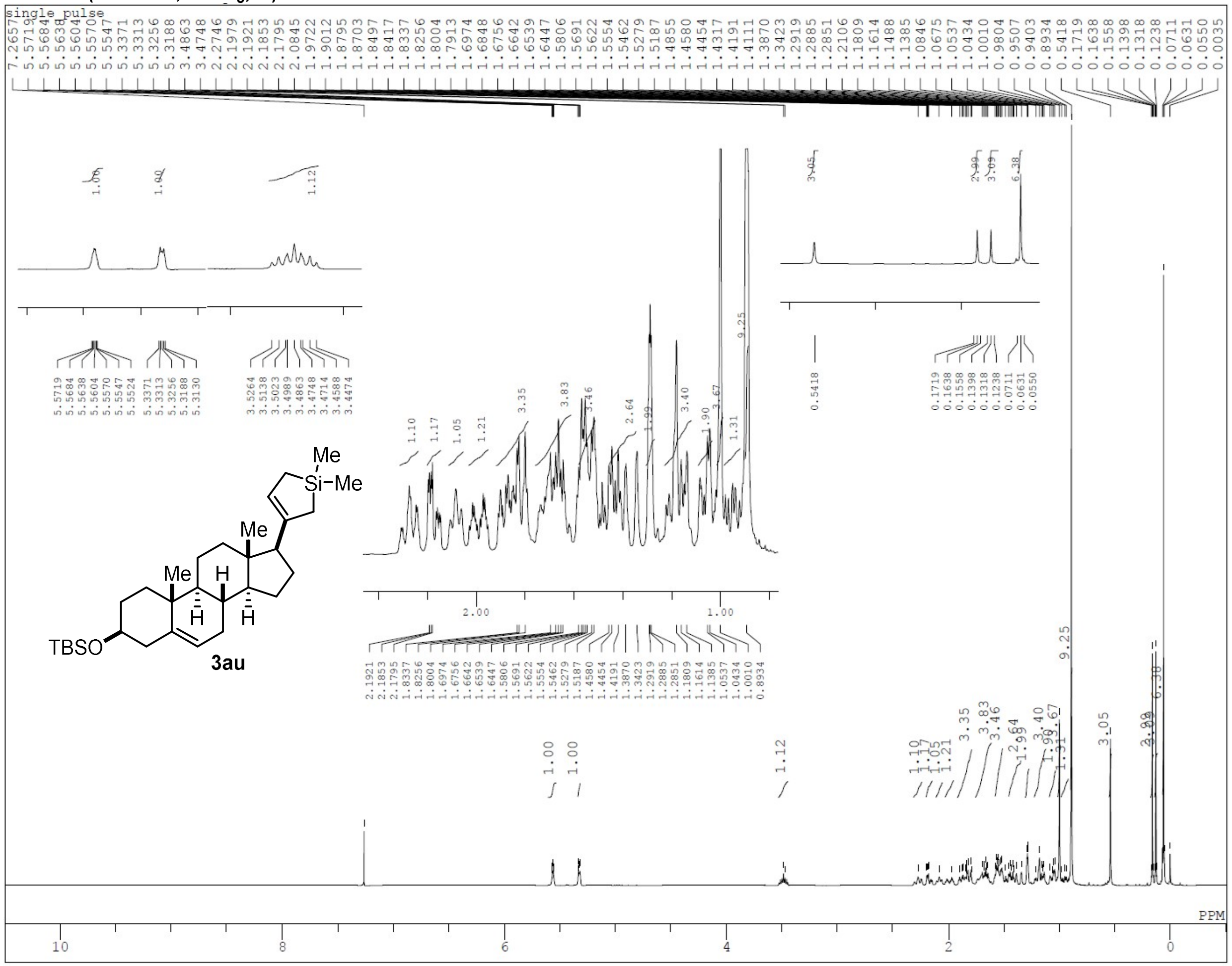

^{13}C NMR (100 MHz, $CDCl_3$, rt)

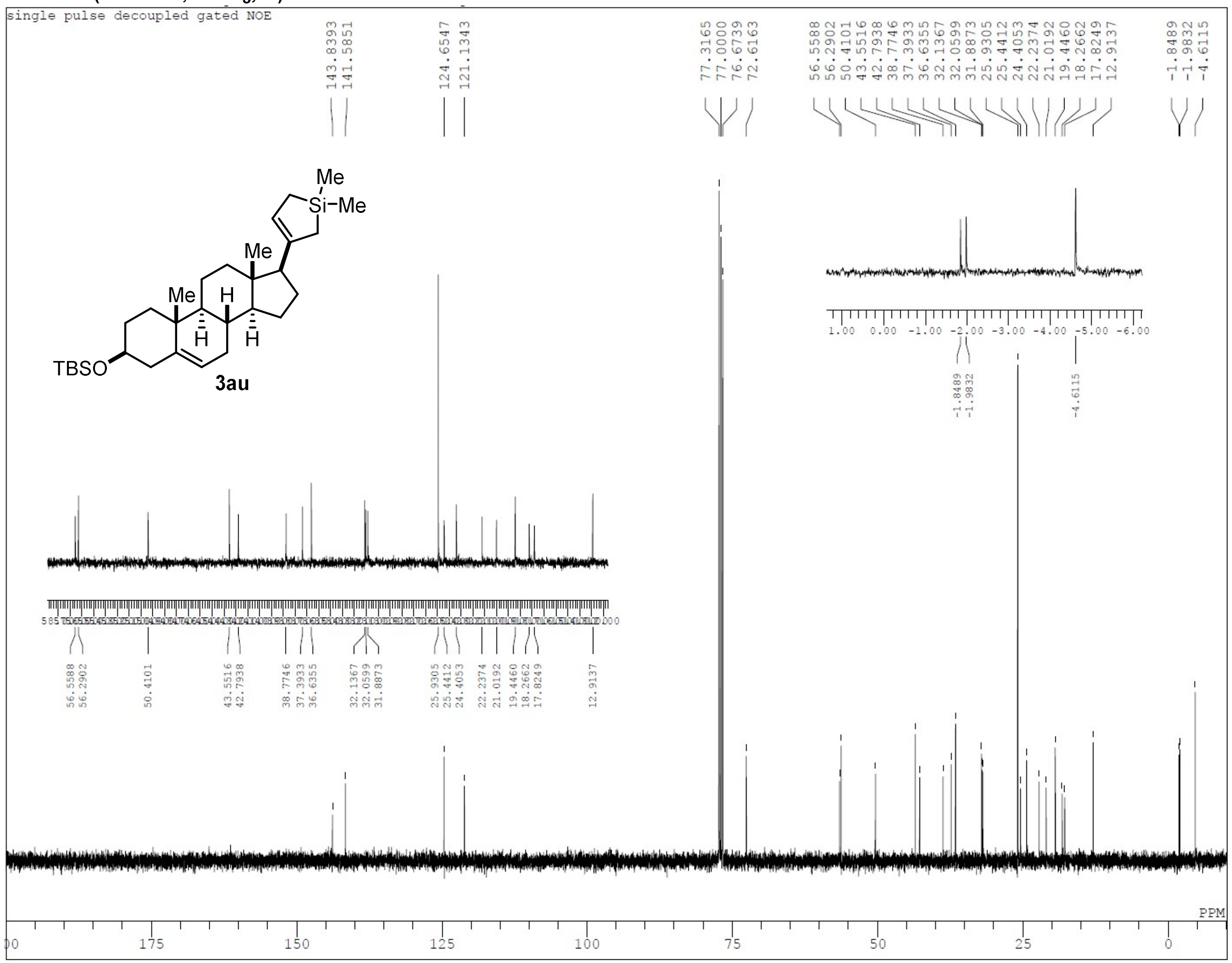

^{29}Si NMR (79 MHz, $CDCl_3$, rt)

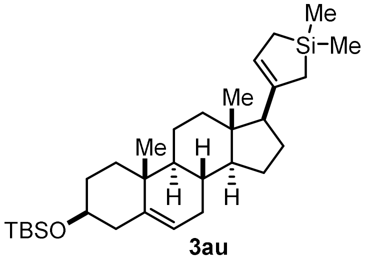

3au

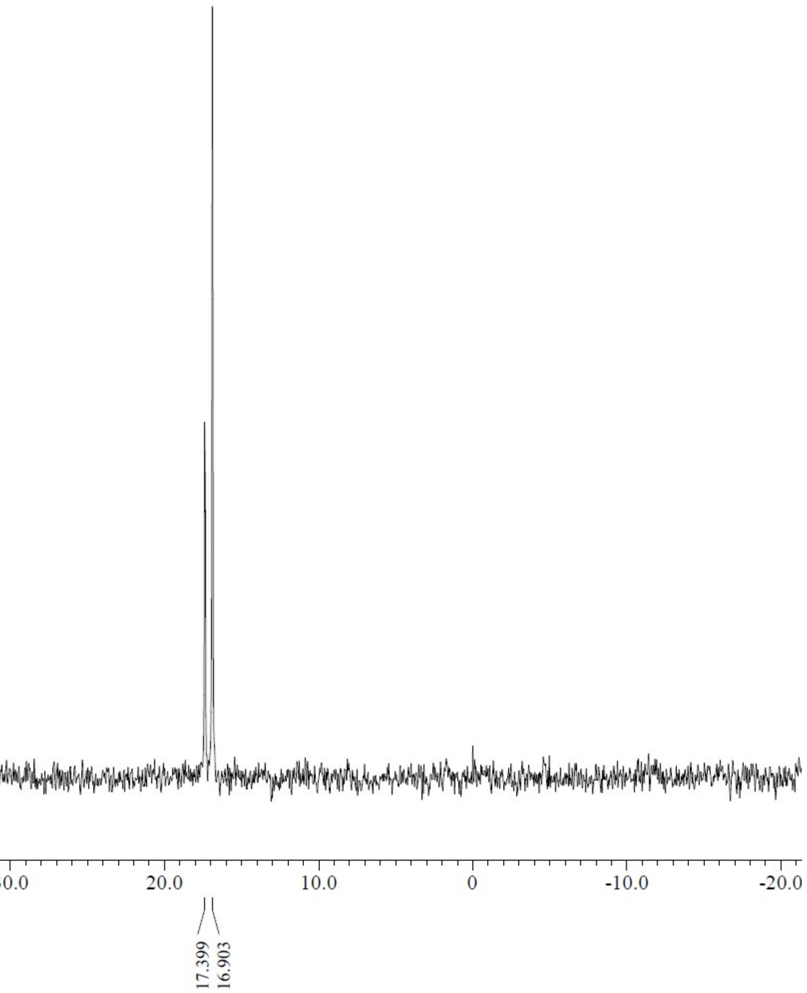

^{1}H NMR (400 MHz, $CDCl_3$, rt)

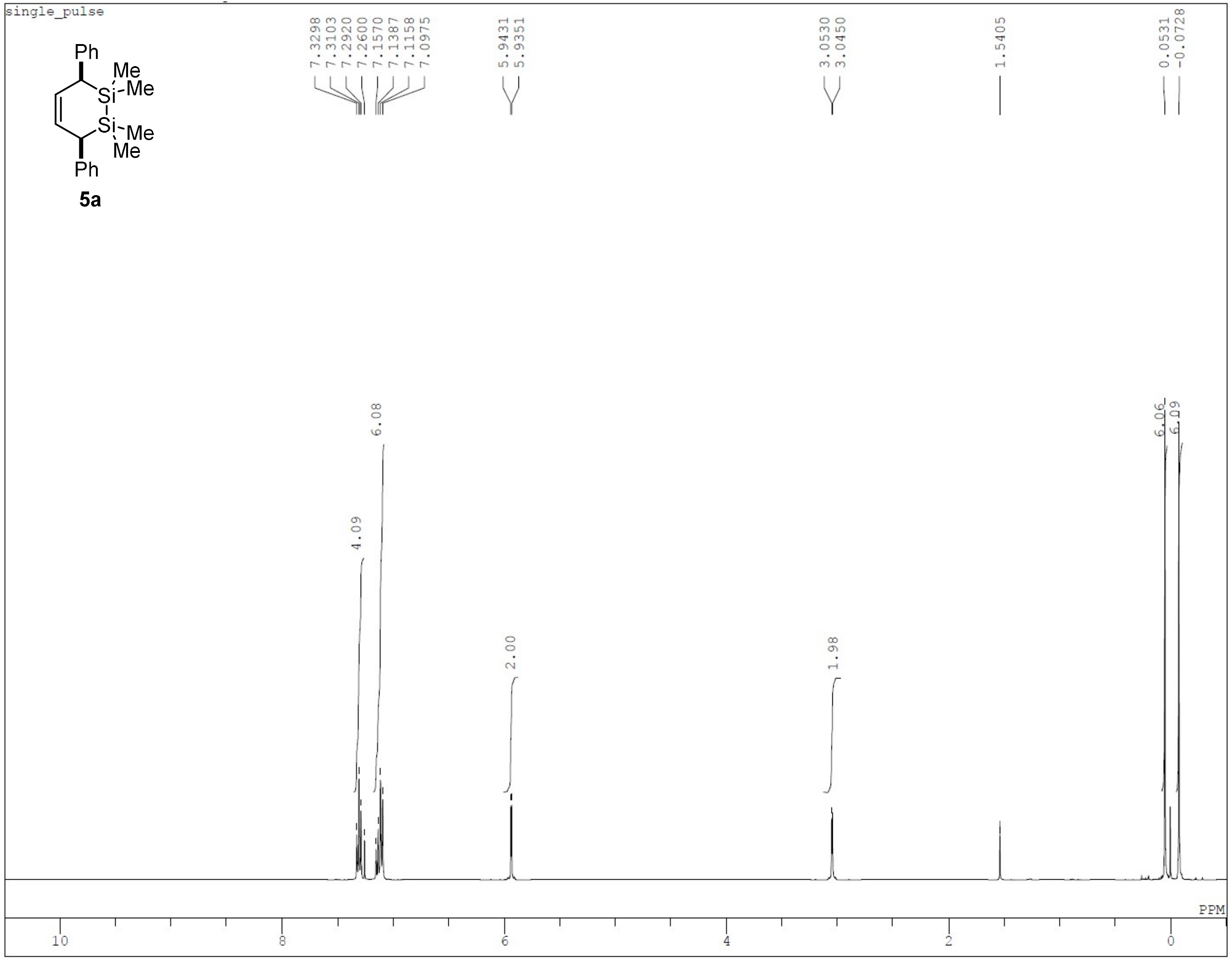

^{13}C NMR (100 MHz, $CDCl_3$, rt)

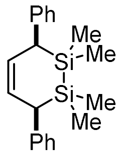

5a

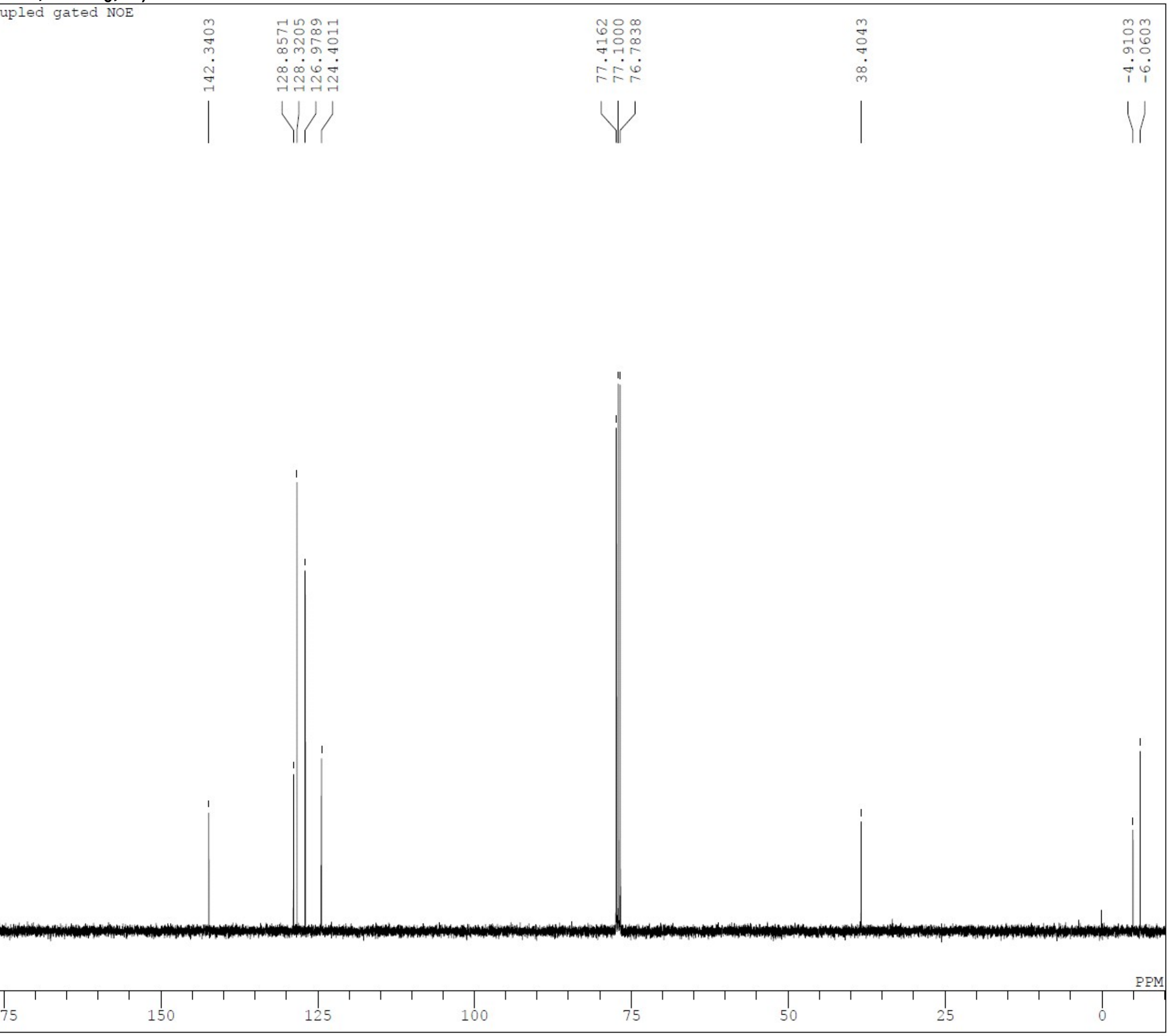

^{29}**Si NMR (79 MHz, $CDCl_3$, rt)**

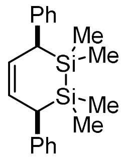

5a

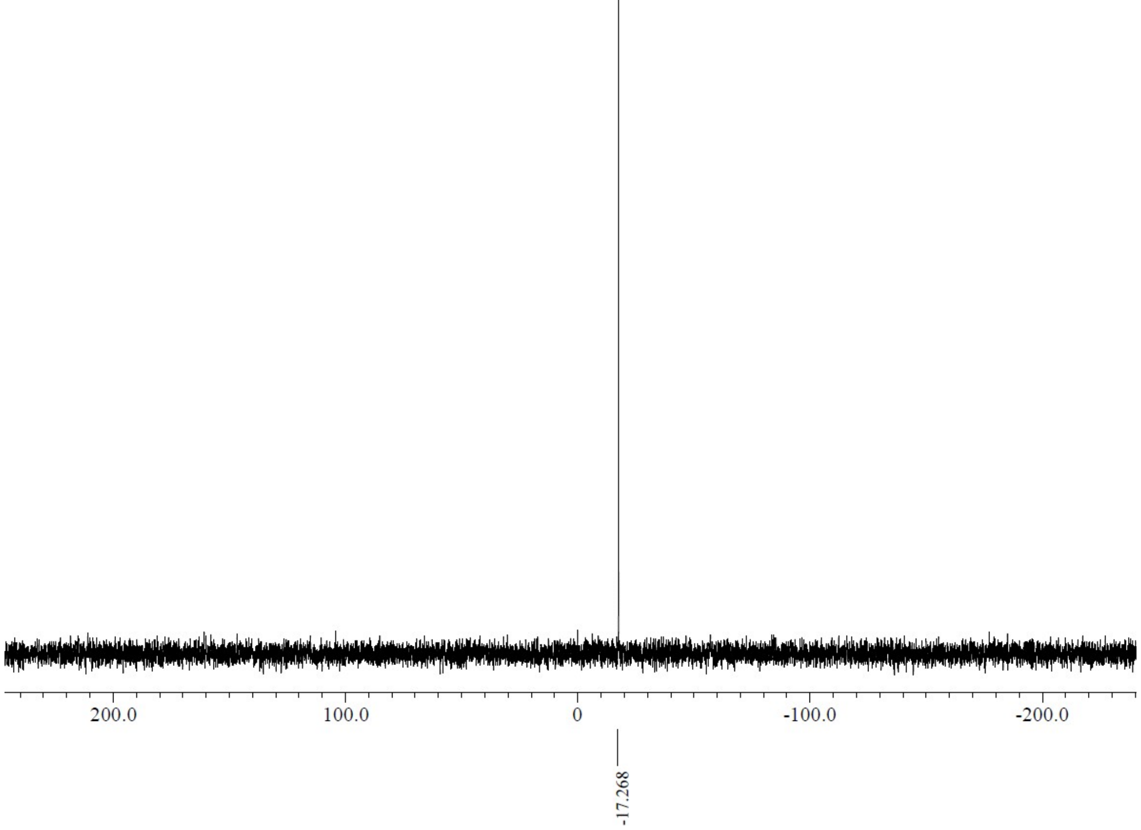

^{1}H NMR (400 MHz, $CDCl_3$, rt)

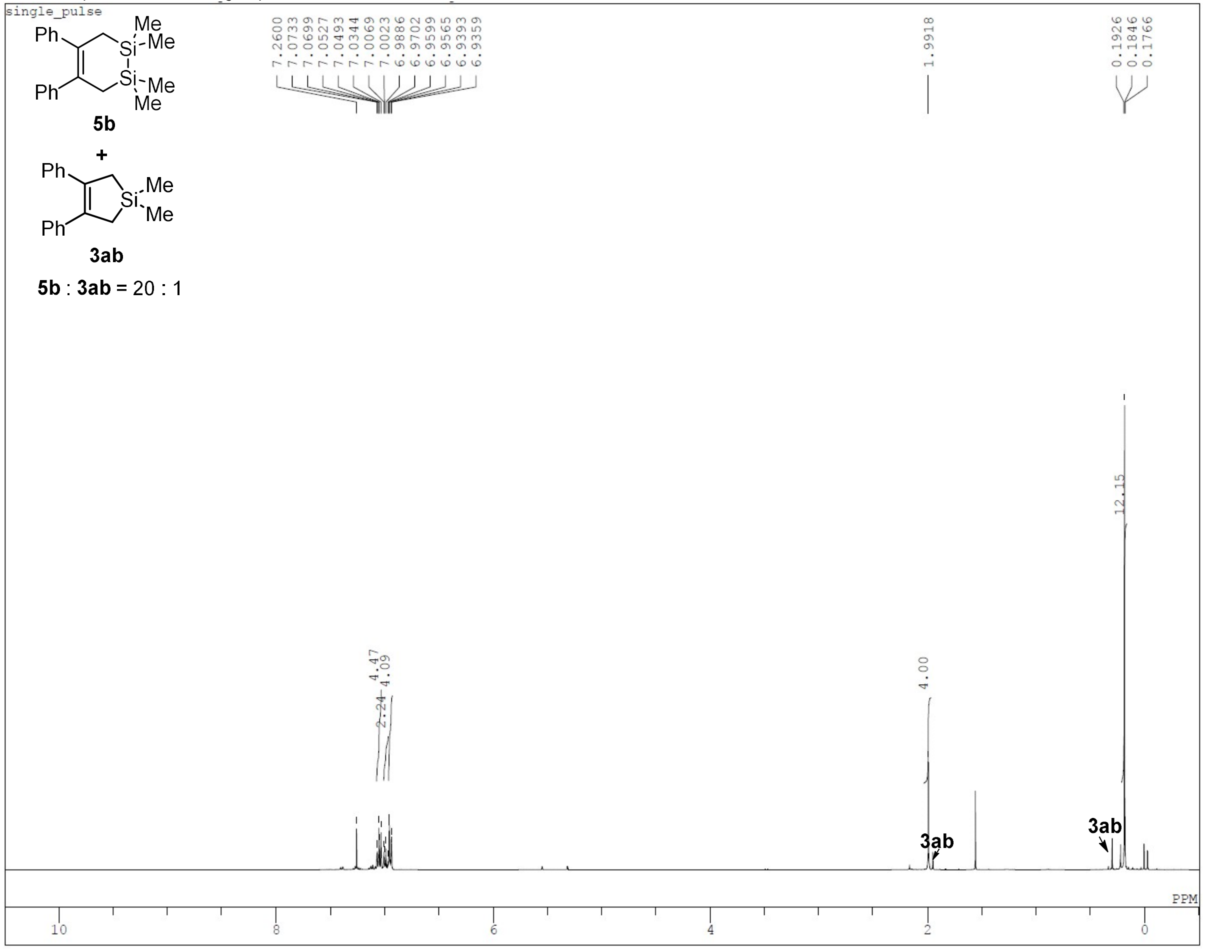

The product **5c** contains 5% **3ab** as byproduct.

^{13}C NMR (100 MHz, $CDCl_3$, rt)

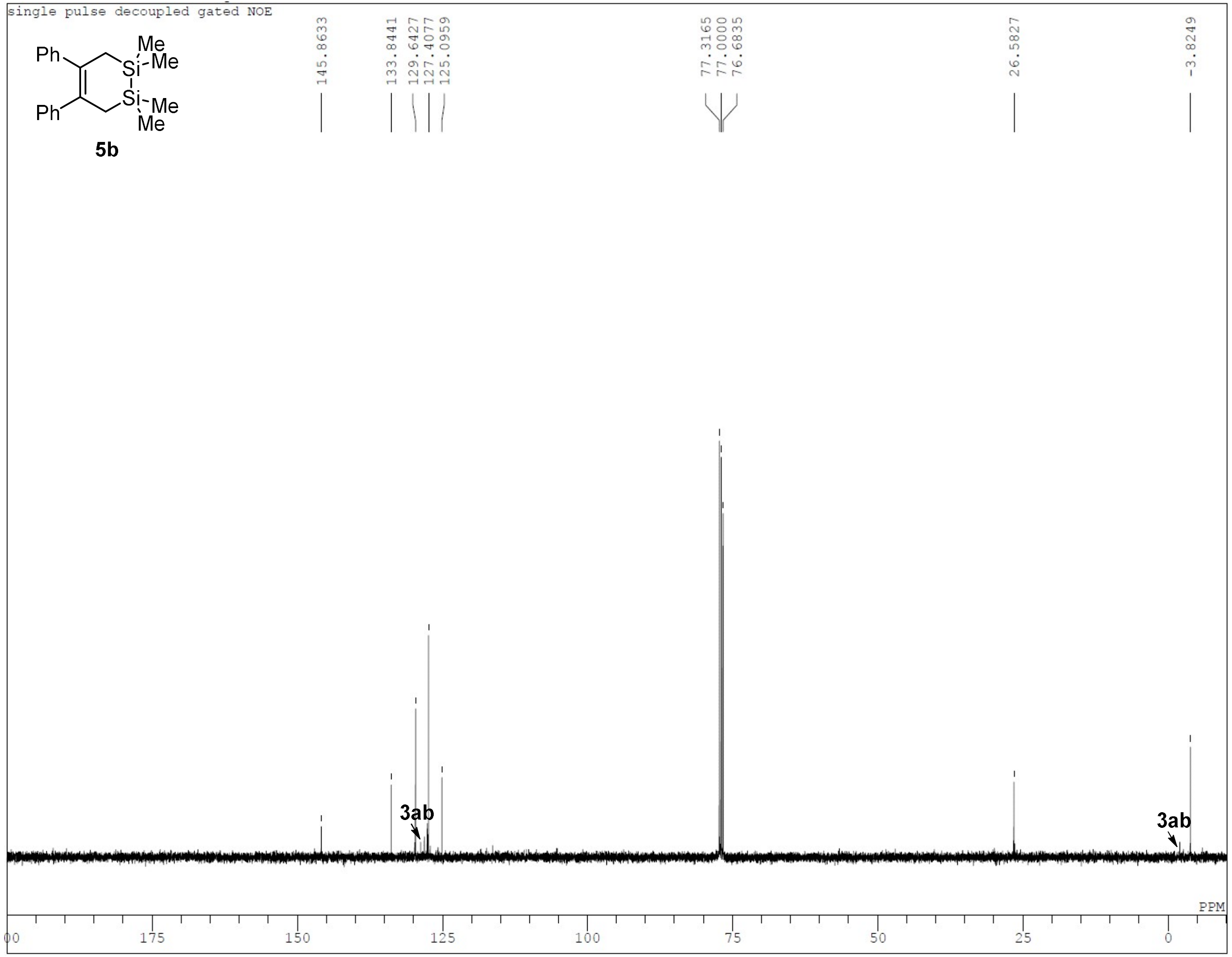

^{29}Si NMR (79 MHz, $CDCl_3$, rt)

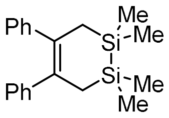

5b

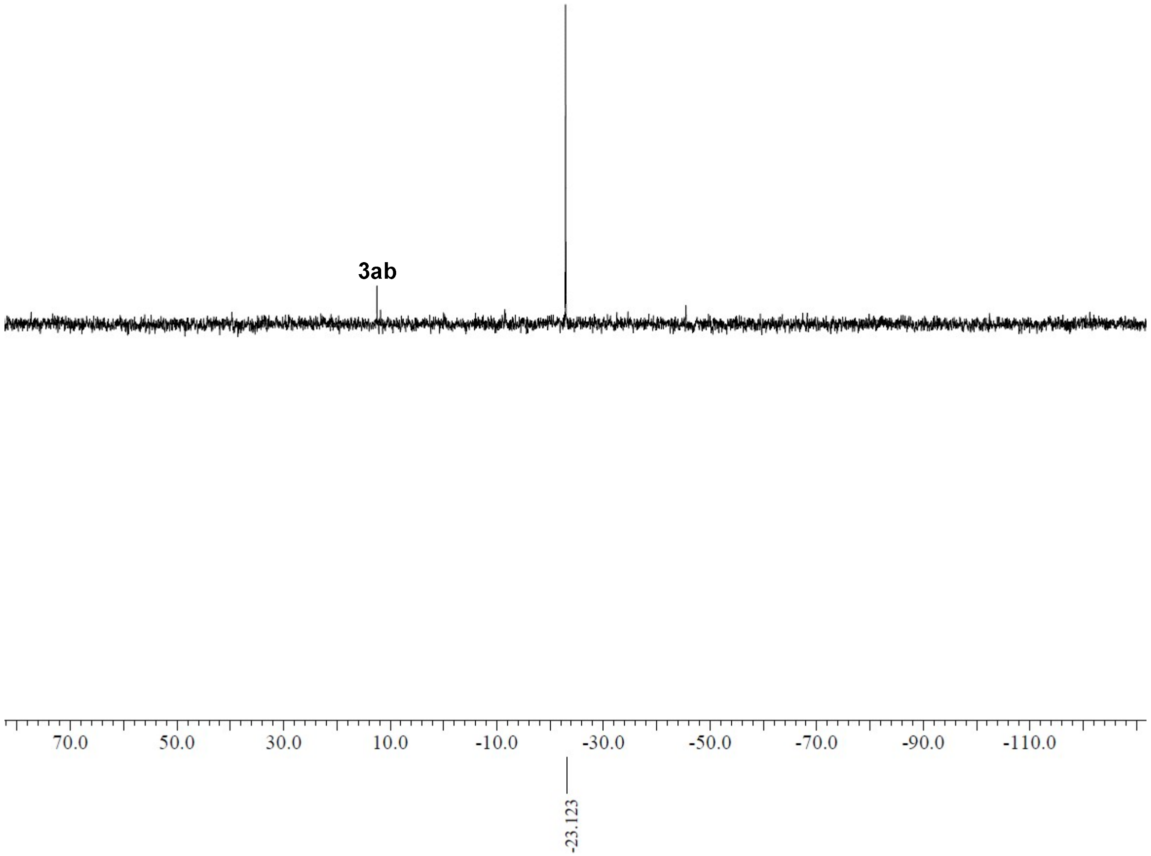

^{1}H NMR (400 MHz, $CDCl_3$, rt)

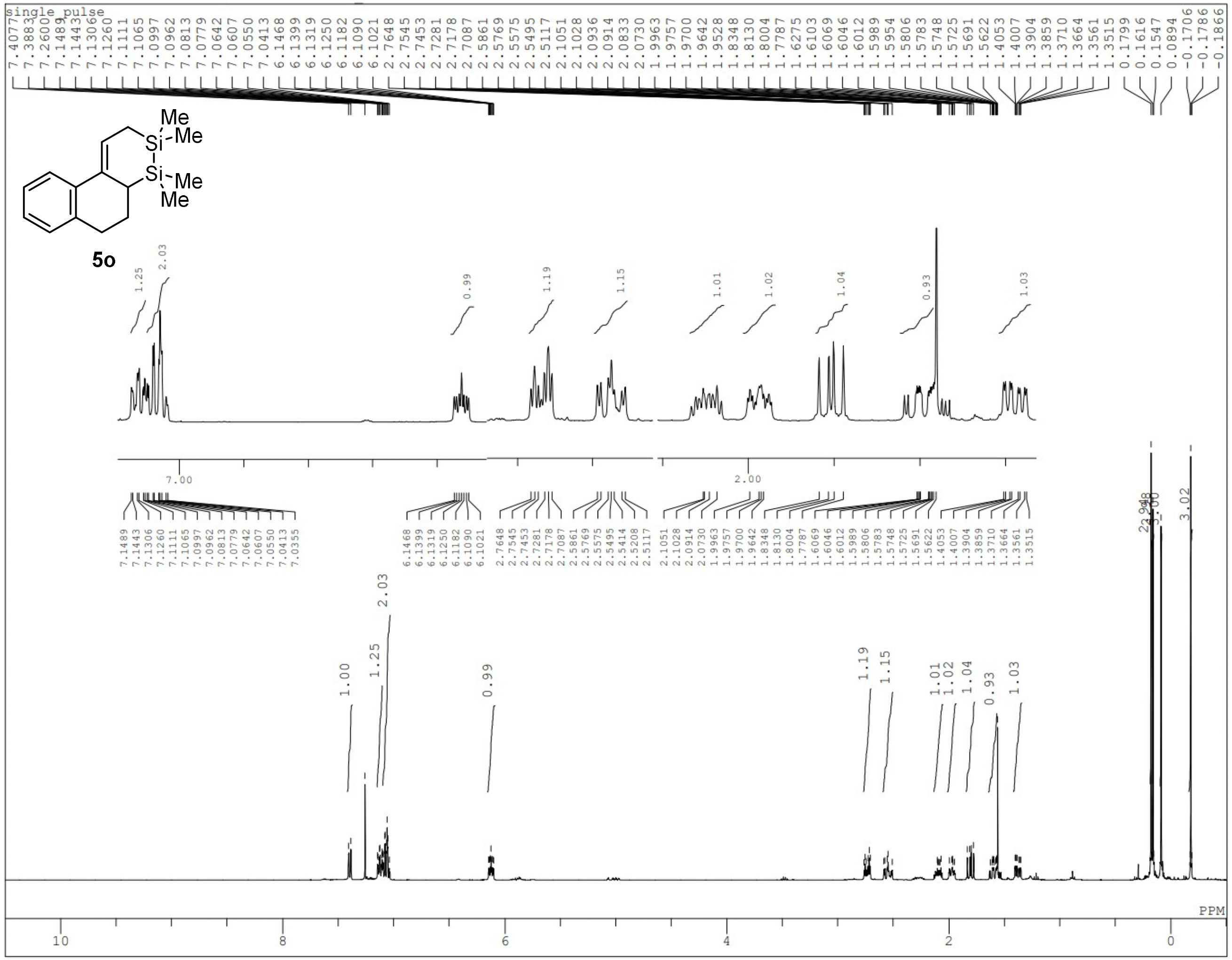

^{13}C NMR (100 MHz, CDCl$_3$, rt)

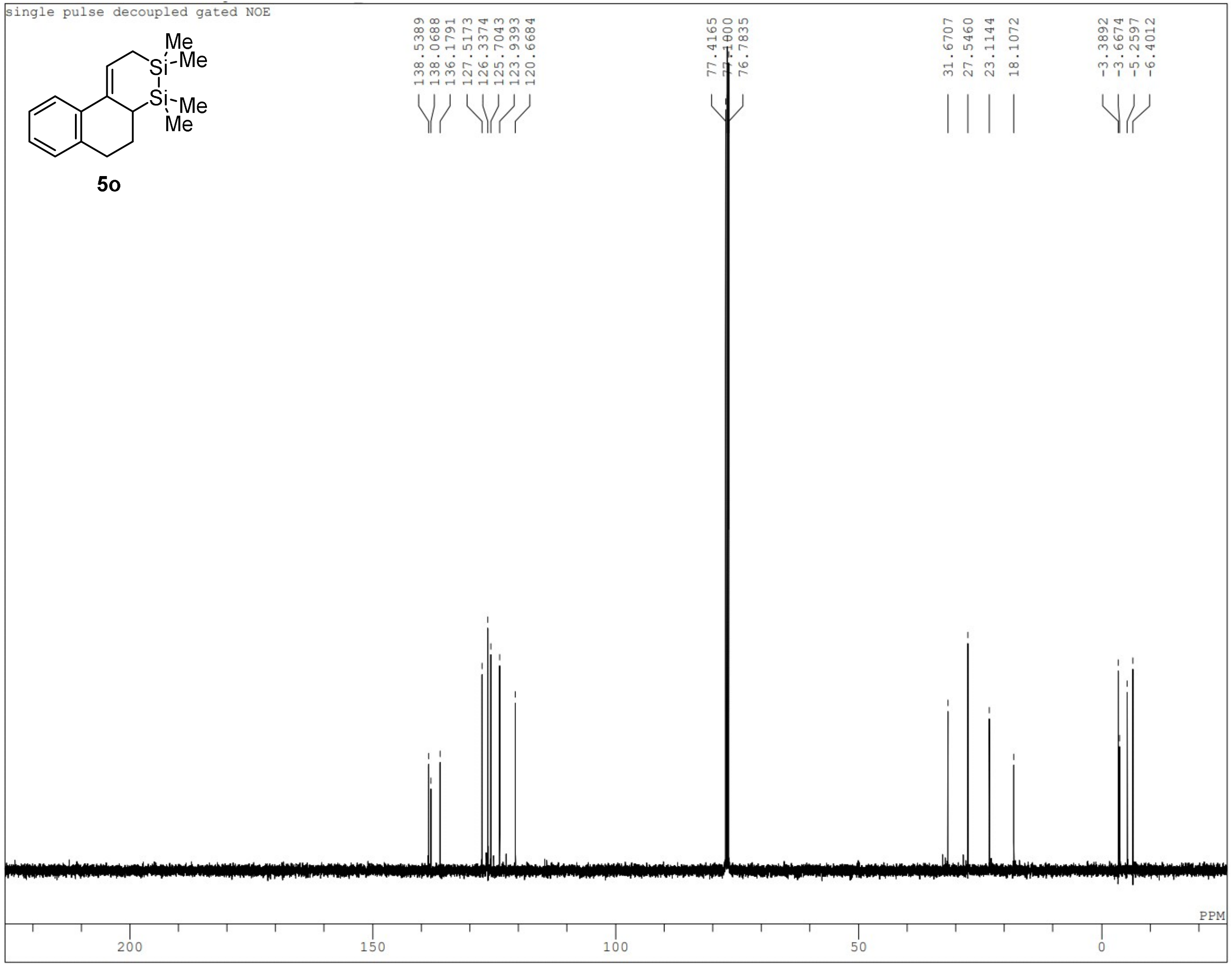

^{29}Si NMR (79 MHz, CDCl$_3$, rt)

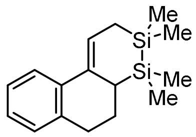

5o

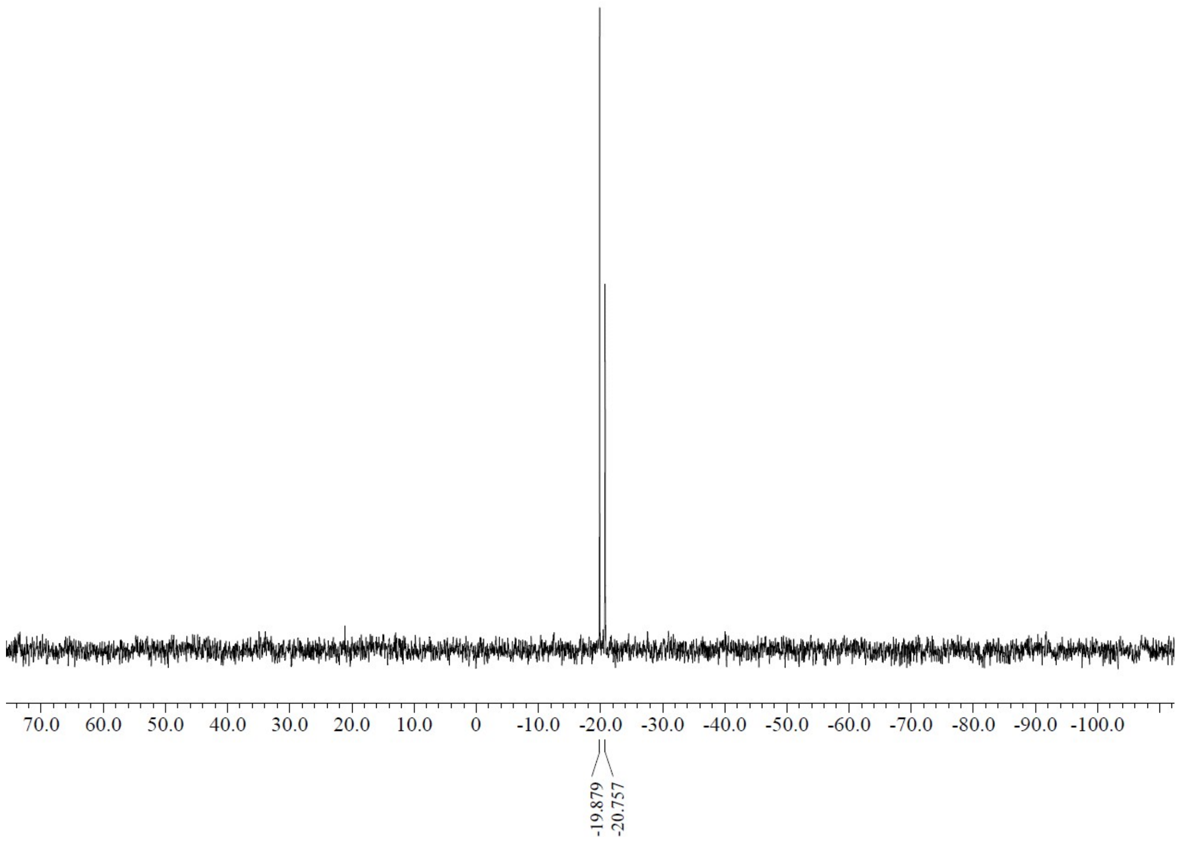

^{1}H NMR (400 MHz, $CDCl_3$, rt)

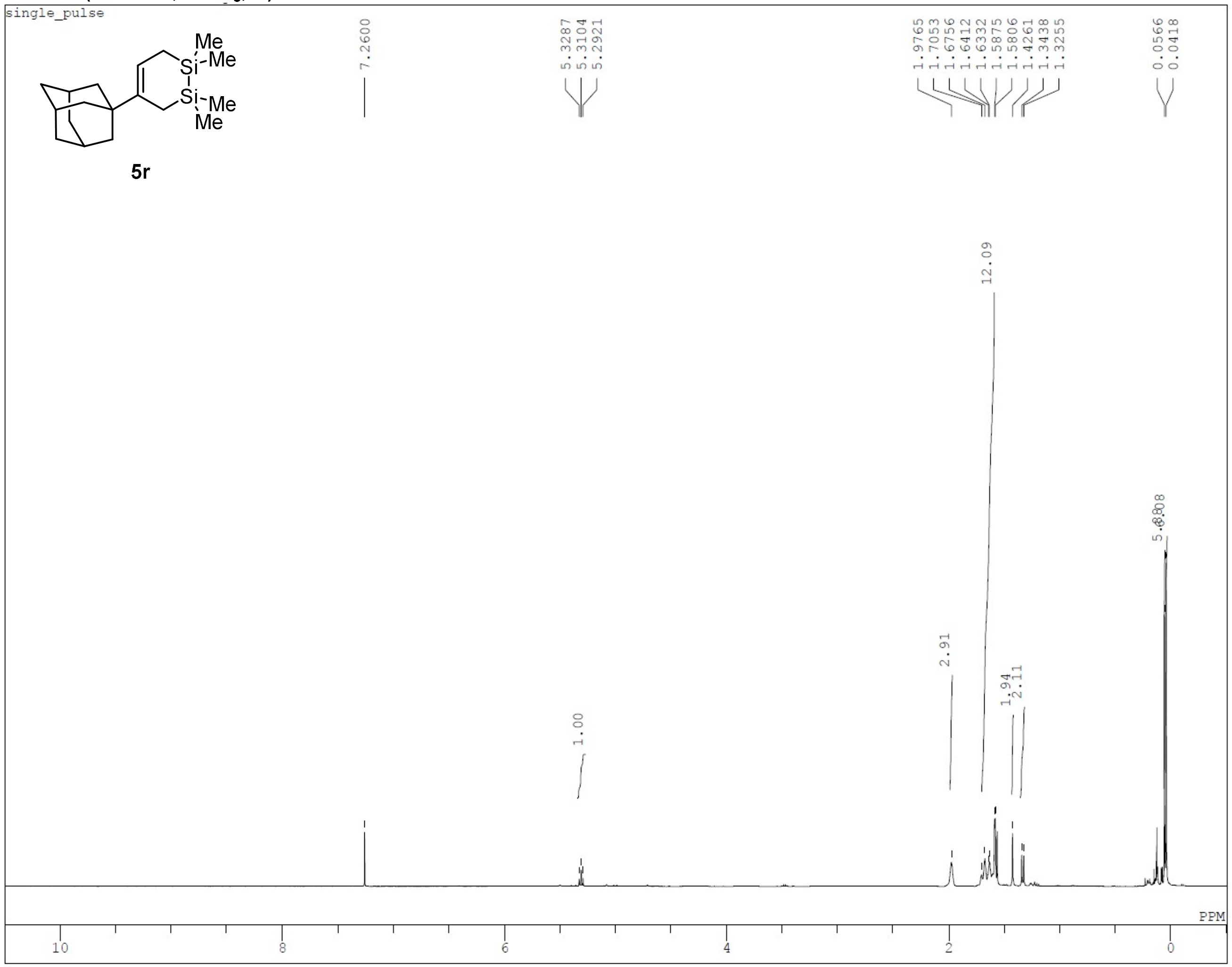

^{13}C NMR (100 MHz, $CDCl_3$, rt)

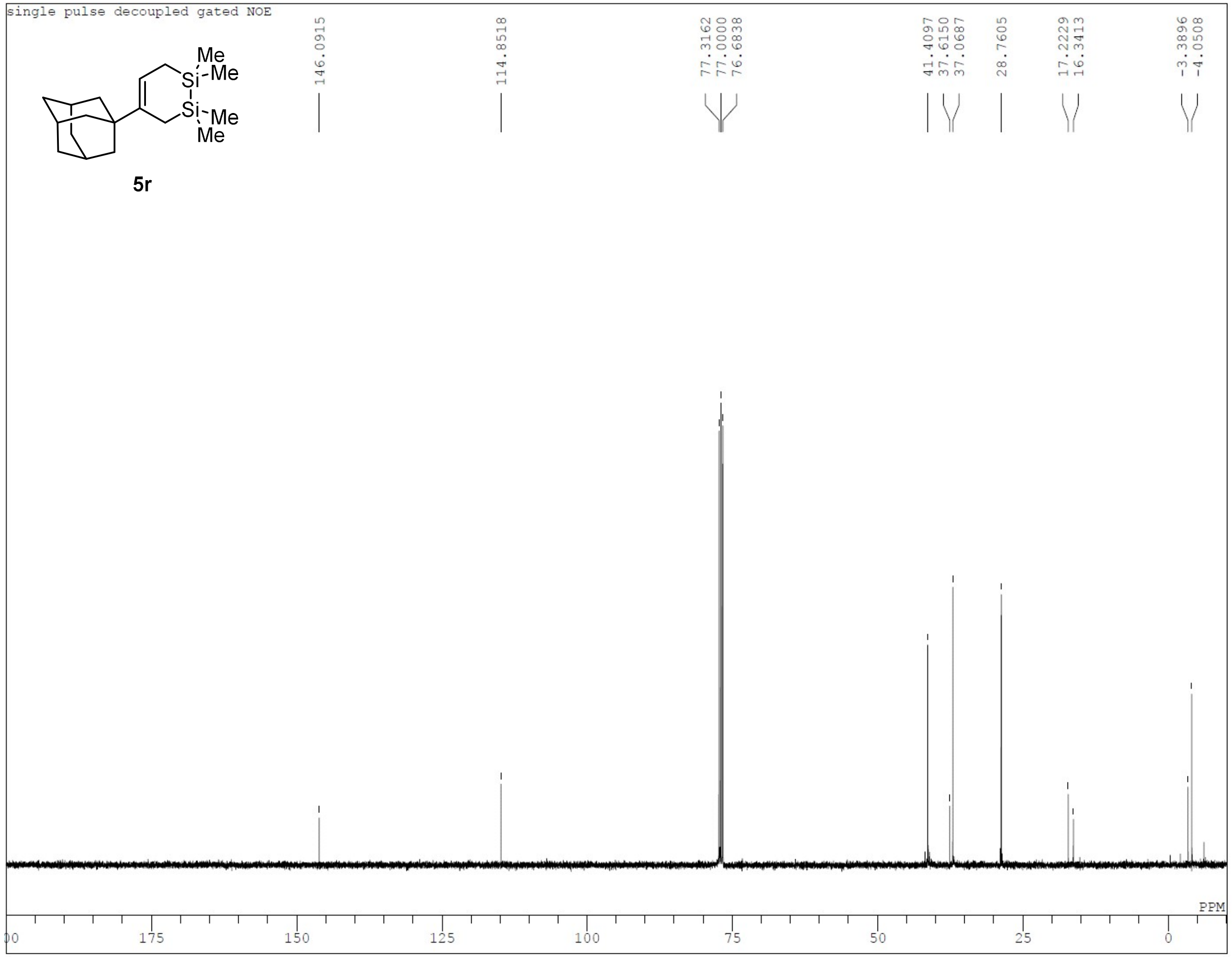

^{29}Si NMR (79 MHz, $CDCl_3$, rt)

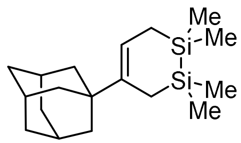

5r

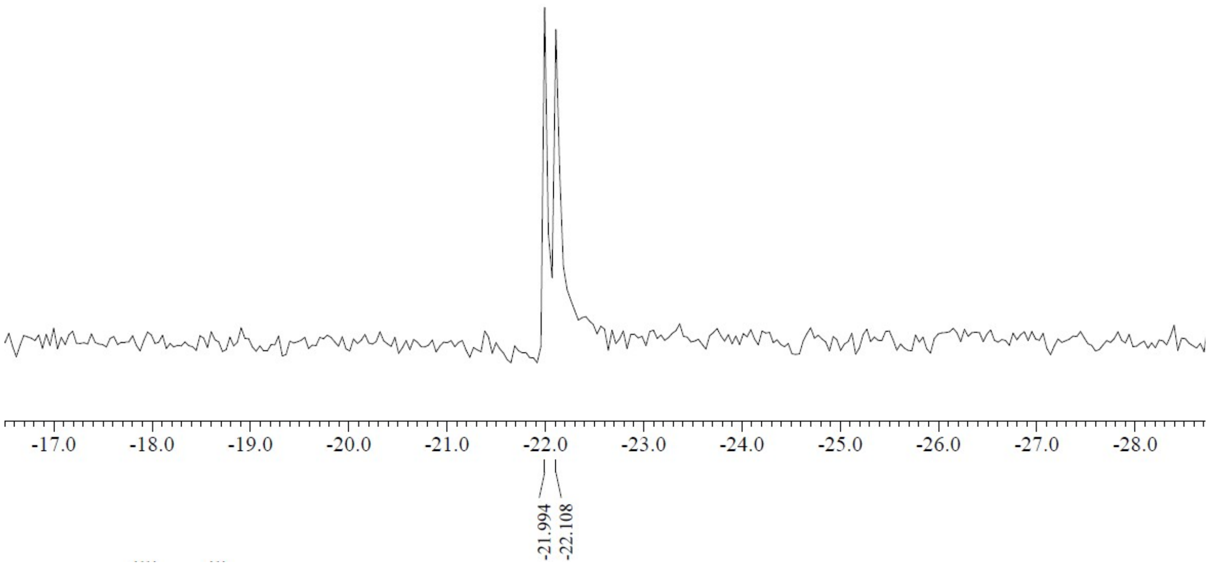

^1H NMR (400 MHz, $CDCl_3$, rt)

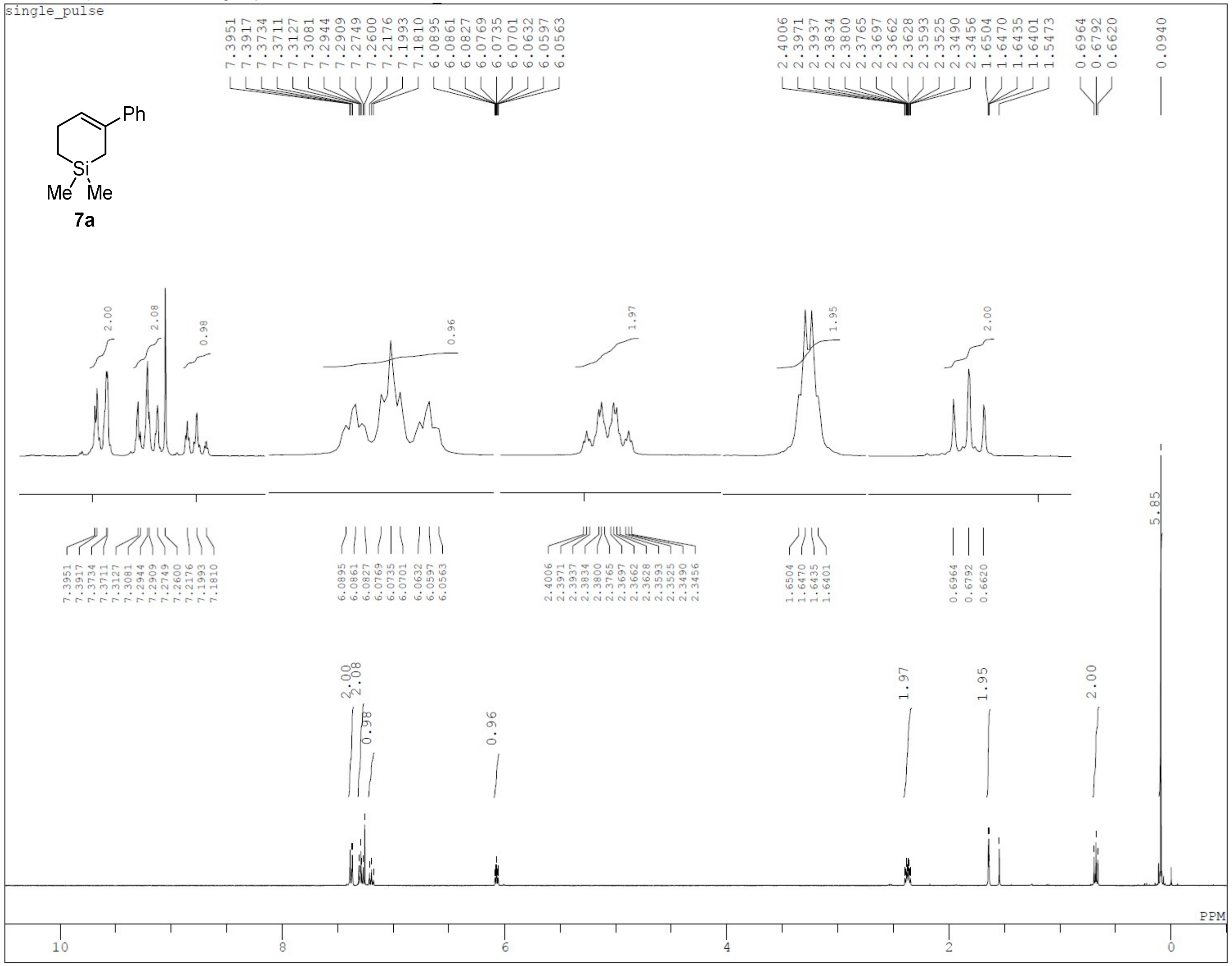

^{13}C NMR (100 MHz, $CDCl_3$, rt)

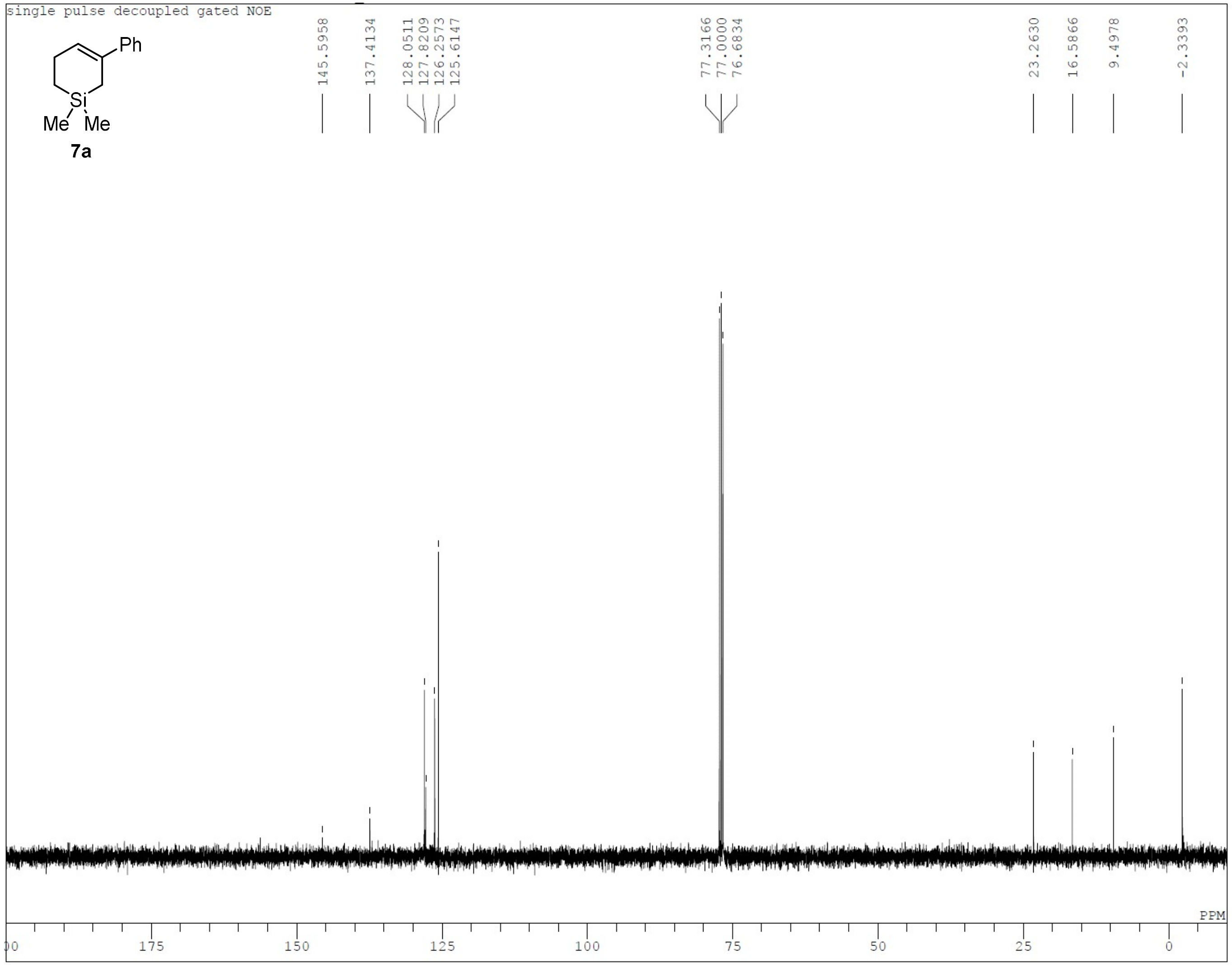

^{29}Si NMR (79 MHz, $CDCl_3$, rt)

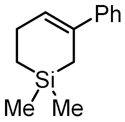

7a

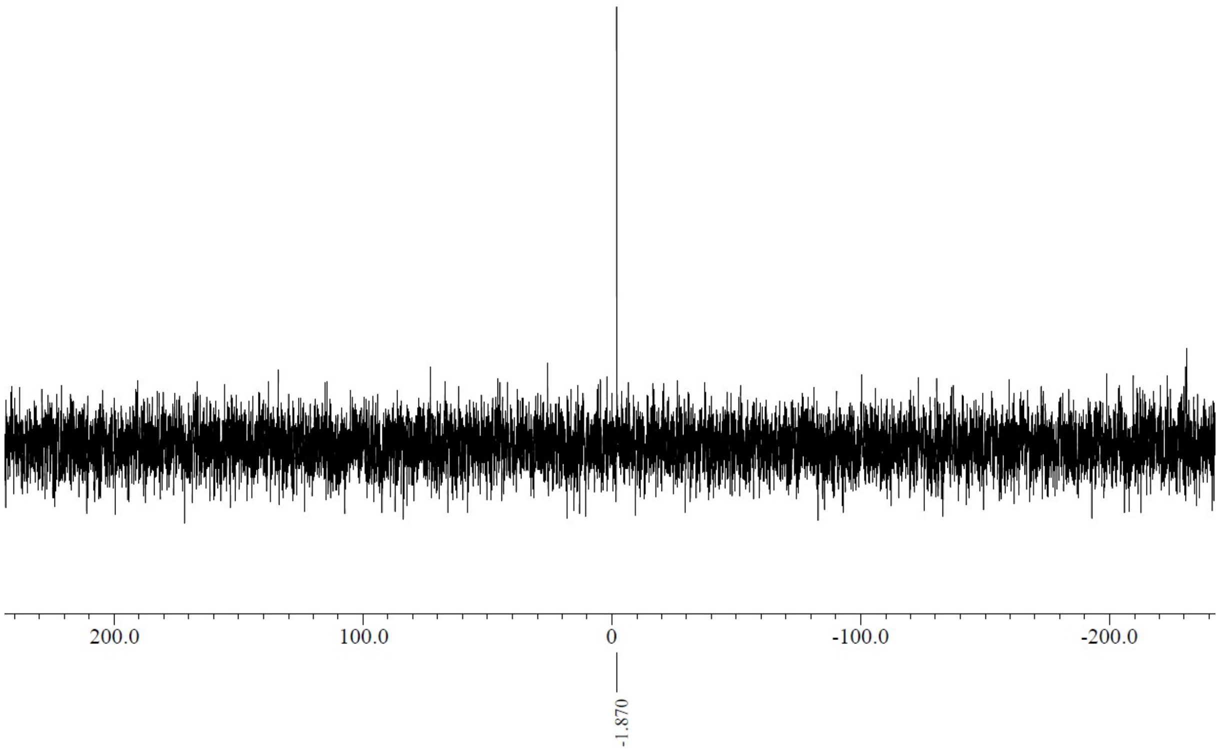

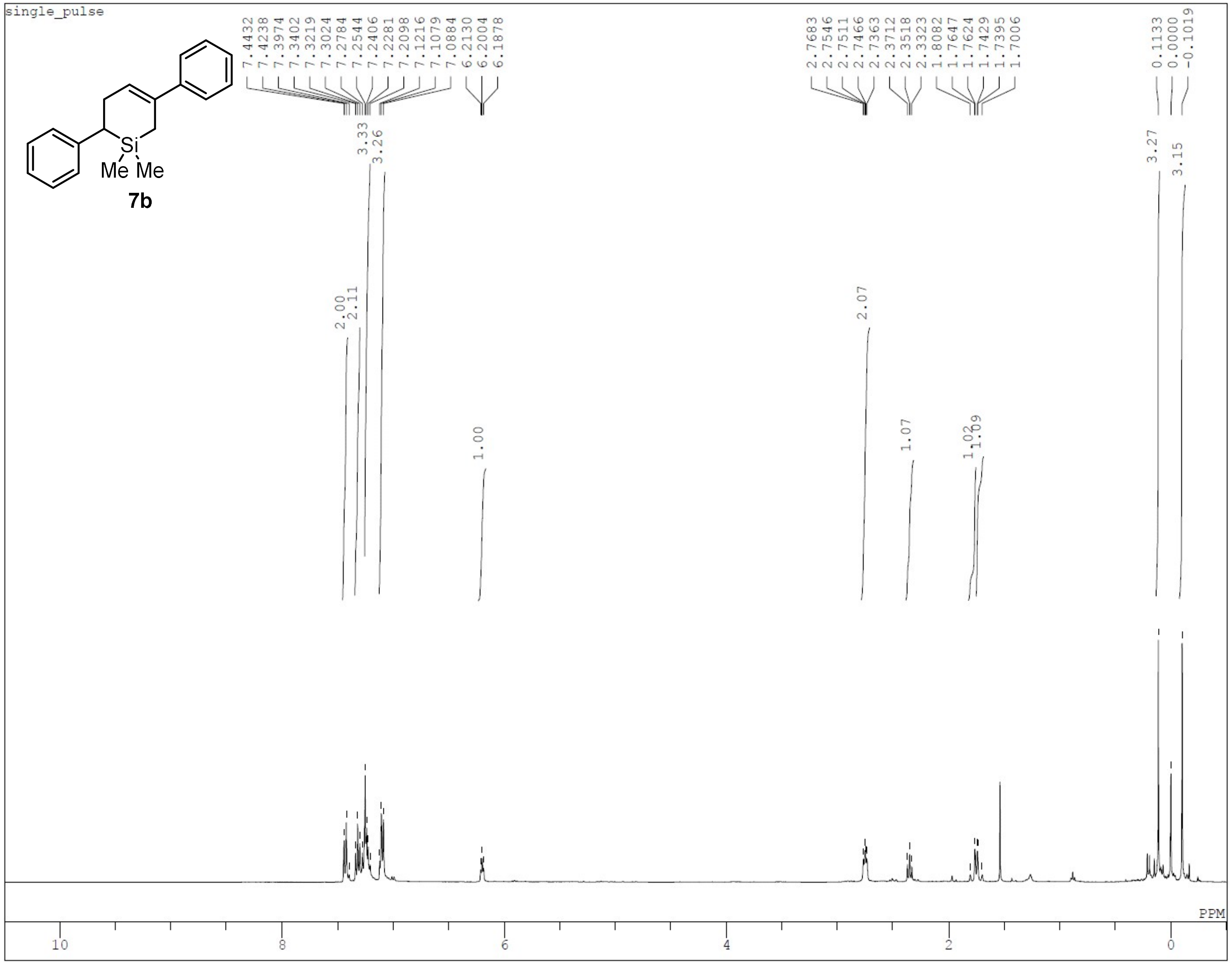
single_pulse
Si
Me Me
7b
7.4432
7.4238
7.3974
7.3402
7.3219
7.3024
7.2784
7.2544
7.2406
7.2281
7.2098
7.1216
7.1079
7.0884
6.2130
6.2004
6.1878
2.7683
2.7546
2.7511
2.7466
2.7363
2.3712
2.3518
2.3323
1.8082
1.7647
1.7624
1.7429
1.7395
1.7006
0.1133
0.0000
-0.1019
3.33
3.26
2.00
2.11
1.00
2.07
1.07
1.02
1.09
3.27
3.15
PPM
10
8
6
4
2
0

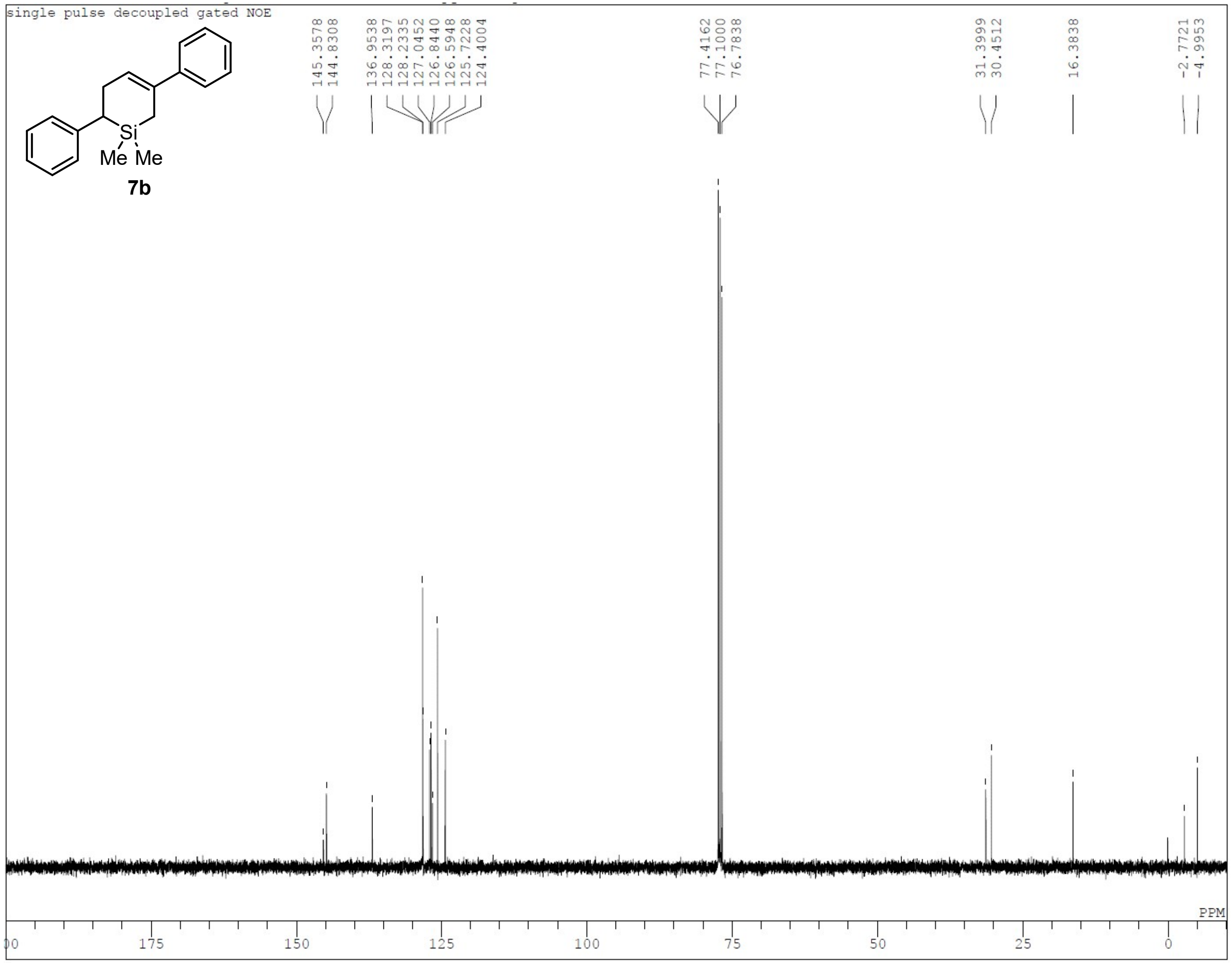
single pulse decoupled gated NOE
Si
Me Me
7b
145.3578
144.8308
136.9538
128.3197
128.2335
127.0452
126.8440
126.5948
125.7228
124.4004
77.4162
77.1000
76.7838
31.3999
30.4512
16.3838
-2.7721
-4.9953
PPM
175
150
125
100
75
50
25
0

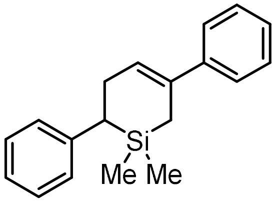

7b

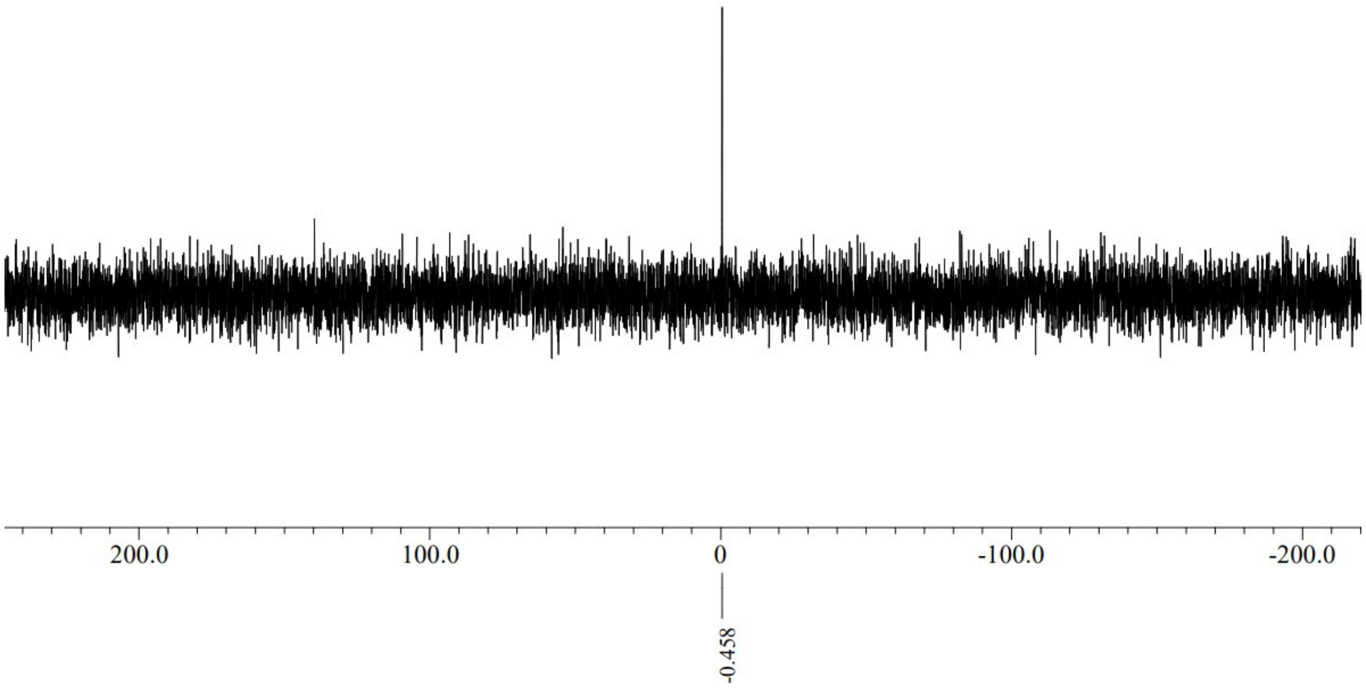

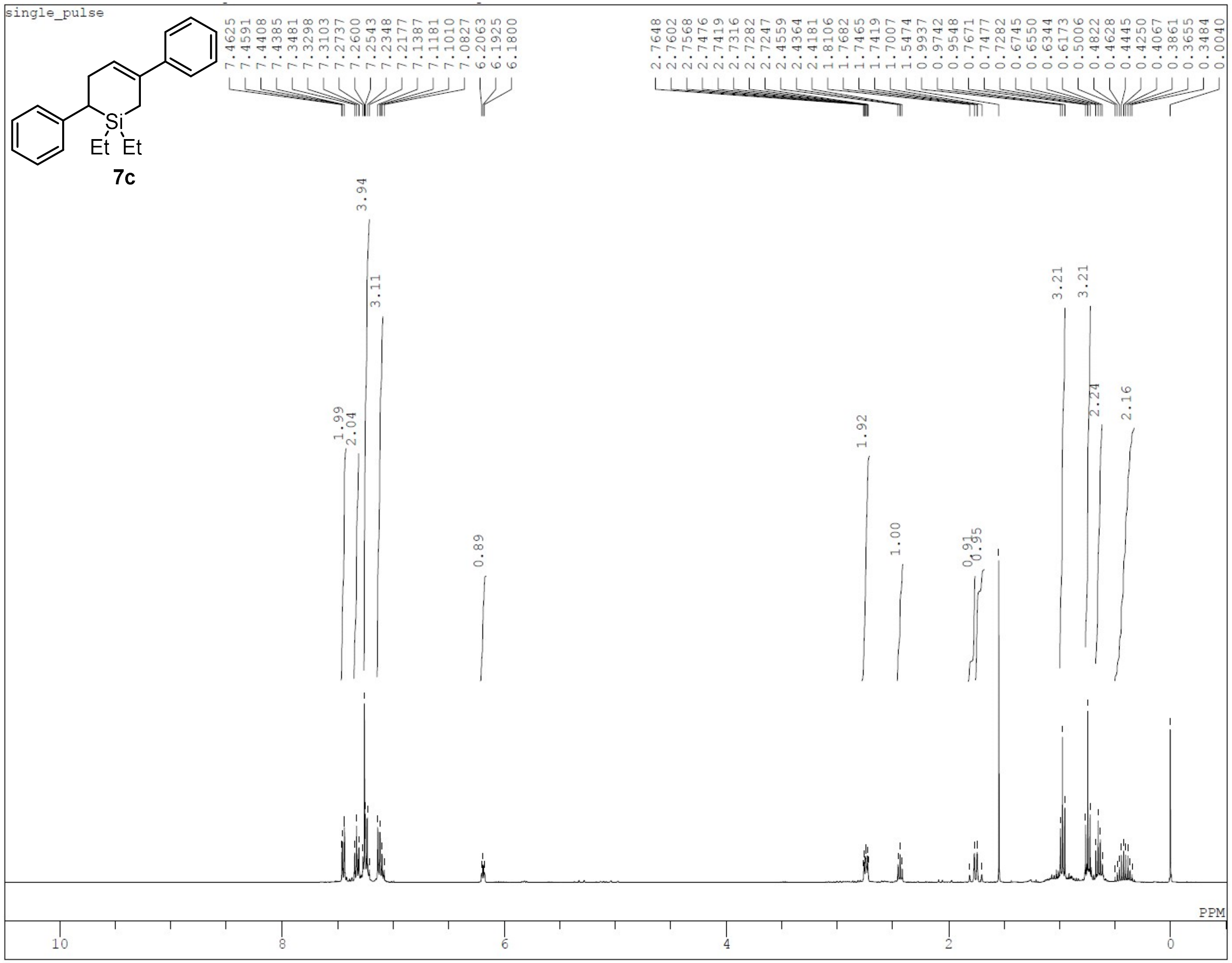

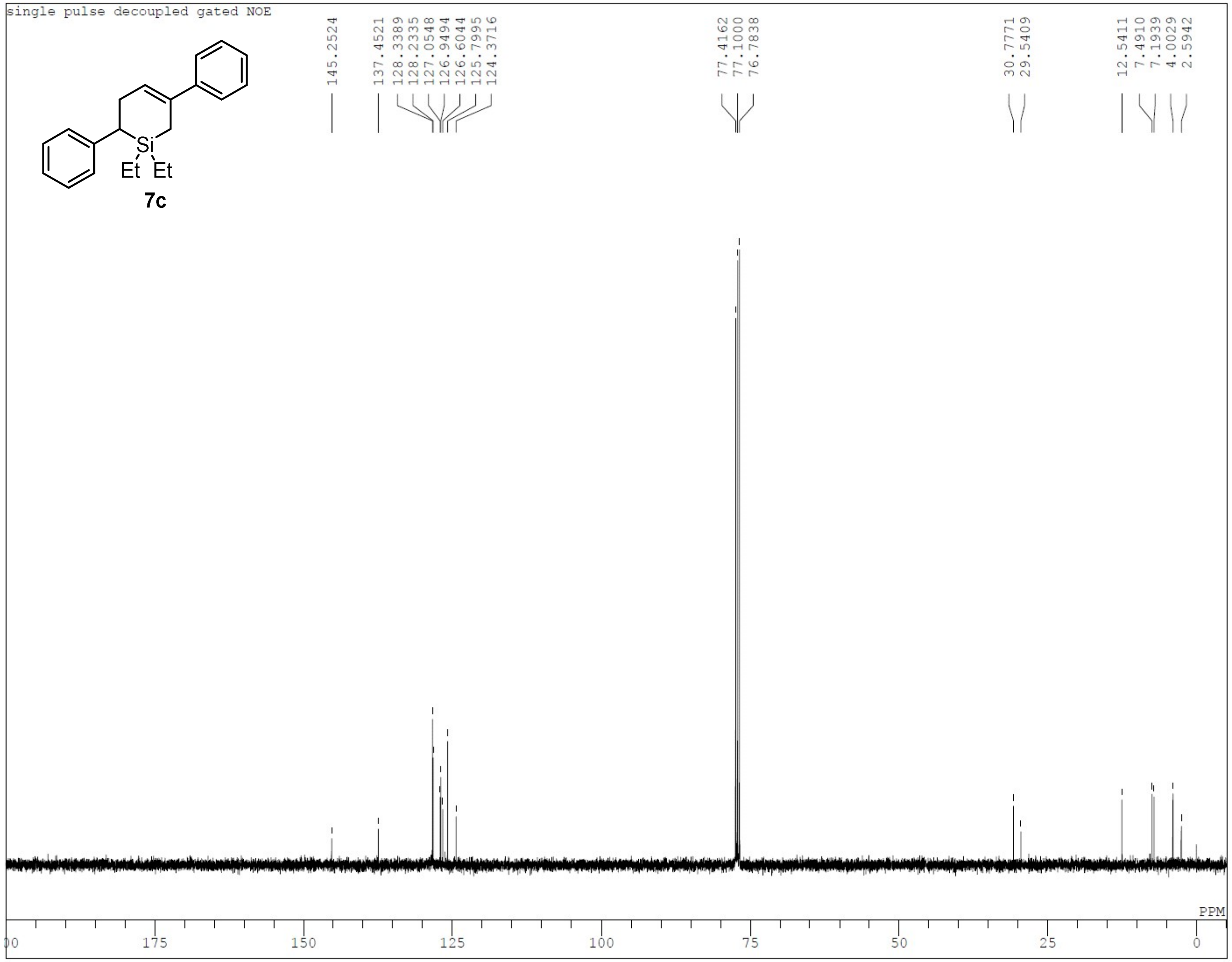
single pulse decoupled gated NOE
Si
Et
Et
7c
145.2524
137.4521
128.3389
128.2335
127.0548
126.9494
126.6044
125.7995
124.3716
77.4162
77.1000
76.7838
30.7771
29.5409
12.5411
7.4910
7.1939
4.0029
2.5942
PPM
175
150
125
100
75
50
25
0

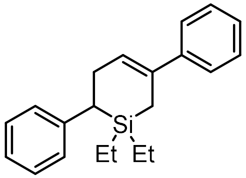

7c

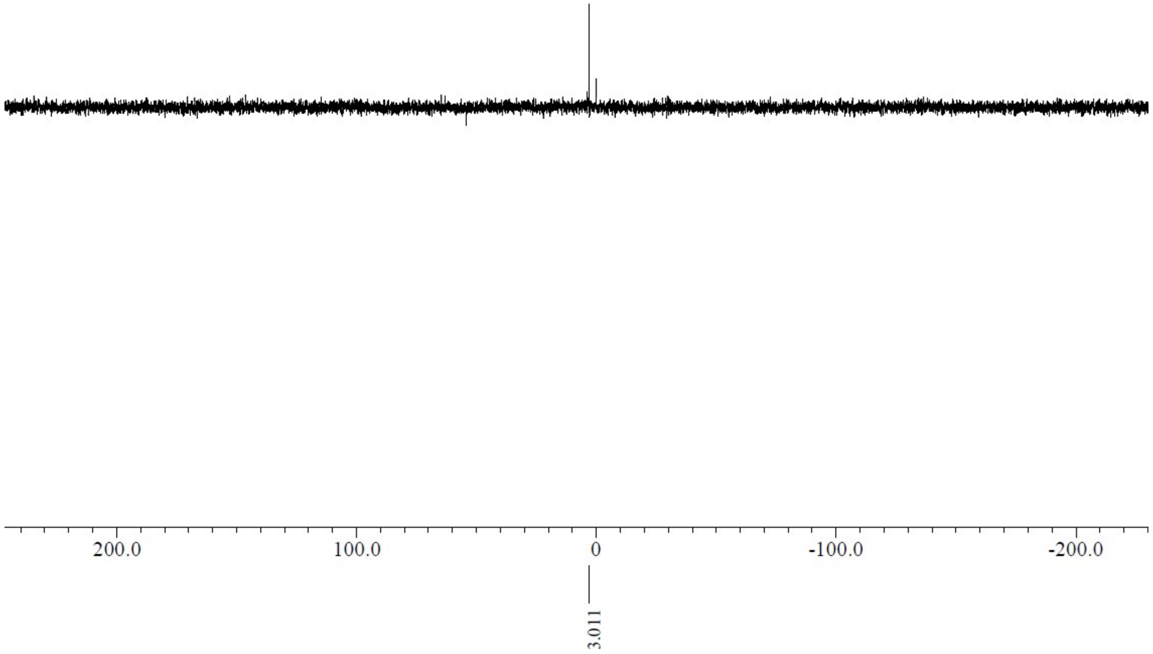

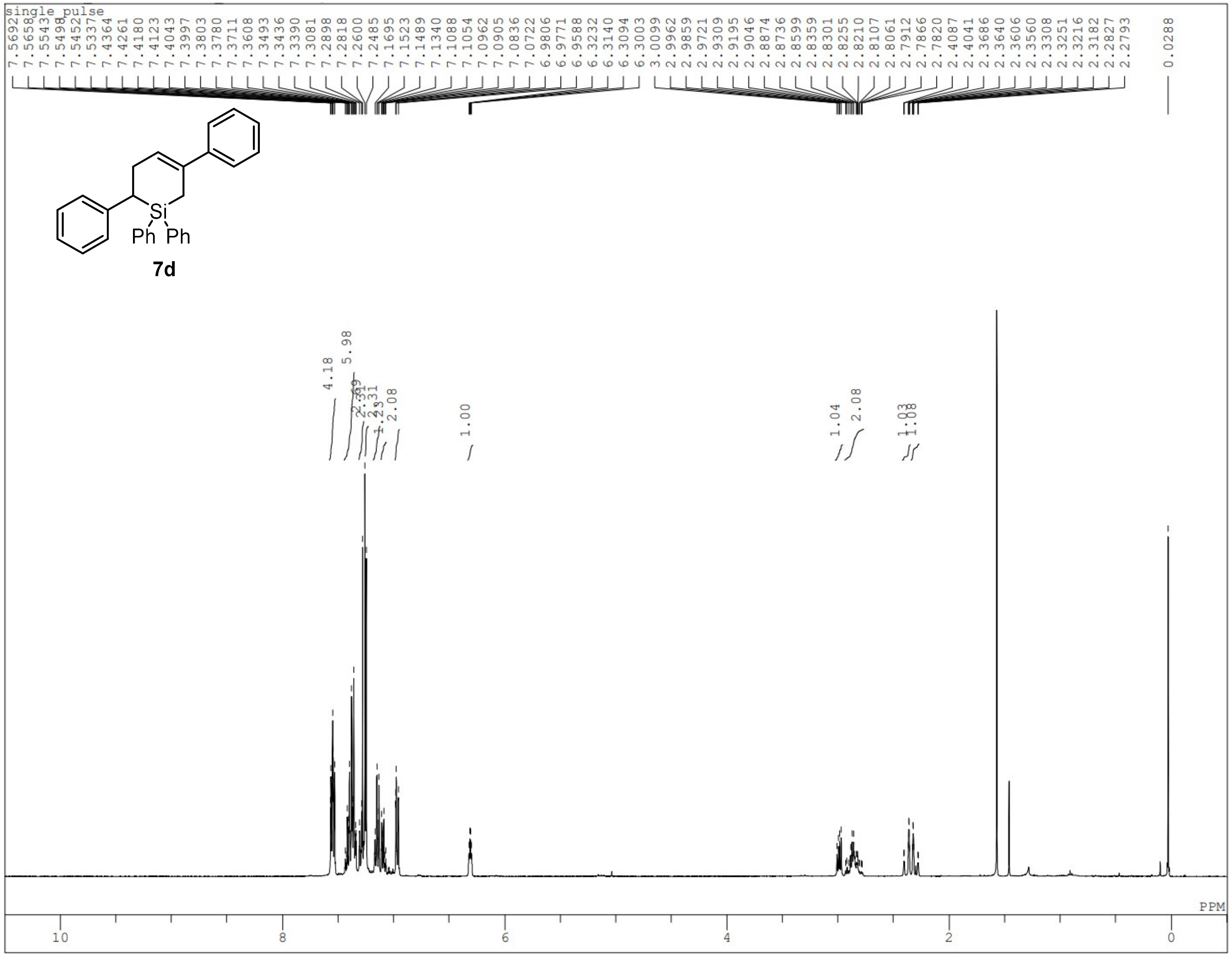

single pulse
7.5692
7.5658
7.5543
7.5498
7.5452
7.5337
7.4364
7.4261
7.4180
7.4123
7.4043
7.3997
7.3803
7.3780
7.3711
7.3608
7.3493
7.3436
7.3390
7.3081
7.2898
7.2818
7.2600
7.2485
7.1695
7.1523
7.1489
7.1340
7.1088
7.1054
7.0962
7.0905
7.0836
7.0722
6.9806
6.9771
6.9588
6.3232
6.3140
6.3094
6.3003
3.0099
2.9962
2.9859
2.9721
2.9309
2.9195
2.9046
2.8874
2.8736
2.8599
2.8359
2.8301
2.8255
2.8210
2.8107
2.8061
2.7912
2.7866
2.7820
2.4087
2.4041
2.3686
2.3640
2.3606
2.3560
2.3308
2.3251
2.3216
2.3182
2.2827
2.2793
0.0288
Si
Ph Ph
7d
4.18
5.98
2.31
2.08
1.00
1.04
2.08
1.03
1.08
PPM
10
8
6
4
2
0

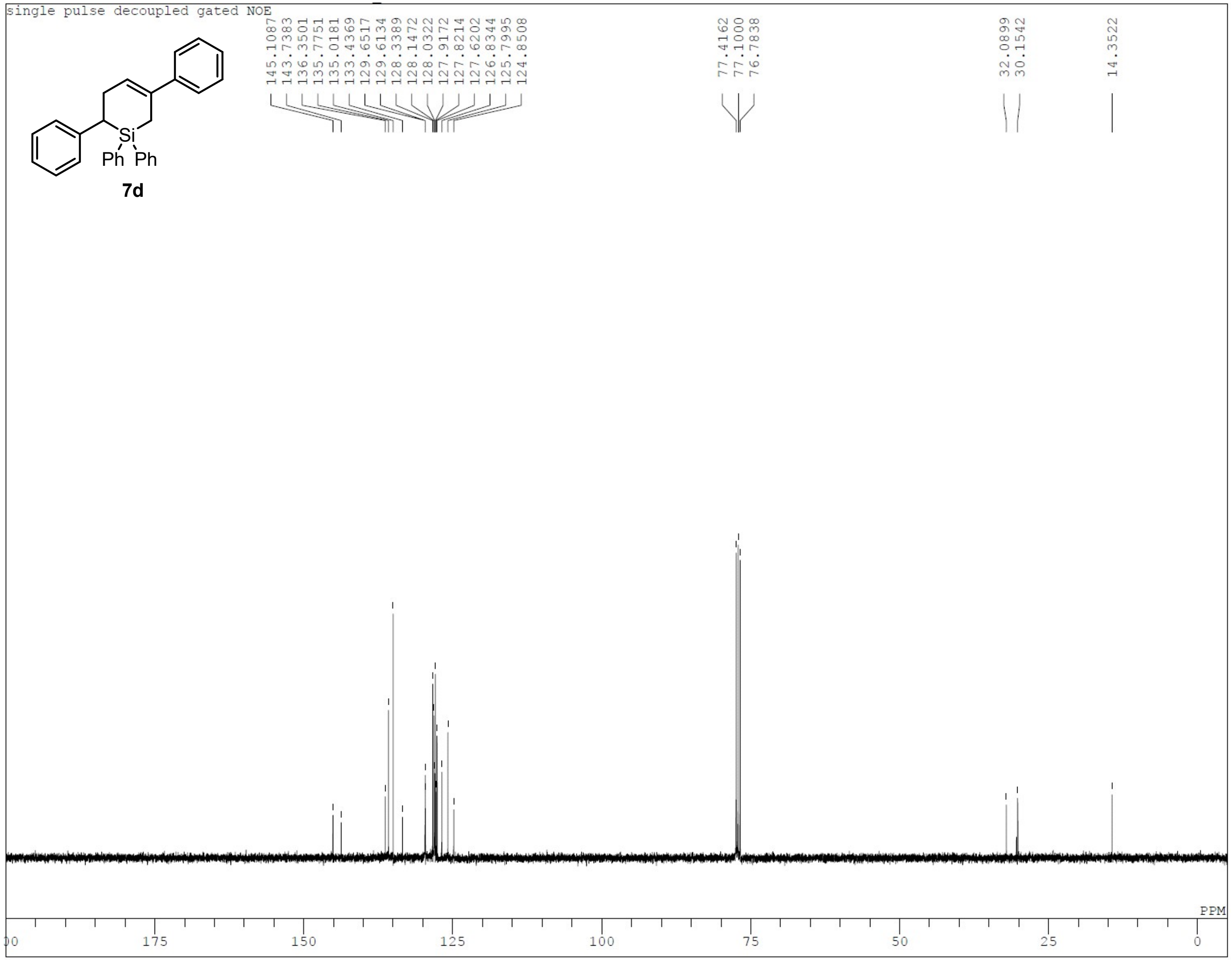
single pulse decoupled gated NOE
145.1087
143.7383
136.3501
135.7751
135.0181
133.4369
129.6517
129.6134
128.3389
128.1472
128.0322
127.9172
127.8214
127.6202
126.8344
125.7995
124.8508
77.4162
77.1000
76.7838
32.0899
30.1542
14.3522
Si
Ph
Ph
7d
PPM
175
150
125
100
75
50
25
0

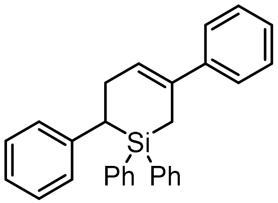

7d

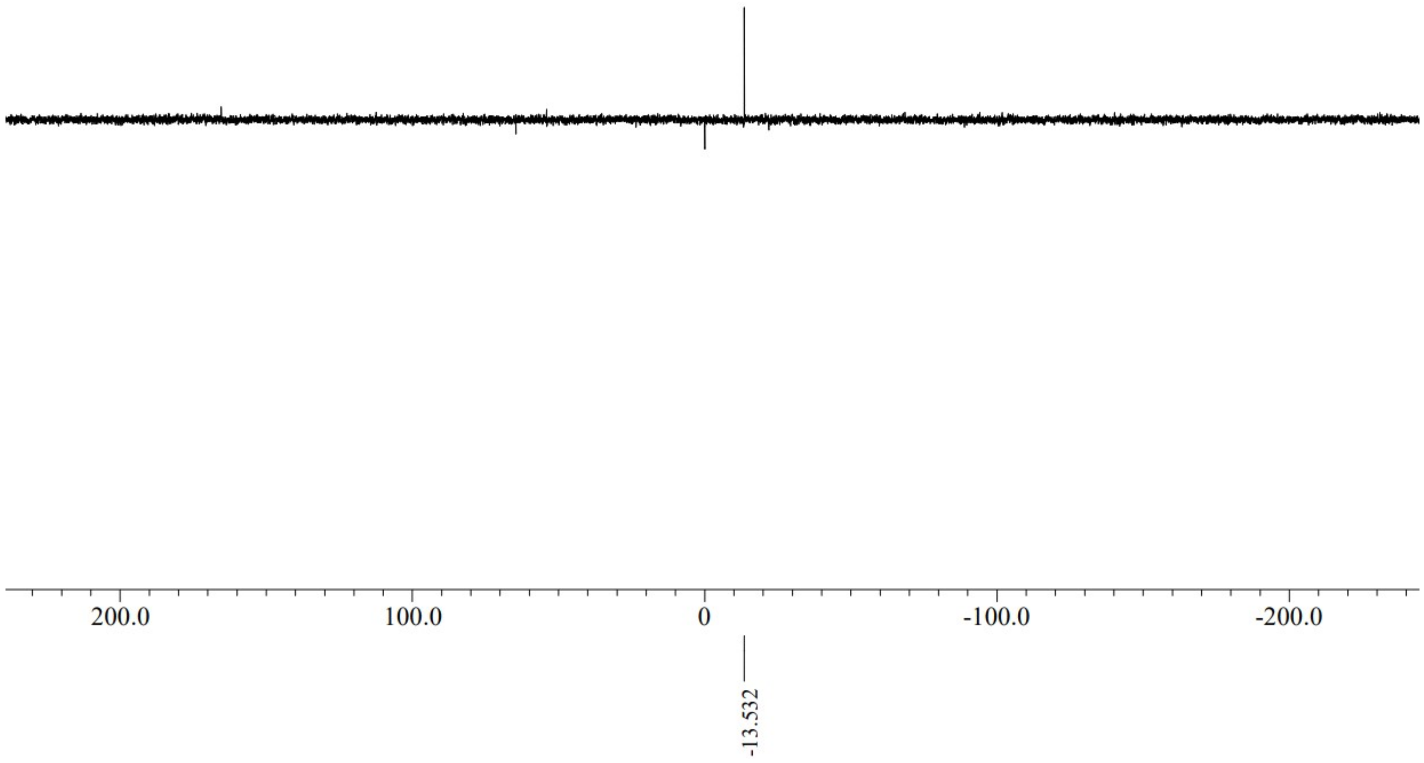

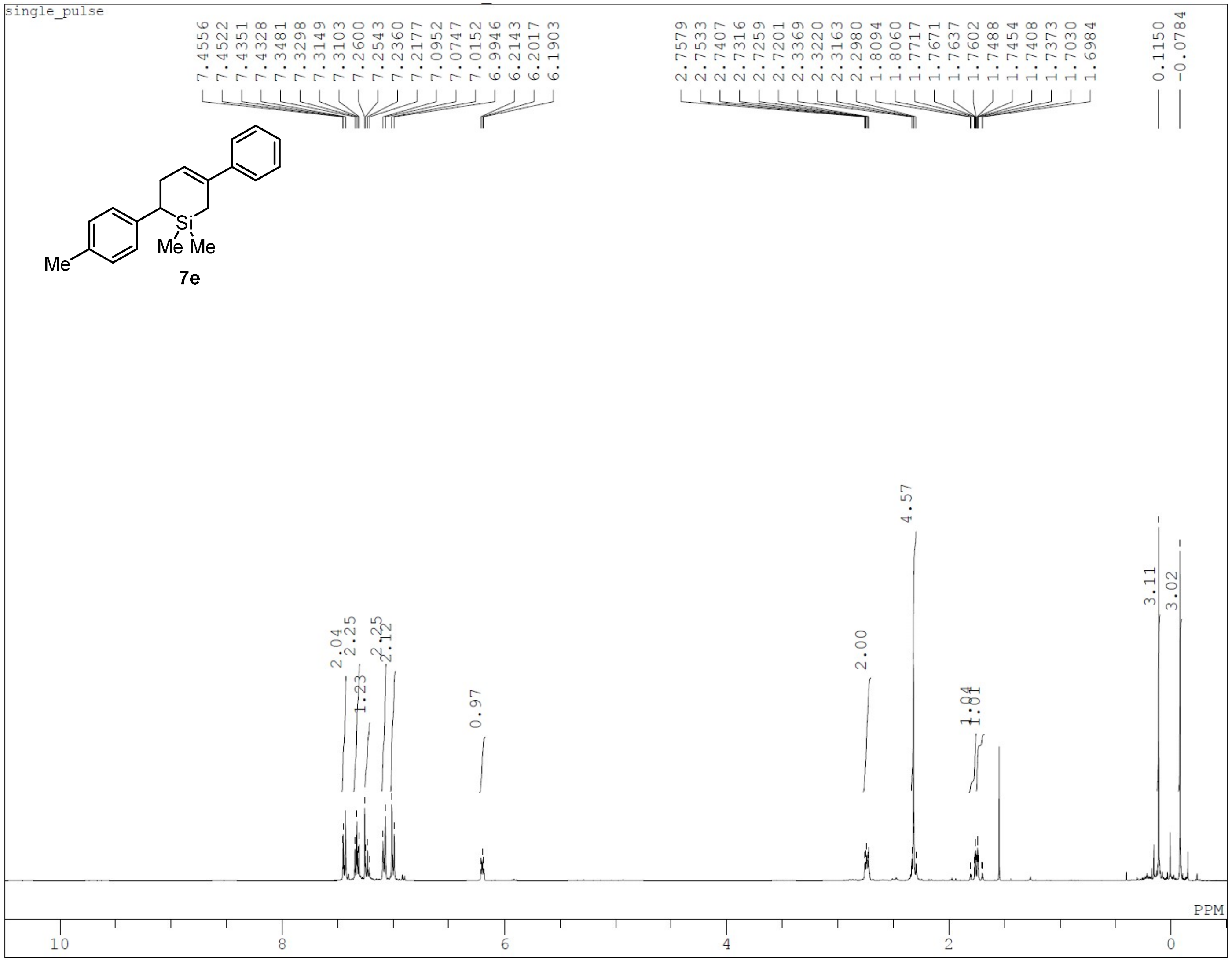
single_pulse
7e
7.4556
7.4522
7.4351
7.4328
7.3481
7.3298
7.3149
7.3103
7.2600
7.2543
7.2360
7.2177
7.0952
7.0747
7.0152
6.9946
6.2143
6.2017
6.1903
2.7579
2.7533
2.7407
2.7316
2.7259
2.7201
2.3369
2.3220
2.3163
2.2980
1.8094
1.8060
1.7717
1.7671
1.7637
1.7602
1.7488
1.7454
1.7408
1.7373
1.7030
1.6984
0.1150
-0.0784
2.04
2.25
1.23
2.25
2.12
0.97
2.00
4.57
1.04
1.01
3.11
3.02
PPM
10
8
6
4
2
0

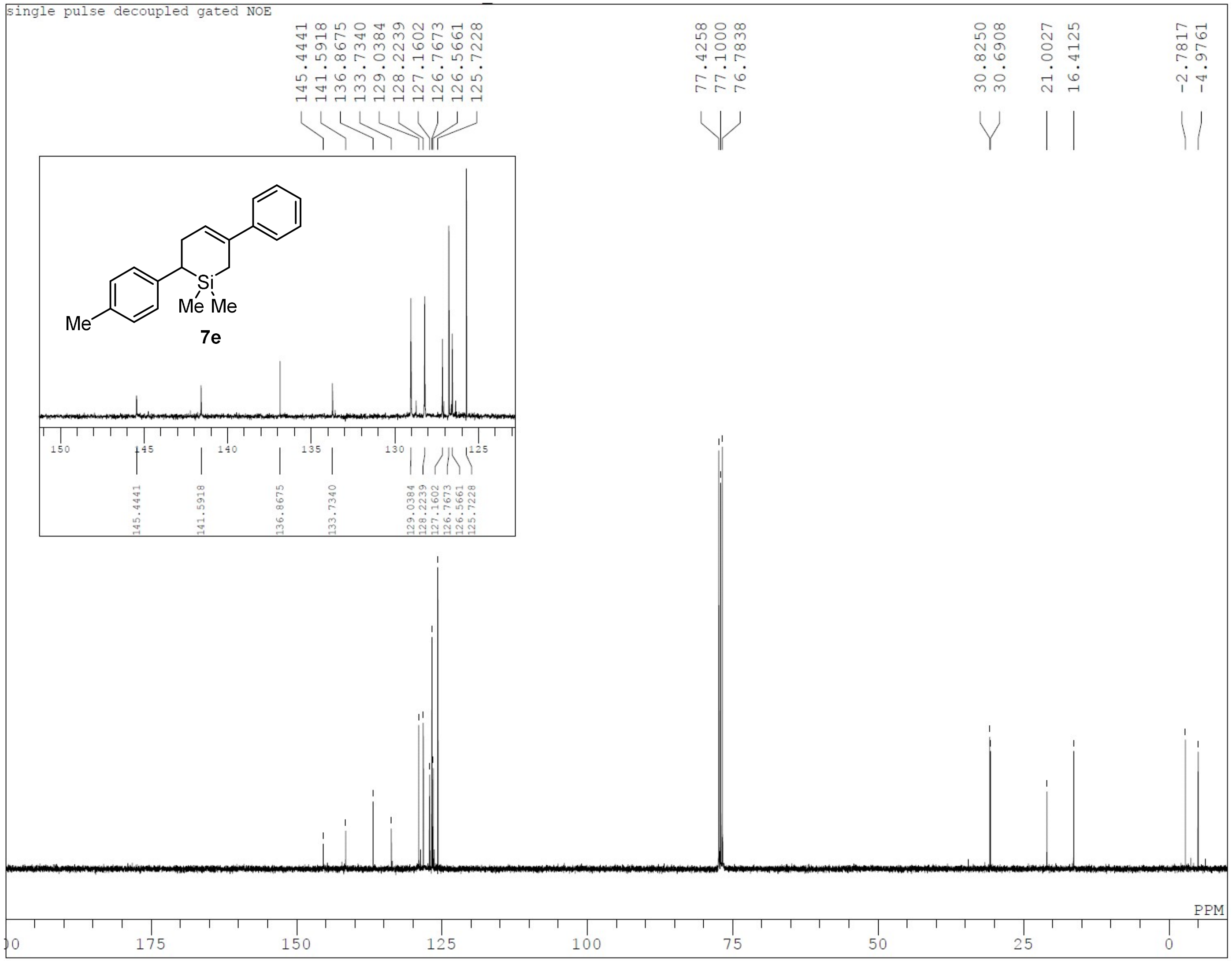
single pulse decoupled gated NOE
145.4441
141.5918
136.8675
133.7340
129.0384
128.2239
127.1602
126.7673
126.5661
125.7228
77.4258
77.1000
76.7838
30.8250
30.6908
21.0027
16.4125
-2.7817
-4.9761
Si
Me
Me
Me
7e
PPM
175
150
125
100
75
50
25
0

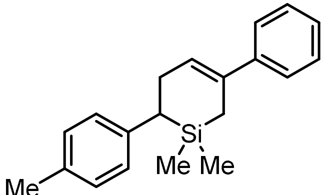

7e

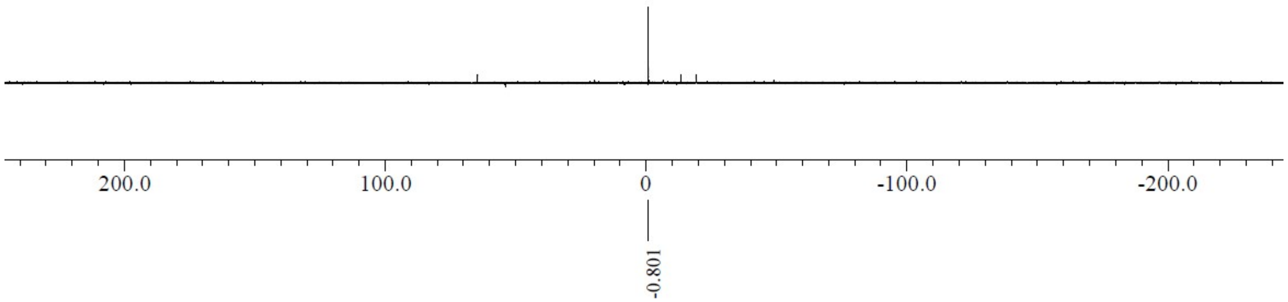

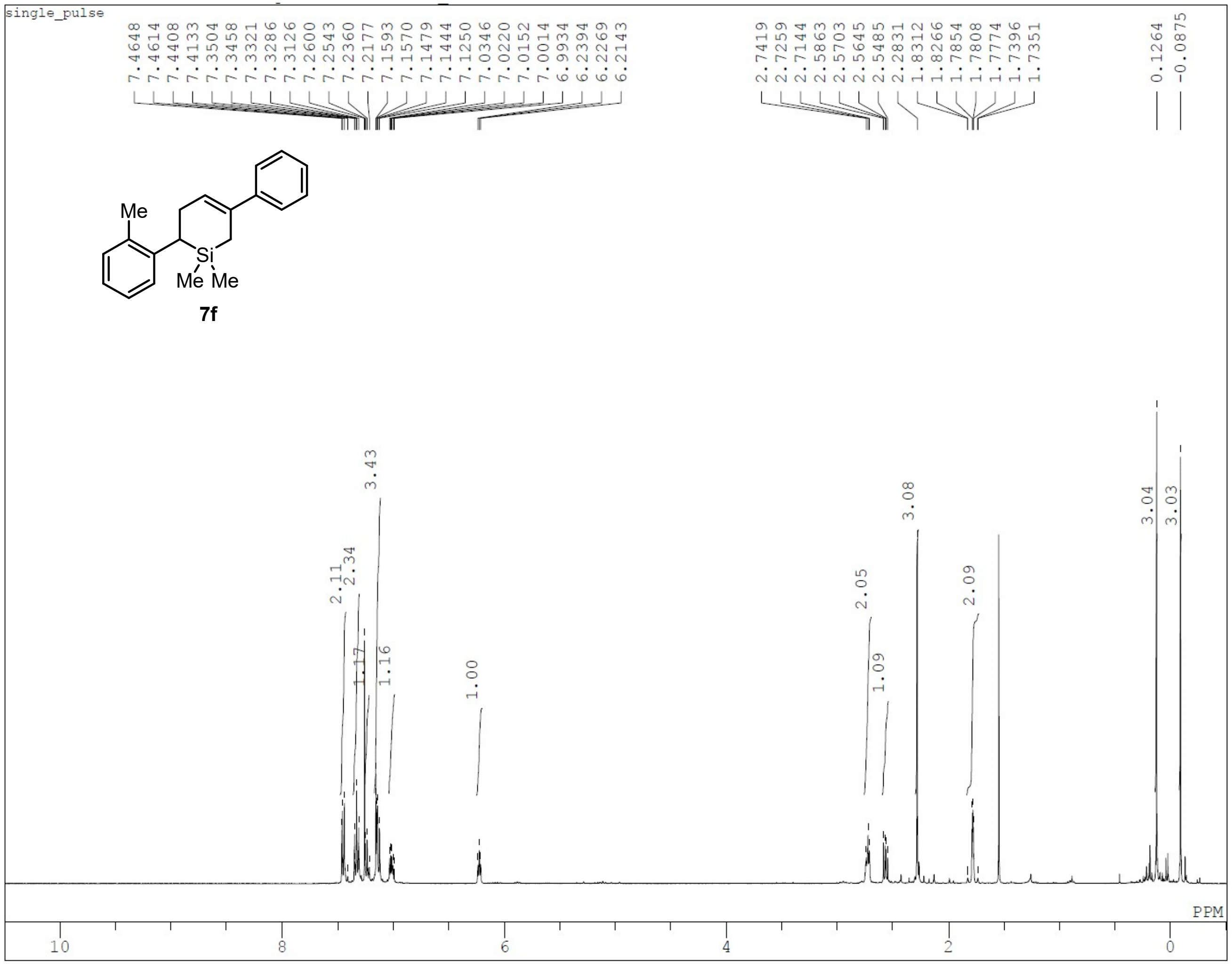
Me
Si
Me Me
7f
single_pulse
7.4648 7.4614 7.4408 7.4133 7.3504 7.3458 7.3321 7.3286 7.3126 7.2600 7.2543 7.2360 7.2177 7.1593 7.1570 7.1479 7.1444 7.1250 7.0346 7.0220 7.0152 7.0014 6.9934 6.2394 6.2269 6.2143
2.7419 2.7259 2.7144 2.5863 2.5703 2.5645 2.5485 2.2831 1.8312 1.8266 1.7854 1.7808 1.7774 1.7396 1.7351
0.1264 -0.0875
2.11 2.34 1.17 3.43 1.16 1.00 2.05 1.09 3.08 2.09 3.04 3.03
PPM
10 8 6 4 2 0

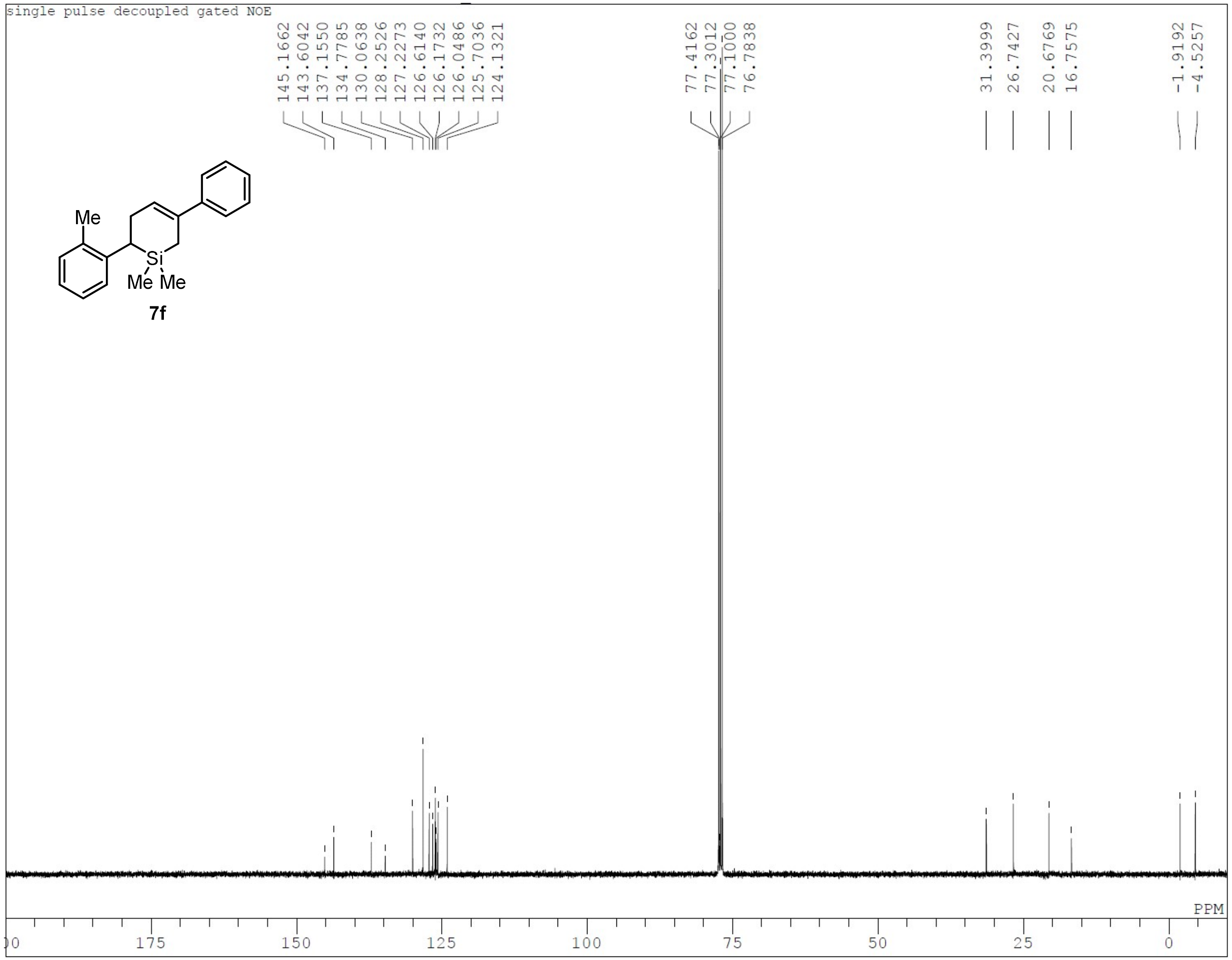
single pulse decoupled gated NOE
145.1662
143.6042
137.1550
134.7785
130.0638
128.2526
127.2273
126.6140
126.1732
126.0486
125.7036
124.1321
77.4162
77.3012
77.1000
76.7838
31.3999
26.7427
20.6769
16.7575
-1.9192
-4.5257
Me
Si
Me Me
7f
PPM
175
150
125
100
75
50
25
0

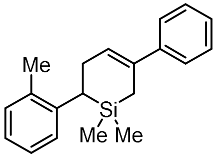
Me
Si
Me Me

7f

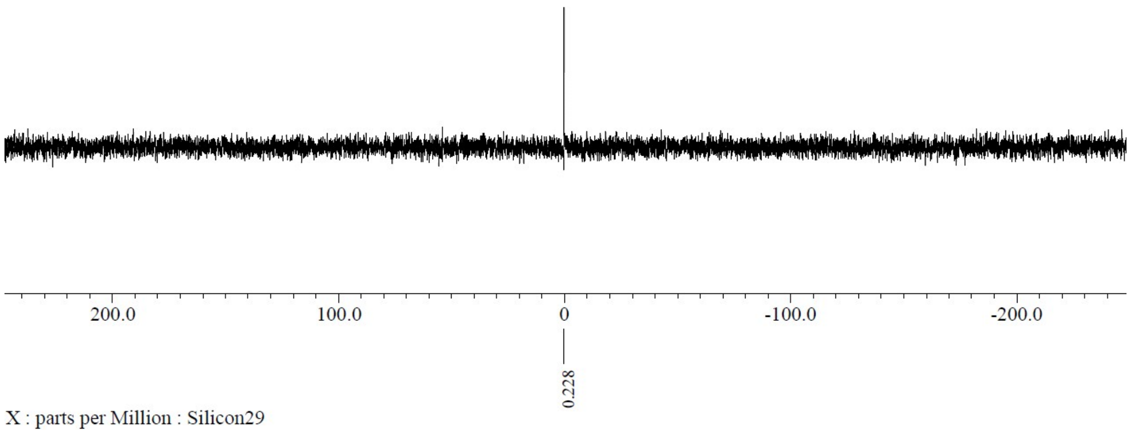
200.0
100.0
0
-100.0
-200.0
0.228
X : parts per Million : Silicon29

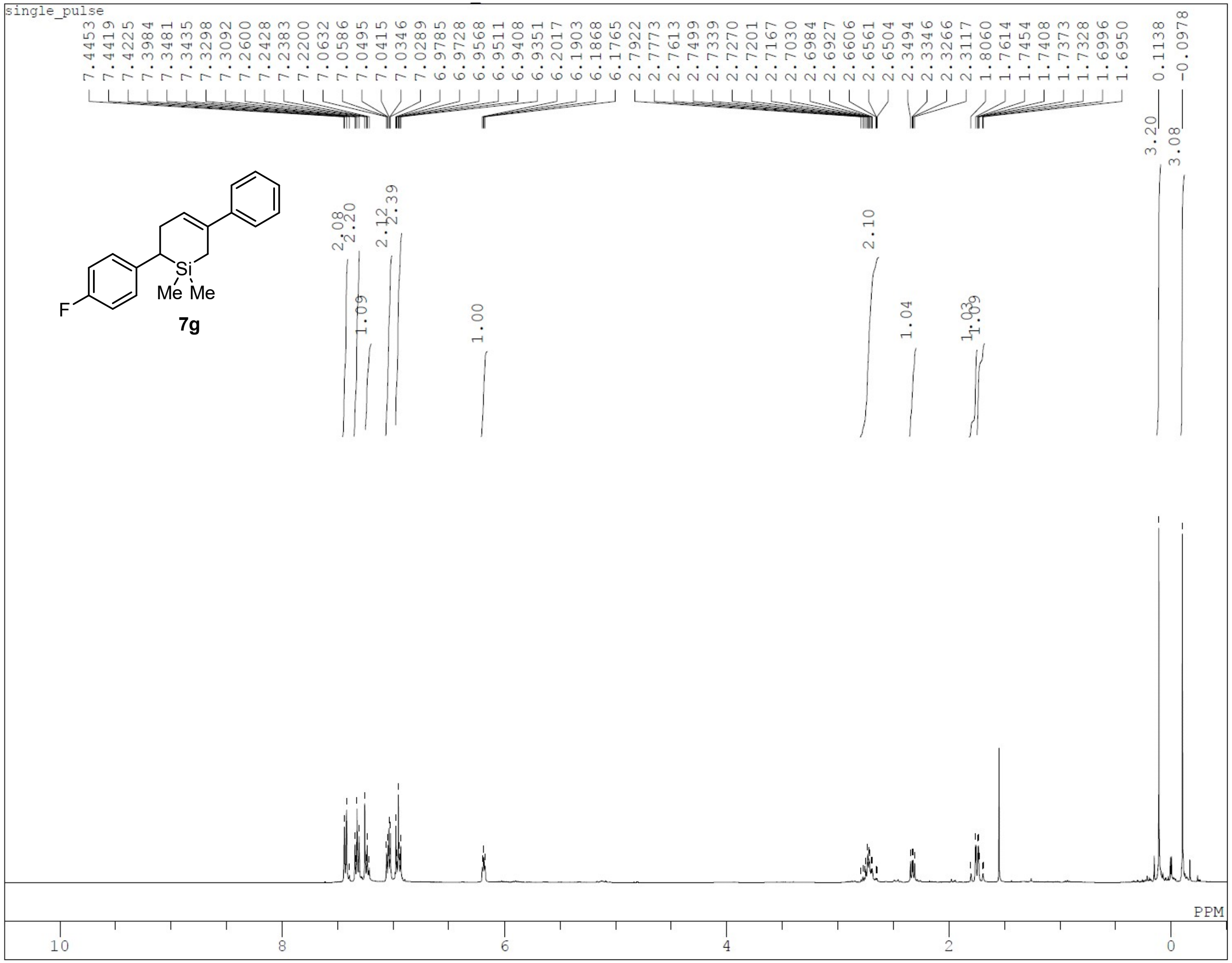
single_pulse
7.4453
7.4419
7.4225
7.3984
7.3481
7.3435
7.3298
7.3092
7.2600
7.2428
7.2383
7.2200
7.0632
7.0586
7.0495
7.0415
7.0346
7.0289
6.9785
6.9728
6.9568
6.9511
6.9408
6.9351
6.2017
6.1903
6.1868
6.1765
2.7922
2.7773
2.7613
2.7499
2.7339
2.7270
2.7201
2.7167
2.7030
2.6984
2.6927
2.6606
2.6561
2.6504
2.3494
2.3346
2.3266
2.3117
1.8060
1.7614
1.7454
1.7408
1.7373
1.7328
1.6996
1.6950
0.1138
-0.0978
2.08
2.20
1.09
2.12
2.39
1.00
2.10
1.04
1.03
1.09
3.20
3.08
F
Si
Me Me
7g
PPM
10
8
6
4
2
0

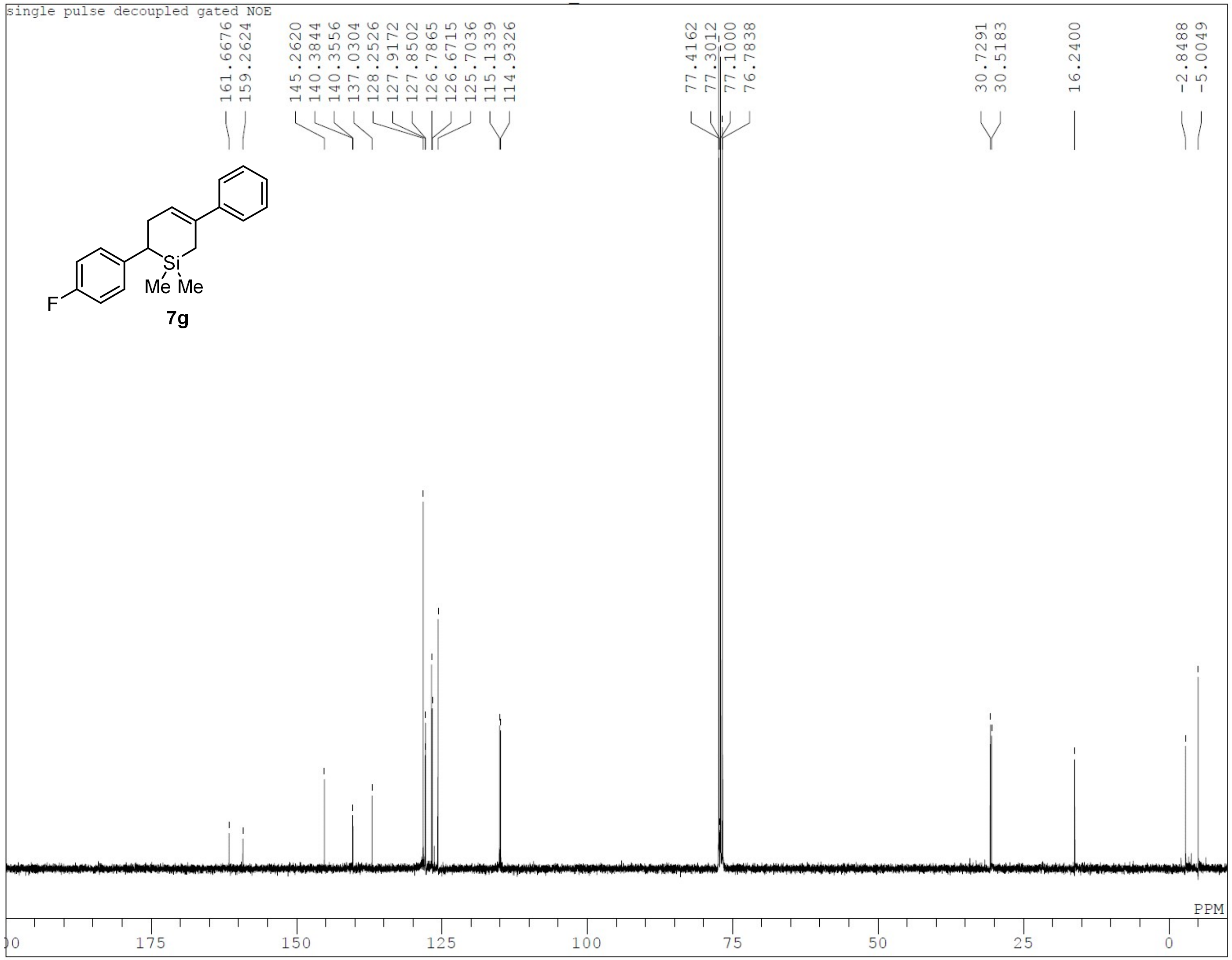
single pulse decoupled gated NOE
161.6676
159.2624
145.2620
140.3844
140.3556
137.0304
128.2526
127.9172
127.8502
126.7865
126.6715
125.7036
115.1339
114.9326
77.4162
77.3012
77.1000
76.7838
30.7291
30.5183
16.2400
-2.8488
-5.0049
F
Si
Me Me
7g
PPM
175
150
125
100
75
50
25
0

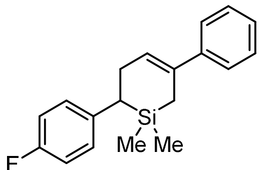

7g

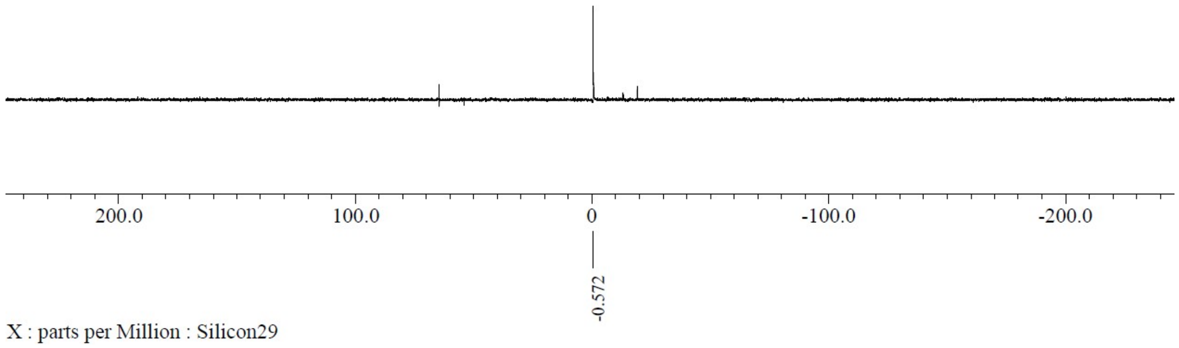

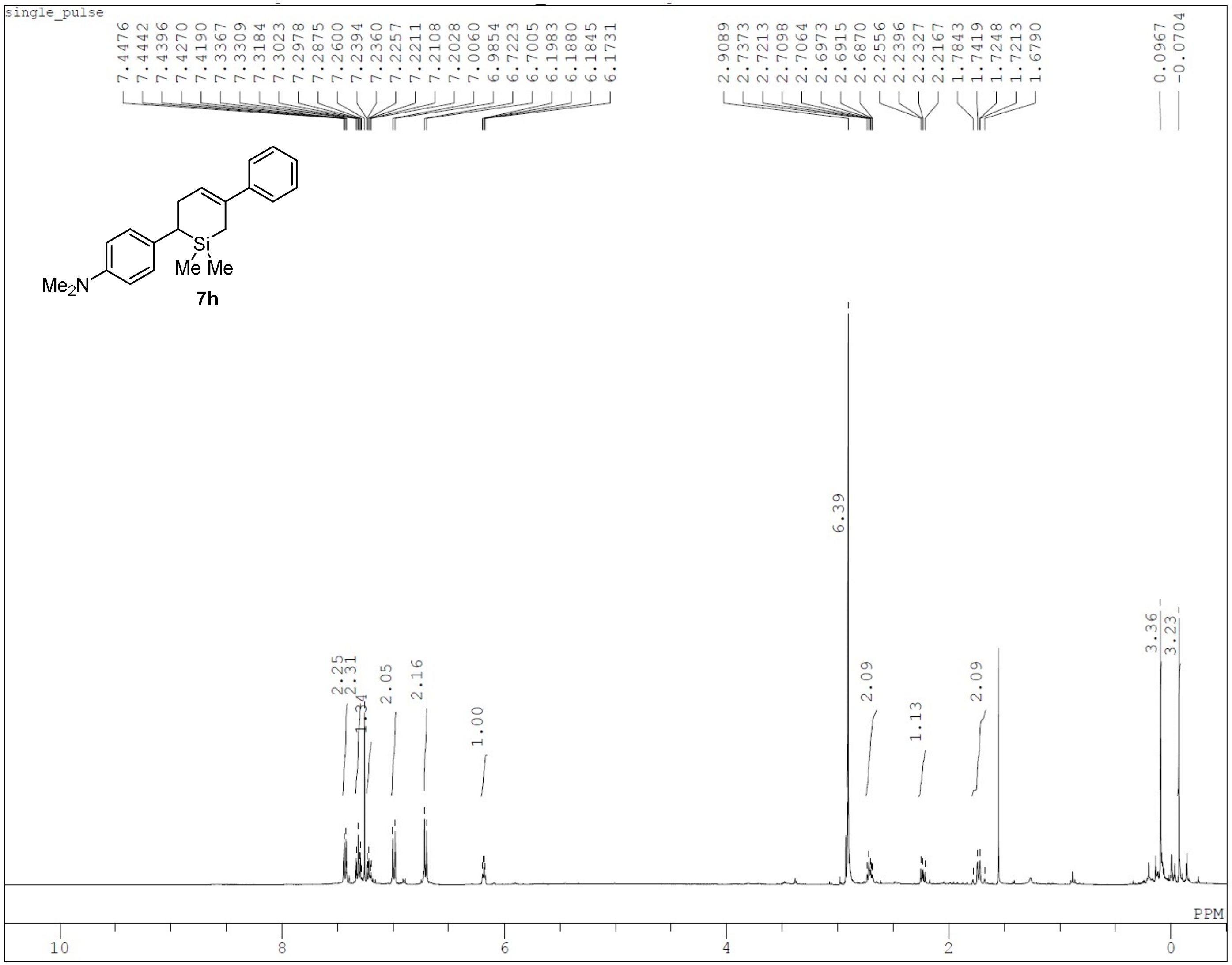

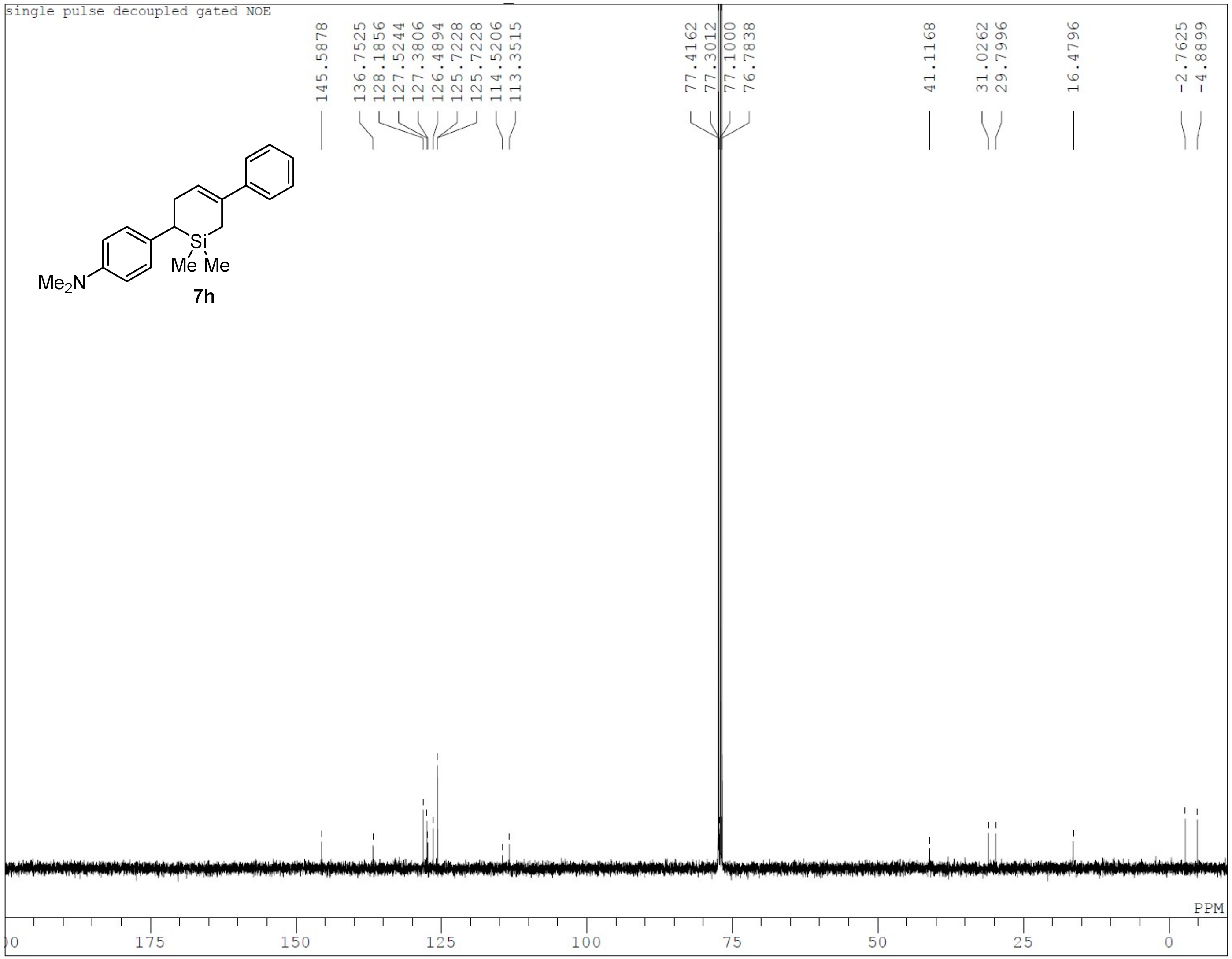
single pulse decoupled gated NOE
145.5878
136.7525
128.1856
127.5244
127.3806
126.4894
125.7228
125.7228
114.5206
113.3515
77.4162
77.3012
77.1000
76.7838
41.1168
31.0262
29.7996
16.4796
-2.7625
-4.8899
Me2N
Si
Me Me
7h
PPM
175
150
125
100
75
50
25
0

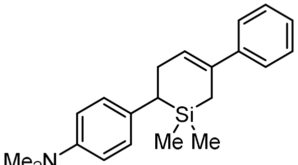
Me2N
Si
Me Me

7h

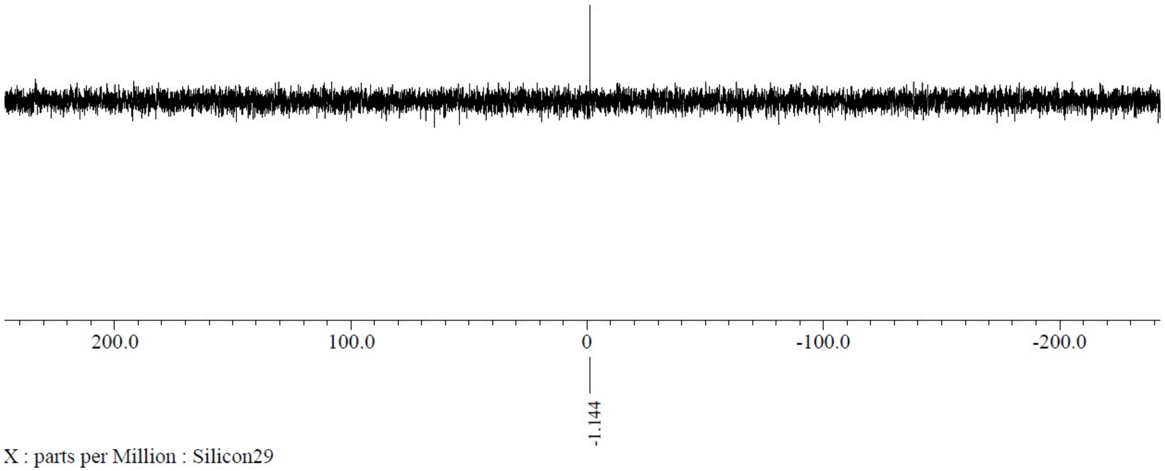
200.0
100.0
0
-100.0
-200.0
-1.144
X : parts per Million : Silicon29

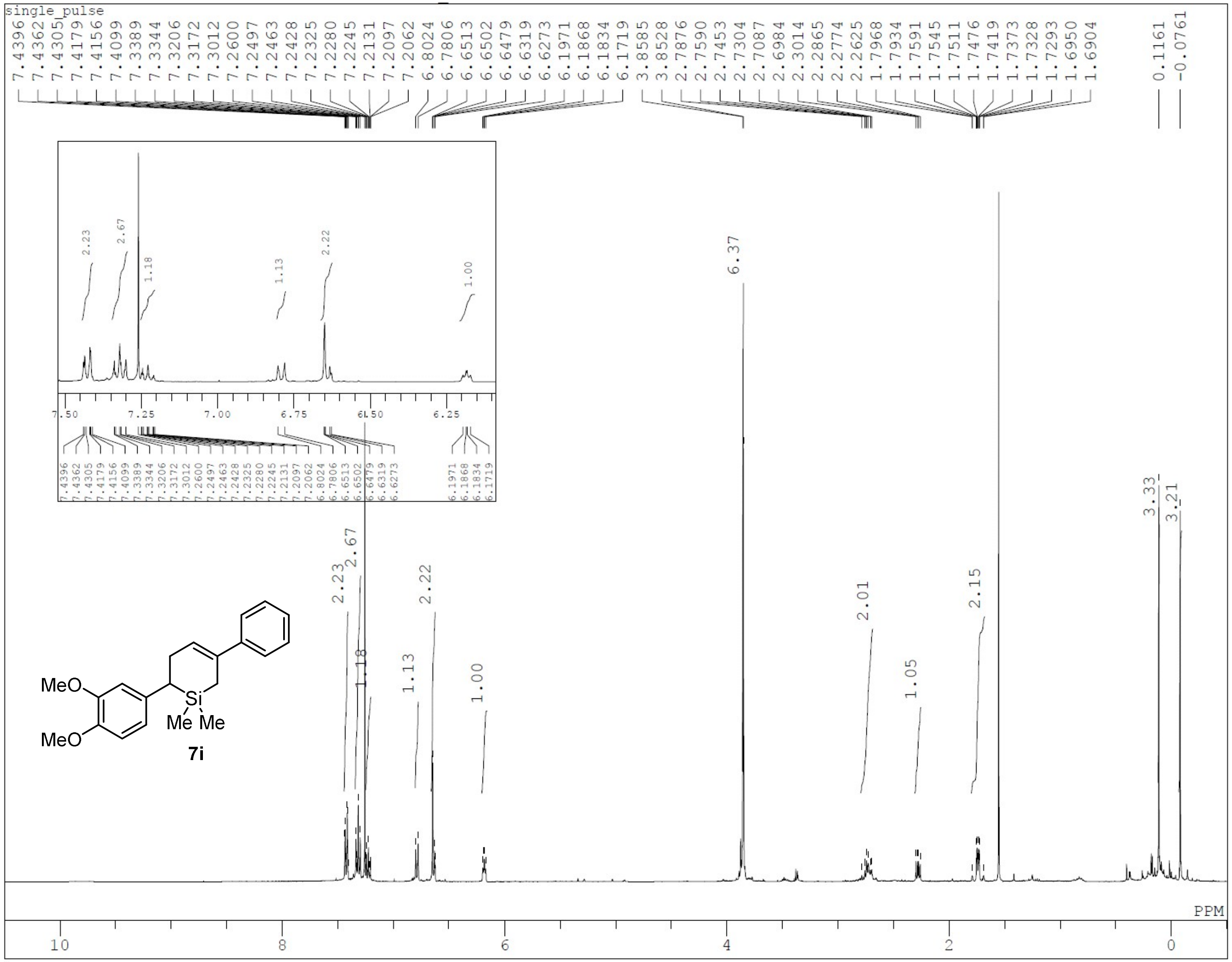
single_pulse
7.4396
7.4362
7.4305
7.4179
7.4156
7.4099
7.3389
7.3344
7.3206
7.3172
7.3012
7.2600
7.2497
7.2463
7.2428
7.2325
7.2280
7.2245
7.2131
7.2097
7.2062
6.8024
6.7806
6.6513
6.6502
6.6479
6.6319
6.6273
6.1971
6.1868
6.1834
6.1719
3.8585
3.8528
2.7876
2.7590
2.7453
2.7304
2.7087
2.6984
2.3014
2.2865
2.2774
2.2625
1.7968
1.7934
1.7591
1.7545
1.7511
1.7476
1.7419
1.7373
1.7328
1.7293
1.6950
1.6904
0.1161
-0.0761
2.23
2.67
1.18
1.13
2.22
1.00
6.37
2.01
1.05
2.15
3.33
3.21
MeO
MeO
Si
Me Me
PPM
10
8
6
4
2
0
7i

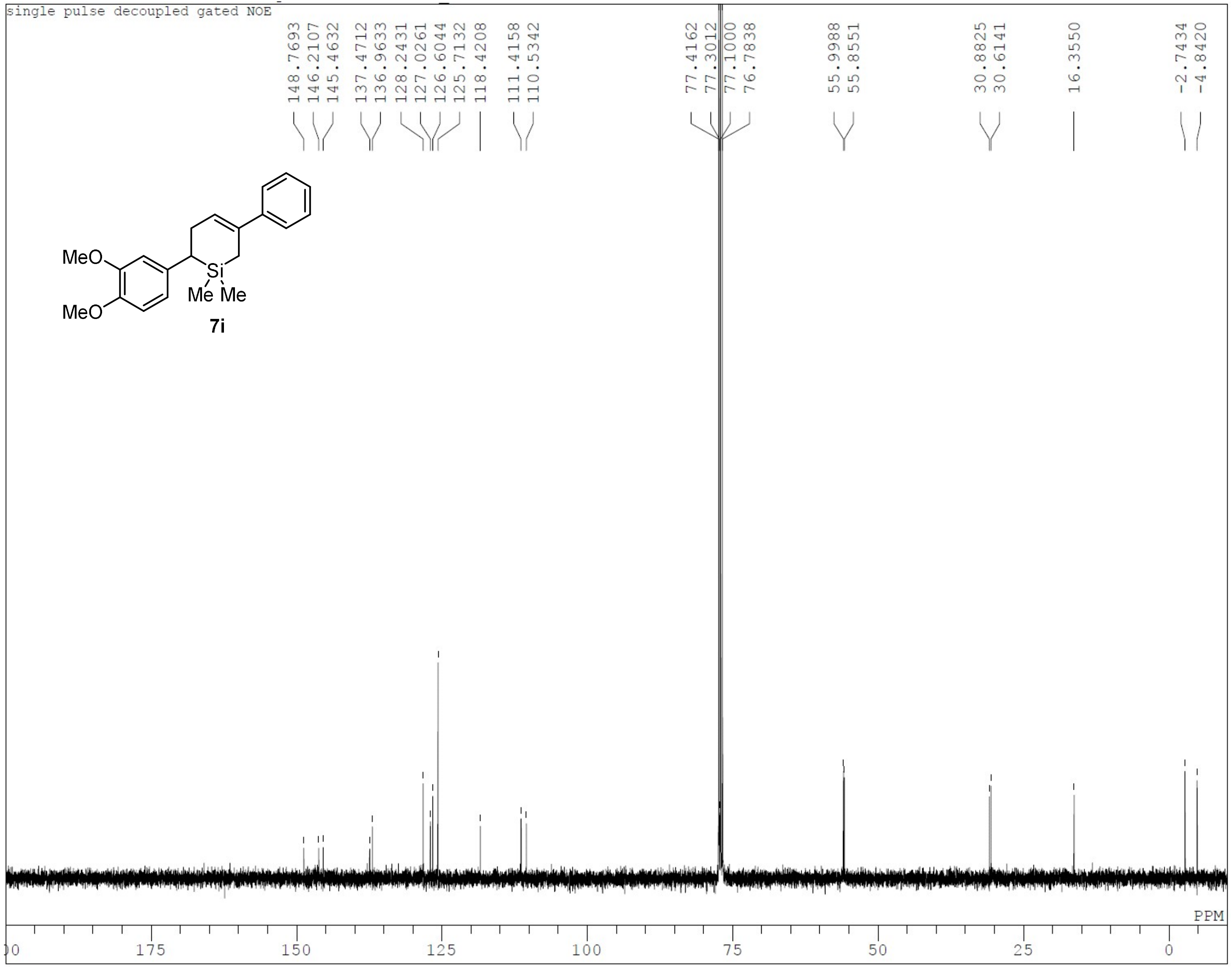
single pulse decoupled gated NOE
148.7693
146.2107
145.4632
137.4712
136.9633
128.2431
127.0261
126.6044
125.7132
118.4208
111.4158
110.5342
77.4162
77.3012
77.1000
76.7838
55.9988
55.8551
30.8825
30.6141
16.3550
-2.7434
-4.8420
MeO
MeO
Si
Me Me
7i
PPM
175
150
125
100
75
50
25
0

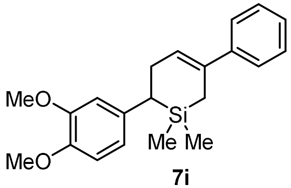
MeO
MeO
Si
Me Me

7i

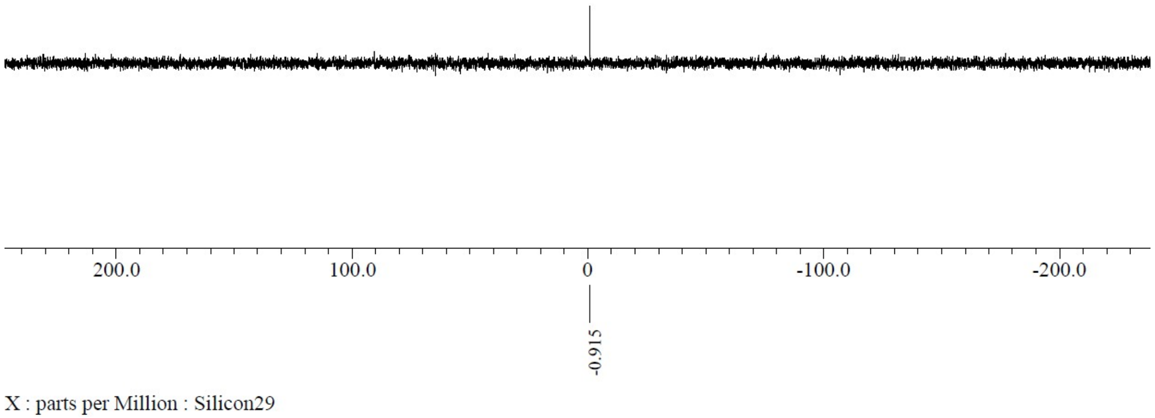
200.0
100.0
0
-100.0
-200.0
-0.915
X : parts per Million : Silicon29

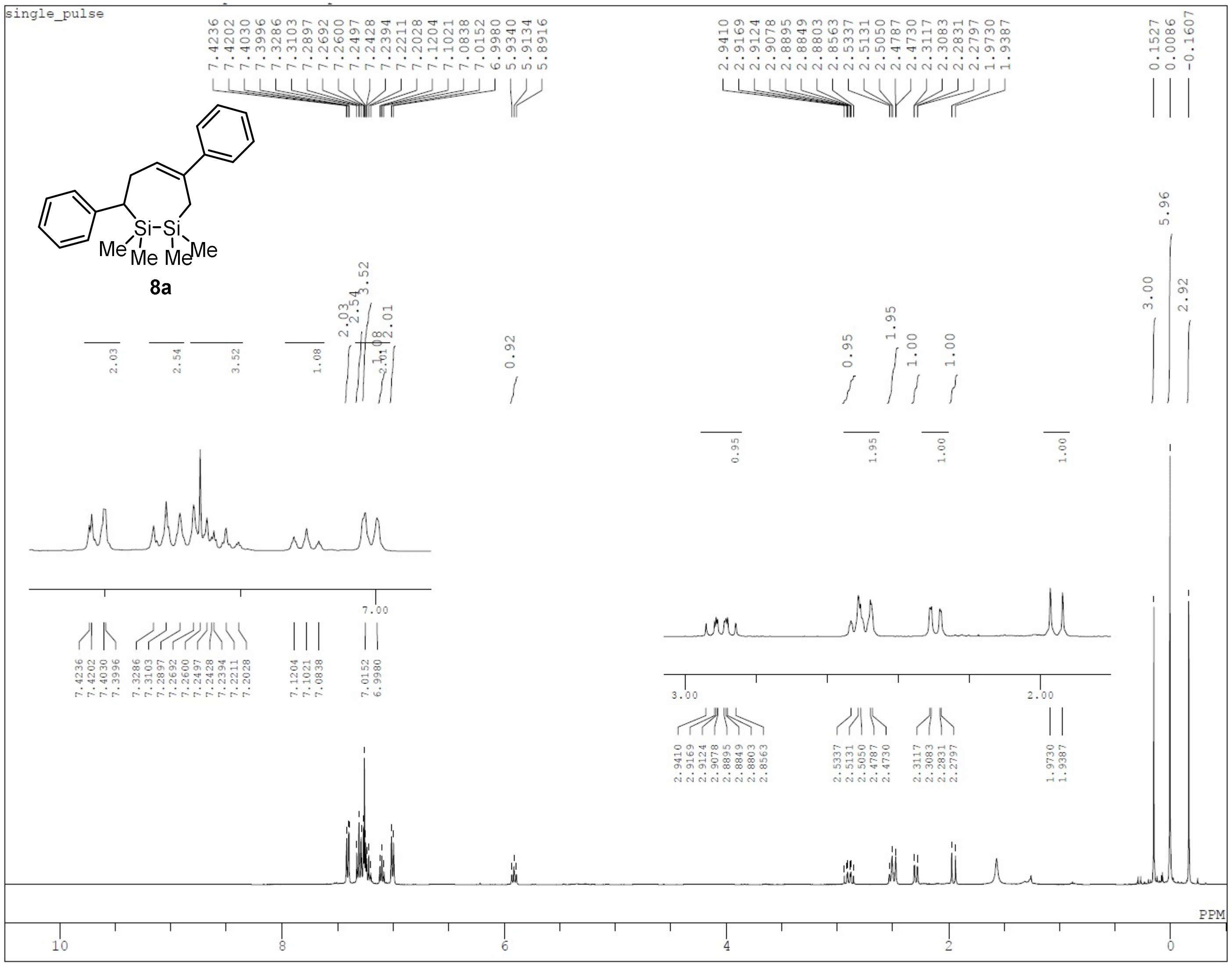

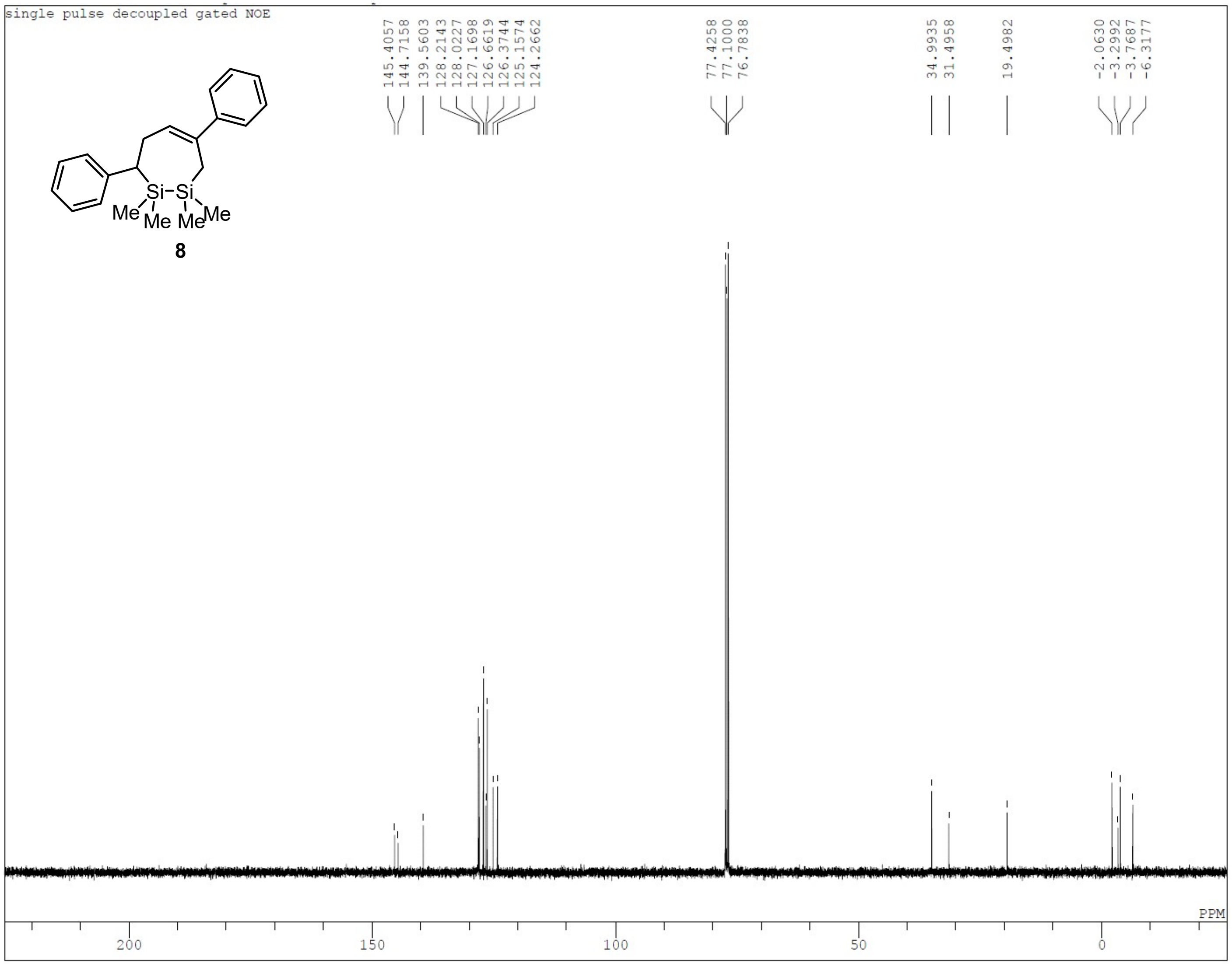
single pulse decoupled gated NOE
145.4057
144.7158
139.5603
128.2143
128.0227
127.1698
126.6619
126.3744
125.1574
124.2662
77.4258
77.1000
76.7838
34.9935
31.4958
19.4982
-2.0630
-3.2992
-3.7687
-6.3177
Si
Si
Me
Me
Me
Me
8
PPM
200
150
100
50
0

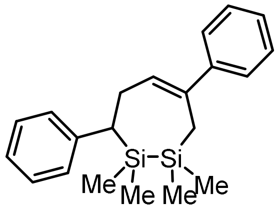

8

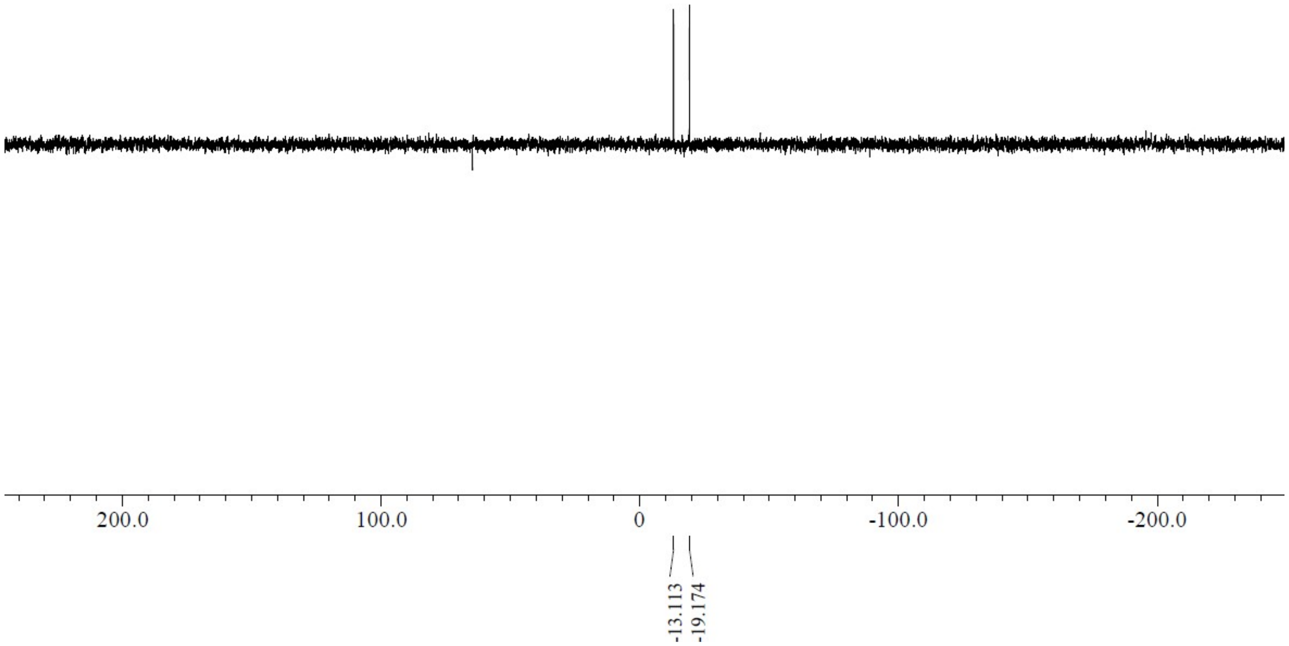

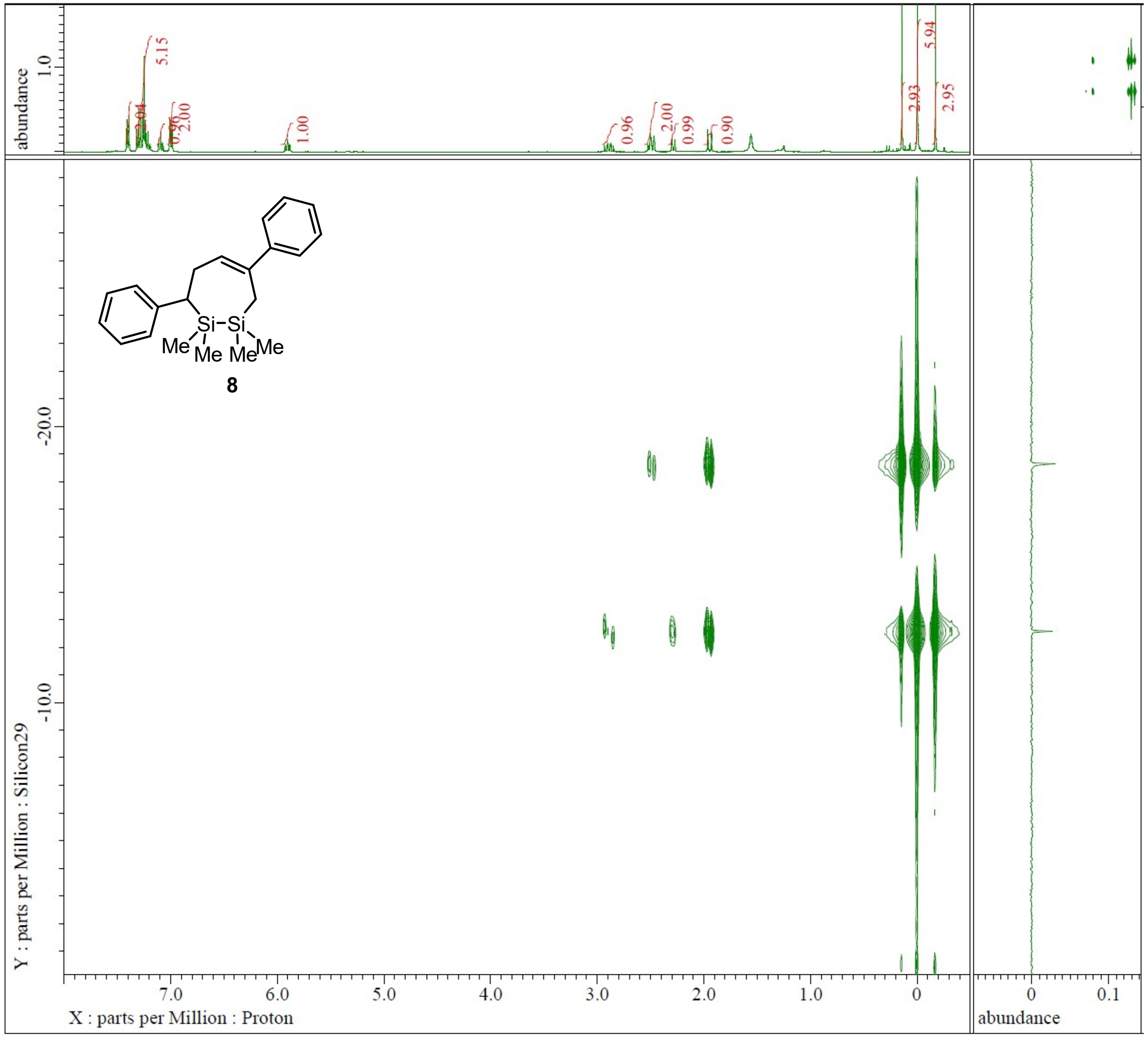
abundance
1.0
5.15
2.04
0.96
2.00
1.00
0.96
2.00
0.99
0.90
2.93
5.94
2.95
Me
Me
Me
Me
Si
Si
8
-20.0
-10.0
Y : parts per Million : Silicon29
7.0
6.0
5.0
4.0
3.0
2.0
1.0
0
X : parts per Million : Proton
0
0.1
abundance

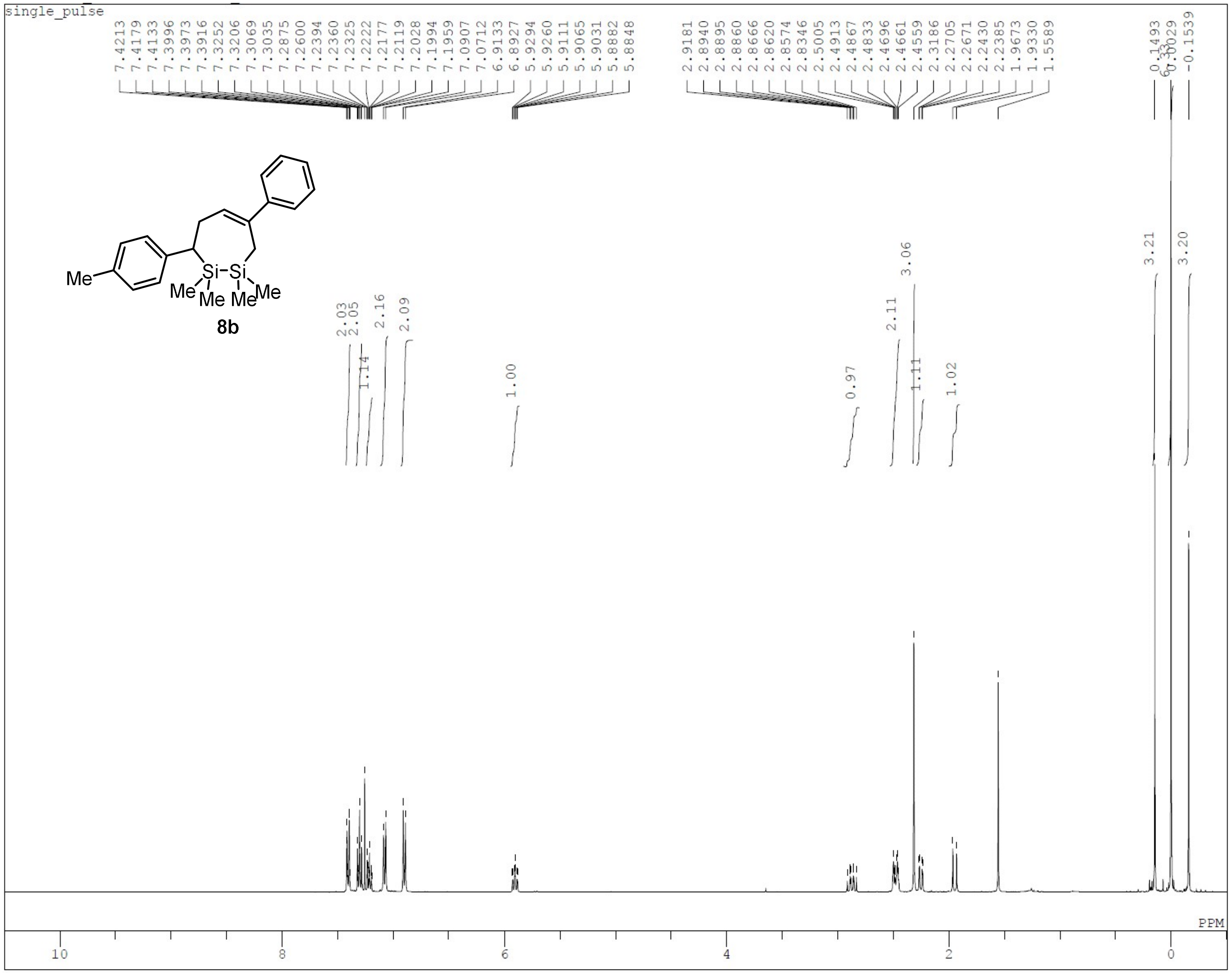
single_pulse
7.4213
7.4179
7.4133
7.3996
7.3973
7.3916
7.3252
7.3206
7.3069
7.3035
7.2875
7.2600
7.2394
7.2360
7.2325
7.2222
7.2177
7.2119
7.2028
7.1994
7.1959
7.0907
7.0712
6.9133
6.8927
5.9294
5.9260
5.9111
5.9065
5.9031
5.8882
5.8848
2.9181
2.8940
2.8895
2.8860
2.8666
2.8620
2.8574
2.8346
2.5005
2.4913
2.4867
2.4833
2.4696
2.4661
2.4559
2.3186
2.2705
2.2671
2.2430
2.2385
1.9673
1.9330
1.5589
0.1493
0.0029
-0.1539
Me
Si
Si
Me
Me
Me
Me
8b
2.03
2.05
1.14
2.16
2.09
1.00
0.97
2.11
3.06
1.11
1.02
3.21
6.33
3.20
PPM
10
8
6
4
2
0

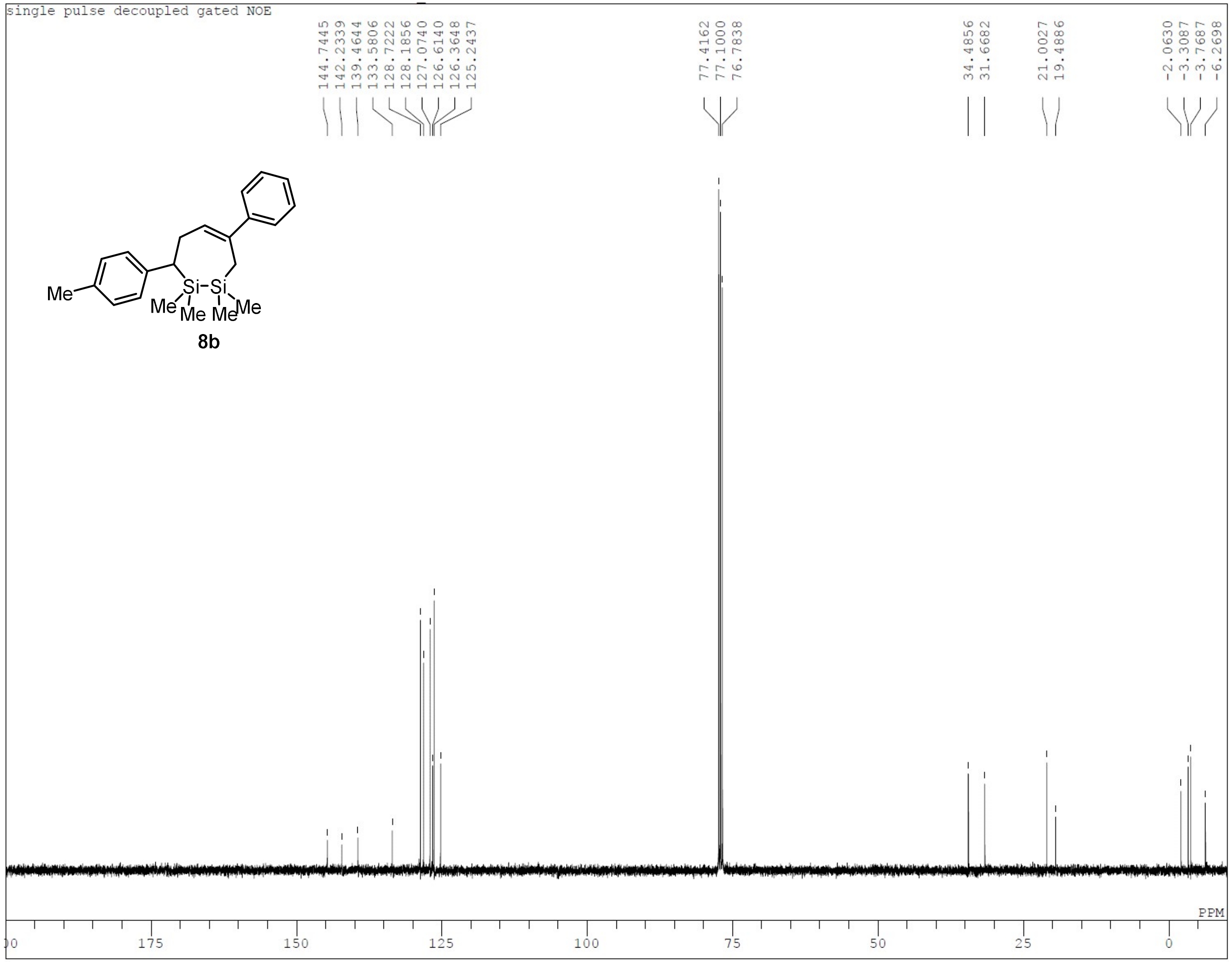
single pulse decoupled gated NOE
144.7445
142.2339
139.4644
133.5806
128.7222
128.1856
127.0740
126.6140
126.3648
125.2437
77.4162
77.1000
76.7838
34.4856
31.6682
21.0027
19.4886
-2.0630
-3.3087
-3.7687
-6.2698
Me
Si
Si
Me
Me
Me
Me
8b
PPM
175
150
125
100
75
50
25
0

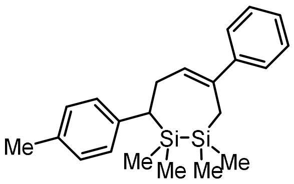

8b

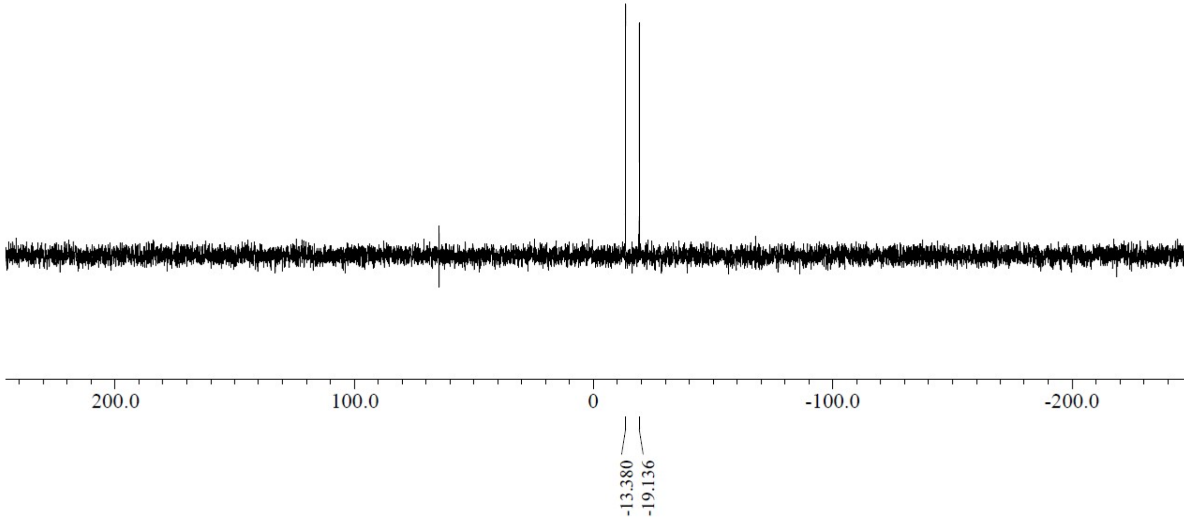

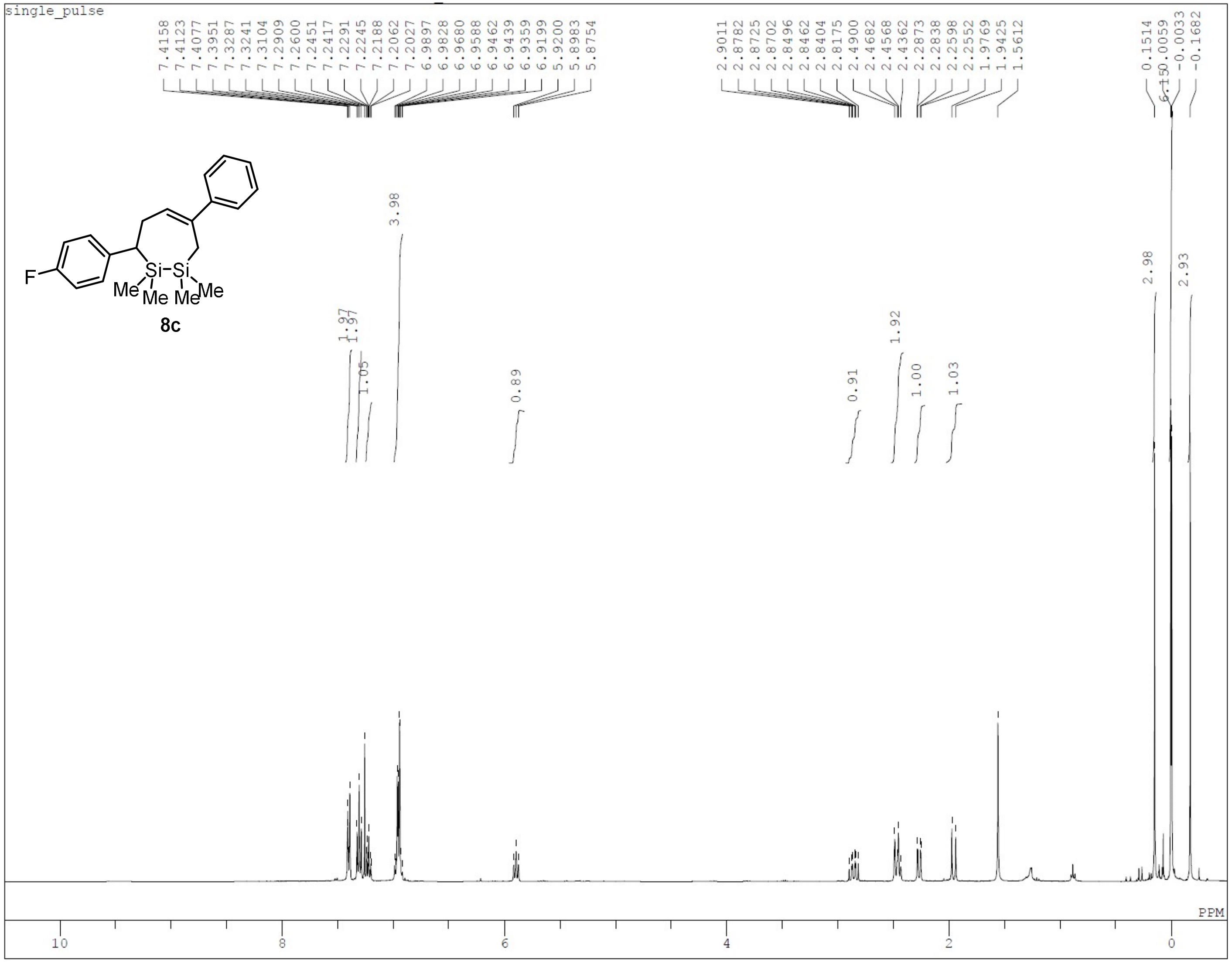
single_pulse
7.4158
7.4123
7.4077
7.3951
7.3287
7.3241
7.3104
7.2909
7.2600
7.2451
7.2417
7.2291
7.2245
7.2188
7.2062
7.2027
6.9897
6.9828
6.9680
6.9588
6.9462
6.9439
6.9359
6.9199
5.9200
5.8983
5.8754
2.9011
2.8782
2.8725
2.8702
2.8496
2.8462
2.8404
2.8175
2.4900
2.4682
2.4568
2.4362
2.2873
2.2838
2.2598
2.2552
1.9769
1.9425
1.5612
0.1514
0.0059
-0.0033
-0.1682
F
Si
Si
Me
Me
Me
Me
8c
1.97
1.97
1.05
3.98
0.89
0.91
1.92
1.00
1.03
2.98
6.10
2.93
PPM
10
8
6
4
2
0

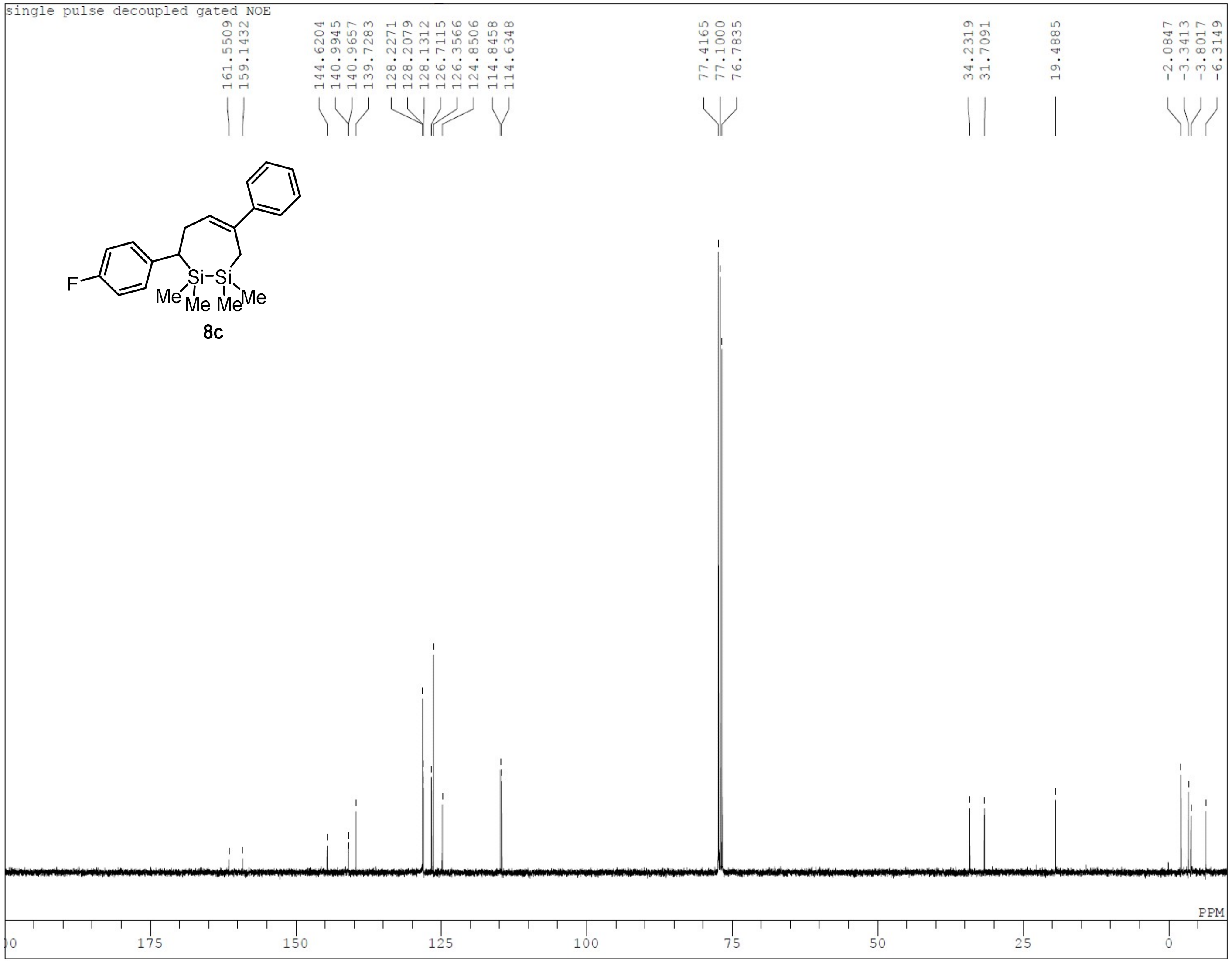
single pulse decoupled gated NOE
161.5509
159.1432
144.6204
140.9945
140.9657
139.7283
128.2271
128.2079
128.1312
126.7115
126.3566
124.8506
114.8458
114.6348
77.4165
77.1000
76.7835
34.2319
31.7091
19.4885
-2.0847
-3.3413
-3.8017
-6.3149
F
Si
Si
Me
Me
Me
Me
8c
PPM
175
150
125
100
75
50
25
0

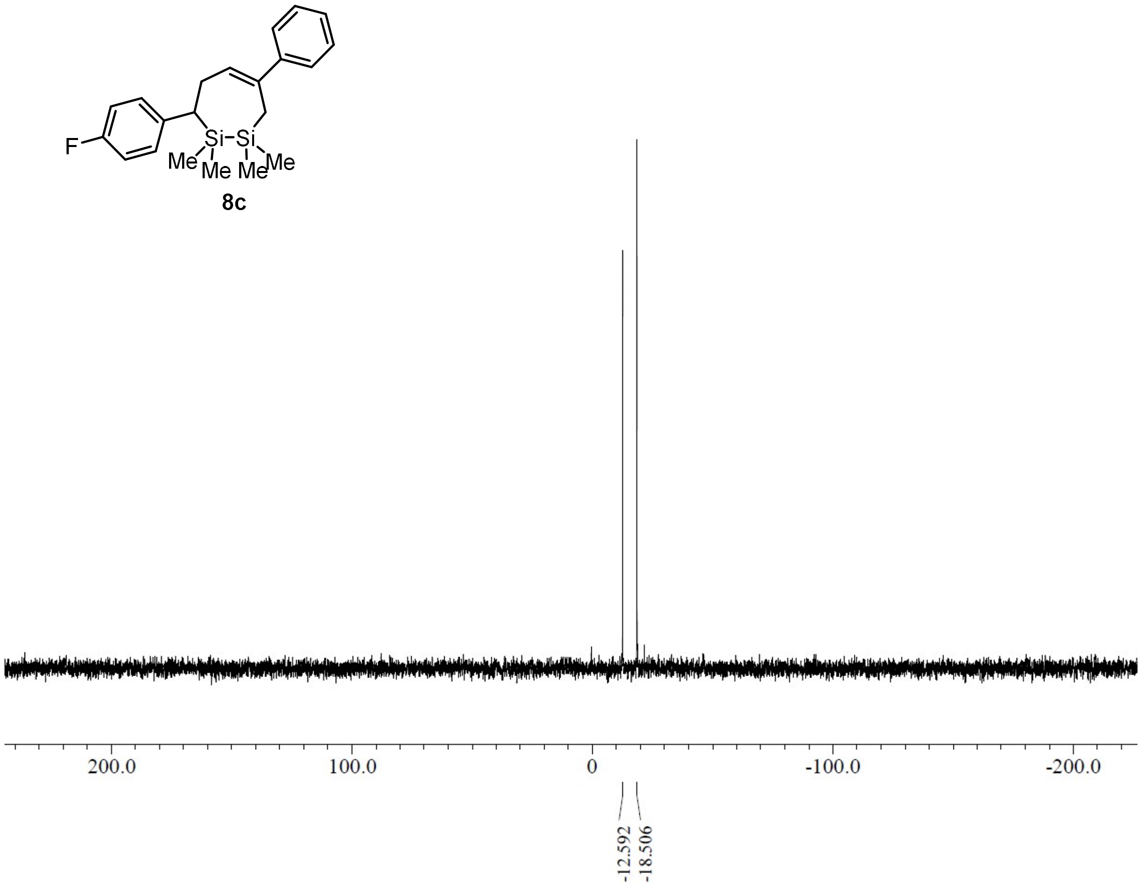

8c

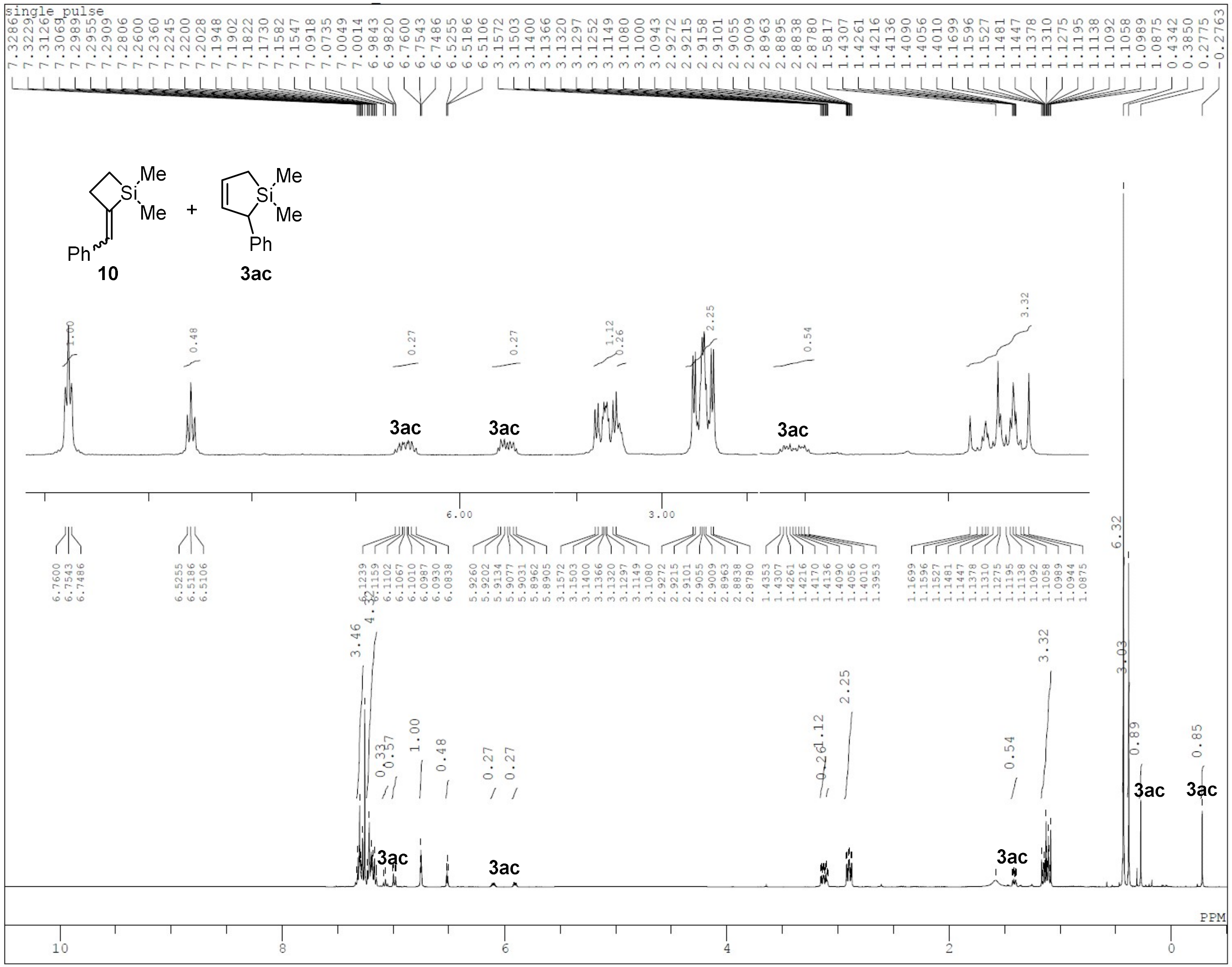

single pulse
10
3ac
3ac
PPM

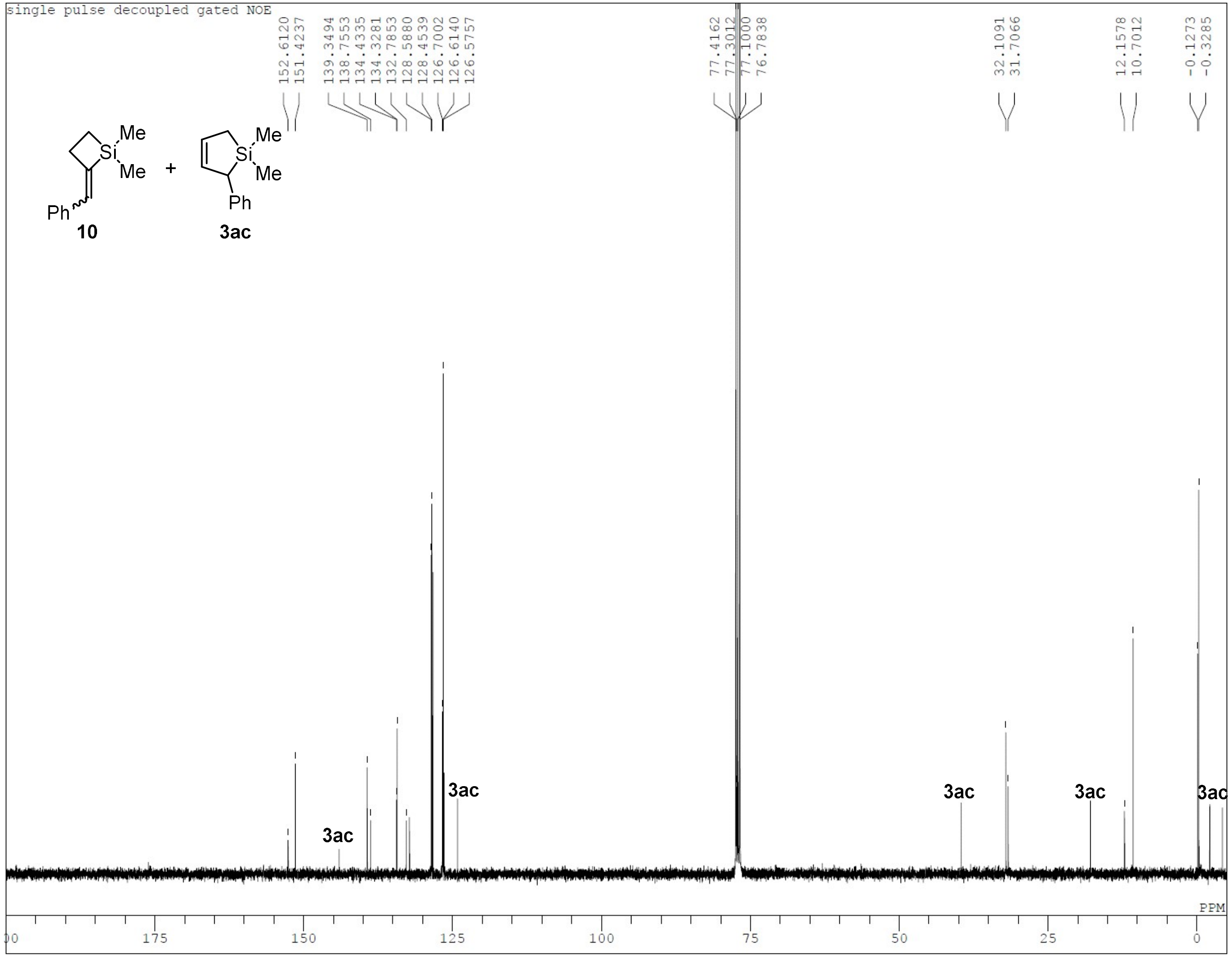
single pulse decoupled gated NOE
152.6120
151.4237
139.3494
138.7553
134.4335
134.3281
132.7853
128.5880
128.4539
126.7002
126.6140
126.5757
77.4162
77.3012
77.1000
76.7838
32.1091
31.7066
12.1578
10.7012
-0.1273
-0.3285
Me
Si
Me
Ph
10
+
Me
Si
Me
Ph
3ac
3ac
3ac
3ac
3ac
3ac
PPM
175
150
125
100
75
50
25
0

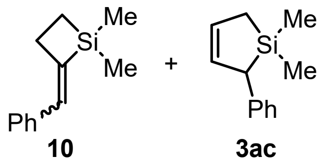
Me
Si
Me
Ph
10
+
Me
Si
Me
Ph
3ac

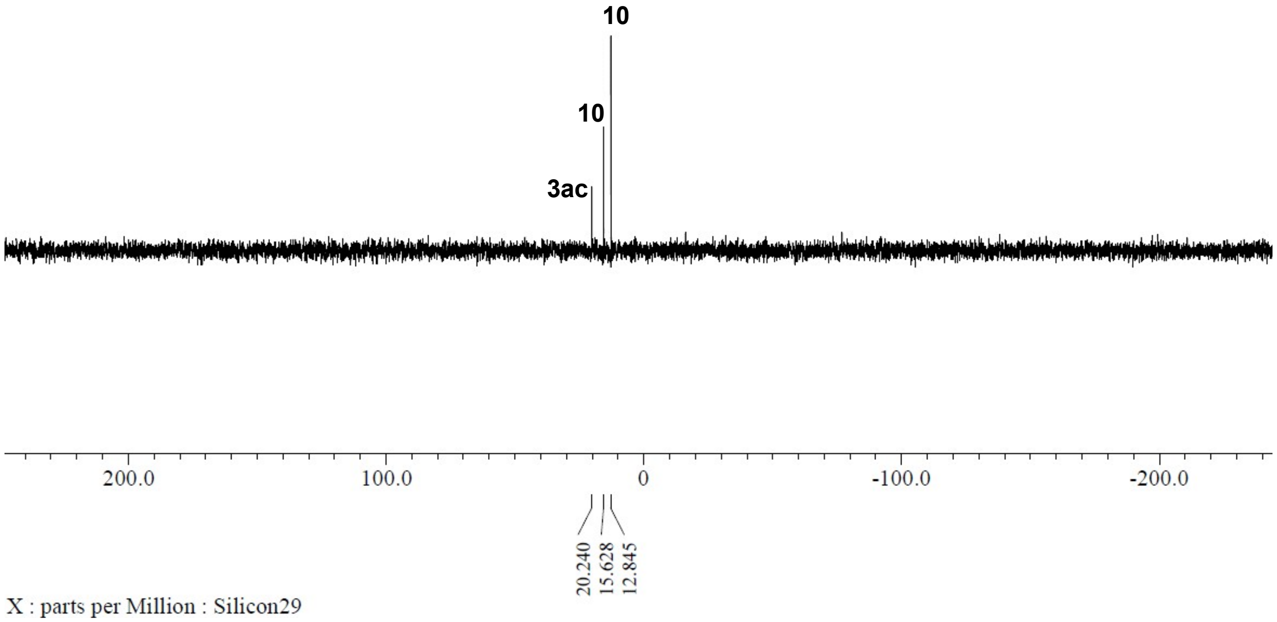
10
10
3ac
200.0
100.0
0
-100.0
-200.0
20.240
15.628
12.845
X : parts per Million : Silicon29

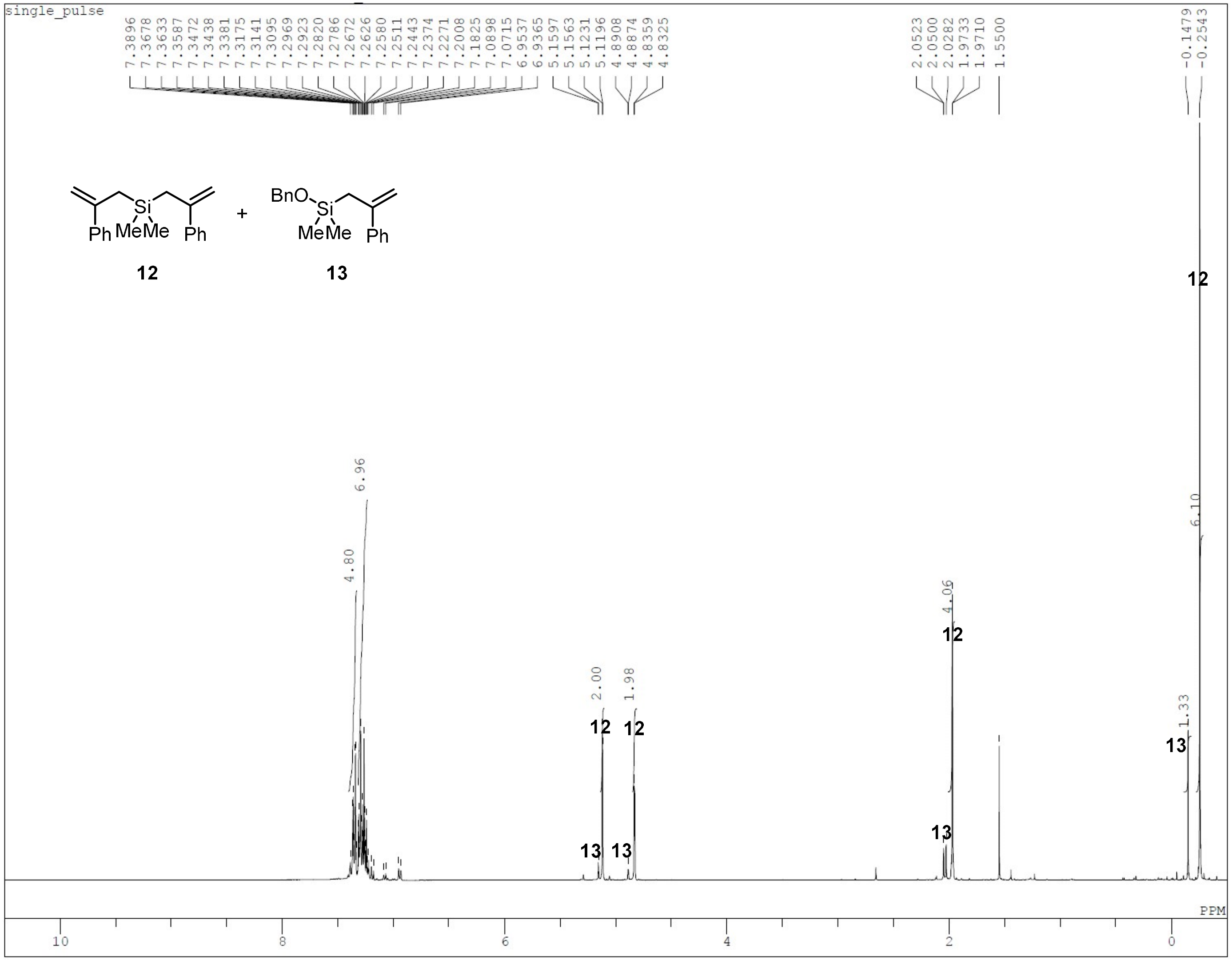
single_pulse
7.3896
7.3678
7.3633
7.3587
7.3472
7.3438
7.3381
7.3175
7.3141
7.3095
7.2969
7.2923
7.2820
7.2786
7.2672
7.2626
7.2580
7.2511
7.2443
7.2374
7.2271
7.2008
7.1825
7.0898
7.0715
6.9537
6.9365
5.1597
5.1563
5.1231
5.1196
4.8908
4.8874
4.8359
4.8325
2.0523
2.0500
2.0282
1.9733
1.9710
1.5500
-0.1479
-0.2543
BnO
Si
MeMe
Ph
12
13
+
6.96
4.80
2.00
1.98
4.06
6.10
1.33
PPM
10
8
6
4
2
0

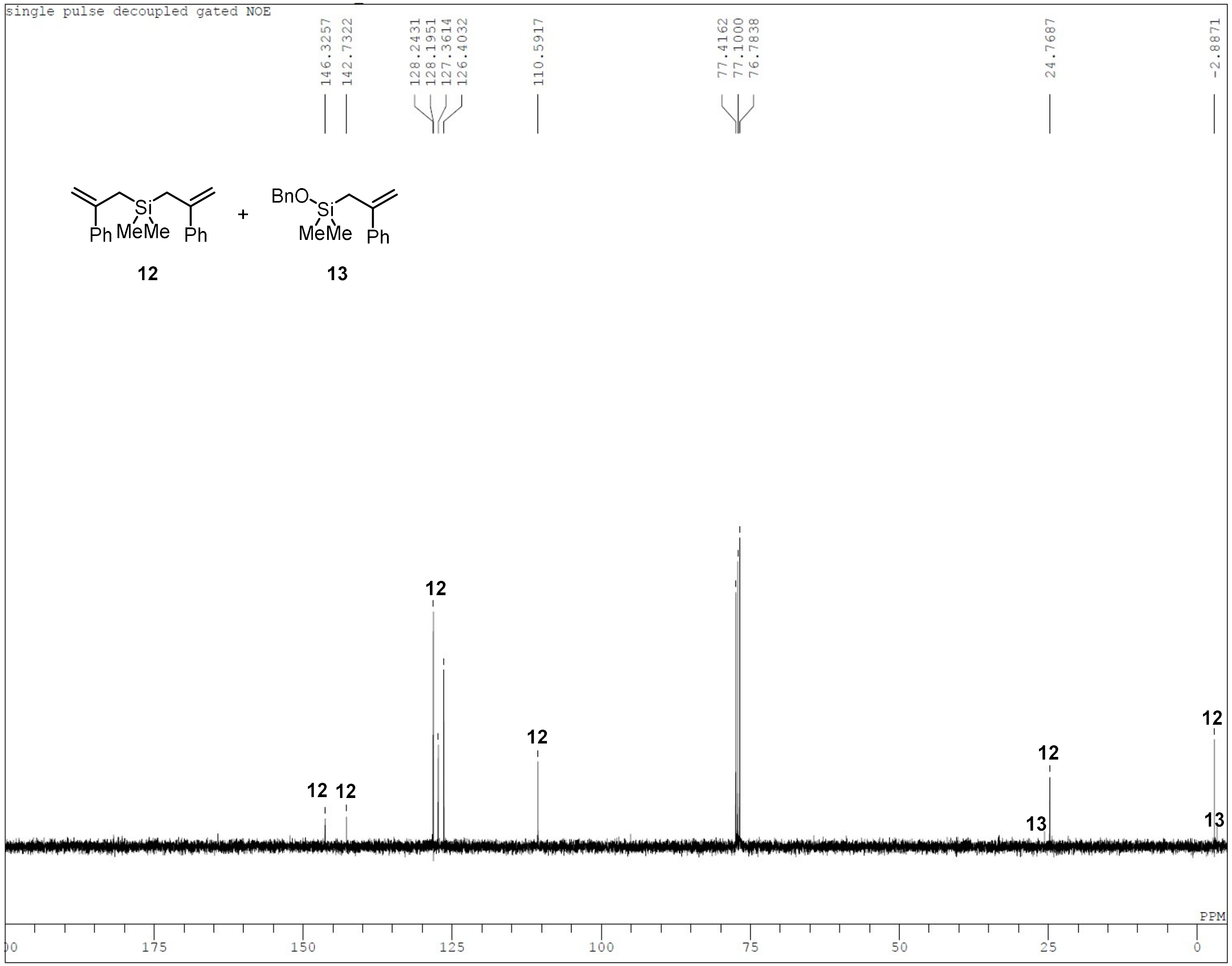
single pulse decoupled gated NOE
146.3257
142.7322
128.2431
128.1951
127.3614
126.4032
110.5917
77.4162
77.1000
76.7838
24.7687
-2.8871
Ph MeMe Ph
12
+
BnO
Si
MeMe Ph
13
PPM
175
150
125
100
75
50
25
0

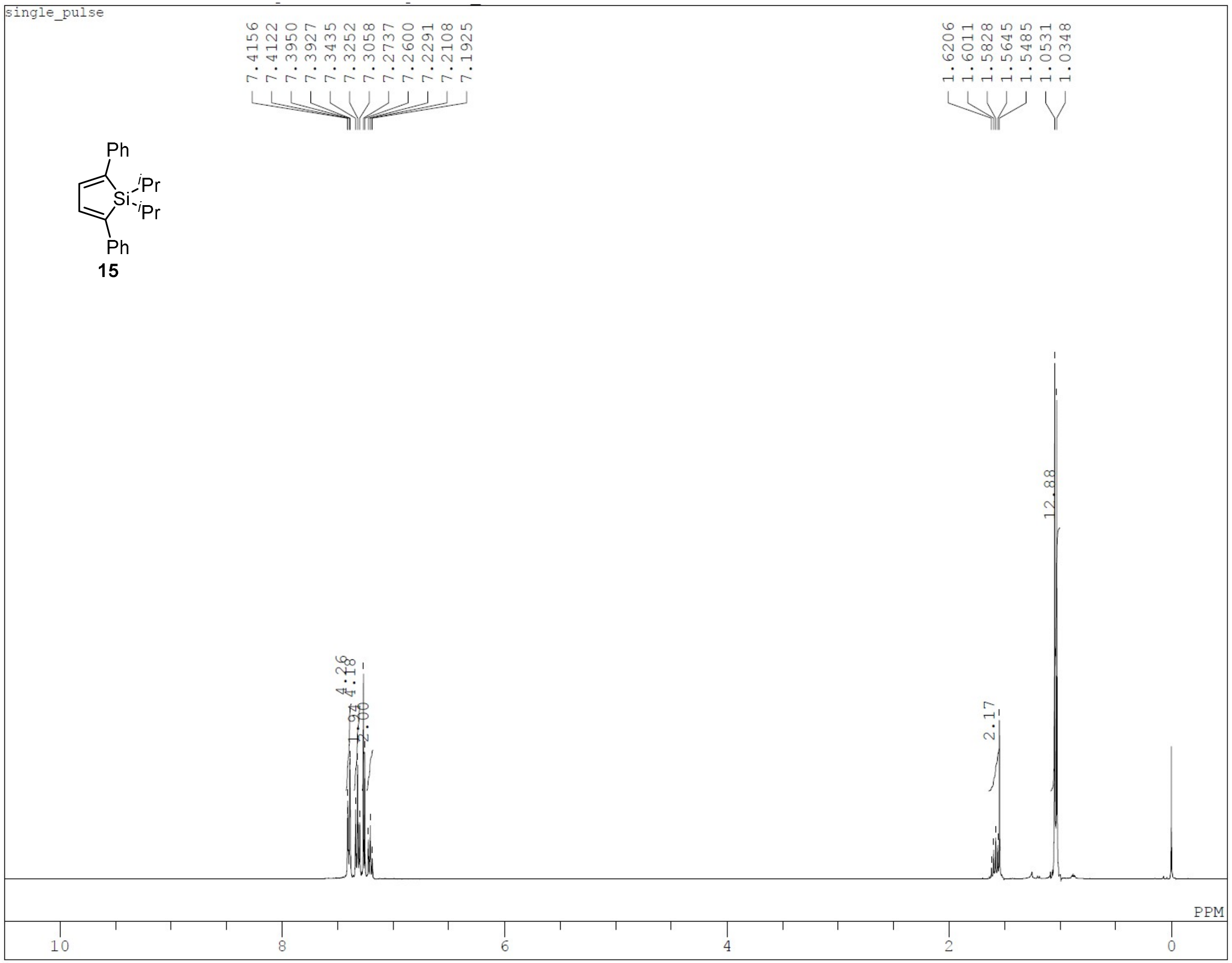
single_pulse
7.4156
7.4122
7.3950
7.3927
7.3435
7.3252
7.3058
7.2737
7.2600
7.2291
7.2108
7.1925
1.6206
1.6011
1.5828
1.5645
1.5485
1.0531
1.0348
Ph
Si
ⁱPr
ⁱPr
Ph
15
12.88
4.26
4.18
1.94
2.00
2.17
PPM
10
8
6
4
2
0

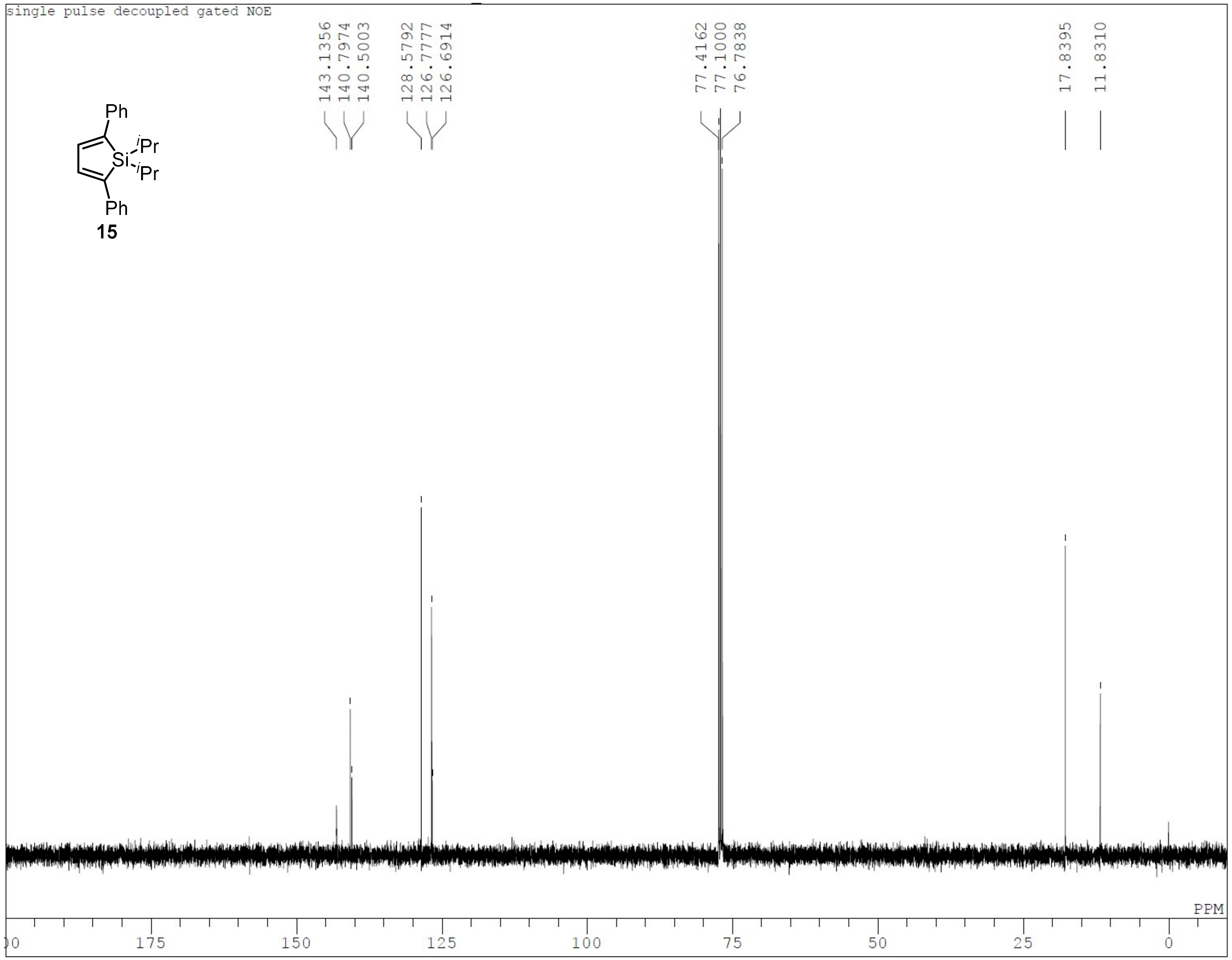
single pulse decoupled gated NOE
143.1356
140.7974
140.5003
128.5792
126.7777
126.6914
77.4162
77.1000
76.7838
17.8395
11.8310
Ph
Si
^{i}Pr
^{i}Pr
Ph
15
PPM
175
150
125
100
75
50
25
0

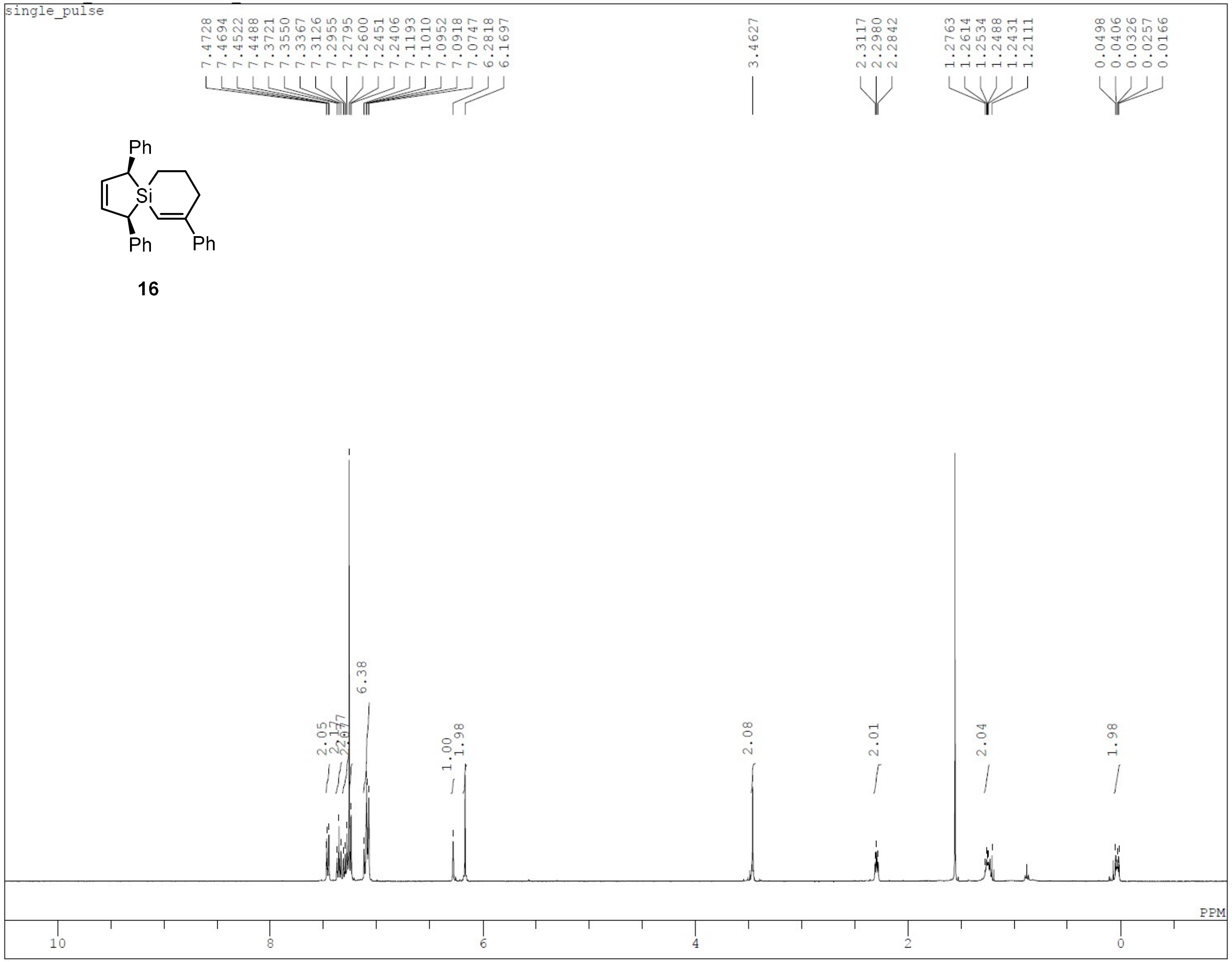
single_pulse
7.4728
7.4694
7.4522
7.4488
7.3721
7.3550
7.3367
7.3126
7.2955
7.2795
7.2600
7.2451
7.2406
7.1193
7.1010
7.0952
7.0918
7.0747
6.2818
6.1697
3.4627
2.3117
2.2980
2.2842
1.2763
1.2614
1.2534
1.2488
1.2431
1.2111
0.0498
0.0406
0.0326
0.0257
0.0166
Ph
Si
Ph
Ph
6.38
2.05
2.17
2.07
1.00
1.98
2.08
2.01
2.04
1.98
PPM
10
8
6
4
2
0

16

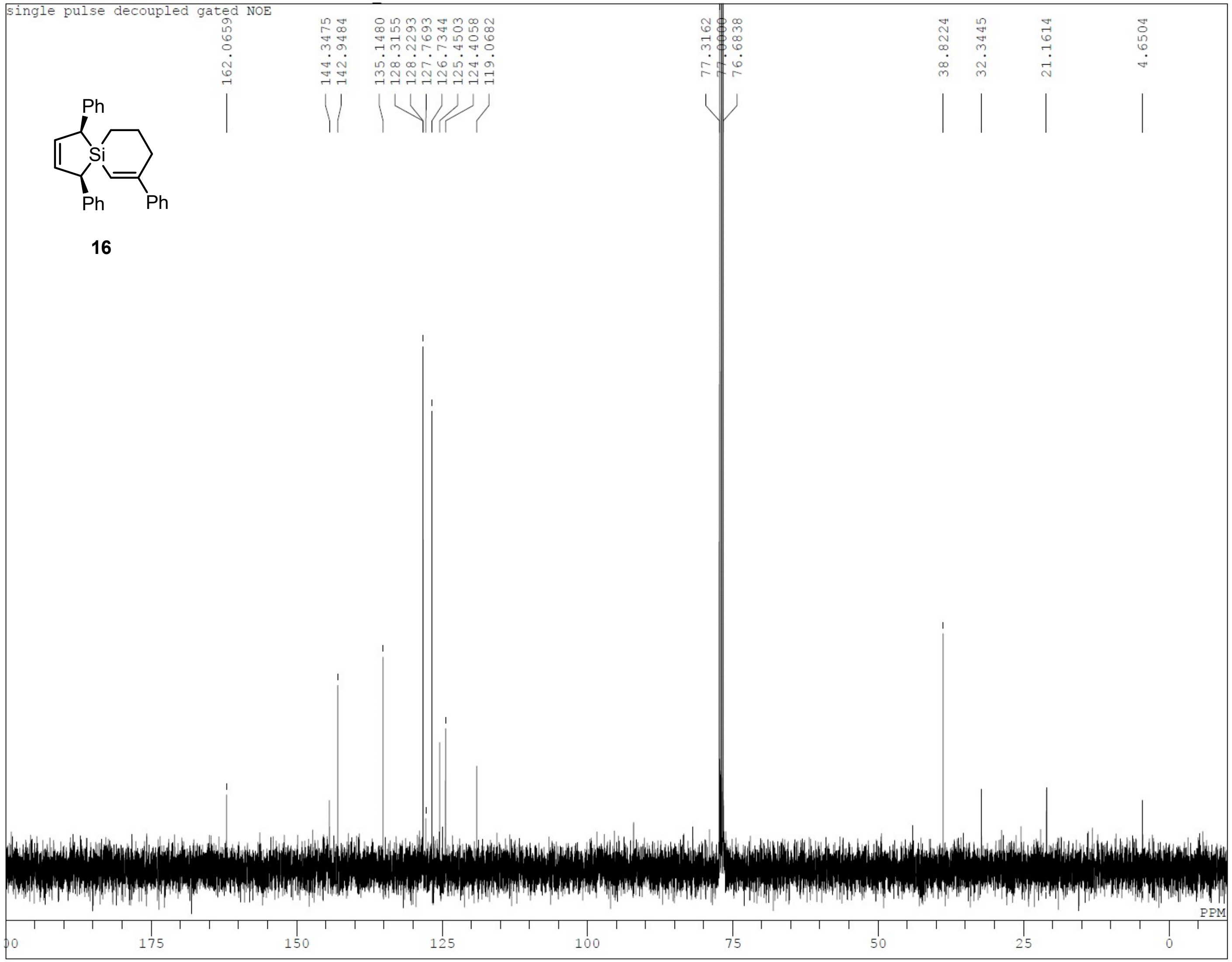
single pulse decoupled gated NOE
162.0659
144.3475
142.9484
135.1480
128.3155
128.2293
127.7693
126.7344
125.4503
124.4058
119.0682
77.3162
77.0000
76.6838
38.8224
32.3445
21.1614
4.6504
Ph
Si
Ph
Ph
PPM
175
150
125
100
75
50
25
0

16

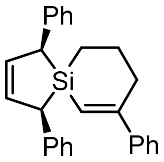

16

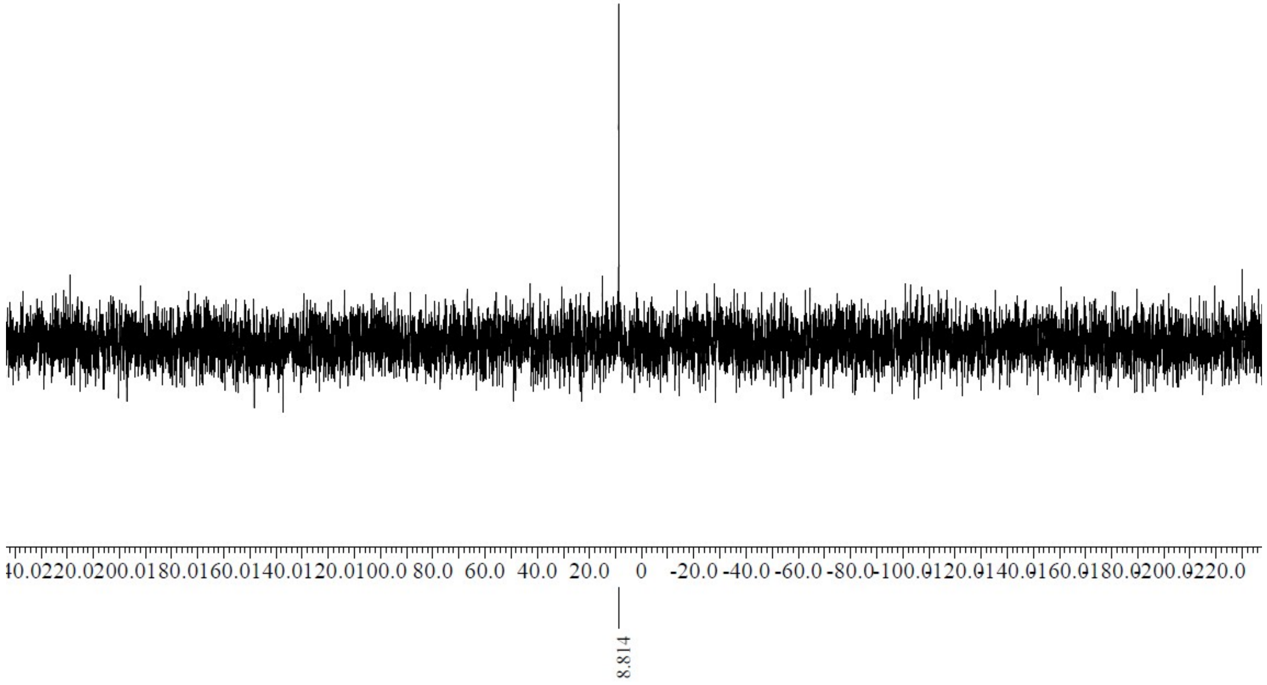

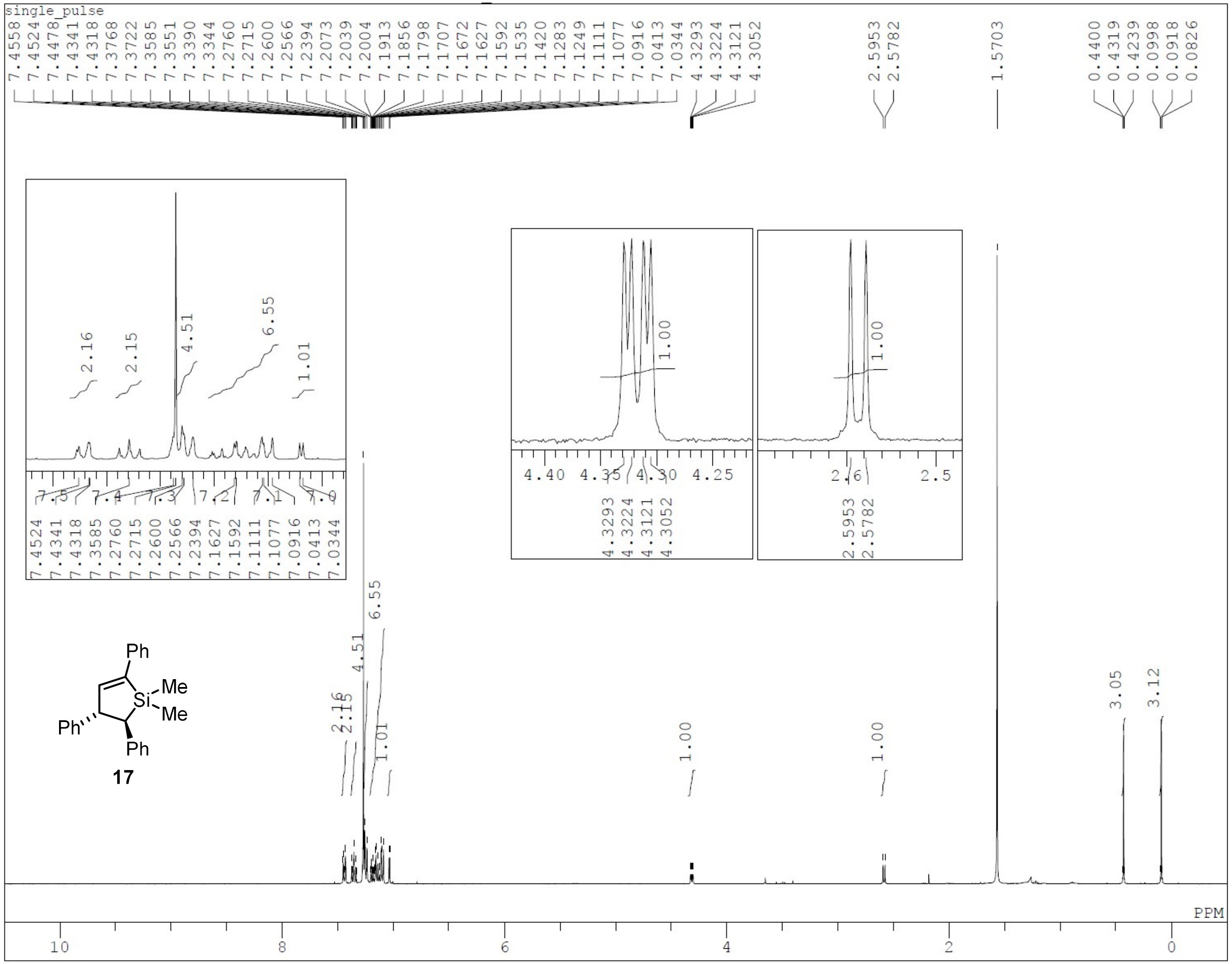

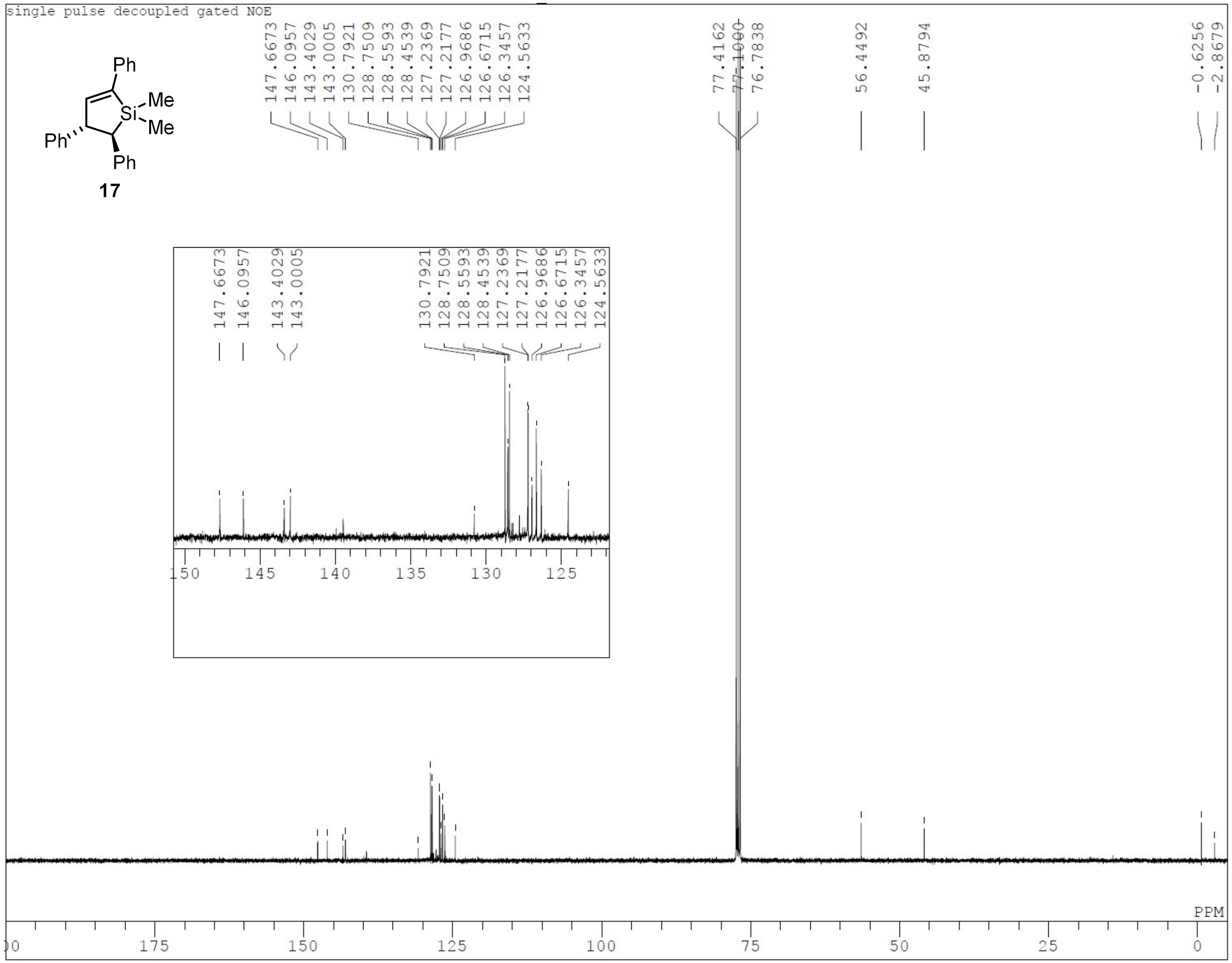
single pulse decoupled gated NOE
Ph
Si
Me
Me
Ph
Ph
17
147.6673
146.0957
143.4029
143.0005
130.7921
128.7509
128.5593
128.4539
127.2369
127.2177
126.9686
126.6715
126.3457
124.5633
77.4162
77.1000
76.7838
56.4492
45.8794
-0.6256
-2.8679
PPM

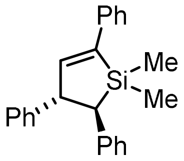
Ph
Me
Si
Me
Ph
Ph

17

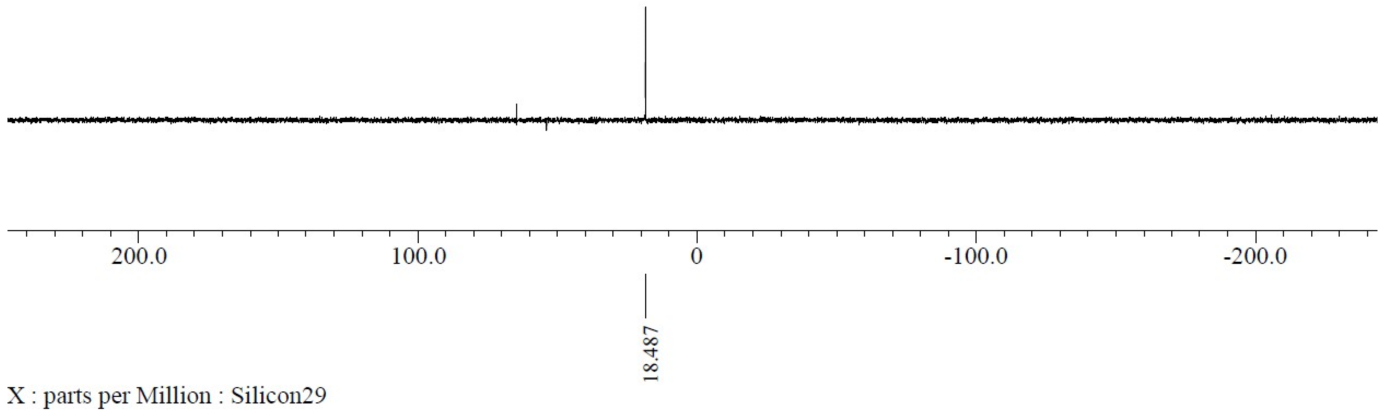
200.0
100.0
0
-100.0
-200.0
18.487
X : parts per Million : Silicon29

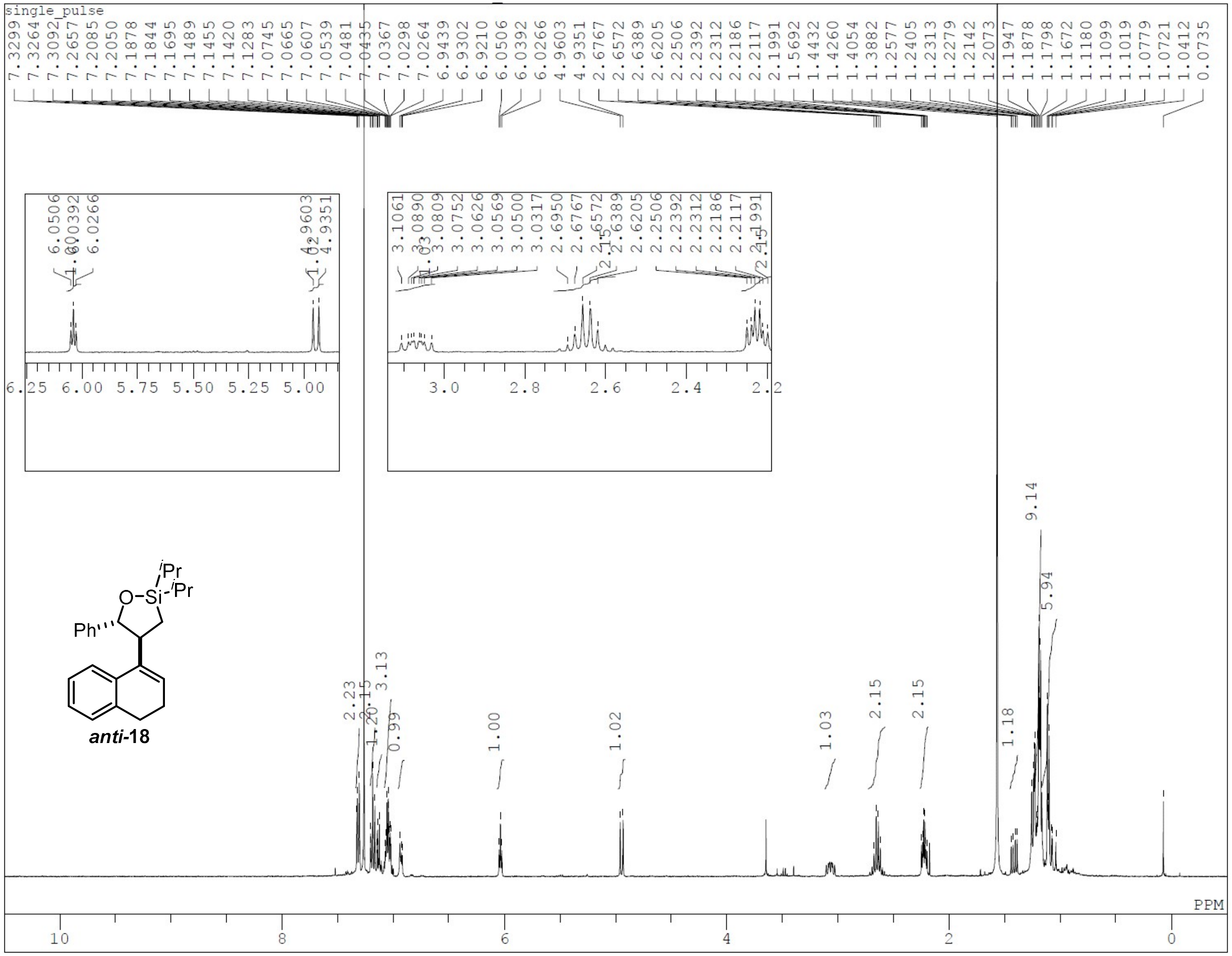
single_pulse
PPM
anti-18

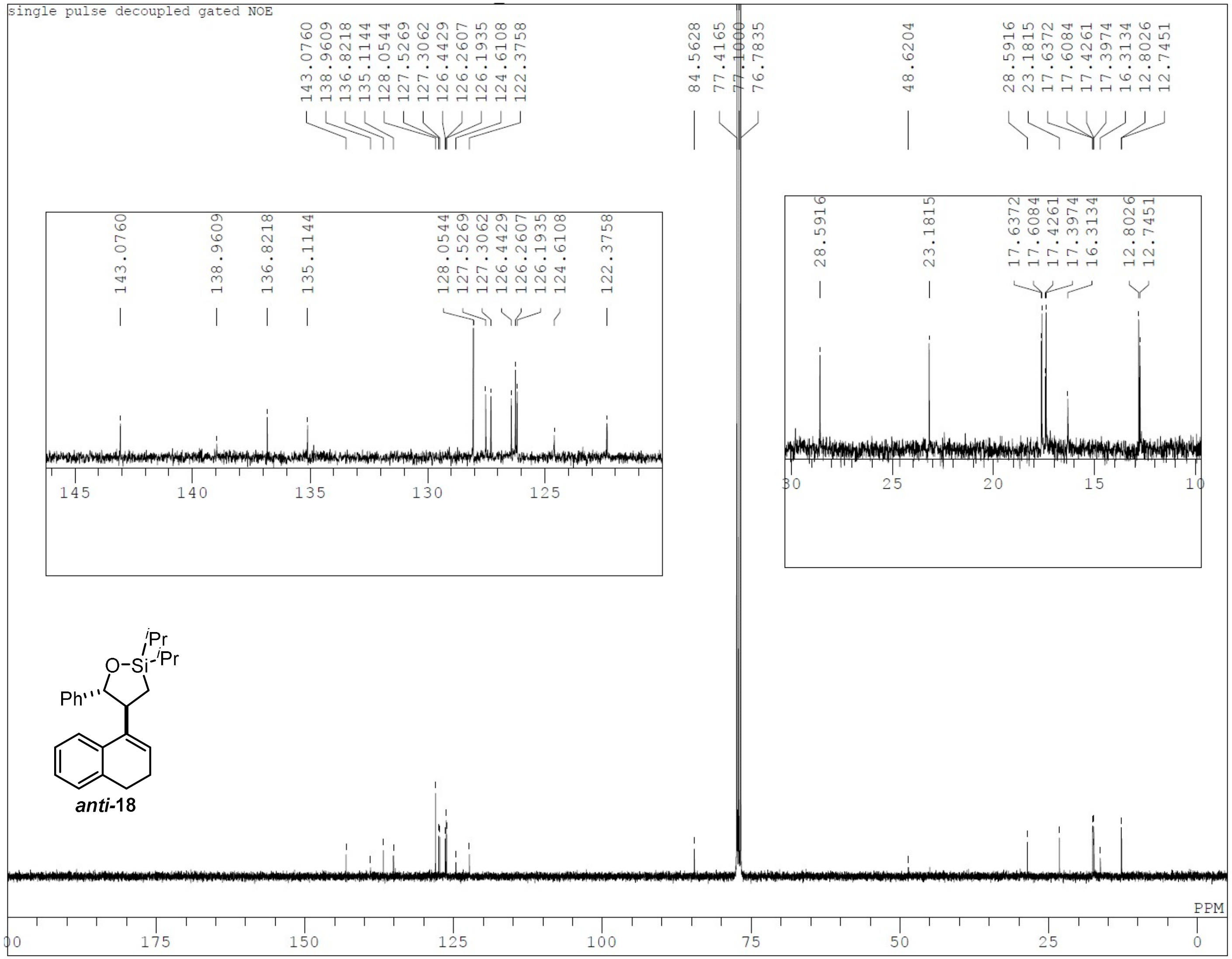
single pulse decoupled gated NOE
143.0760
138.9609
136.8218
135.1144
128.0544
127.5269
127.3062
126.4429
126.2607
126.1935
124.6108
122.3758
84.5628
77.4165
77.1000
76.7835
48.6204
28.5916
23.1815
17.6372
17.6084
17.4261
17.3974
16.3134
12.8026
12.7451
145 140 135 130 125
30 25 20 15 10
PPM
175 150 125 100 75 50 25 0
iPr
iPr
O-Si
Ph
anti-18

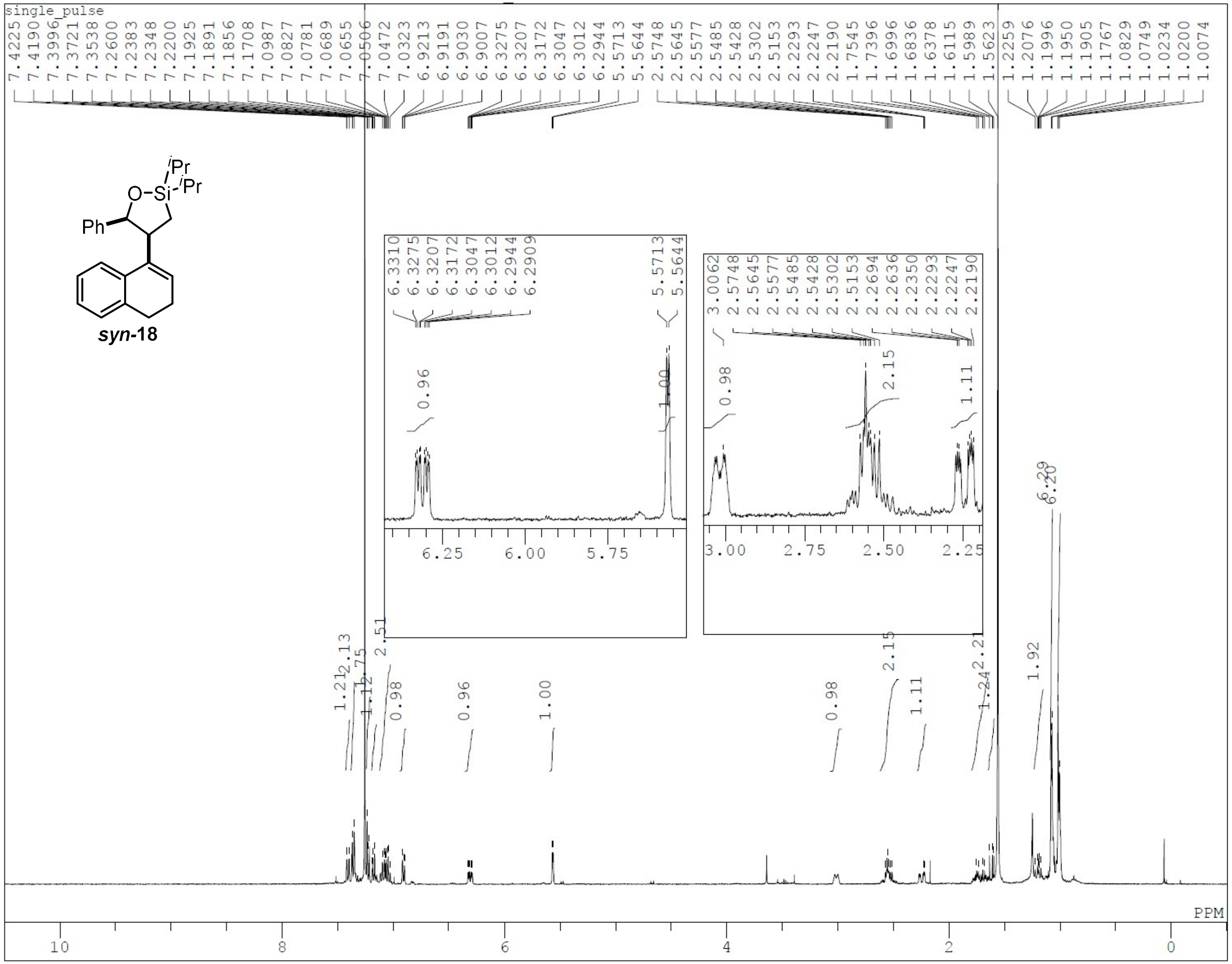

single_pulse
syn-18
PPM

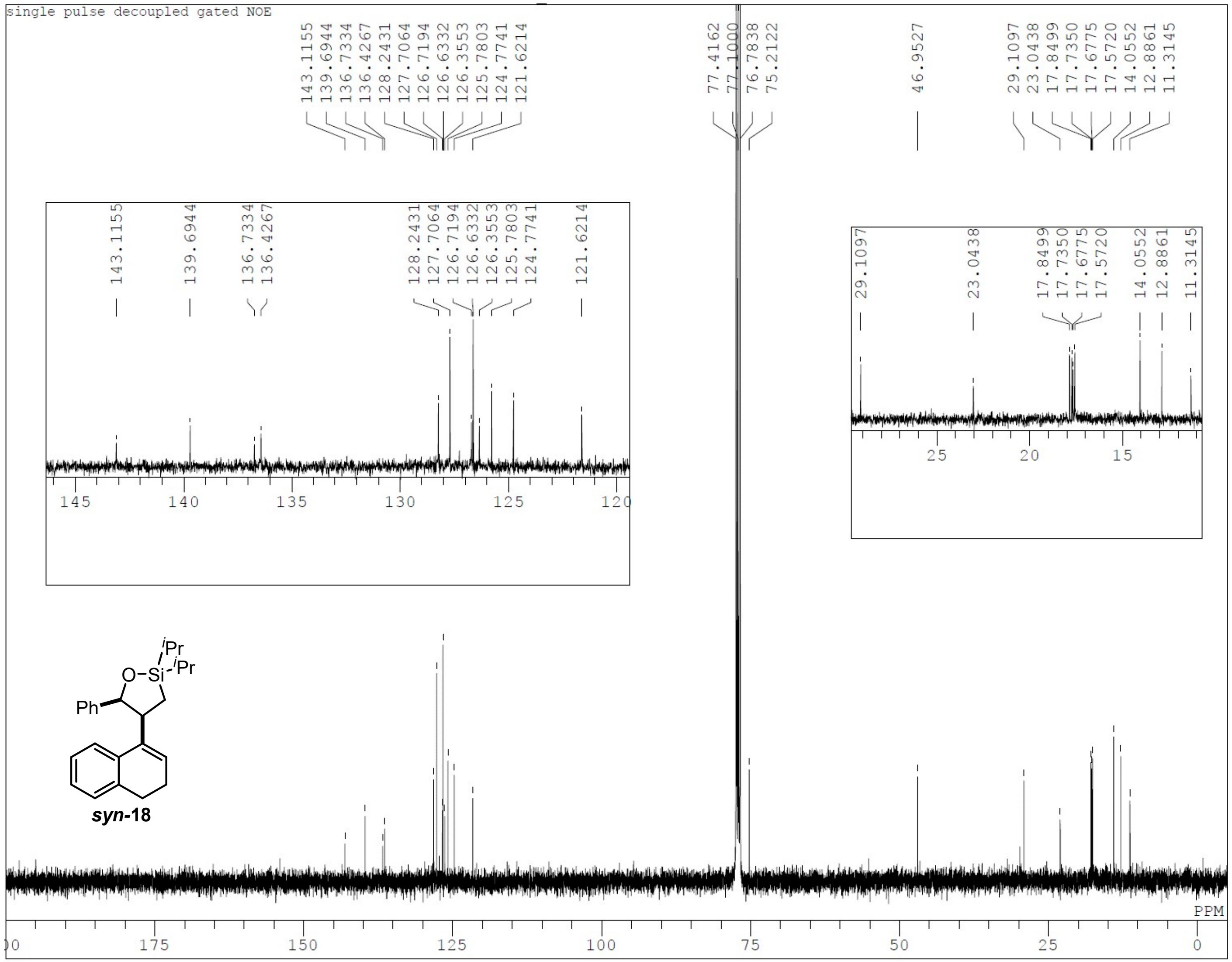
single pulse decoupled gated NOE
143.1155
139.6944
136.7334
136.4267
128.2431
127.7064
126.7194
126.6332
126.3553
125.7803
124.7741
121.6214
77.4162
77.1000
76.7838
75.2122
46.9527
29.1097
23.0438
17.8499
17.7350
17.6775
17.5720
14.0552
12.8861
11.3145
PPM
Ph
O
Si
iPr
iPr
syn-18

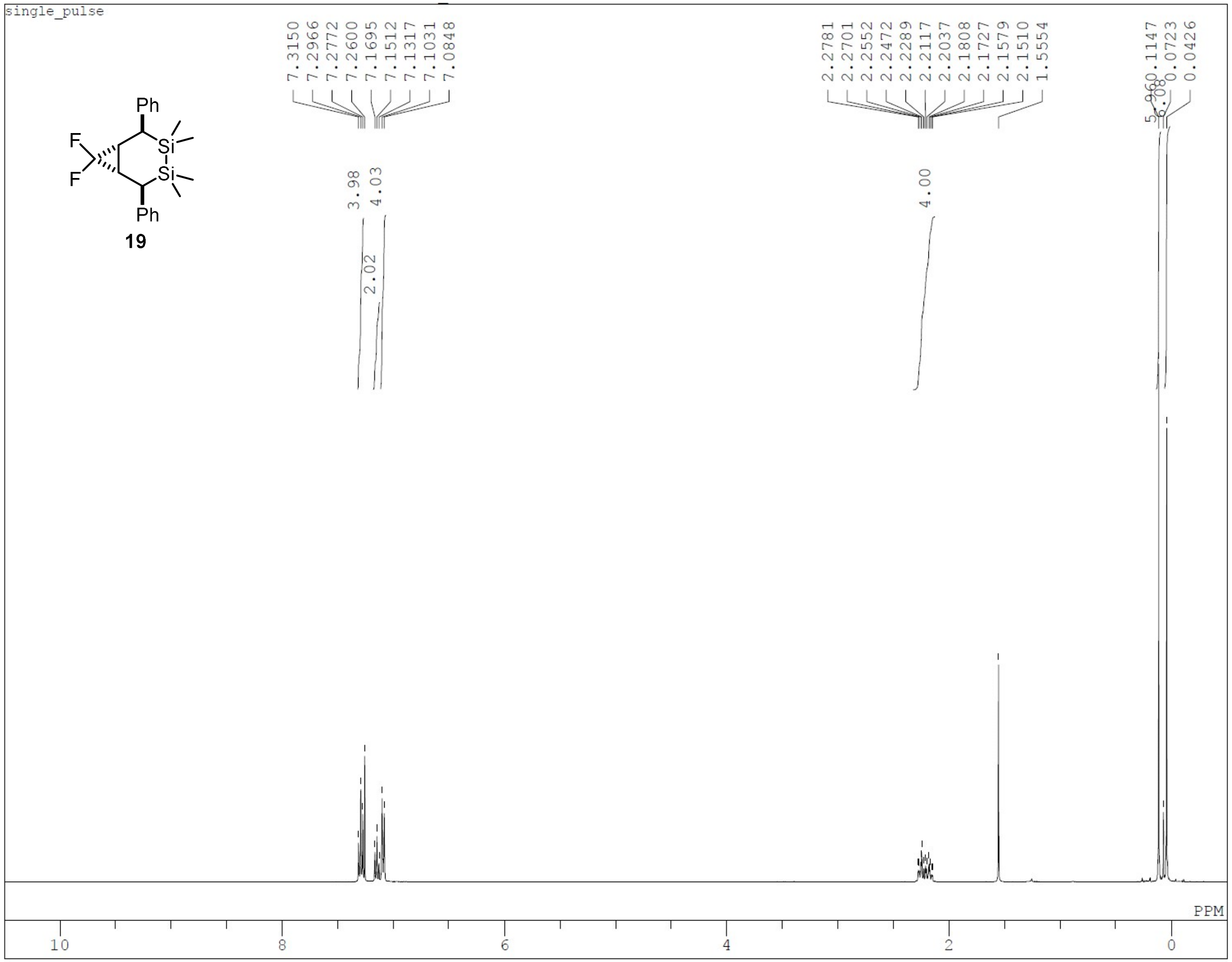
single_pulse
Ph
F
F
Si
Si
Ph
19
7.3150
7.2966
7.2772
7.2600
7.1695
7.1512
7.1317
7.1031
7.0848
2.2781
2.2701
2.2552
2.2472
2.2289
2.2117
2.2037
2.1808
2.1727
2.1579
2.1510
1.5554
0.1147
0.0723
0.0426
3.98
4.03
2.02
4.00
5.96
6.08
PPM
10
8
6
4
2
0

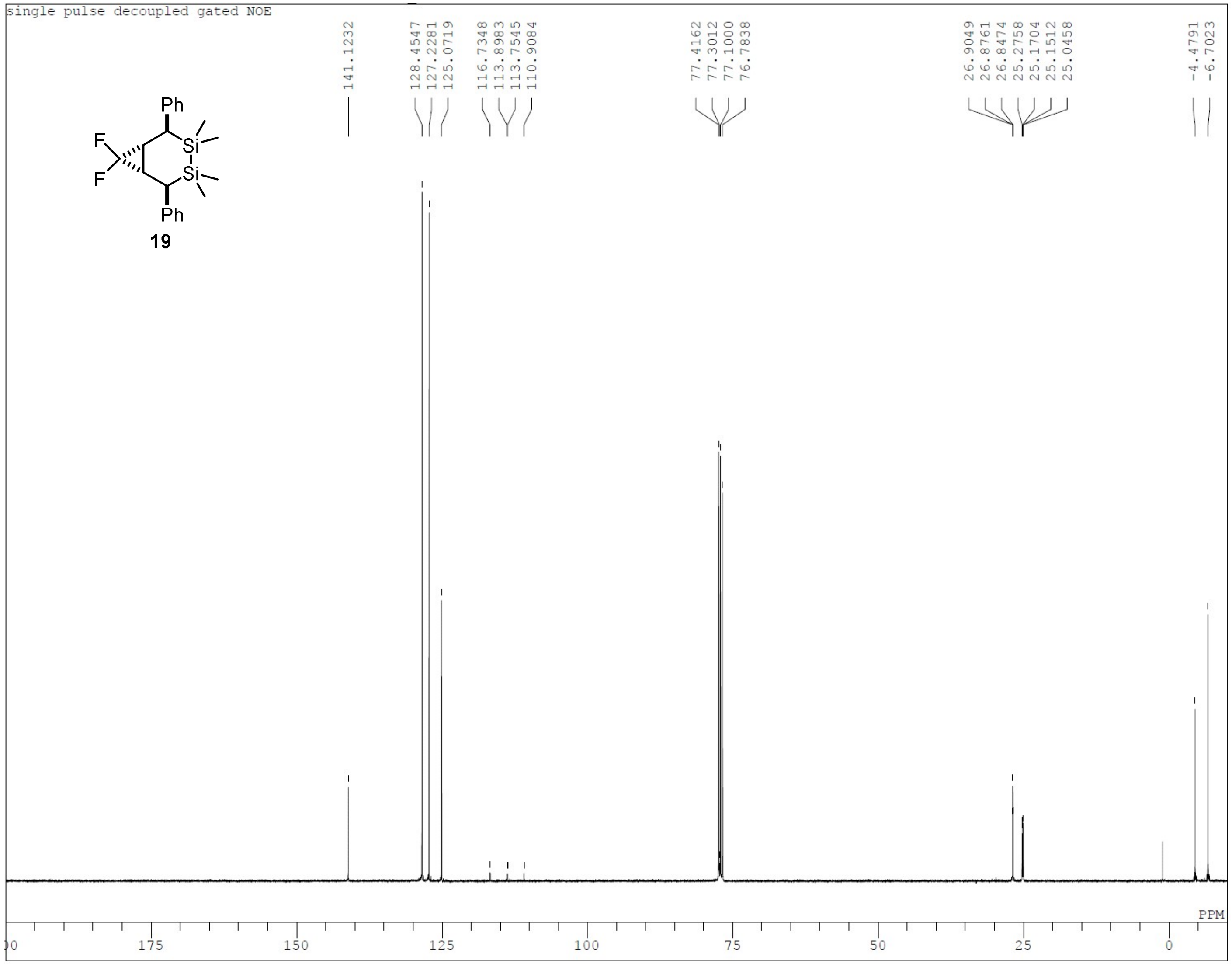
single pulse decoupled gated NOE
141.1232
128.4547
127.2281
125.0719
116.7348
113.8983
113.7545
110.9084
77.4162
77.3012
77.1000
76.7838
26.9049
26.8761
26.8474
25.2758
25.1704
25.1512
25.0458
-4.4791
-6.7023
Ph
F
F
Si
Si
Ph
19
PPM
175
150
125
100
75
50
25
0

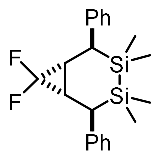
Ph
F
Si
Si
F
Ph

19

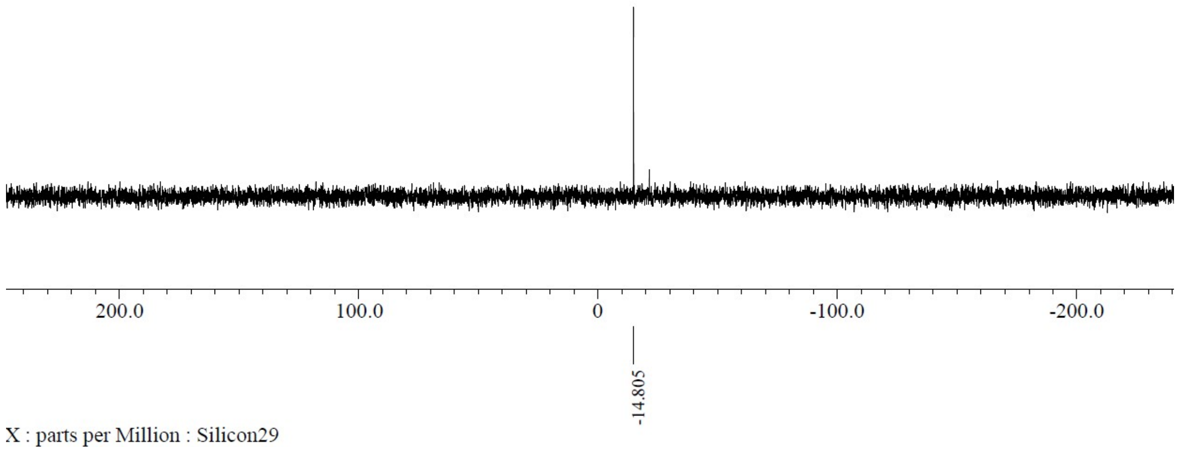
200.0
100.0
0
-100.0
-200.0
-14.805
X : parts per Million : Silicon29

19; $^{1}H/^{13}C$ HMQC NMR

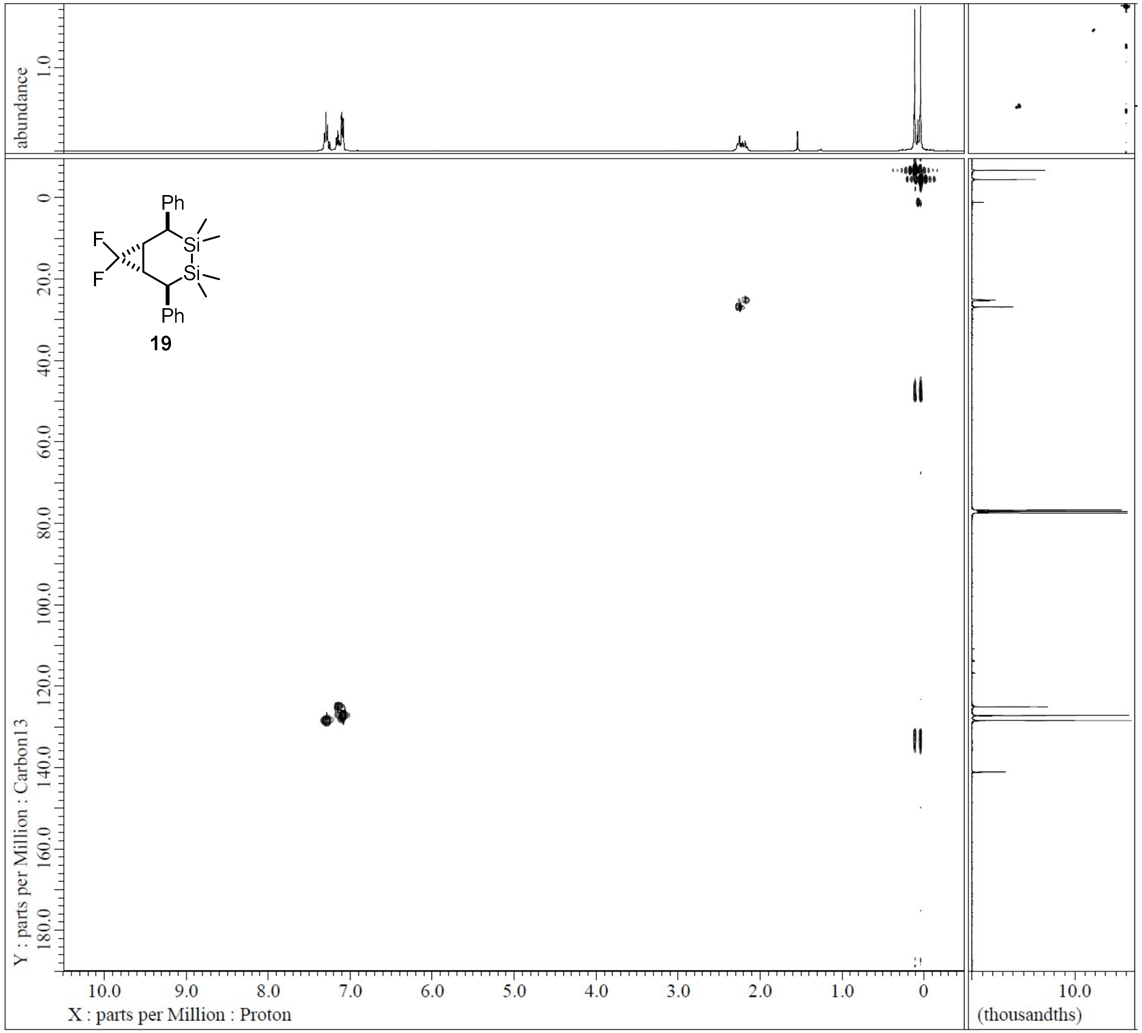

19; $^{1}H/^{13}C$ HMQC NMR (zoomed-1)

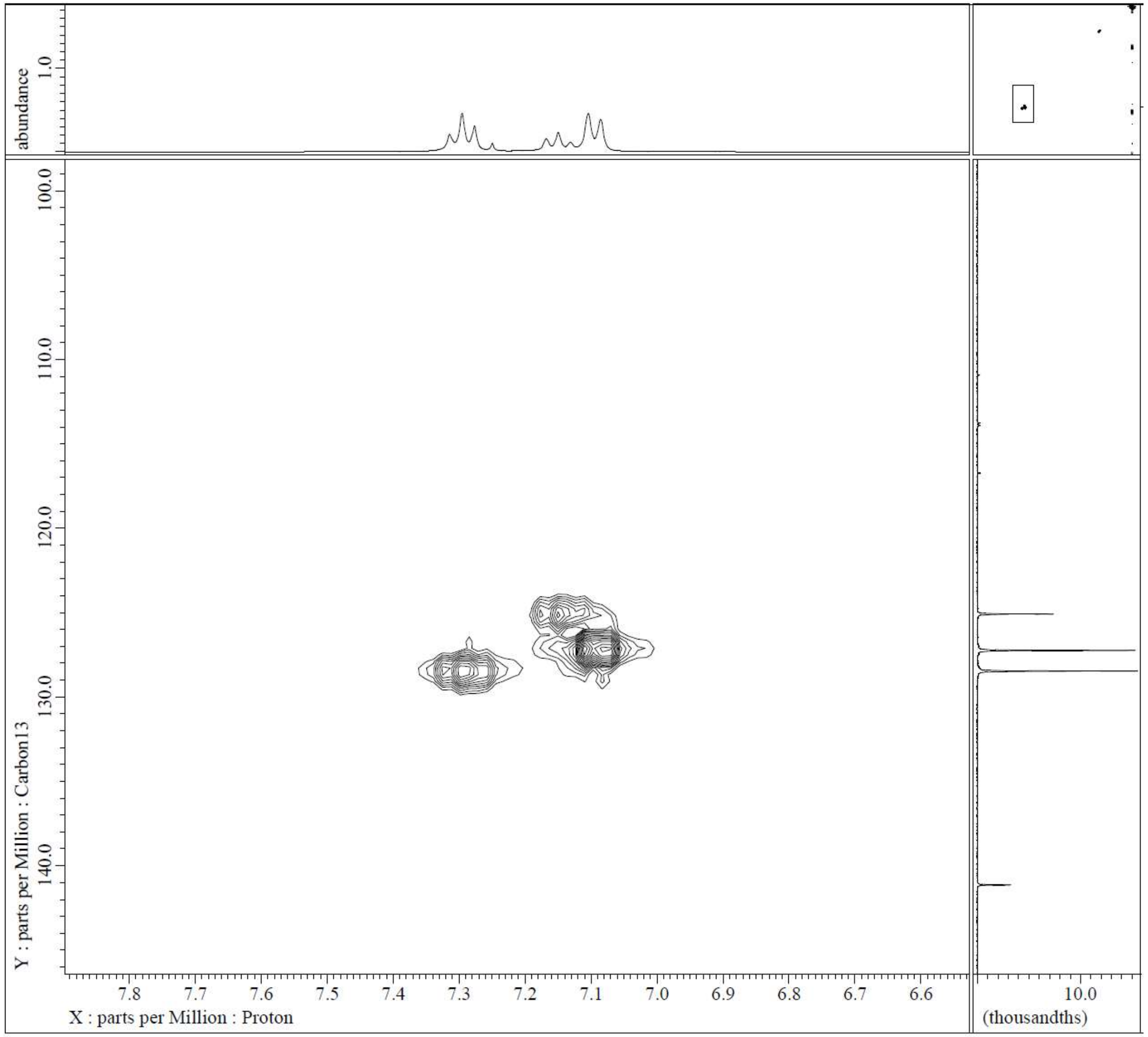

19; $^{1}H/^{13}C$ HMQC NMR (zoomed-2)

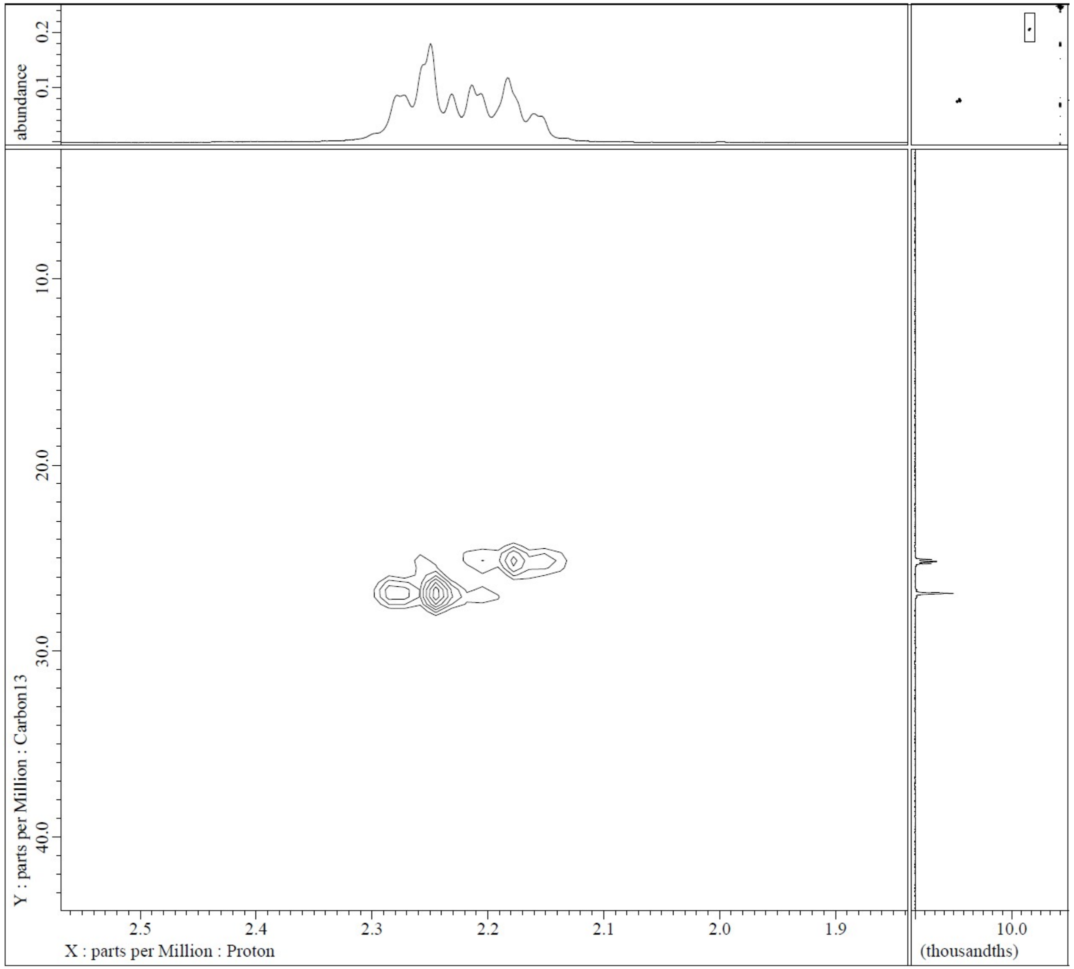

19; $^{1}H/^{13}C$ HMBC NMR

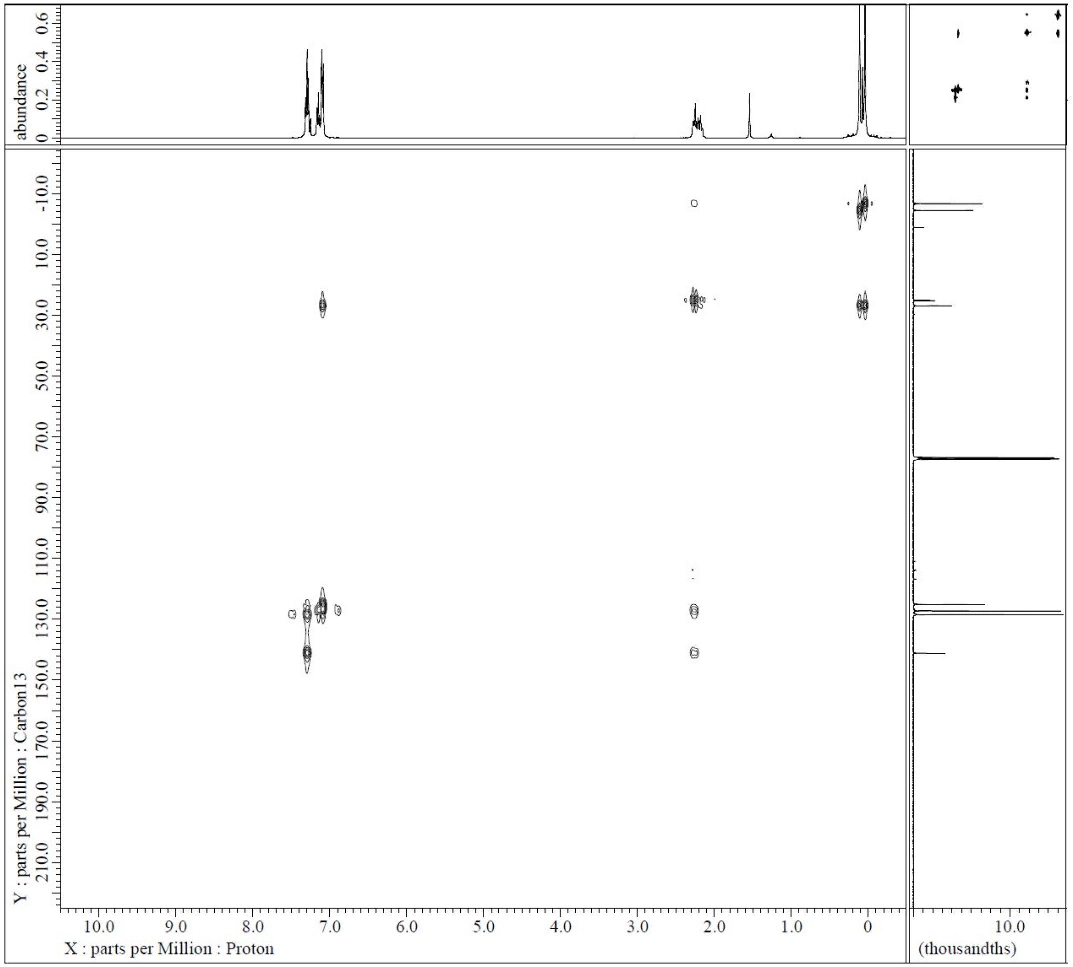

19; $^1H/^{13}C$ HMBC NMR (zoomed)

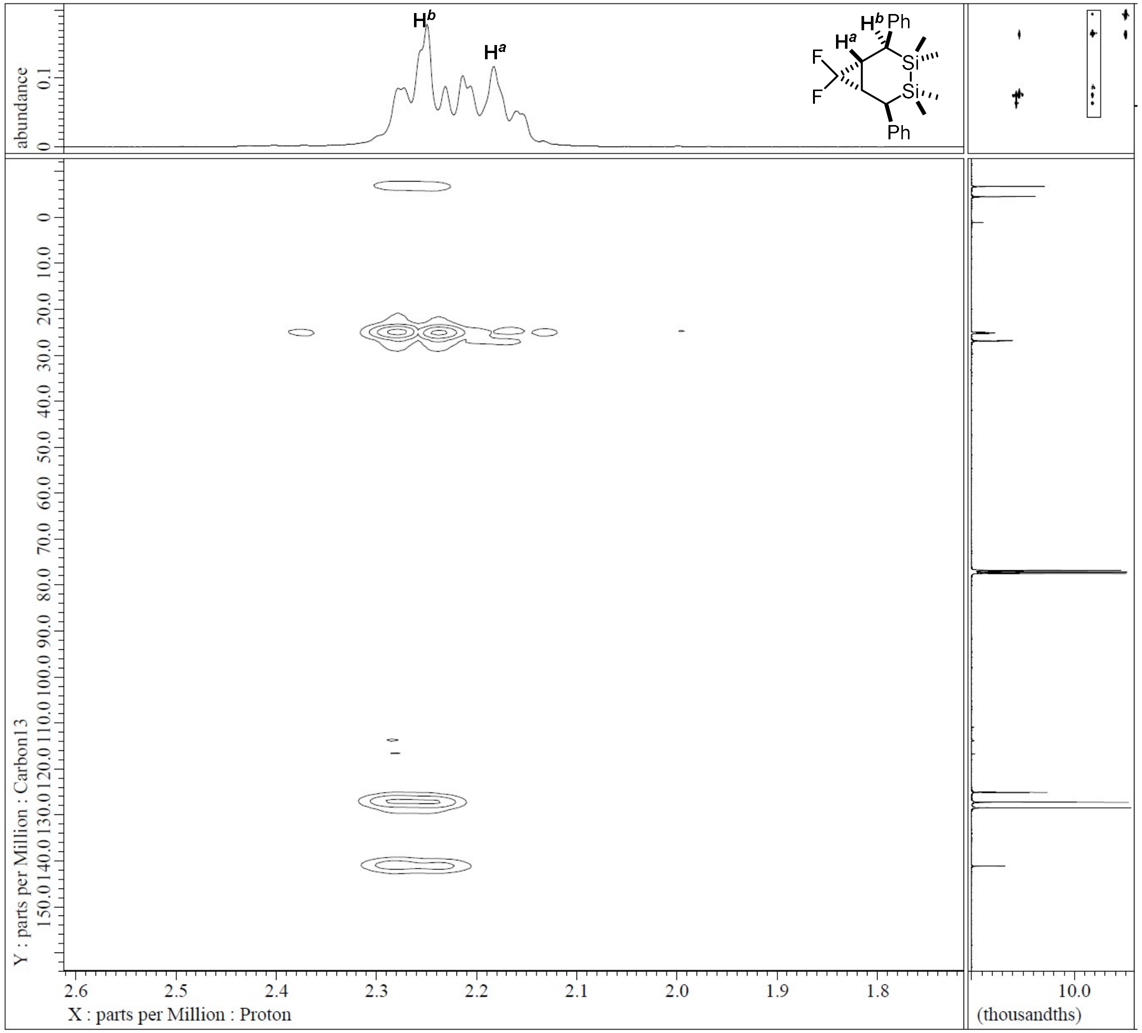

19; NOE

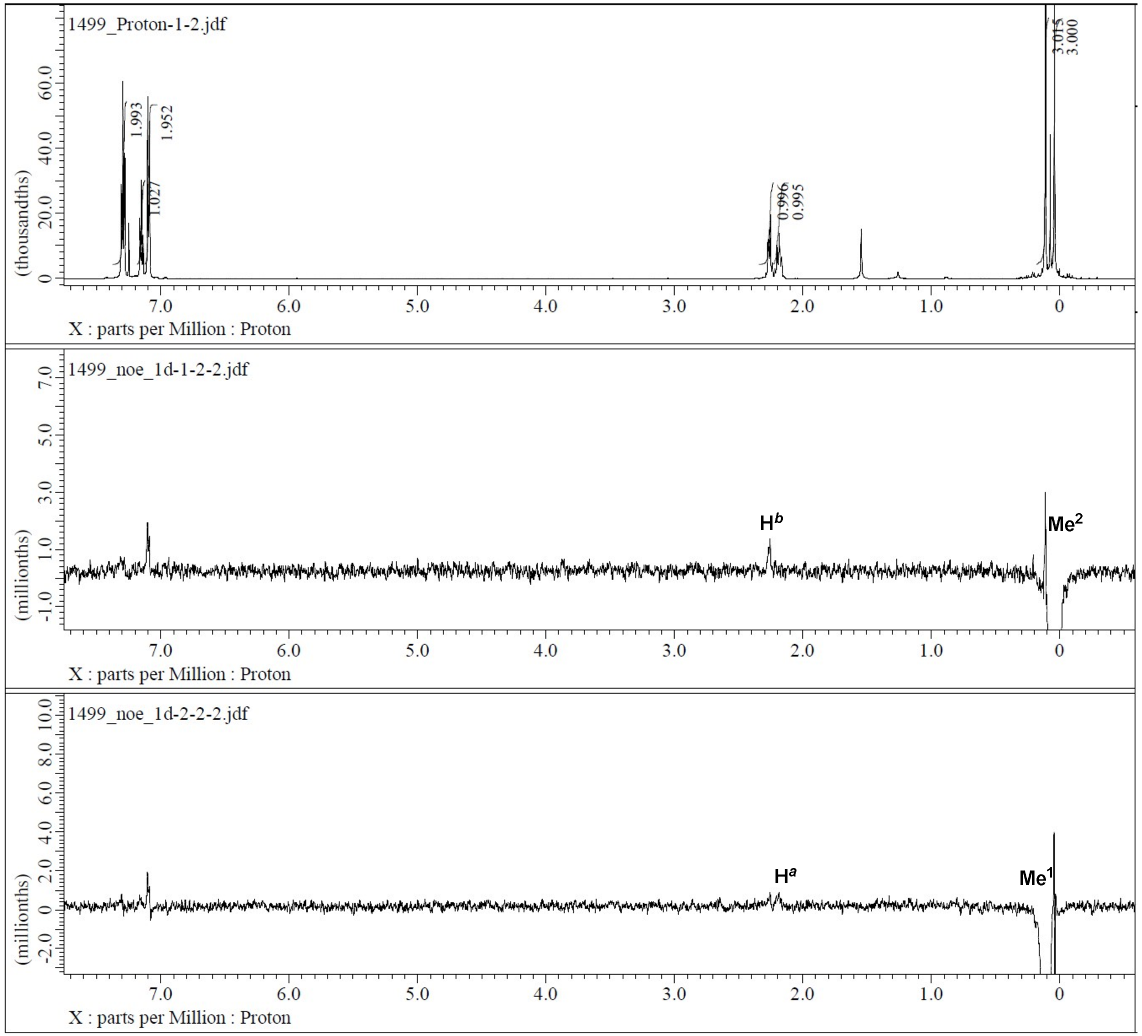
1499_Proton-1-2.jdf
(thousandths)
X : parts per Million : Proton
1499_noe_1d-1-2-2.jdf
(millionths)
H^b
Me^2
X : parts per Million : Proton
1499_noe_1d-2-2-2.jdf
(millionths)
H^a
Me^1
X : parts per Million : Proton

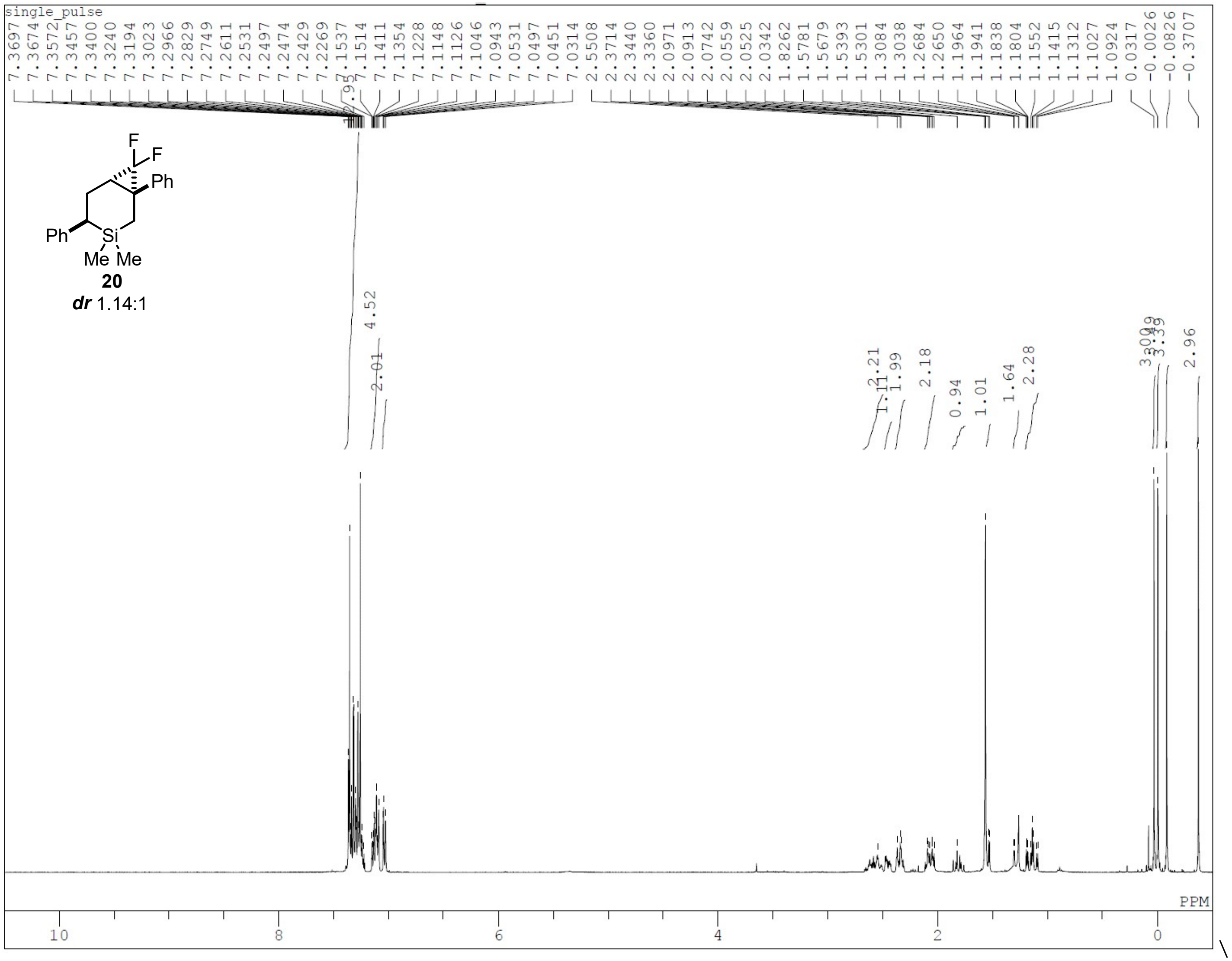
single_pulse
F
F
Ph
Ph
Si
Me Me
20
dr 1.14:1
PPM
10
8
6
4
2
0

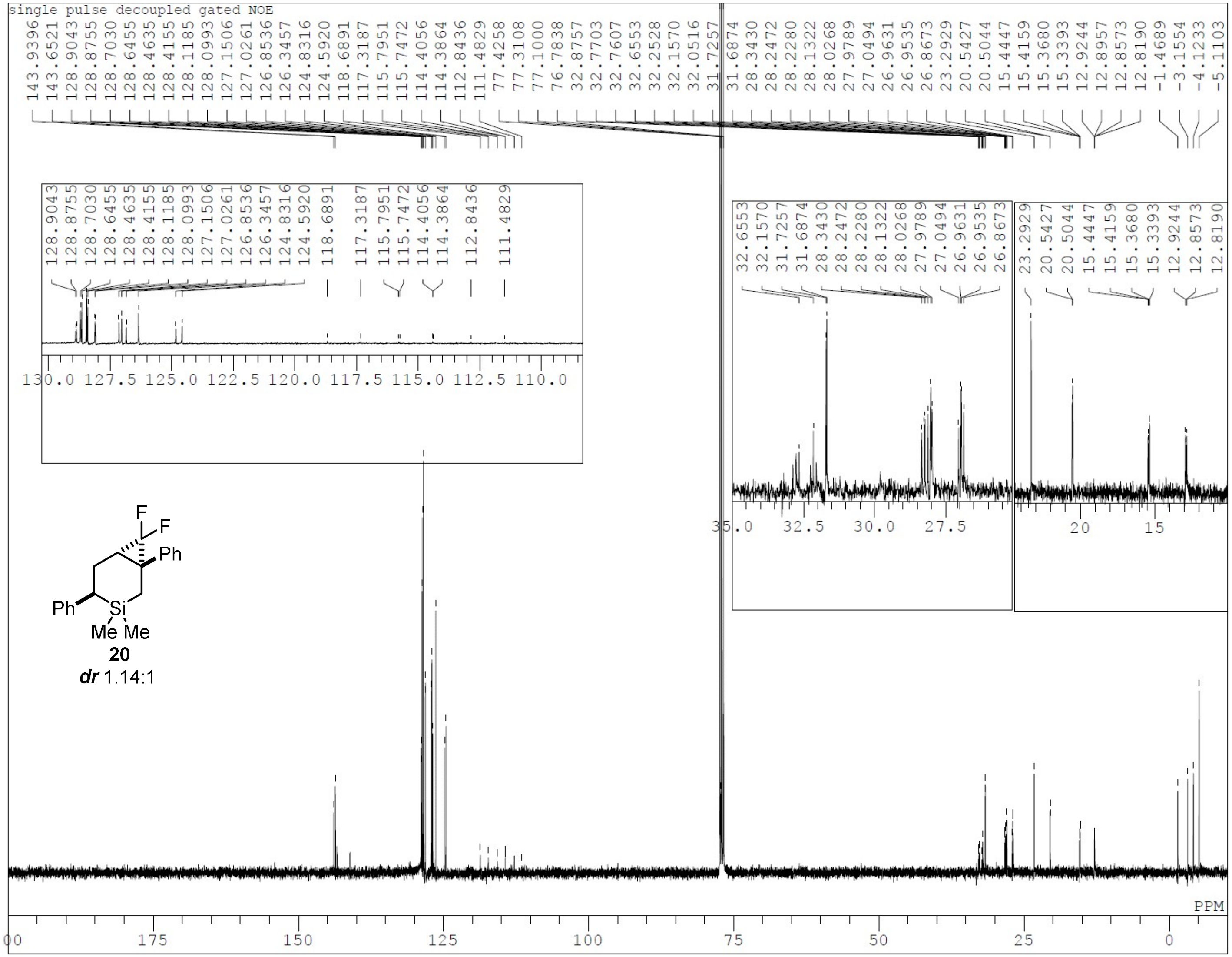
single pulse decoupled gated NOE
143.9396 143.6521 128.9043 128.8755 128.7030 128.6455 128.4635 128.4155 128.1185 128.0993 127.1506 127.0261 126.8536 126.3457 124.8316 124.5920 118.6891 117.3187 115.7951 115.7472 114.4056 114.3864 112.8436 111.4829 77.4258 77.3108 77.1000 76.7838 32.8757 32.7703 32.7607 32.6553 32.2528 32.1570 32.0516 31.7257 31.6874 28.3430 28.2472 28.2280 28.1322 28.0268 27.9789 27.0494 26.9631 26.9535 26.8673 23.2929 20.5427 20.5044 15.4447 15.4159 15.3680 15.3393 12.9244 12.8957 12.8573 12.8190 -1.4689 -3.1554 -4.1233 -5.1103
F
F
Ph
Ph
Si
Me Me
20
dr 1.14:1
PPM
175
150
125
100
75
50
25
0

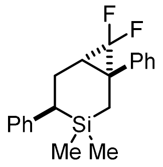
F
F
Ph
Ph
Si
Me Me

20

dr 1.14:1

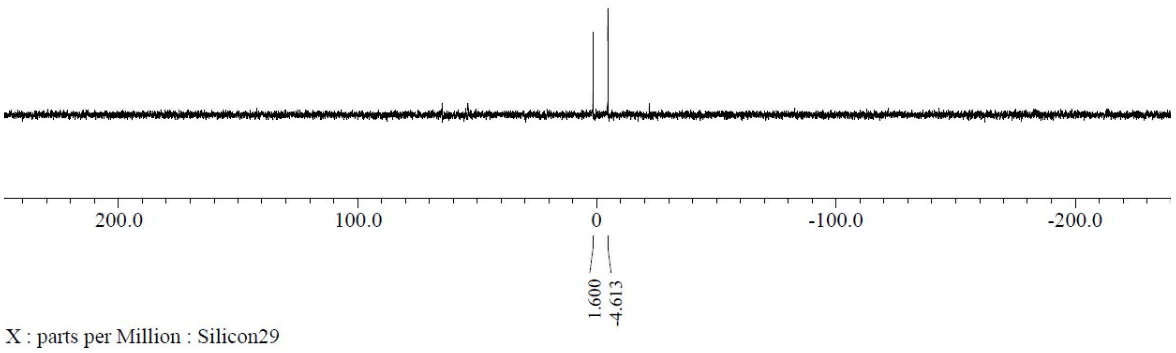
200.0
100.0
0
-100.0
-200.0
1.600
-4.613
X : parts per Million : Silicon29

20; $^1H/^{13}C$ HSQC NMR

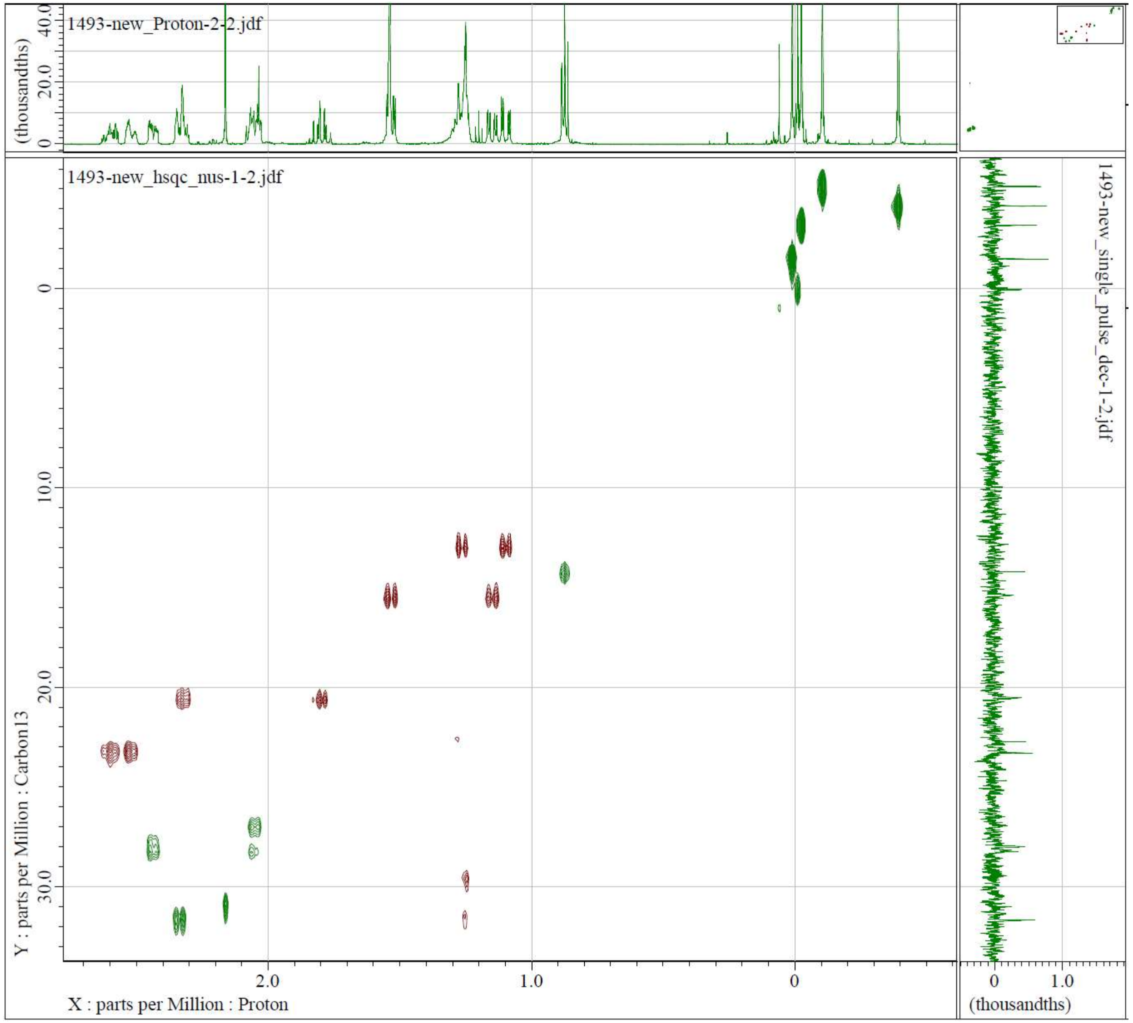

20; $^{1}H/^{13}C$ HSQC NMR (zoomed)

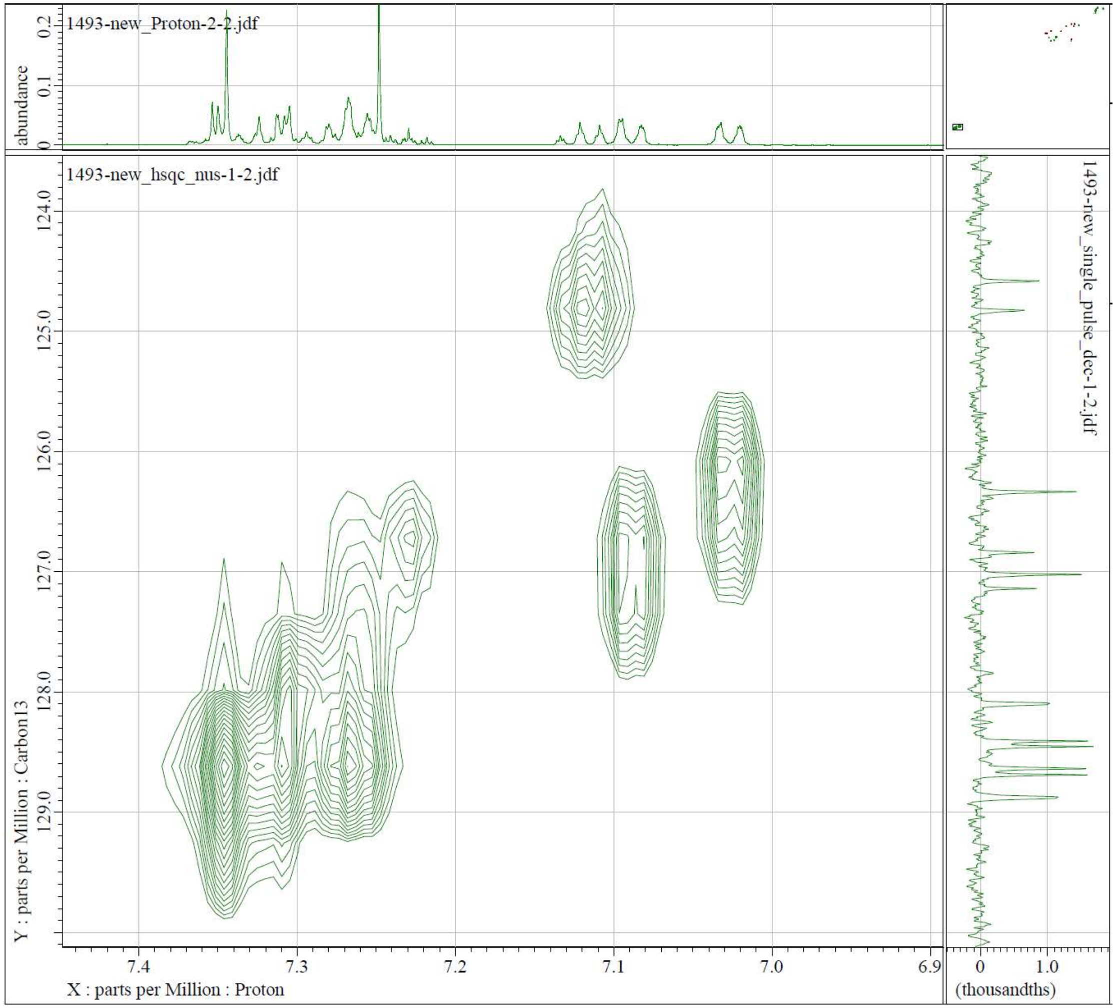

20; NOE

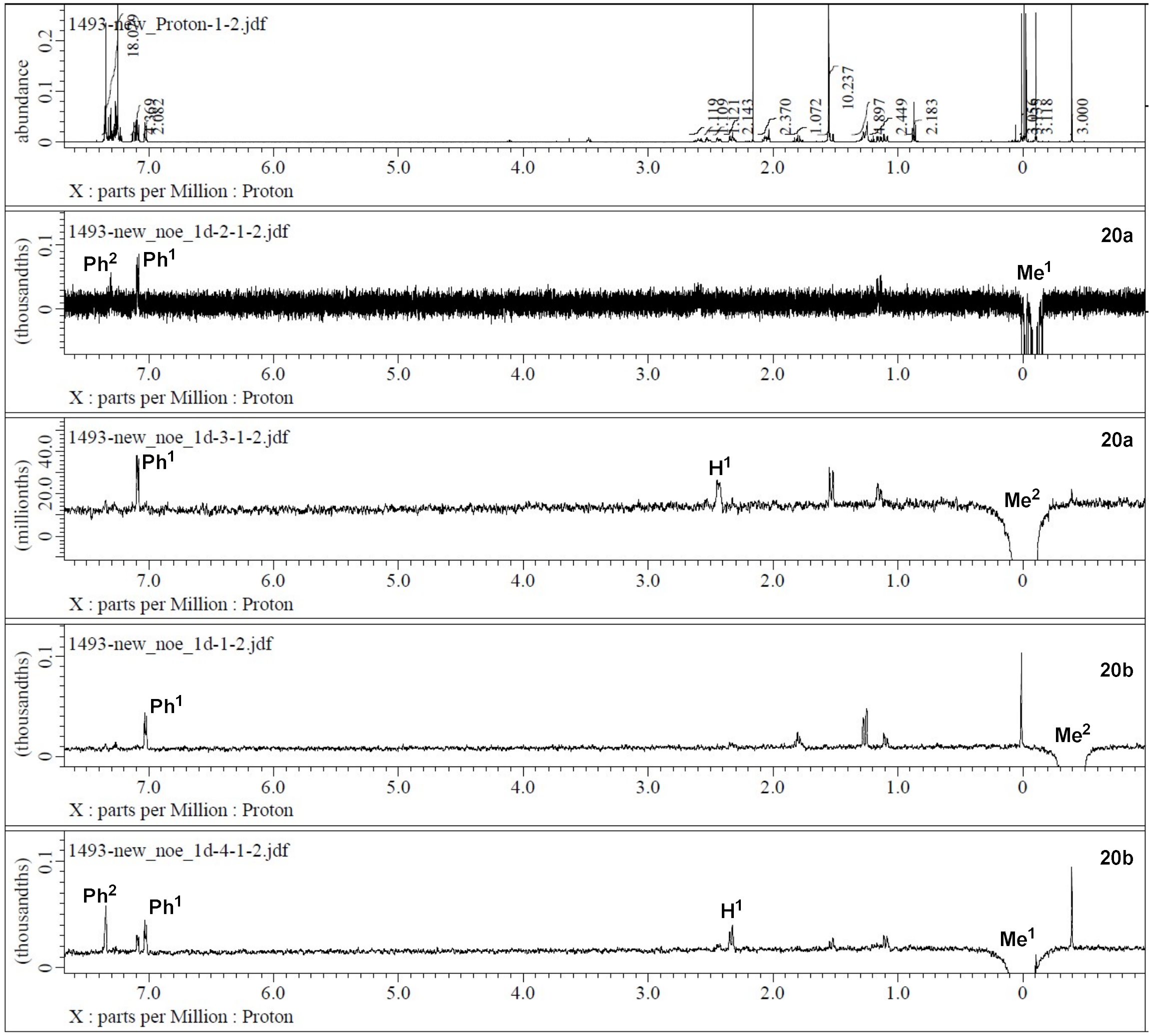
1493-new_Proton-1-2.jdf
abundance
X : parts per Million : Proton
1493-new_noe_1d-2-1-2.jdf
20a
Ph^{2}
Ph^{1}
Me^{1}
(thousandths)
1493-new_noe_1d-3-1-2.jdf
20a
Ph^{1}
H^{1}
Me^{2}
(millionths)
1493-new_noe_1d-1-2.jdf
20b
Ph^{1}
Me^{2}
1493-new_noe_1d-4-1-2.jdf
20b
Ph^{2}
Ph^{1}
H^{1}
Me^{1}
